# Oxidative Cleavage of Alkenes by O_2_ with a Non-Heme Manganese Catalyst

**DOI:** 10.1021/jacs.1c05757

**Published:** 2021-06-23

**Authors:** Zhiliang Huang, Renpeng Guan, Muralidharan Shanmugam, Elliot L. Bennett, Craig M. Robertson, Adam Brookfield, Eric J. L. McInnes, Jianliang Xiao

**Affiliations:** † Department of Chemistry, University of Liverpool, Liverpool L69 7ZD, U.K.; ‡ Department of Chemistry and Photon Science Institute, The University of Manchester, Manchester M13 9PL, U.K.

## Abstract

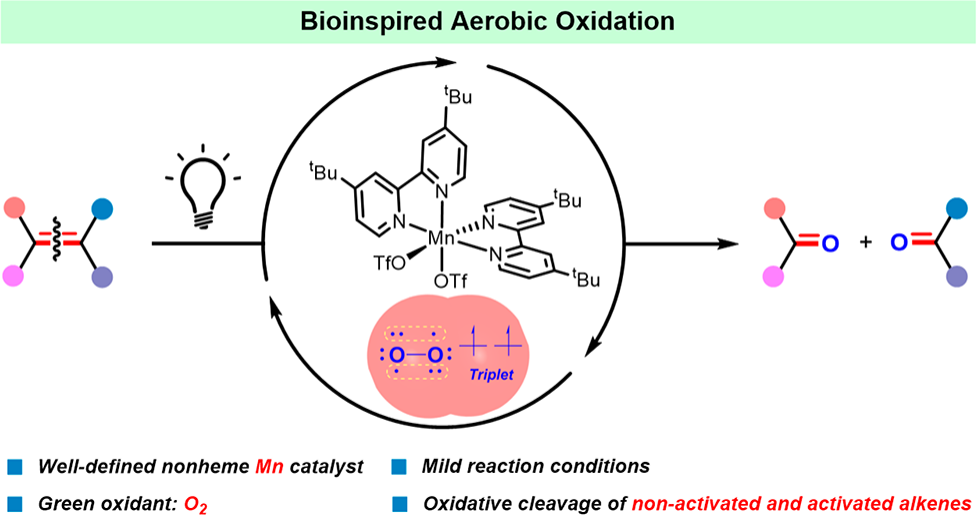

The oxidative cleavage of C=C double bonds with molecular oxygen to produce carbonyl compounds is an important transformation in chemical and pharmaceutical synthesis. In nature, enzymes containing the first-row transition metals, particularly heme and non-heme iron-dependent enzymes, readily activate O_2_ and oxidatively cleave C=C bonds with exquisite precision under ambient conditions. The reaction remains challenging for synthetic chemists, however. There are only a small number of known synthetic metal catalysts that allow for the oxidative cleavage of alkenes at an atmospheric pressure of O_2_, with very few known to catalyze the cleavage of nonactivated alkenes. In this work, we describe a light-driven, Mn-catalyzed protocol for the selective oxidation of alkenes to carbonyls under 1 atm of O_2_. For the first time, aromatic as well as various nonactivated aliphatic alkenes could be oxidized to afford ketones and aldehydes under clean, mild conditions with a first row, biorelevant metal catalyst. Moreover, the protocol shows a very good functional group tolerance. Mechanistic investigation suggests that Mn–oxo species, including an asymmetric, mixed-valent bis(μ-oxo)-Mn(III,IV) complex, are involved in the oxidation, and the solvent methanol participates in O_2_ activation that leads to the formation of the oxo species.

## Introduction

**Scheme 1:**
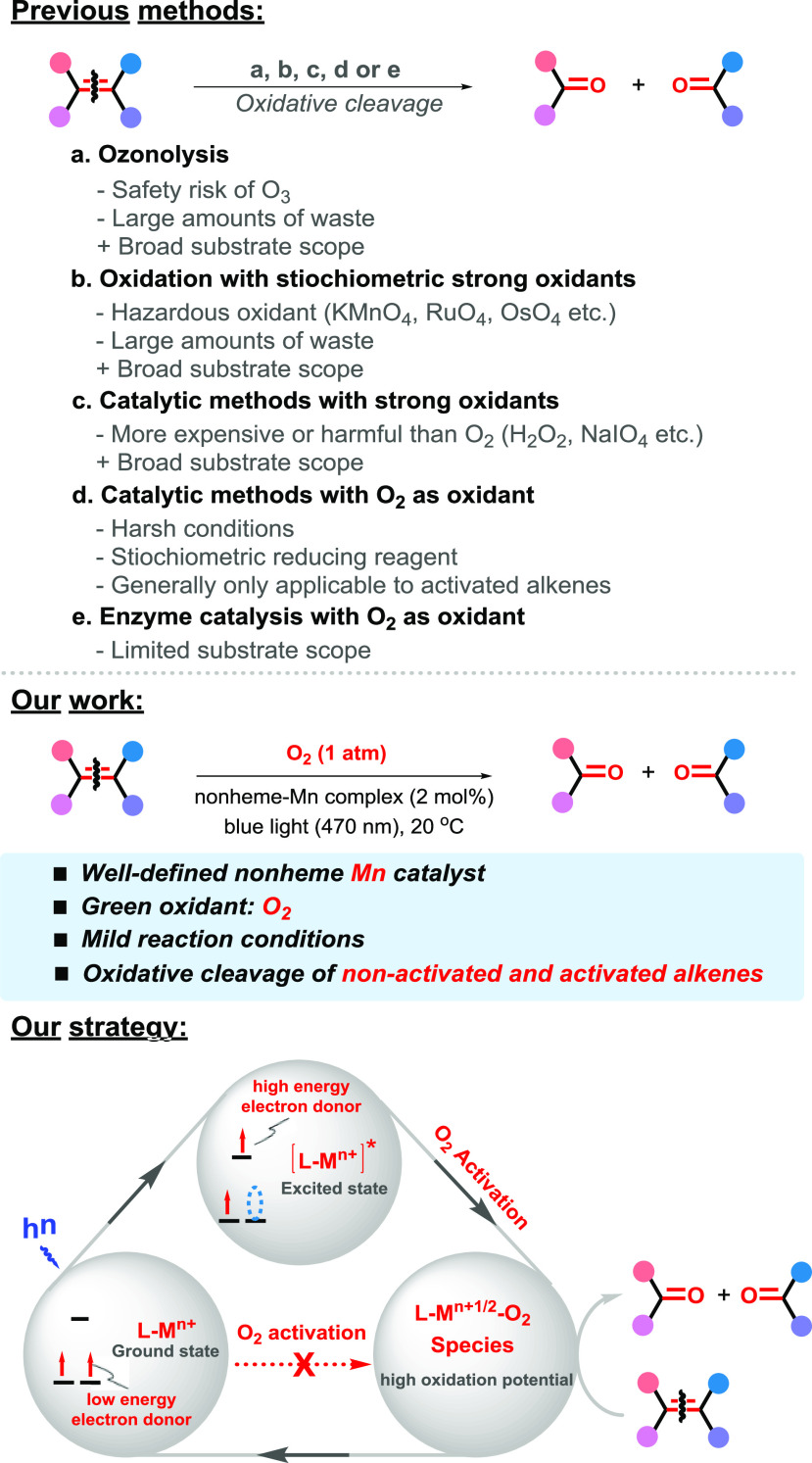
Methods for the Oxidative Cleavage of Alkenes and Schematic Showing of a Visible-Light-Driven Dioxygen-Activation Strategy for the Reaction of O_2_ with a Metal Catalyst

Alkenes are one of the most important chemicals on which the chemical industry is built. Apart from those that are derived from oil and natural gas in immense quantities, alkenes are widespread in drugs, natural products, polymers, and countless organic chemicals.^1^ The C=C double bonds thus provide a ubiquitous functionality for accessing bespoke chemicals. In this context, oxidative cleavage of C=C double bonds to the corresponding carbonyls is of particular interest, as ketones and aldehydes are one of the few most often used functionalities in organic synthesis.^1a^ Indeed, oxidative cleavage of alkenes has been practiced for over a century in research laboratories and on various industrial scales.^2^ The standard method for the direct oxidative cleavage of alkenes is ozonolysis (Scheme 1a),^3^ which is widely performed by the pharmaceutical and fine chemical industries.^4^ However, the use of ozone as oxidant is associated with intrinsic safety issues, the need for specialty equipment, and generation of over stoichiometric amounts of waste.^5^ Alternative oxidants, such as *m*-chloroperoxybenzoic acid, PhIO/HBF_4_, KMnO_4_, CrO_2_Cl_2_, RuO_4_, and OsO_4_, are well-known to selectively transform alkenes into carbonyls,^2b,6^ but they are expensive and/or toxic, while creating waste (Scheme 1b).

In response to the mounting environmental and safety issues, the past four decades have witnessed the study of numerous transition metal catalysts for oxidative cleavage of alkenes (Scheme 1c,d).^2c,6,7^ More recently, organo- and photocatalytic methods have been put forward.^7a,8^ Although various mild oxidants have been examined, the use of O_2_ remains of utmost interest, as it is the most easily available, least expensive, and cleanest oxidant. Pleasingly, a number of the reported methods now allow O_2_ to be used as the oxidant under various conditions.^7a,9^ Notwithstanding the progress made, almost all the methods reported so far can only deal with activated alkenes, such as styrene and derivatives. *In particular, until now, there appear to be no known catalysts that can be used to cleave the more spread, nonactivated aliphatic alkenes by using atmospheric O*_2_, *while showing a substrate scope of potential for organic synthesis*. To the best of our knowledge, there are only three reports in the literature that describe the oxidative cleavage of a few nonactivated aliphatic alkenes with O_2_, two of which necessitated 3–5 equiv of reductant (Mn and Co catalysts) while the third relied on a Pd catalyst with harsh conditions (2% Pd, 8 atm O_2_, 100 °C, 24 h).^10^ In addition to the chemical methods, enzymatic methods have been explored;^6b^ however, they are limited by substrate specificity (Scheme 1e). Following on from our recent study of aerobic cleavage of styrene derivatives with molecular iron and copper catalysts,^9a,11^ herein, we report that *the merger of a non-heme Mn(II) catalyst with visible light enables the oxidative cleavage of both aromatic and aliphatic alkenes under 1 atm of O*_2_*at room temperature* (Scheme 1).

Being one of the most abundant, inexpensive, and environmentally friendly metals on earth, manganese has been extensively studied for biomimetic O_2_ activation in the past four decades or so.^12^ The combination of a base metal catalyst with O_2_ would provide an ideal “oxygenase-like” synthetic protocol for oxidative cleavage of alkenes. However, “despite O_2_ activation at Fe or Mn being a prominent target for more than 40 years, there are still relatively few complexes that activate O_2_ in a rationally designed and controlled manner” and particularly, the use of benign and inexpensive O_2_ for selective oxidation with the biologically relevant Mn remains “a significant challenge for the synthetic chemist.”^12b^ This may be partly due to the different oxidation potentials required for oxidizing a substrate versus activation of O_2_.

Photoirradiation has been explored to tune the redox potential of metal catalysts.^13^ Bearing in mind the remarkable property of a photoexcited catalyst being both more oxidizing and more reducing than its ground state, we envisaged a visible-light-driven dioxygen activation strategy for the oxidative cleavage of alkenes with metal catalysts (Scheme 1). First, a molecular metal catalyst is excited by visible light to alter its redox potential. The more reducing excited state could then activate O_2_ to afford a more-oxidizing high-valent metal–oxygen species, which could react with an alkene, leading to its oxidation. In pioneering studies by Goldberg et al., light has been shown to promote catalytic C–H oxidation with Mn(III) complexes.^12b,14^

## Results and Discussion

### Reaction Development

**Scheme 2:**
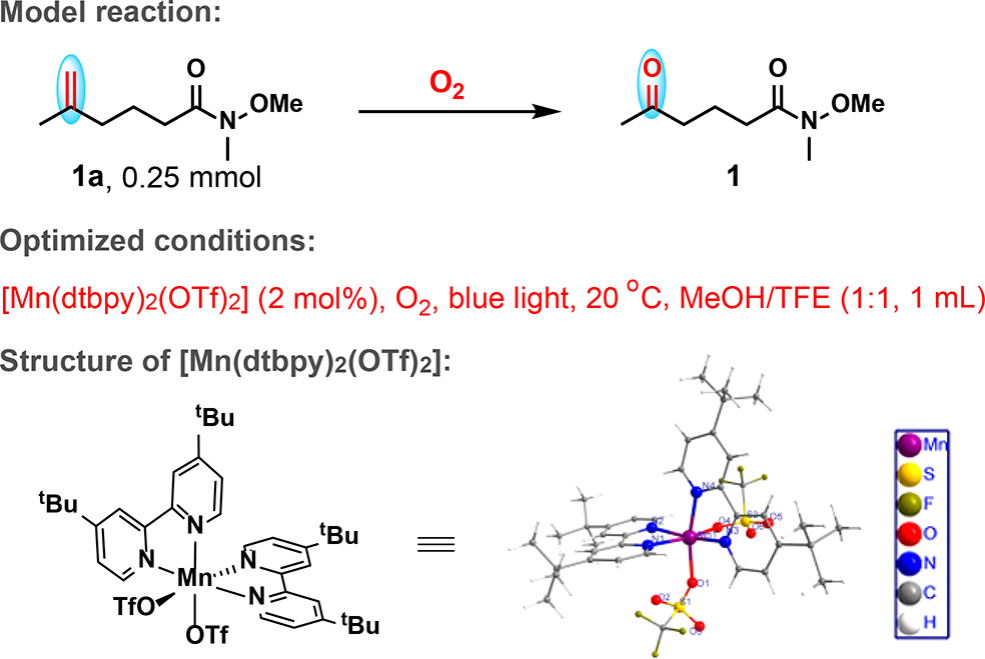
Optimized Conditions for the Oxidative Cleavage of **1a** by O_2_ and the X-ray Structure of [Mn(dtbpy)_2_(OTf)_2_] See the Supporting Information for details of optimization.

We set out to examine the oxidation of an aliphatic alkene, *N*-methoxy-*N*,5-dimethylhex-5-enamide (**1a**) (Scheme 2). To produce activated oxygen species from O_2_ that can cleave the C=C double bond, a range of open-shell transition-metal complexes and metal salts in combination with polydentate nitrogen ligands were examined (Table S1 in the Supporting Information). Delightfully, when the reaction was performed with Mn(OTf)_2_ and 2,2′-bipyridine type ligands under blue light irradiation (470 nm, 9 W) in MeOH at 20 °C, the desired product **1**, resulting from the C=C double bond cleavage, was obtained (Table S1, entries 1–12). Screening of ligands revealed 4,4′-di-*tert*-butyl-2,2′-dipyridine (dtbpy) to be most effective, affording **1** in 32% yield (Table S1, entry 6). However, its combination with other metal salts, such as Cu(OTf)_2_, Fe(OTf)_2_, and CoCl_2_, was ineffective, affording no target product (Table S1, entries 13–15). Notably, methanol as solvent is critical; no conversion of **1a** was observed when 1,2-dichloroethane, tetrahydrofuran, benzene, ethanol, isopropanol, or acetonitrile was utilized (Table S1, entries 16–21). Further optimization shows that a mixture of MeOH and 1,1,1-trifluoroethane (TFE) (1:1 volume) is optimal, boosting the yield of **1** to 62% (Table S1, entry 22). Note that blue light is essential for the oxidation, without which the chemistry could not proceed even at 70 °C (Table S1, entries 25 and 26; also see Figure S12).

The structure of the manganese complex [Mn(dtbpy)_2_(OTf)_2_], resulting from the reaction of Mn(OTf)_2_ with the dtbpy ligand, has been determined by X-ray diffraction (Scheme 2 and Scheme S1). The complex shows a somewhat distorted octahedral geometry with expected Mn–N bond distances (Tables S7 and S8). It is worth noting that the isolated [Mn(dtbpy)_2_(OTf)_2_] displayed a similar activity as that prepared in situ (Table S1, entries 22 and 23), indicating that it is formed in the *in situ* reaction. Our subsequent investigation was centered around using [Mn(dtbpy)_2_(OTf)_2_] (2 mol %) as catalyst and O_2_ (1 atm) as oxidant in MeOH/TFE (1:1) with continuous blue light irradiation at room temperature.

### Scope of Reaction

**Scheme 3:**
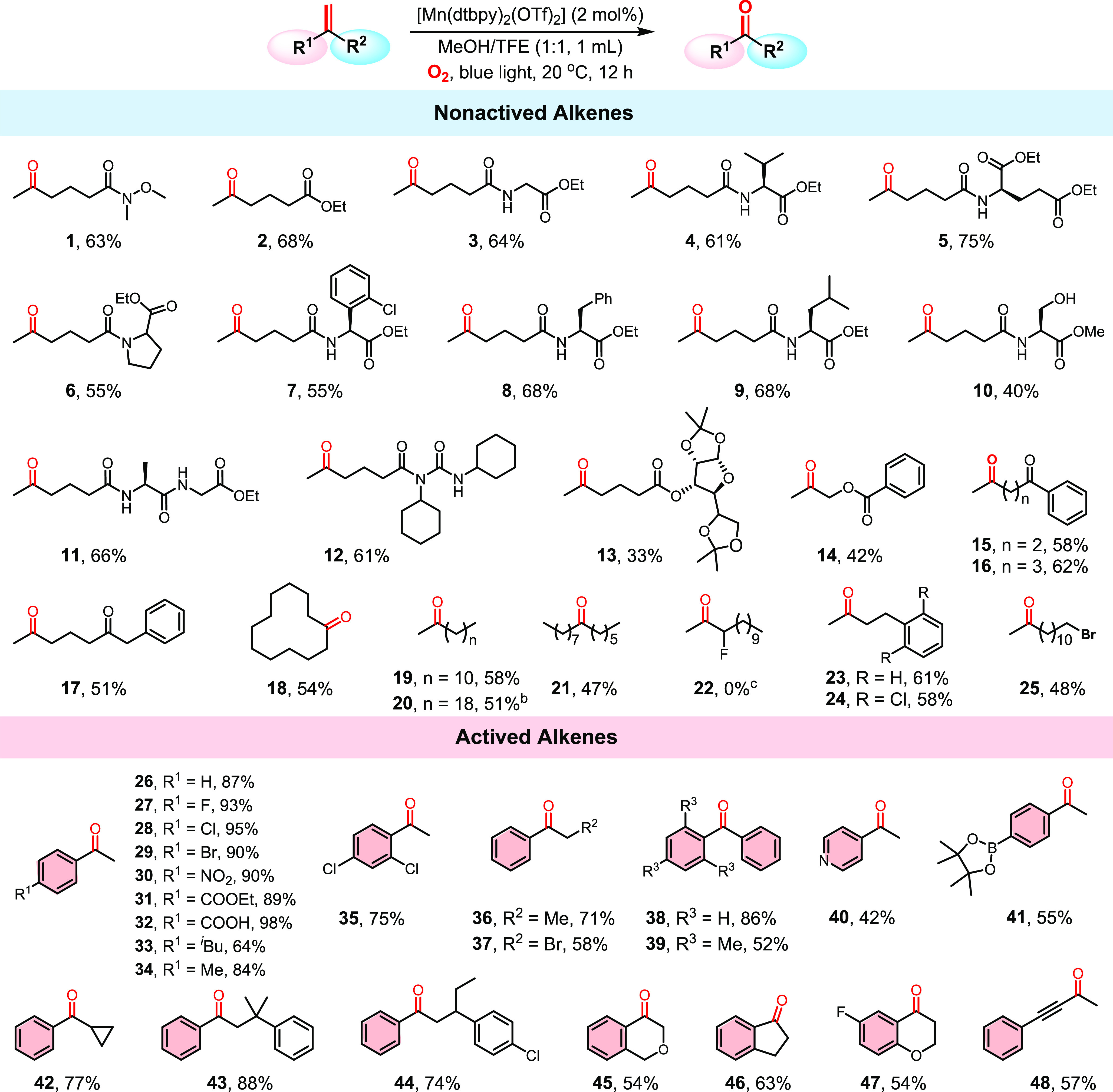
Oxidation of 1,1-Disubstituted Alkenes All the reactions were performed with alkene (0.5 mmol), [Mn(dtbpy)_2_(OTf)_2_] (2 mol %), in MeOH/TFE (1:1, 2 mL) with blue light irradiation (9 W, 470 nm) at 20 °C under O_2_ (1 atm) for 12 h. Isolated yields are given. 1 mL of EtOAc was added. Starting material was fully recovered.

To demonstrate the generality of this photo-Mn catalytic system, we first examined a series of nonactivated 1,1-disubstituted aliphatic alkenes. As is shown in Scheme 3, a wide variety of such olefins were selectively converted to the corresponding ketones. Thus, aliphatic alkenes bearing amide, ester, or amino acid functionalities are all suitable, affording the desired products in moderate yields (**1**–**10**). Dipeptide, urea, or glucose-containing alkenes could be oxidized selectively as well (**11**–**13**), showing potential applications in bioconjugate chemistry. Olefins with a synthetically important carbonyl functionality could be tolerated (**14**–**17**), and meanwhile, olefins without other functional groups were also oxidatively cleaved to furnish ketone products in good yields (**18**–**21** and **23**). However, 3-fluoro-2-methyltridec-1-ene, in which the C=C double is deactivated by an adjacent electron-withdrawing fluorine group, could not afford any cleavage product (**22**), highlighting the sensitivity of the catalyst to substrate electronic effect. In contrast, the electron-withdrawing, but well-separated, bromide did not stop the formation of ketone **25**. The presence of halogen substituents in the oxidation products, such as chloride in **24** and bromide in **25**, makes the ketone products more versatile in further applications. Still worth noting is that the oxidation-susceptible benzylic C–H bond (viz. **23**, **24** and below) remained intact during the oxidation, showing the protocol to be chemoselective. The catalysis thus makes 1,1-disubstitued C=C bonds an easily accessible latent carbonyl functionality.

As maybe expected, this photo-Mn enabled protocol is well suited for activated alkenes. Thus, as is seen in Scheme 3, styrene and related derivatives were oxidatively cleaved to yield carbonyl compounds in a highly selective manner and good to excellent yields. Moreover, it shows an excellent functional group compatibility, with halogen, nitro, ester, acid, cyclopropyl, alkynyl, pyridine, and boronic ester units all tolerated (**26**–**48**). Both benzylic and tertiary C–H bonds, which are prone to oxidation, were also tolerated during the oxidation (**42**, **44**–**46**), adding more support to the high chemoselectivity mentioned above. A gram-scale oxidative cleavage was also demonstrated. Under the standard conditions but with 0.01% mol (0.9 mg) of catalyst, α-methylstyrene (1.18 g, 10 mmol) was oxidized to ketone **26** with a turnover number of 4000 in 12 h (Scheme S2). It is noted that this new strategy allows for milder reaction conditions in comparison with the previously reported Fe- and organo-catalytic methods,^8b,8d,9a,9b^ and shows improved compatibility with electron-withdrawing groups in comparison with the photocatalytic method developed by Wang et al.^8c^

The oxidation was then extended to monosubstituted, internal disubstituted, trisubstituted, and tetrasubstituted alkenes as well as dialkenes. As shown in Table 1, a diverse range of such alkenes can be oxidized to afford the corresponding carbonyls or their methanol-protected form, dimethyl acetals. Thus, the unsaturated fatty acid derivative **49** was cleaved into an aldehyde, which was *in situ* converted into the acetal **50**. Cyclooctene **51** gave rise to two isolable, valuable oxidation products **52** and **53** in 41% overall yield, the former of which is likely to result from the oxidation of the initially formed aldehyde. Worth noting is that the formation of the epoxide **53** may indicate the involvement of a high valent Mn–oxo species during the oxidation.^15^ The C=C double bonds of trisubstituted alkenes, such as those in **54**, **56**, and **58**, were all oxidized, affording carbonyl products and derivatives. For instance, the cleavage of **58** led to benzophenone **38** and the overoxidation product of benzaldehyde, methyl benzoate **57**, in good yield (see the Supporting Information for details). The viability of the protocol in oxidatively cleaving tetrasubstituted alkenes is seen in the examples of **59**, **61**, and **62**, the isolated products being the ketones of higher molecular weight in the case of **59** and **61**. Note that the cyclopentene **62** underwent ring-opening, affording the diketone **16**. Interestingly, dialkenes are also viable for this transformation, with both the C=C double bonds selectively oxidized to afford the corresponding dicarbonyl compounds in good yields (**63**–**65** and **67**).

**Table 1:**
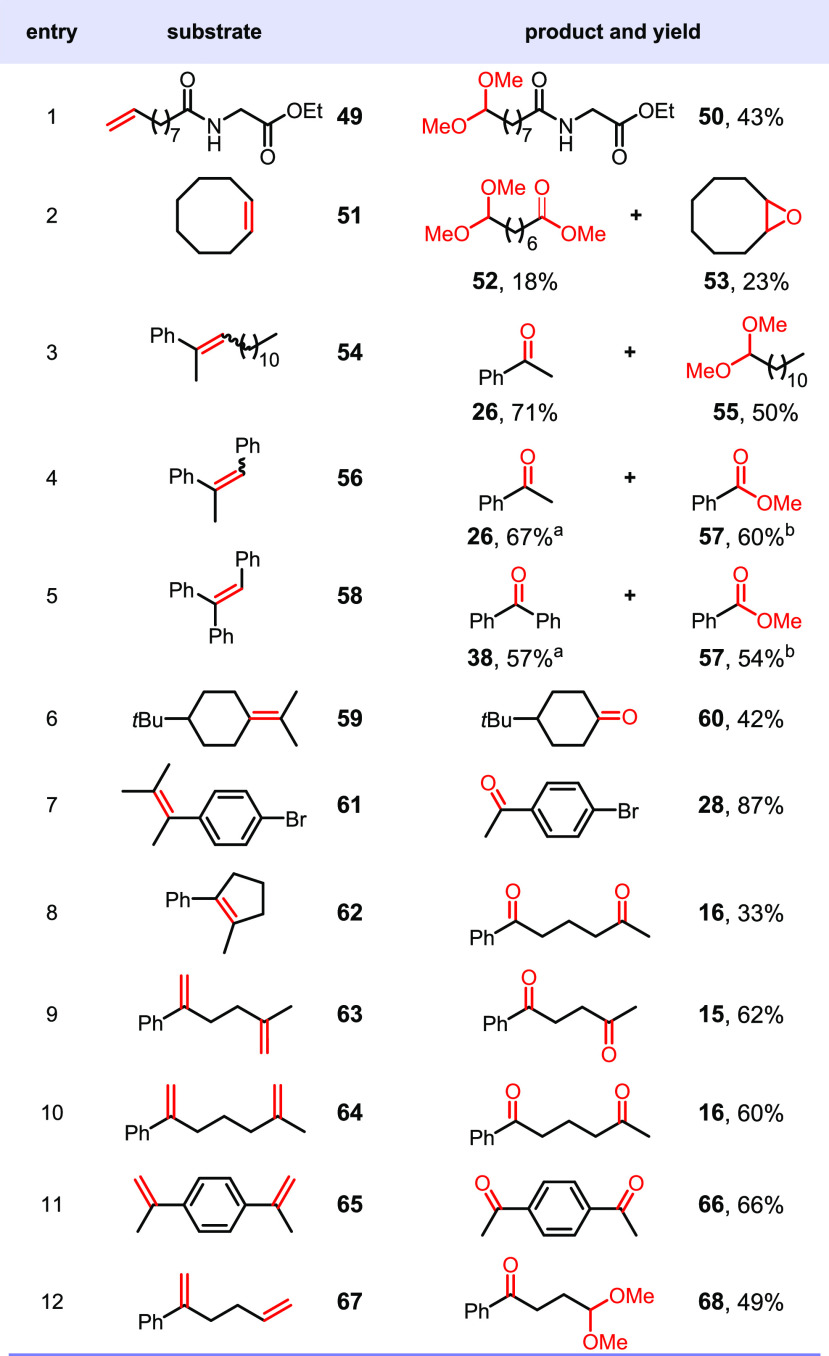
Oxidation of Mono-, Di-, Tri-, and Tetrasubstituted Alkenes and Dialkenes^a^

a All the reactions were performed with alkene (0.5 mmol), [Mn(dtbpy)_2_(OTf)_2_] (2 mol %), in MeOH/TFE (1:1, 2 mL) with blue light (9 W, 470 nm) at 20 °C under O_2_ (1 atm) overnight. Isolated yields are given.

b The yield was obtained by ^1^H NMR analysis with mesitylene as internal standard.

To further demonstrate the practical applicability of this photo-Mn protocol in aerobic oxidation of C=C double bonds, we attempted the oxidative cleavage of a wide range of natural products and their derivatives. As shown in Table 2, terpenes could be selectively oxidized into terpenoids, which are interesting chemical intermediates for fragrance (**70** and **72**). The nopinone **70**, produced industrially from ozonolysis of **69**, has been used to synthesize Nabilone,^16^ a synthetic cannabinoid used to treat nausea and vomiting during cancer treatment, and the prostaglandin D_2_ (PGD_2_) receptor antagonist S-5751.^17^ However, a detailed safety study of the industrial ozonolysis has revealed the high risk of explosion and a runaway reaction in this multistep process (−57, −27, 0, and finally 25 °C).^17^ (+)-Nootkatone **73**, a sesquiterpene ketone from the heartwood of yellow cedar, was cleaved to afford the corresponding methyl ketone in good yield. Interestingly, although there are two C=C double bonds in the structure of (+)-Nootkatone, only the electron-rich *exo* double bond was selectively oxidized. The α-cyperone derivative **75** and the 20-methylpregna-4,20-diene-3,6-dione **77**, which are active pharmaceutical ingredients, underwent oxidative cleavage to give the desired product **76** and **78** in a highly chemoselective manner. Vitamin K1 could also be oxidized efficiently, affording the valuable hexahydrofarnesyl acetone **80**, a member of sesquiterpenoids and used in jasmine compositions.^18^ These reactions and those above are often accompanied by some side products (for examples, see the Supporting Information).

**Table 2:**
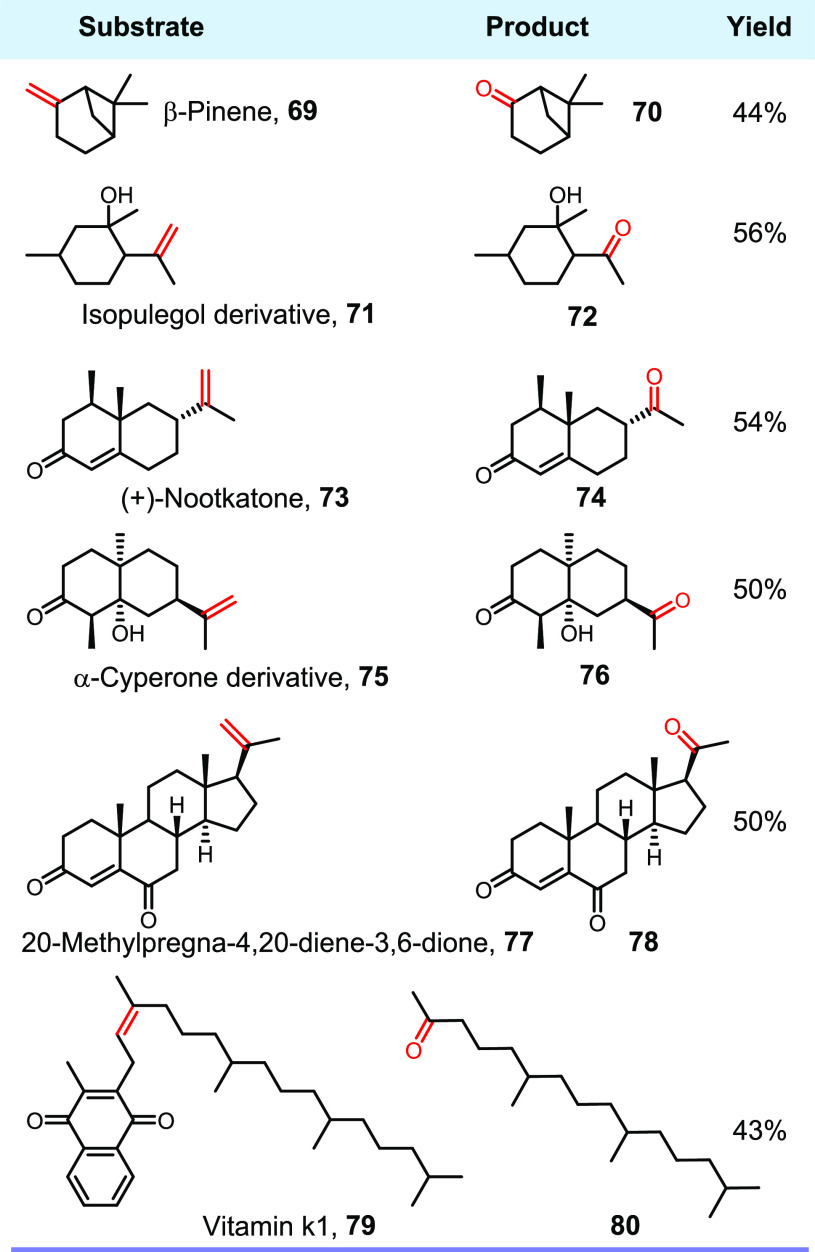
Oxidation of Natural Products or Their Derivatives^a^

a All the reactions were performed with alkene (0.5 mmol), [Mn(dtbpy)_2_(OTf)_2_] (2 mol %), in MeOH/TFE (1:1, 2 mL) with blue light (9 W, 470 nm) at 20 °C under O_2_ (1 atm) overnight. Isolated yields are given.

### Mechanistic Possibilities

While the detailed mechanism of the oxidation remains largely unclear, we have performed a range of experiments, aiming to shed light on possible reaction pathways. As reactions between olefins and singlet oxygen (^1^O_2_), which can be generated from O_2_, light, and a photosensitizer, are well-known to give oxidative cleavage products,^19^ we thought that it is important to first determine whether ^1^O_2_ plays a role in the photo-Mn enabled oxidative cleavage. The ^1^O_2_ trap 9,10-diphenylanthracene (DPA) is known to react rapidly with ^1^O_2_ to give an endoperoxide product (*k* ≈ 1.3 × 10^6^ M^–1^ s^–1^).^20^ When α-methylstyrene was subjected to the standard oxidation conditions but in the presence of DPA as a ^1^O_2_ trap, no endoperoxide was detected, and importantly, the expected cleavage product acetophenone **26** was formed in 70% yield (Scheme S3). In addition, when four well-known photosensitizers, eosin Y disodium salt, [Ru(bpy)_3_·6H_2_O], [Ir(dFppy)_3_], and Rose Bengal, which are all known to be capable of producing ^1^O_2_ under blue light irradiation,^21^ were used individually as replacement catalyst for the oxidative cleavage of **1a**, none were found to catalyze the reaction (Table S3). These observations indicate that the photo-Mn promoted oxidative cleavage of alkenes does not involve catalytic formation of ^1^O_2_.

**Figure 1:**
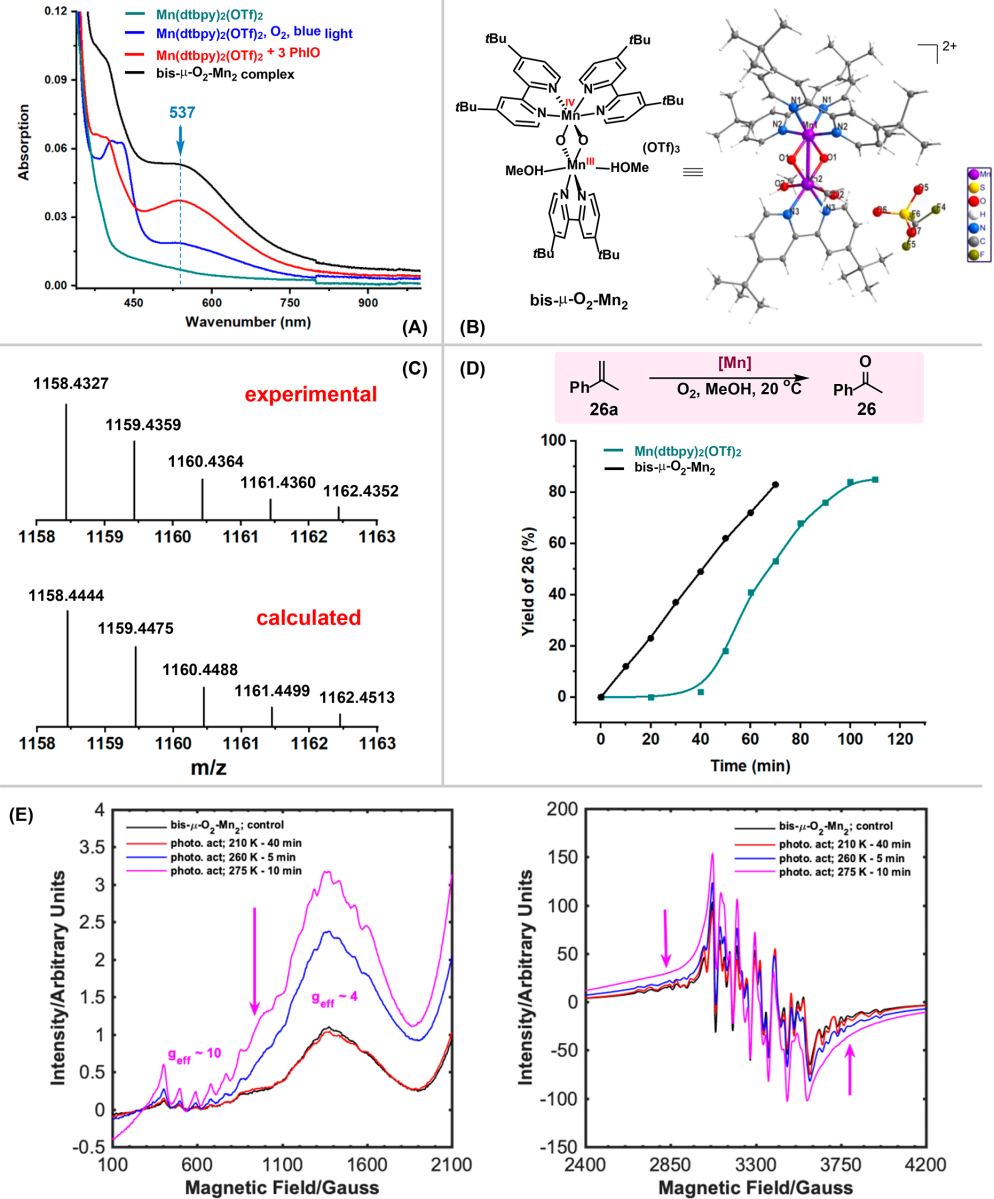
(A) UV–vis spectra of [Mn(dtbpy)_2_(OTf)_2_] with or without oxidants and of bis-μ-O_2_-Mn_2_ complex (0.25 mM, in MeOH). (B) X-ray structure of the bis-μ-O_2_-Mn_2_ complex (for details, see Figure S7 and section 9.2 in the Supporting Information). (C) HRMS spectrum of the bis-μ-O_2_-Mn_2_ complex formed in the *in situ* reaction of [Mn(dtbpy)_2_(OTf)_2_] with O_2_. (D) Kinetic behavior of the oxidation of α-methylstyrene catalyzed by [Mn(dtbpy)_2_(OTf)_2_] and bis-μ-O_2_-Mn_2_. (E) Perpendicular mode, CW-EPR spectra of the bis-μ-O_2_-Mn_2_ dimer (black trace) dissolved in cold (208 K) MeOH, followed by irradiation with blue light under air for 40 min (red trace), 5 min (blue), and 10 min (magenta) at the specified temperatures. The left-hand panel shows the zoomed-in *g*_eff_ = 10 and *g*_eff_ = 4–6 regions of the spectra in the right-hand panel. Conditions: MW power 10 dB, MA 5G, time constant 82 ms, conversion time 10 ms, sweep time 120 s, receiver gain 30 dB, average microwave frequency 9.385 GHz, temperature 20 K.

Bearing in mind that the oxidative catalysis in question likely involves high-valent Mn–oxygen species, we then followed the photopromoted Mn activation of O_2_ by UV–vis spectroscopy. As shown in Figure 1A, when the [Mn(dtbpy)_2_(OTf)_2_] complex was exposed to O_2_ under the irradiation of blue light for 1 h, a new absorption band at ∼537 nm appeared, indicative of the formation of a new Mn–oxygen species.^22^ In line with this conjecture, a color change from pale-yellow to greenish-brown was observed. Notably, both blue light and O_2_ are essential for the formation of this species; in the absence of either one, the complex remained unchanged. To help assign the photoinduced absorption, a well-studied oxidant, PhIO, was reacted with [Mn(dtbpy)_2_(OTf)_2_] in the dark under N_2_ (room temperature, MeOH solvent). Compared with the oxidation using O_2_, the reaction with the stronger oxidant PhIO was immediate, resulting in the same greenish-brown solution. As can be seen, an identical absorption band at 537 nm was observed (Figure 1A, red line), suggesting that the two conditions lead to the same Mn–oxygen species.

Delightfully, we were able to isolate the Mn–oxygen species and determine its structure by X-ray diffraction. As is shown in Figure 1B, it is a mixed-valent oxo-bridged binuclear Mn(III,IV) complex, [Mn(dtbpy)_2_(μ-O)_2_Mn(dtbpy)(MeOH)_2_](OTf)_3_ (bis-μ-O_2_-Mn_2_). Unlike common bis-μ-oxo Mn(III,IV) dimers, the complex features two asymmetric metal centers in significantly different environments, with one ligated by two dtbpy ligands while the other by only one. The former is likely to be Mn(IV) and the latter Mn(III), judging from the Mn–O and Mn–N bond distances.^23^ Most revealing is that the isolated complex shows a clear UV–vis absorption band at 537 nm (Figure 1A, black line), suggesting that the same oxo-bridged species is formed when [Mn(dtbpy)_2_(OTf)_2_] is irradiated under O_2_ or reacted with PhIO under N_2_, albeit with differing concentrations. HRMS analysis also indicates that these two different reactions give rise to the same Mn(III,IV) dimer (Figure S8). Figure 1C shows the experimental and calculated mass spectrum of the molecular ion resulting from the loss of a HOTf and a ^–^OTf fragment from the parent dimer.

Furthermore, the methanol solutions of bis-μ-O_2_-Mn_2_ before and after blue light irradiation were monitored by electron paramagnetic resonance (EPR) spectroscopy. As shown in Figure S13, the methanol solution of bis-μ-O_2_-Mn_2_ gives a complex multiline spectrum, which is consistent with the *S* = 1/2 ground state of the antiferromagnetically coupled Mn(III/IV) dimer.^24^ Notably, at room temperature, the Mn(III/IV) dimer partially decomposes to Mn(II), which could be further oxidized to the dimer by air under irradiation, and we see clear signatures of Mn(III) in parallel mode EPR spectra (Figure S14). Significantly, irradiating the MeOH-dissolved Mn(III/IV) dimer with blue light increases the intensity of a signal in the *g*_eff_ = 4 region, with a noticeable enhancement of hyperfine structure (ca. 90 G), along with the development of a pronounced shoulder at ca. 1000 G, while the multiline structure in the *g* = 2 region is lost (Figure 1E). This could be consistent with generation of high-valent species from the Mn(III/IV) dimer. We see clear evidence of Mn(III), and although inconclusive, a Mn(IV) monomer (*S* = 3/2) with large zero-field splitting would be expected to give signals in the *g*_eff_ = 4–6 region: similar signals have previously been assigned to Mn(IV).^25^

The time course of the oxidation of a model substrate α-methylstyrene **26a** reveals additional insight. As shown in Figure 1D, when the model reaction was performed by using [Mn(dtbpy)_2_(OTf)_2_] under the standard conditions, a clear induction period of ca. 40 min was observed (green line). However, when the oxidation was performed with bis-μ-O_2_-Mn_2_ as catalyst, the induction period disappeared, and a zero-order kinetic profile on the substrate was observed at least up to 60% conversion. Notably, when the Mn–oxygen species generated from PhIO was employed as the catalyst, the induction period largely disappeared as well (Figure S9). These observations point to that under the catalytic conditions the precatalyst [Mn(dtbpy)_2_(OTf)_2_] is first converted by O_2_ into the bis-μ-O_2_-Mn_2_ complex in an induction period. We suspect that the oxo dimer is, however, catalytically inactive;^26^ under continuous irradiation, it may undergo rate-determining photolysis to a monomeric Mn-oxo species that starts the catalytic turnover,^27^ leading to the zero-order kinetics. Indeed, when the bis-μ-O_2_-Mn_2_ complex was used as catalyst for the aerobic oxidation of **26a**, light was still required; in its omission, the oxidation stalled (Scheme S4). This monomeric oxo species could be the high-valent species detected by the EPR.

Because MeOH is critical for this aerobic oxidation reaction, we suspected that it might be involved in the O_2_ activation that leads to the formation of the bis-μ-O_2_-Mn_2_ complex. To gain insight into the possible role of MeOH, the kinetic isotope effect was examined in the oxidative cleavage of **26a**. As is shown in Figure S10, CH_3_OH and CH_3_OD gave the same introduction period and same rate of **26a** oxidation. On the other hand, CD_3_OD afforded an induction period shorter by ca. 10 min but a similar rate of oxidizing **26a**. This somewhat surprising inverse kinetic isotope effect indicates that, apart from coordination to the metal center (Figure 1B), MeOH participates in the photoassisted oxidation of [Mn(dtbpy)_2_(OTf)_2_] by O_2_ to give the dioxo–Mn(III,IV) dimer, presumably by acting as an electron donor; however, the cleavage of C–H and O–H bond of MeOH is not the rate-determining step of its formation.

**Scheme 4:**
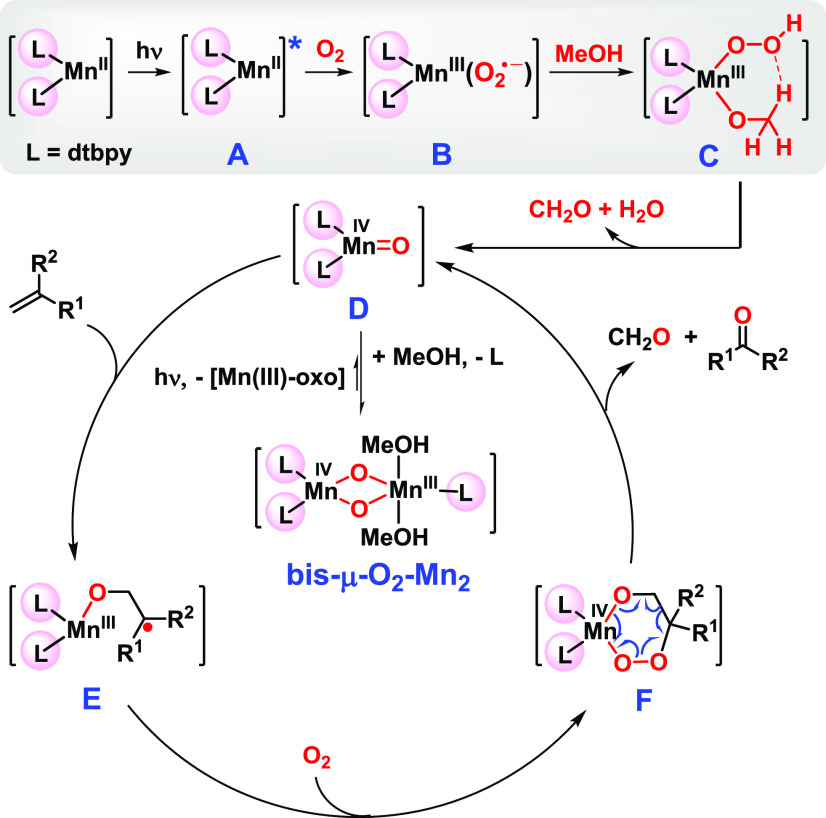
A Putative Mechanism for the Photo-Mn Promoted Oxidative Cleavage of Alkenes

Based on the above results and literature precedents, a putative mechanism for the oxidative cleavage is tentatively suggested (Scheme 4). As illustrated for a 1,1-disubstituted alkene, the Mn(II) catalyst is first excited by blue light to afford a more active Mn(II) intermediate **A**, which reacts with O_2_ to form a Mn(III)–superoxo species **B**.^12b,28^ Hydrogen abstraction by **B** from methanol, presumably coordinated to Mn(III), gives rise to an intermediate **C**, which then transfers to the Mn(IV)–oxo **D** with release of formaldehyde (was detected by GC-MS, see Figure S17) and water.^28c^ While catalytically active, the monomeric **D** may easily transfer to the more stable, observable bis-μ-O_2_-Mn_2_ complex. Under the blue light irradiation, the oxo dimer dissociates, presumably reversibly, to **D** and a Mn(III) species. Having a radical character, **D** adds to the alkene to produce a new radical species **E**, which would be active toward O_2_, forming a six-membered metal–peroxo species **F**. The unstable species **F** would rapidly decompose to form the ketone product and formaldehyde while regenerating **D**. In the whole catalytic reaction, bis-μ-O_2_-Mn_2_ is an off-cycle species. Its formation via **C** and **D** involves the participation of methanol as an electron and hydrogen donor, and the inverse kinetic isotope effect when using CD_3_OD may have several origins.^29^ Continuous irradiation is necessary, as the active species **D** could undergo dimerization with off-cycle Mn(III) species to re-form the oxo dimer. A similar mechanistic pathway is seen in the work of Bruice on oxidative cleavage of C=C double bonds by a Mn(IV)=O porphyrin complex.^15c^

Additional support for the mechanism is found in the ^16^O_2_–^18^O_2_ tracer experiments. As shown in Scheme S6, when the alkene **62** was oxidized with pure ^16^O_2_ or ^18^O_2_, only ^16^O- or ^18^O-labeled cleavage product was observed; however, when a mixture of ^16^O_2_–^18^O_2_ was used, all statistically possible products were formed. The formation of the statistical mixture is consistent with what is expected from the mechanism; that is, the two oxygen atoms in the product **16** originate from two different O_2_ molecules, while ruling out a pathway where the cleavage product results from an dioxetane intermediate.^9a^

## Conclusions

In summary, a non-heme Mn(II)-catalyzed aerobic cleavage of activated as well as nonactivated C=C double bonds has been established for the first time. Both aliphatic and aromatic alkenes could be oxidized with this “oxygenase-like” protocol, affording synthetically versatile carbonyl compounds from widespread olefins. The oxidation proceeds under an atmospheric O_2_ pressure at room temperature, providing an atom-economic, environment- and user-friendly approach for oxidative cleavage of alkenes. Mechanistic investigations indicate that an asymmetric, mixed-valent bis(μ-oxo)dimanganese(III,IV) complex is formed as a key but off-cycle intermediate in the catalysis. Notably, blue light irradiation of the precatalyst and participation of MeOH are all key elements in the activation of O_2_ and formation of the oxo dimer, and light is necessary to generate the active Mn(IV)=O species and sustain catalytic turnover.

## Supporting Information Available

Experimental details and procedures, optimization studies, mechanistic experiments, crystallographic data, and spectral data for all compounds (PDF)

## Accession Codes

CCDC 1997610 and 2050295 contain the supplementary crystallographic data for this paper. These data can be obtained free of charge via www.ccdc.cam.ac.uk/data_request/cif, or by emailing data_request@ccdc.cam.ac.uk, or by contacting The Cambridge Crystallographic Data Centre, 12 Union Road, Cambridge CB2 1EZ, UK; fax: +44 1223 336033.
