# Supplementary material for: Oxidative Cleavage of Alkenes by O2 with a Non-Heme Manganese Catalyst
Source: J Am Chem Soc. 2021 Jun 23;143(26):10005–13. doi: 10.1021/jacs.1c05757 (PMC8297864; doi:10.1021/jacs.1c05757)
Supplement: Supplementary file 1 — ja1c05757_si_001.pdf [file ja1c05757_si_001.pdf]

Supporting Information

Oxidative Cleavage of Alkenes by O_2 with a Non-Heme Manganese Catalyst

Zhiliang Huang,[1] Renpeng Guan,[1] Muralidharan Shanmugam,[2] Elliot L. Bennett,[1] Craig M. Robertson,[1] Adam Brookfield,[2] Eric J. L. McInnes[2] and Jianliang Xiao[1,*]

[1] Department of Chemistry, University of Liverpool, Liverpool L69 7ZD, U.K.

[2] Department of Chemistry and Photon Science Institute, The University of Manchester, Manchester M13 9PL, U.K.

E-mail: j.xiao@liverpool.ac.uk

Contents

1. General information……………………………………….……….........S1
2. Information of the blue light photoreactor……….……………………….........S2
3. Preparation of substrates……………………..……………..…………....S3
4. General procedure for the aerobic oxidative cleavage of alkenes……...…..…...S9
5. Mechanistic investigations………………………………………….….…….....S18
6. Analytical data of products…………………………….………......…...S37
7. References……………………..………………………...………...….......S63
8. NMR spectra of products………………………………………………………S70
9. Crystallographic data……………………..……………………..………...….......S130

1. General information

All manipulations were carried out using standard Schlenk techniques. All glassware was oven dried at 120 °C for more than 1 hour prior to use. $Mn(OTf)_2$, 2,2,2-trifluoroethanol (TFE), anhydrous methanol, substituted 2,2'-bipyridines, methyltriphenylphosphonium bromide, 4-acetylbutyric acid, *β*-pinene, *α*-methylstyrene,

1-chloro-4-(prop-1-en-2-yl)benzene, 2,4-diphenyl-4-methyl-1-pentene, 1-methyl-4-(prop-1-en-2-yl)benzene, 1,1-diphenylethylene, trans-α-methylstilbene, (+)-Nootkatone and vitamin K1 were purchased from commercial suppliers and used without further purification. Unless otherwise noted, analytical grade solvents and commercially available reagents were used as received. Analytical thin-layer chromatography (TLC) was conducted with TLC Silica gel 60 F254 (Merck) and plates were revealed under UV irradiation, iodine, potassium permanganate or vanillin staining. Flash column chromatography was performed using Aldrich Silica Gel 60 and columns were packed according to the dry method and equilibrated with the appropriate eluent prior to use. HPLC grade solvents were used and the solvent mixtures used as eluent are understood as volume/volume. All new compounds were characterized by ^{1}H NMR, ^{13}C NMR and HRMS. The known compounds were characterized by ^{1}H NMR and ^{13}C NMR. The ^{1}H and ^{13}C NMR spectra were recorded on a Bruker Advance 400 NMR spectrometer at 400 MHz (^{1}H NMR) and 101 MHz (^{13}C NMR). The chemical shifts (δ) were given in part per million relative to internal tetramethylsilane (0 ppm for ^{1}H) and $CDCl_3$ (77.00 ppm for ^{13}C). Mass spectra were obtained by chemical ionization (CI) or electrospray ionization (ESI) at the Analytical Services of the Chemistry Department or the Materials Innovation Factory, University of Liverpool. UV-Vis spectra were measured on the Agilent Cary 5000 at the Materials Innovation Factory, University of Liverpool.

2. Information of the blue light photoreactor

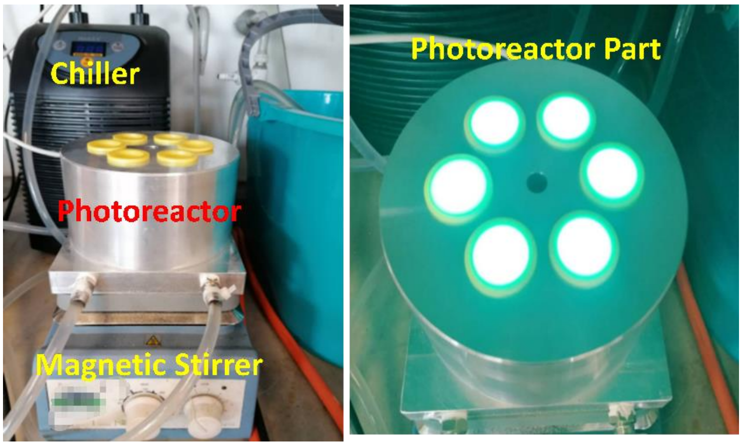

Fig. S1 Photoreactor used in this study.

Information of the blue LEDs: 2.95 V Blue LED SMD, Lumileds LUXEON Rebel LXML-PB01-0040; dominant wavelength or peak wavelength (minimum: 460 nm, typical: 470 nm, maximum: 485 nm); typical spectral half-width (20 nm); typical temperature coefficient of dominant or peak wavelength (0.05 nm/°C); typical total included angle (160°); typical view angle (125°). Each hole on the photoreactor was fixed with three LEDs, with total power being 9 W for each reaction tube.

3. Preparation of substrates

3.1 Synthesis of N-methoxy-N,5-dimethylhex-5-enamide (1a)

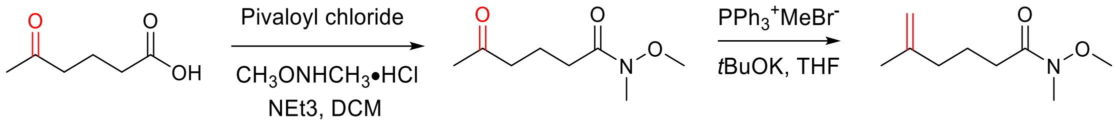

S1: To an oven dried 100 mL round bottom flask, 4-acetylbutyric acid (1.0 equiv.) and DCM (3 mL/mmol) was added under N_2 atmosphere. The resulting solution was cooled to 0 °C and stirred for 15 mins. Pivaloyl chloride (1.0 equiv.) and triethyl amine (1.1 equiv.) were added sequentially, and the resulting mixture was allowed to stand at 0 °C for 1 h. To this mixture, triethyl amine (2.0 equiv.) and N,O-dimethylhydroxylamine hydrochloride (1.0 equiv.) were added. After stirring for another 6 h at room temperature, the reaction mixture was diluted with 1N HCl and extracted with DCM. The organic layer was washed with saturated aqueous $NaHCO_3$ solution and dried over anhydrous Na_2SO_4. The crude product was purified by silica gel column chromatography (EtOAc/petroleum ether) to obtain the desired product *N*-methoxy-*N*-methyl-5-oxohexanamide in 86% yield.

S2: In an oven dried round bottom flask, methyltriphenylphosphonium bromide (3.57 g, 10 mmol) and anhydrous THF (20 mL) were combined under N_2 atmosphere. The resulting suspension was cooled to 0 °C, and KO^{t}Bu (1.23 g, 11 mmol) was added. The resulting yellow suspension was stirred at 0 °C for 45 mins. To this suspension, a solution of *N*-methoxy-*N*-methyl-5-oxohexanamide (0.87 g, 5 mmol) in anhydrous THF (5 mL) was added dropwise. The resulting mixture was allowed to stir at room temperature overnight. The reaction mixture was quenched with water (20 mL), and

extracted with EtOAc (3 × 20 mL). The combined organic layer was dried over anhydrous Na_2SO_4, and purified by silica gel column chromatography (EtOAc/petroleum ether) to obtain the desired product *N*-methoxy-*N*,5-dimethylhex-5-enamide (**1a**).

3.2 Synthesis of ethyl 5-methylhex-5-enoate (2a)

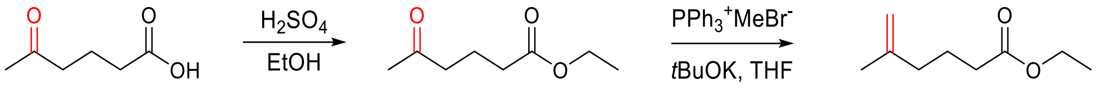

In an oven dried 100 mL round bottom flask, 4-acetylbutyric acid (2.60 g, 20 mmol) was dissolved in ethanol (30 mL). Then, a catalytic amount of sulfuric acid (0.1 equiv.) was added to the solution, and the mixture was stirred and heated at reflux. After 16 h, the reaction solvent was evaporated under reduced pressure to yield the crude product. The crude mixture is then washed with saturated aqueous NaCl solution (20 mL) and extracted by EtOAc (3 x 20 mL). The organic layer was dried over anhydrous $MgSO_4$ and concentrated under reduced pressure to yield the ethyl 5-oxohexanoate in quantitative yield, which was used without further purification.

Ethyl 5-methylhex-5-enoate (**2a**) was finally synthesized via methylenation of ethyl 5-oxohexanoate by utilizing the similar procedure with **S2**.

3.3 The synthesis of 3a – 12a, and 49[1]

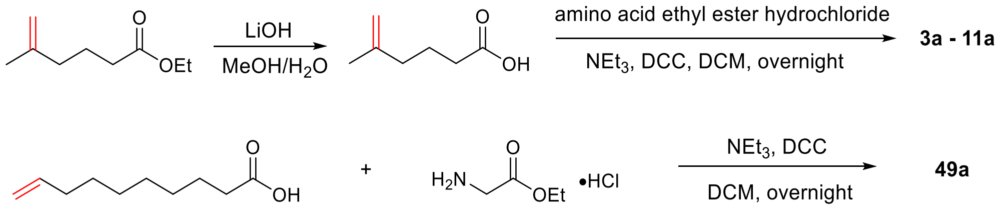

To a solution of ester **2a** (3.12 g, 20 mmol) in THF (30 mL) was added a solution of LiOH (5.00 equiv.) in H_2O/MeOH (1/1, 72 mL). The reaction mixture was heated to 60 °C for 1 h or until starting material was consumed by TLC analysis. The reaction mixture was then cooled to room temperature, and partitioned between Et_2O (20 mL) and 1 M aq. NaOH (20 mL). The aqueous layer was extracted with Et_2O (3 x 20 mL),

and the combined organic layers were washed with 1 M aq. NaOH (40 mL). Then, the combined aqueous phase was acidified to pH = 1 with 1 M aq. HCl and extracted with Et_2O (3 x 50 mL). Combined organic layers were washed with brine (50 mL), dried over anhydrous $MgSO_4$, and concentrated under reduced pressure to yield 5-methylhex-5-enoic acid. The crude products were carried on directly without further purification.

An amino acid ethyl ester hydrochloride (1.1 equiv.), which is commercially available, and DCM (5 mL/mmol) were combined in an oven dried Schlenk tube. Then, the solution was cooled to 0 ^oC, and triethylamine (1.2 equiv.) was injected via a syringe. After stirring for 10 mins, DCC (1.2 equiv.) and 5-methylhex-5-enoic acid or dec-9-enoic acid (1 equiv.) were added, and the resulting mixture was allowed to stir at room temperature overnight. After the completion of the reaction, the organic solution was collected after filtration and washing with DCM. Pure product was obtained by silica gel column chromatography (EtOAc/petroleum ether).
Note: **12a** was the byproduct during the synthesis of **3a** – **11a**.

3.4 The synthesis of 13a

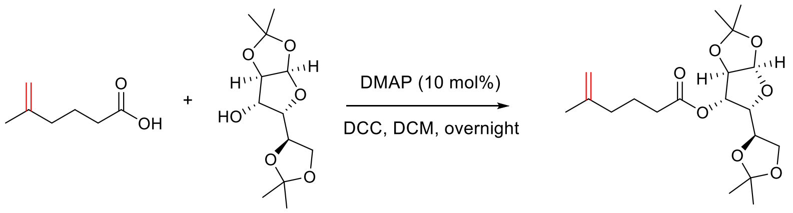

A suspension of diacetone-*D*-glucose (520 mg, 2 mmol), 5-methylhex-5-enoic acid (282 mg, 2.2 mmol), DCC (413 mg, 2 mmol) and DMAP (24 mg, 0.2 mmol) in dichloromethane (10 ml) was stirred at room temperature for 24 h. The mixture was filtered, and the filtrate was washed with water (3×30 mL), 5% aqueous HOAc (3×30 mL) and water (3×30 mL), dried over anhydrous Na_2SO_4 and concentrated under reduced pressure. Finally, the pure product **13a** was obtained by flash chromatrography on silica gel.

3.5 The synthesis of 5-methyl-1-phenylhex-5-en-1-one (16a) and 6-methyl-1-phenylhept-6-en-2-one (17a)

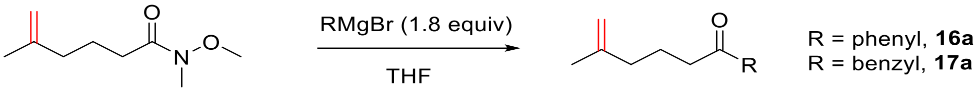

To an oven dried round bottom flask, a solution of **1a** (0.86 g, 5 mmol) in dry THF (10 mL) was added. The resulting solution was cooled to 0 °C, and fresh RMgBr (1.8 equiv.) was added dropwise. After stirring for 2 h at 0 °C, the reaction was quenched by adding saturated aqueous NH_4Cl solution (10 mL). Organic phase was separated, and the aqueous layer was extracted with EtOAc (3 × 10 mL). The combined organic phase was washed with saturated aqueous NaCl solution (2 × 20 mL), dried over anhydrous Na_2SO_4 and concentrated under reduced pressure. The crude product was purified by silica gel column chromatography (EtOAc/petroleum ether) to obtain the desired product.

3.6 The synthesis of 15a, 19a, 20a

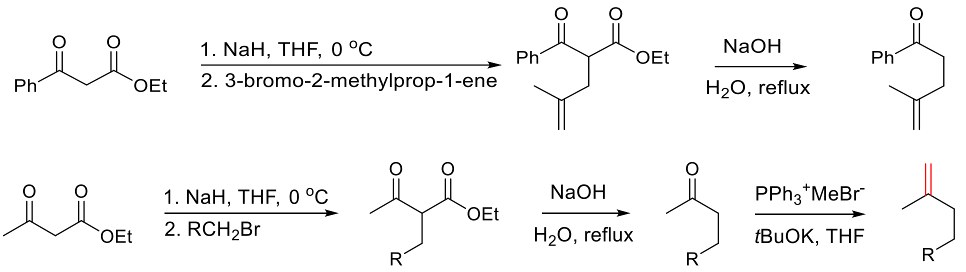

To a mixture of NaH (10 mmol, 60% dispersion in mineral oil) in dry THF (40 mL) in ice water bath was added ethyl 3-oxobutanoate or ethyl 3-oxo-3-phenylpropanoate (10 mmol) dropwise, and the resulting mixture was stirred for about 20 mins. In the same temperature, the corresponding bromide (10 mmol) was added dropwise, and then the reaction was warmed to room temperature and stirred for 6-12 hours. The reaction was quenched with sat. NH_4Cl solution (10 mL) and water (10 mL) sequentially. The subsequent mixture was extracted with EtOAc (20 mL x 3). The organic phase was washed with brine, dried over Na_2SO_4, and concentrated under reduced pressure to obtain the crude 1,3-dicarbonyl compounds.

The crude 1,3-dicarbonyl compound was allowed to stir under reflux temperature after the addition of 20 mL of 5% NaOH aq. The reaction mixtures was cooled after 2 hours, and was acidified to pH = 1 with 1 M aq. HCl and extracted with ethyl acetate (2 x 20 mL). The combined organic phase was dried over anhydrous Na_2SO_4 and concentrated

under reduced pressure. The pure ketone products were obtained by silica gel column chromatography (EtOAc/petroleum ether).

19a and **20a** were synthesized via a further methylenation of the corresponding ketones above by utilizing the similar procedure with **S2**.

3.7 The synthesis of 18a, 21a – 25a, 63 and 64

18a and **21a** – **25a** were synthesized via methylenation of the corresponding ketones by utilizing the similar procedure with **S2**. **63** and **64** were synthesized via methylenation of 4-methyl-1-phenylpent-4-en-1-one (**15a**) and 5-methyl-1-phenylhex-5-en-1-one (**16a**).

3.8 The synthesis of pentadec-2-en-2-ylbenzene (54)

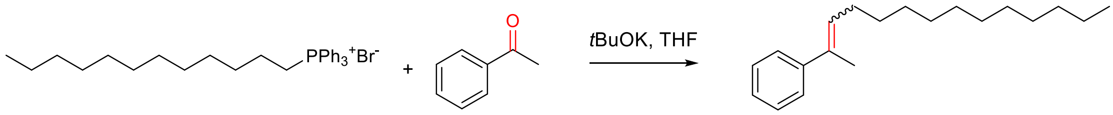

In an oven dried round bottom flask, dodecyltriphenylphosphonium bromide (5.11 g, 10 mmol) and anhydrous THF (20 mL) were combined under N_2 atmosphere. The resulting suspension was cooled to 0 °C, and KO^{t}Bu (1.23 g, 11 mol) was added. The resulting yellow suspension was stirred at 0 °C for 45 mins. To this suspension, a solution of acetophenone (0.60 g, 5 mmol) in anhydrous THF (5 mL) was added dropwise. The resulting mixture was allowed to stir at room temperature overnight, quenched by water (20 mL) and extracted with EtOAc (3 × 20 mL). The combined organic layer was dried over anhydrous Na_2SO_4 and purified by silica gel column chromatography (EtOAc/petroleum ether) to obtain the desired product **54**.

3.9 The synthesis of 1-(tert-butyl)-4-(propan-2-ylidene)cyclohexane (59)

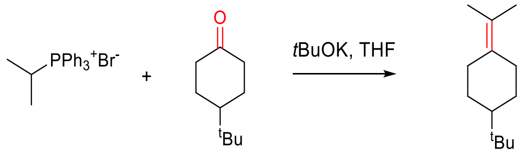

In an oven dried round bottom flask, isopropyltriphenylphosphonium bromide (3.85 g, 10 mmol) and anhydrous THF (20 mL) were combined under N_2 atmosphere. The resulting suspension was cooled to 0 °C and KO^{t}Bu (1.23 g, 11 mmol) was added. The

resulting yellow suspension was stirred at 0 °C for 45 mins. To this suspension, a solution of 4-(*tert*-butyl)cyclohexan-1-one (0.77 g, 5 mmol) in anhydrous THF (5 mL) was added dropwise. The resulting mixture was allowed to stir at room temperature overnight, quenched by water (20 mL) and extracted with EtOAc (3 × 20 mL). The combined organic layer was dried over anhydrous Na_2SO_4 and purified by silica gel column chromatography (EtOAc/petroleum ether) to obtain the desired product **59**.

3.10 The synthesis of 71

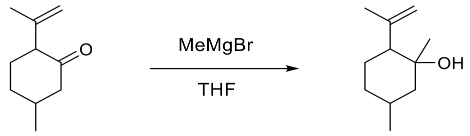

In an oven dried round bottom flask, 5-methyl-2-(prop-1-en-2-yl)cyclohexan-1-one (0.76 g, 5 mmol) and THF (10 mL) were combined under N_2 atmosphere. The solution was cooled to 0 °C, then MeMgBr (3 M, 3 mL) was added dropwise. The resulting mixture was stirred at room temperature until starting material was consumed by TLC analysis, and quenched by adding saturated aqueous NH_4Cl solution (10 mL). Organic phase was separated and the aqueous layer was extracted with EtOAc (3 × 10 mL/mmol). The combined organic phase was washed with saturated aqueous NaCl solution (2 × 20 mL), dried over anhydrous Na_2SO_4 and concentrated under reduced pressure. The crude product was purified by silica gel column chromatography (EtOAc/petroleum ether) to obtain the desired product.

2-Methylallyl benzoate (**14a**),[2] 1-Fluoro-4-(prop-1-en-2-yl)benzene (**27a**),[3] 1-bromo-4-(prop-1-en-2-yl)benzene (**29a**),[3] 1-nitro-4-(prop-1-en-2-yl)benzene (**30a**),[3] ethyl 4-(prop-1-en-2-yl)benzoate (**31a**),[4] 4-(prop-1-en-2-yl)benzoic acid (**32a**),[4] 1-isobutyl-4-(prop-1-en-2-yl)benzene (**33a**),[5] 2,4-dichloro-1-(prop-1-en-2-yl)benzene (**35a**),[6] but-1-en-2-ylbenzene (**36a**),[7] (3-bromoprop-1-en-2-yl)benzene (**37a**),[8] 1,3,5-trimethyl-2-(1-phenylvinyl)benzene (**39a**),[9] 4-(prop-1-en-2-yl)pyridine (**40a**),[7] 4,4,5,5-tetramethyl-2-(4-(prop-1-en-2-yl)phenyl)-1,3,2-dioxaborolane (**41a**),[10] (1-cyclopropylvinyl)benzene (**42a**),[7] 4-methyleneisochromane (**45a**)[11], 1-methylene-2,3-dihydro-1*H*-indene (**46a**),[12] 6-fluoro-4-methylenechromane (**47a**),[13] (3-methylbut-3-en-1-yn-1-yl)benzene (**48a**),[14]

ethene-1,1,2-triyltribenzene (**58**),[15] 1-bromo-4-(3-methylbut-2-en-2-yl)benzene (**61**),[16] (2-methylcyclopent-1-en-1-yl)benzene (**62**),[17] and 1,4-di(prop-1-en-2-yl)benzene (**65**),[18] (1R,4aR,7R,8aR)-8a-hydroxy-1,4a-dimethyl-7-(prop-1-en-2-yl)octahydronaphthalen-2(1H)-one (**75**),[19] 20-methylpregna-4,20-diene-3,6-dione (**77**)[20] were synthesized according to the literature.

4. General procedure for the aerobic oxidative cleavage of alkenes

4.1 The synthesis of $Mn(dtbpy)_2(OTf)_2$

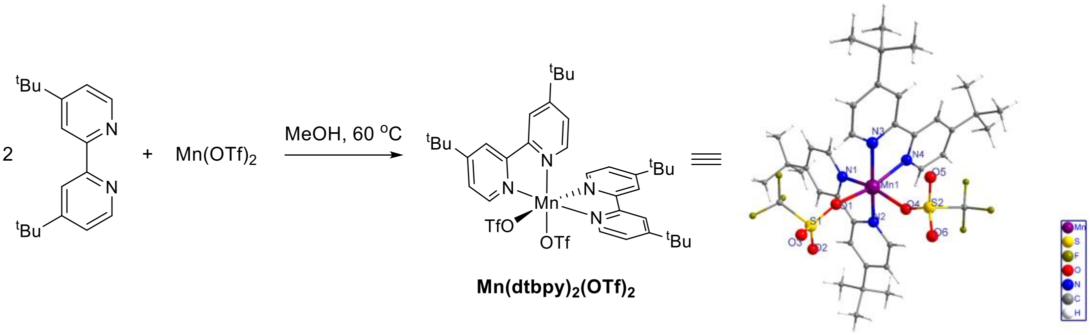

Scheme S1. Structure of $[Mn(dtbpy)_2(OTf)_2]$ determined by single-crystal X-ray diffraction. Selected bond distances: Mn1-N1, 2.246(2) Å; Mn1-N3, 2.238(2) Å; Mn1-O1, 2.146(2) Å; Mn1-O4, 2.152(2) Å. Selected bond angles: N1-Mn1-N2, 71.77(7)°; N1-Mn1-N4, 92.97(7)°; N1-Mn1-N3, 99.73(7)°; N1-Mn1-O1, 90.65(7)°; N1-Mn1-O4, 163.81(7)°. See Section 8.1 for more details.

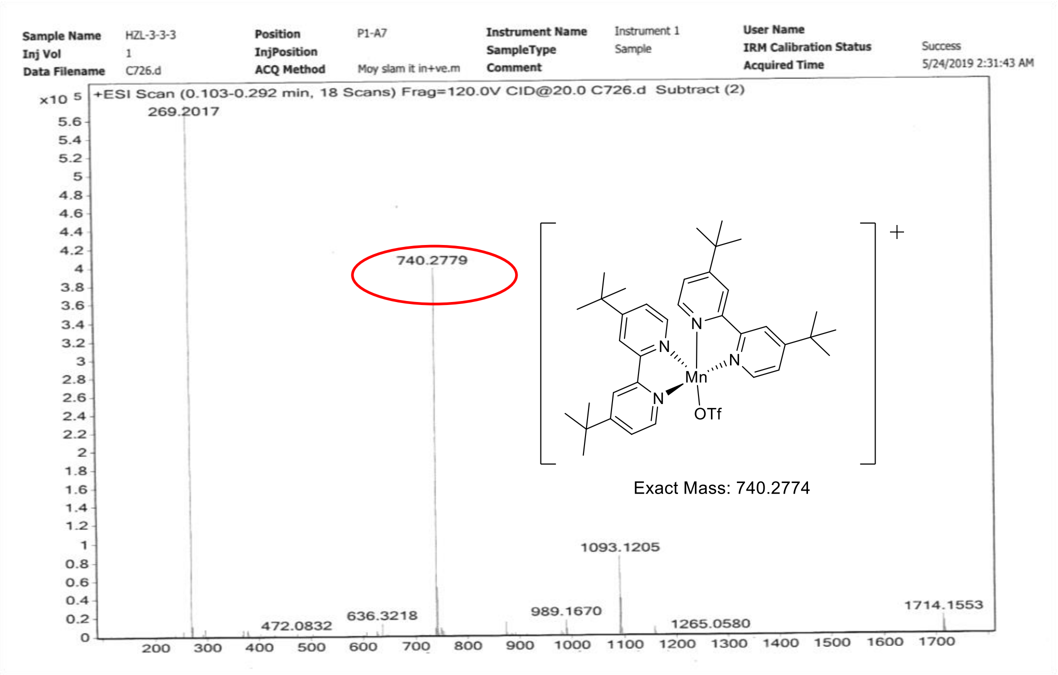

Fig. S2 HRMS data of complex $[Mn(dtbpy)_2(OTf)_2]$.

In an oven dried Schlenk tube, $Mn(OTf)_2$ (0.71 g, 2 mmol) and 4,4'-di-tert-butyl-2,2'-bipyridine (1.07 g, 4 mmol) were combined. Then the reaction tube was vacuumed and purged with nitrogen three times. 4 mL of methanol was added via syringe under nitrogen. The Schlenk tube was allowed to stir at 60 °C overnight in dark. After the completion of the reaction, methanol was removed by evaporation under reduced pressure to yield the crude product. The pure $Mn(dtbpy)_2(OTf)_2$ was obtained as single crystals by slow crystallization in DCM/hexane. The Structure of $Mn(dtbpy)_2(OTf)_2$ was determined by single-crystal X-ray diffraction (Scheme S1) and HRMS (Fig. S2).

4.2 General procedure for the aerobic oxidative cleavage of 1a under various conditions

To an oven dried Schlenk tube, a Mn complex (2 mol%), $Mn(OTf)_2$, $Cu(OTf)_2$, $Fe(OTf)_2$ or $CoCl_2$ (2 mol%) and ligand (**L1**, 2 mol%; or **L2**-**L10**, 4 mol%) were added. Then the reaction tube was vacuumed and purged with nitrogen three times. 1 mL of solvent was injected through a syringe under N_2. Then, the reaction mixture was heated at 60 °C for 1 h to *in-situ* prepare the Mn(II) catalyst. The reaction tube was cooled to room temperature and then connected to an oxygen balloon. Finally, after the addition of **1a** (0.25 mmol), the reaction tube was allowed to stir at 20 °C under blue light for 12 h. Then, water (2 mL) was added, and the solution was exctracted by ethyl acetate (3 mL x 3). The combined organic phase was dried over anhydrous Na_2SO_4 and concentrated under reduced pressure to produce the crude product. The product yield of **1** was obtained by ^1H NMR analysis with mesitylene as internal standard. The results were shown in Table S1.

Table S1. Optimisation of reaction conditions

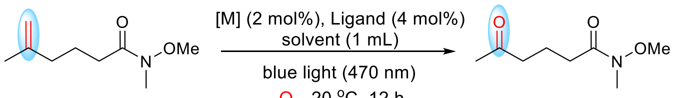

1a, 0.25 mmol → **1**

Entry	[M]	Ligand	solvent	yield of **1**[a]
1	[Mn(BPP)(OTf)$_2$]	-	MeOH	0%
2	Mn(OTf)$_2$	**L1**	MeOH	0%
3	[Mn(dabpy)$_2$(OTf)$_2$]	-	MeOH	21%
4	Mn(OTf)$_2$	**L2**	MeOH	0%
5	Mn(OTf)$_2$	**L3**	MeOH	27%
6	Mn(OTf)$_2$	**L4**	MeOH	32%
7	Mn(OTf)$_2$	**L5**	MeOH	20%
8	Mn(OTf)$_2$	**L6**	MeOH	14%
9	Mn(OTf)$_2$	**L7**	MeOH	5%
10	Mn(OTf)$_2$	**L8**	MeOH	25%
11	Mn(OTf)$_2$	**L9**	MeOH	24%
12	Mn(OTf)$_2$	**L10**	MeOH	0%
13	Cu(OTf)$_2$	**L4**	MeOH	0%
14	Fe(OTf)$_2$	**L4**	MeOH	0%
15	CoCl$_2$	**L4**	MeOH	0%
16	Mn(OTf)$_2$	**L4**	DCE	0%
17	Mn(OTf)$_2$	**L4**	THF	0%
18	Mn(OTf)$_2$	**L4**	benzene	0%
19	Mn(OTf)$_2$	**L4**	EtOH	0%
20	Mn(OTf)$_2$	**L4**	*i*PrOH	0%
21	Mn(OTf)$_2$	**L4**	CH_3CN	0%
22	Mn(OTf)$_2$	**L4**	MeOH/TFE (1:1)	62%
23	[Mn(dtbpy)$_2$(OTf)$_2$]	-	MeOH/TFE (1:1)	65%(63%)
24	Mn(OTf)$_2$	-	MeOH/TFE (1:1)	0%
25[b]	[Mn(dtbpy)$_2$(OTf)$_2$]	-	MeOH/TFE (1:1)	0%
26[c]	[Mn(dtbpy)$_2$(OTf)$_2$]	-	MeOH/TFE (1:1)	0%

[a] Yield determined by ^{1}H NMR with mesitylene as internel standard, isolated yield in parentheses; [b] Without blue light, 20 °C; [c] Without blue light, 70 °C.

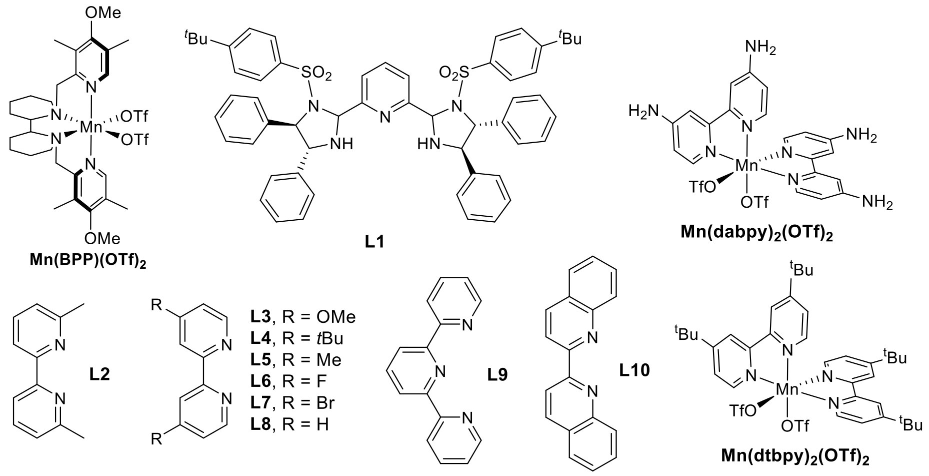

4.3 General procedure for the aerobic oxidative cleavage of 1a in the presence of peroxides or AIBN

The aerobic oxidation of **1a** involving radical inotators was investigated, and the results are shown in Table S2.

To an oven dried Schlenk tube, [Mn(dtbpy)$_2$(OTf)$_2$] (4.4 mg, 2 mol%) was added. Then

the reaction tube was vacuumed and purged with oxygen via an oxygen balloon. Methanol (0.5 mL) and 2,2,2-trifluoroethanol (0.5 mL) was injected through a syringe. Finally, after the addition of **1a** (42.8 mg, 0.25 mmol) and peroxides or AIBN (0.375 mmol), the reaction tube was allowed to stir at 20 °C or 90 °C in dark for 12 h. Then, water (2 mL) was added, and the solution was exctracted by ethyl acetate (3 mL x 3). The combined organic phase was dried over anhydrous Na_2SO_4 and concentrated under reduced pressure to produce the crude product. The product yield of **1** was obtained by ^{1}H NMR analysis with mesitylene as internal standard.

Table S2. Oxidation of 1a in the presence of peroxide.

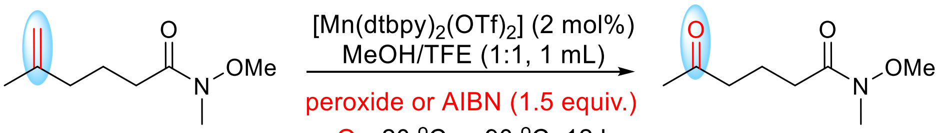

1a, 0.25 mmol   **1**

Entry	Additive	Temp.	yield of **1**[a]
1	H_2O_2 (30% in water)	20 °C	0%
2	TBHP	20 °C	0%
3	DTBP	20 °C	0%
4	Benzoyl peroxide	20 °C	0%
5	$Na_2S_2O_8$	20 °C	0%
6	AIBN	20 °C	0%
7	-	90 °C	0%
8	H_2O_2 (30% in water)	90 °C	0%
9	TBHP	90 °C	0%
10	DTBP	90 °C	trace
11	Benzoyl peroxide	90 °C	0%
12	$Na_2S_2O_8$	90 °C	0%
13	AIBN	90 °C	0%
14[b]	AIBN	20 °C	62%
15	CH_2O (20 mol%)	20 °C	0%

[a] Yield determined by ^{1}H NMR with mesitylene as internel standard; [b] With blue light.

As shown in Table S2, all those well-known radical initiators could not promote the aerobic cleavage of the C=C double bond in **1a** in the absence of blue light, even at a high temperature.

4.4 General procedure for the aerobic oxidative cleavage of 1a in the presence of well-known photosensitizers

To an oven dried Schlenk tube, a photocatalyst (2 mol%) was added. Then the reaction tube was vacuumed and purged with oxygen via an oxygen balloon. Methanol (0.5 mL) and 2,2,2-trifluoroethanol (0.5 mL) was injected through a syringe. Finally, after the addition of **1a** (42.8 mg, 0.25 mmol), the reaction tube was allowed to stir at 20 °C under blue light for 12 h. Then, water (2 mL) was added, and the solution was exctracted by ethyl acetate (3 mL x 3). The combined organic phase was dried over anhydrous Na_2SO_4 and concentrated under reduced pressure to produce the crude product. The product yield of **1** was obtained by ^1H NMR analysis with mesitylene as internal standard.

Four well-known photosensitizers capable of producing singlet oxygen with blue light were used as replacement catalysts for the oxidative cleavage of **1a**. No desired oxidative cleavage product **1** was observed when eosin Y, $Ru(bpy)_3 \cdot 6H_2O$, $Ir(bpy)_3$ or rose bengal was employed (Table S3). The poor selectivity toward **1** with these $^1O_2$-generating photocatalysts indicates that the formation of **1** under the catalysis of $[Mn(dtbpy)_2(OTf)_2]$ and blue light irradiation involves no singlet oxygen as a key oxidizing intermediate.

Table S3. Oxidation of 1a in the presence of well-kown photosensitizers.

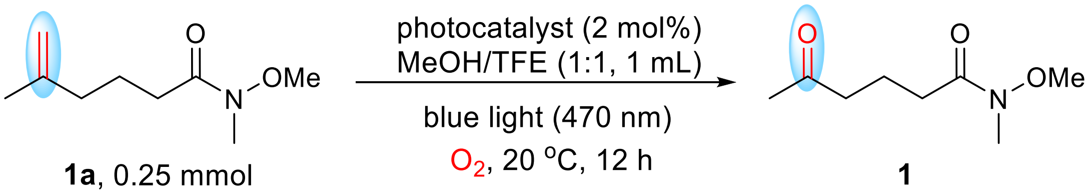

Entry	Photocatalyst	yield of **1**[a]
1	Eosin Y disodium salt	0%
2	$[Ru(bpy)_3 \cdot 6H_2O]$	0%
3	$[Ir(dFppy)_3]$	0%
4	Rose bengal	0%

[a] Yield determined by ^1H NMR with mesitylene as internel standard.

4.5 Standard procedure for aerobic oxidative cleavage of alkenes

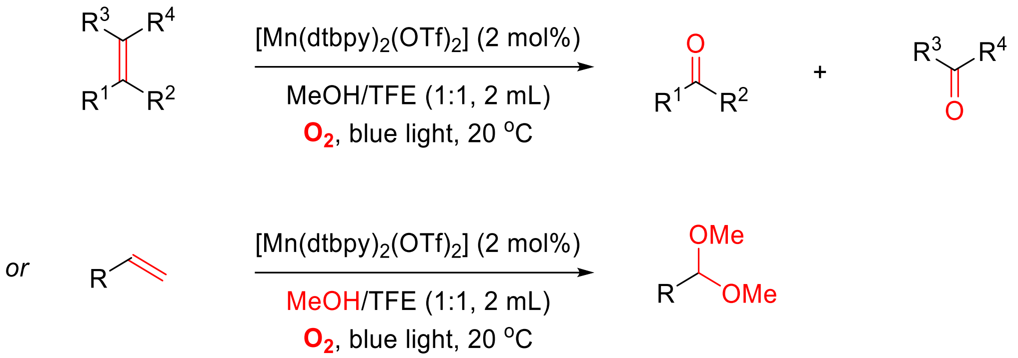

To an oven dried Schlenk tube, $[Mn(dtbpy)_2(OTf)_2]$ (8.9 mg, 2 mol%) was added. Then the reaction tube was vacuumed and purged with oxygen via an oxygen balloon. Methanol (1 mL) and 2,2,2-trifluoroethanol (1 mL) was injected through a syringe. Finally, after the addition of an alkene (0.5 mmol), the reaction tube was allowed to stir at 20 °C under blue light for 5 – 24 h until the alkene was consumed by TLC analysis. After the crude mixture was concentrated under vacuum, the pure product was obtained by flash chromatography on silica gel with hexane/ethyl acetate, unless otherwise noted.

*Oxidative cleavage of alkene **56**:* To an oven dried Schlenk tube, $[Mn(dtbpy)_2(OTf)_2]$ (8.9 mg, 2 mol%) was added. Then the reaction tube was vacuumed and purged with oxygen via an oxygen balloon. Methanol (1 mL) and 2,2,2-trifluoroethanol (1 mL) was injected through a syringe. Finally, after the addition of alkene **56** (97.0 mg, 0.5 mmol), the reaction tube was allowed to stir at 20 °C under blue light for 12 h until the alkene was consumed by TLC analysis. Then, water (2 mL) was added, and the solution was exctracted by ethyl acetate (3 mL x 3). The combined organic phase was dried over anhydrous Na_2SO_4 and concentrated under reduced pressure to produce the crude product. The product yields of **26** and **57** were obtained by ^{1}H NMR analysis with mesitylene (17.3 mg) as internal standard (see the spectrum in Fig. S3).

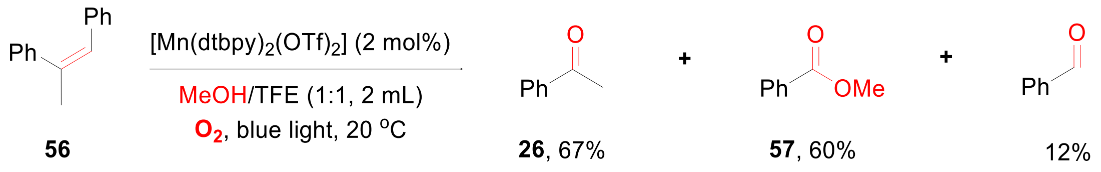

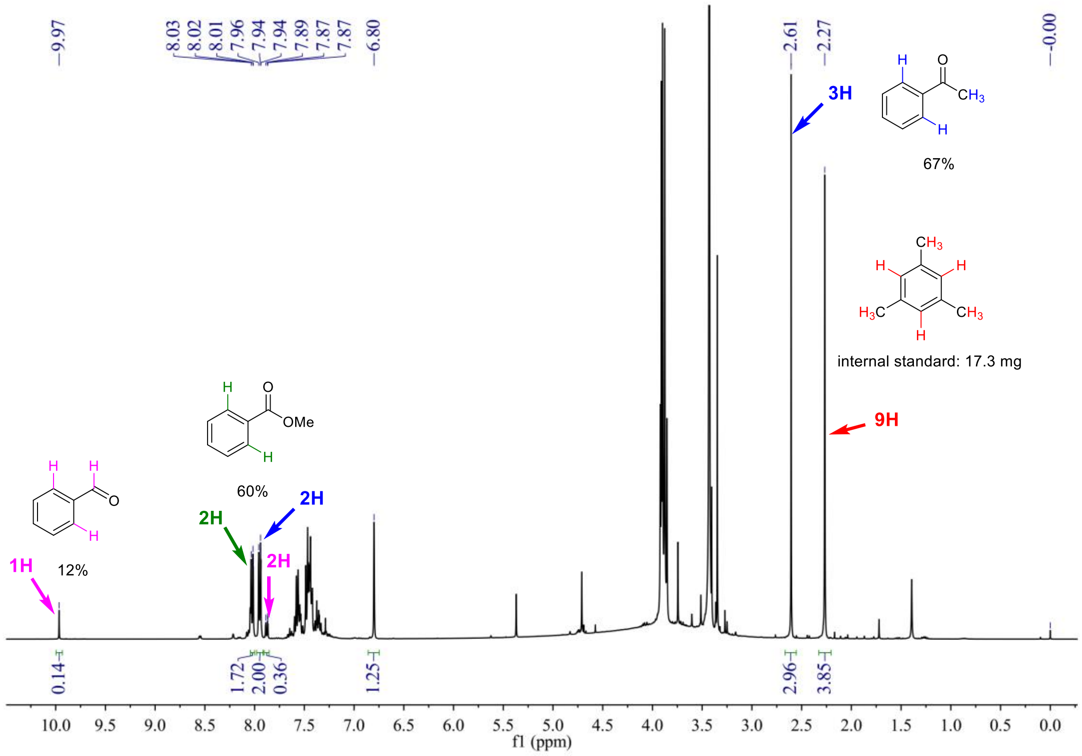

Fig. S3 Crude ^{1}H NMR spectrum of the reaction mixture of **56** oxidation including internal standard.

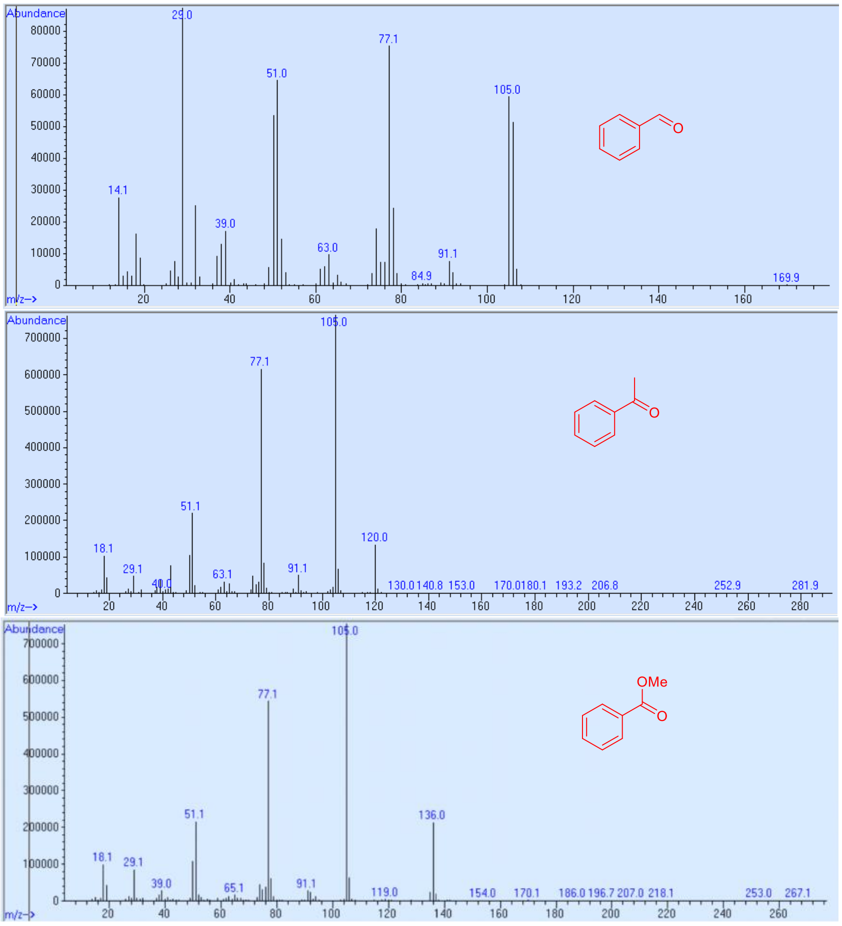

Fig. S4 GC-MS spectra of the prodcuts of **56** oxidation.

__Oxidative cleavage of alkene 58:__ To an oven dried Schlenk tube, [Mn(dtbpy)$_2$(OTf)$_2$] (8.9 mg, 2 mol%) was added. Then the reaction tube was vacuumed and purged with oxygen via an oxygen balloon. Methanol (1 mL) and 2,2,2-trifluoroethanol (1 mL) was injected through a syringe. Finally, after the addition of alkene **58** (128 mg, 0.5 mmol), the reaction tube was allowed to stir at 20 °C under blue light for 12 h until the alkene was consumed by TLC analysis. Then, water (2 mL) was added, and the solution was exctracted by ethyl acetate (3 mL x 3). The combined organic phase was dried over anhydrous Na_2SO_4 and concentrated under reduced pressure to produce the crude product. The product yields of **38** and **57** were obtained by ^{1}H NMR analysis with mesitylene (34.6 mg) as internal standard (see spectrum in Fig. S5).

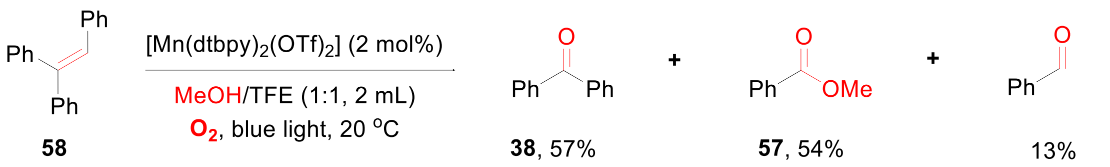

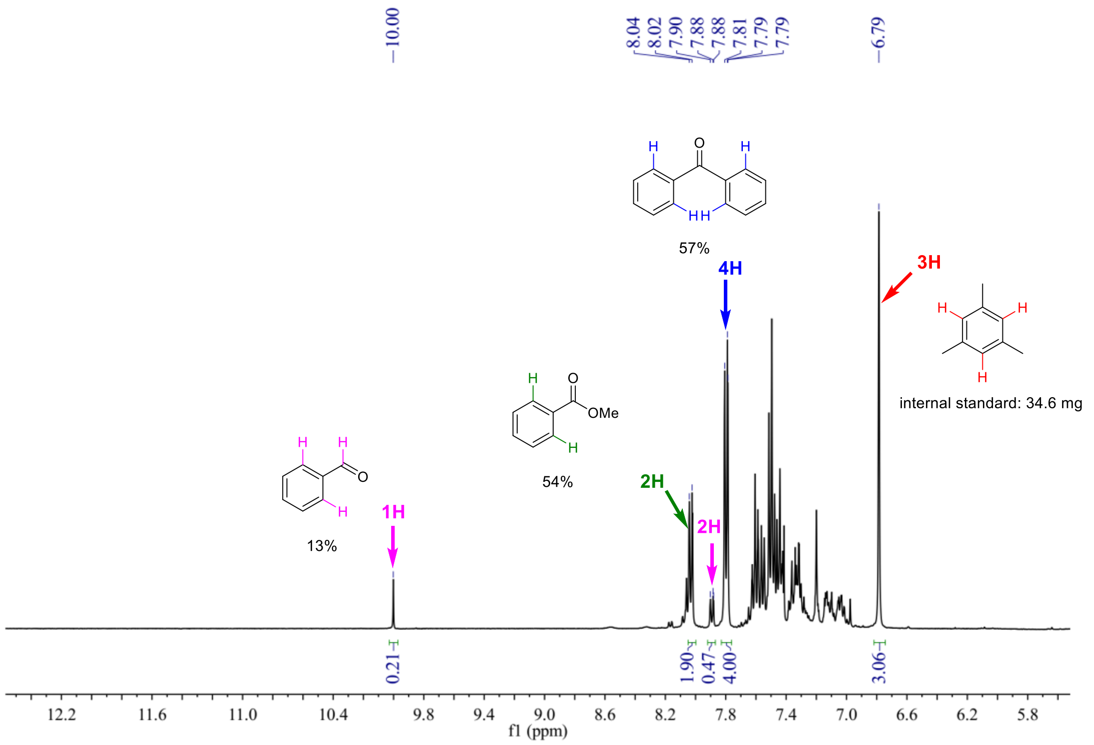

Fig. S5 Crude ^{1}H NMR spectrum of the reaction mixture of **58** oxidation including internal standard.

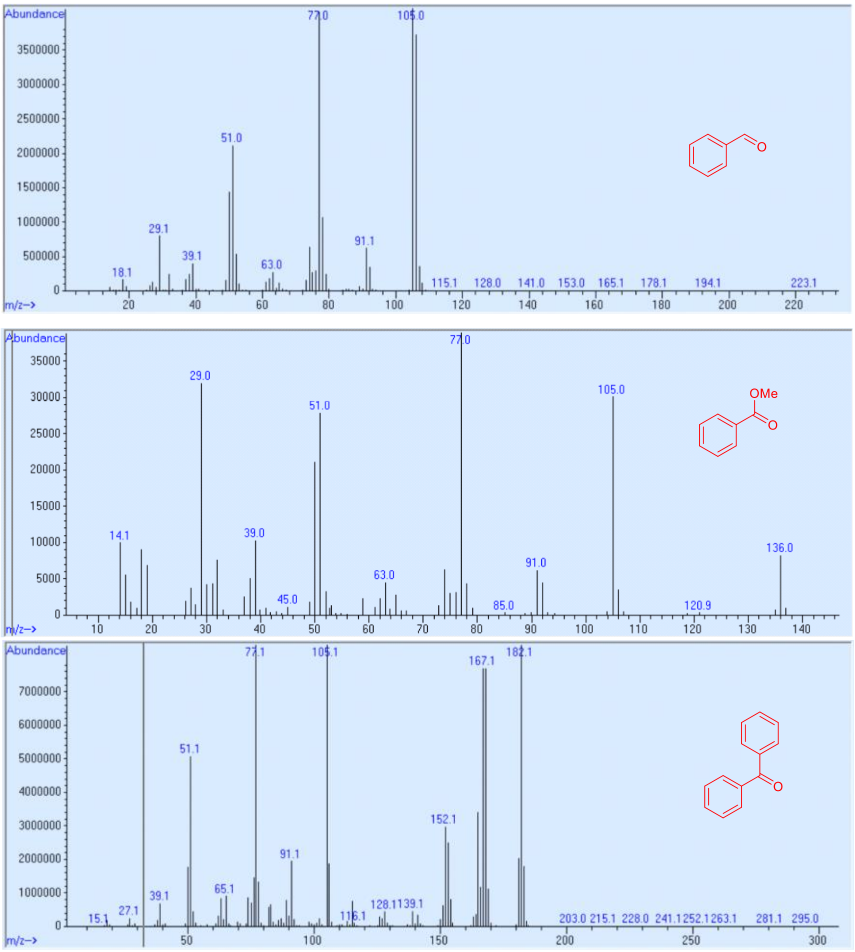

Fig. S6 GC-MS spectra of the prodcuts of **58** oxidation.

4.6 Procedure for gram-scale aerobic oxidative cleavage of α-methylstyrene

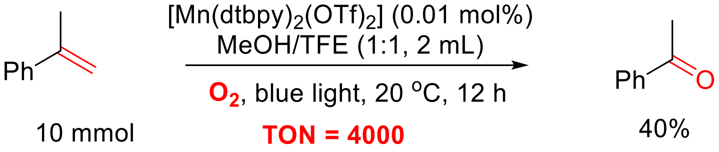

Scheme S2. Gram-scale aerobic cleavage of *α*-methylstyrene

To an oven dried Schlenk tube, [Mn(dtbpy)$_2$(OTf)$_2$] (0.9 mg, 0.01 mol%) was added. Then the reaction tube was vacuumed and purged with oxygen via an oxygen balloon. Methanol (1 mL) and 2,2,2-trifluoroethanol (1 mL) was injected through a syringe. Finally, after the addition of *α*-methylstyrene (1.18 g, 10.0 mmol), the reaction tube was allowed to stir at 20 °C under blue light for 12 h. Then, water (2 mL) was added, and the solution was exctracted by DCM (5 mL x 3). The combined organic phase was dried over anhydrous Na_2SO_4 and concentrated under reduced pressure to produce the crude product. Finally, the product yield of acetophenone (40%) was obtained by ^{1}H NMR

analysis with mesitylene as internal standard.

5. Mechanistic investigations

5.1 Singlet oxygen trap experiment

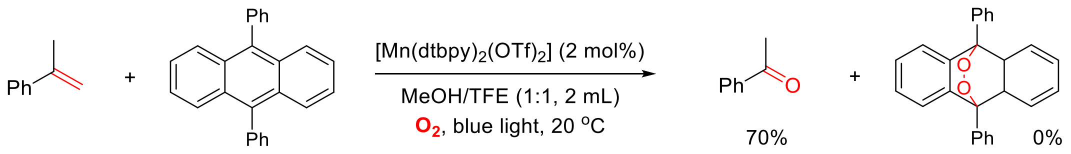

Scheme S3. Oxidative cleavage of *α*-methylstyrene in the presence of $^1O_2$ trap.

To an oven dried Schlenk tube, [Mn(dtbpy)$_2$(OTf)$_2$] (8.9 mg, 2 mol%) was added. Then the reaction tube was vacuumed and purged with oxygen via an oxygen balloon. Methanol (1 mL) and 2,2,2-trifluoroethanol (1 mL) was injected through a syringe. Finally, after the addition of *α*-methylstyrene (59 mg, 0.5 mmol) and 9,10-diphenylanthracene (33 mg, 0.1 mmol, singlet oxygen trap), the reaction tube was allowed to stir at 20 °C under blue light for 2 h. No 9,10-diphenyl-4a,9,9a,10-tetrahydro-9,10-epidioxyanthracene was observed by TLC/GC-MS/^{1}H NMR analysis. The product yield of acetophenone (70%) was obtained by ^{1}H NMR analysis with mesitylene as internal standard. These results indicate that singlet oxygen is not involved as the key intermediate during the alkene oxidation.

5.2 Chracterization of bis-μ-O_2-Mn_2 complex

5.2.1 Formation of the bis-μ-O_2-Mn_2 *complex via O_2 activation by Mn(dtbpy)$_2$(OTf)$_2$*

In an oven dried Schlenk tube, [Mn(dtbpy)$_2$(OTf)$_2$] (8.9 mg, 0.01 mmol) was added. Then the reaction tube was vacuumed and purged with O_2 via an oxygen balloon. 2 mL of methanol was added via syringe. The Schlenk tube was allowed to stir under blue light at 20 °C for 1 h. A clear color change from pale yellow to greenish brown was observed during the oxidation. Then, the solution was analysed with HRMS.

5.2.2 Formation of bis-μ-O_2-Mn_2 *complex via the oxidation of Mn(dtbpy)$_2$(OTf)$_2$ by PhIO*

To an oven dried Schlenk tube, [Mn(dtbpy)$_2$(OTf)$_2$] (8.9 mg, 0.01 mmol) was added.

Then the reaction tube was vacuumed and purged with N_2 three times. 2 mL of methanol and PhIO (6.6 mg, 0.03 mmol) were added under N_2. The Schlenk tube was allowed to stir at 20 °C for 20 mins in dark. A clear color change from pale yellow to greenish brown was observed immediately during the oxidation. Then, the solution was analysed with HRMS.

5.2.3 The isolation of bis-μ-O_2-Mn_2 *complex*

In an oven dried Schlenk tube, [Mn(dtbpy)$_2$(OTf)$_2$] (88.9 mg, 0.1 mmol) was added. Then the reaction tube was vacuumed and purged with N_2 three times. 2 mL of methanol and PhIO (66 mg, 0.3 mmol) were added under N_2. The Schlenk tube was allowed to stir at 20 °C for 20 mins in dark. A clear color change from pale yellow to greenish brown was observed during the oxidation. After the completion of the reaction, methanol was removed by evaporation under reduced pressure to yield the crude product, which was washed with diethyl ether (10 mL) three times. The pure **bis-μ-O_2-Mn_2** complex was obtained as single crystals (black color) by slow crystallization in MeOH (1 mL)/EtOAc (3 mL)/hexane (20 mL).

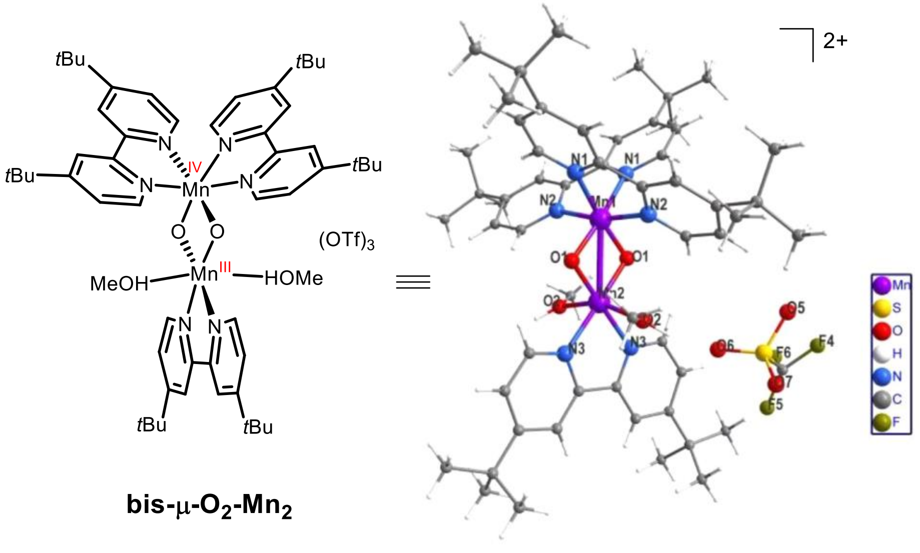

Fig. S7 Structure of **bis-μ-O_2-Mn_2** complex determined by single-crystal X-ray diffraction. Selected bond distances: Mn1-Mn2, 2.6650(10) Å; Mn1-N1, 2.069(3) Å; Mn1-N2, 2.011(3) Å; Mn1-N3, 2.041(3) Å; Mn1-O1, 1.786(2) Å; Mn2-O1, 1.840(2) Å; Mn2-O2, 2.257(3) Å. Selected bond angles: O1-Mn1-O1^1, 86.98(15)°; O1-Mn2-O^1, 83.79(15)°; Mn1-O1-Mn2, 94.61(11)°; N2-Mn1-N2^1, 167.61(17)°; O2-Mn2-O2^1, 161.60(16)°. See Section 8.2 for more details.

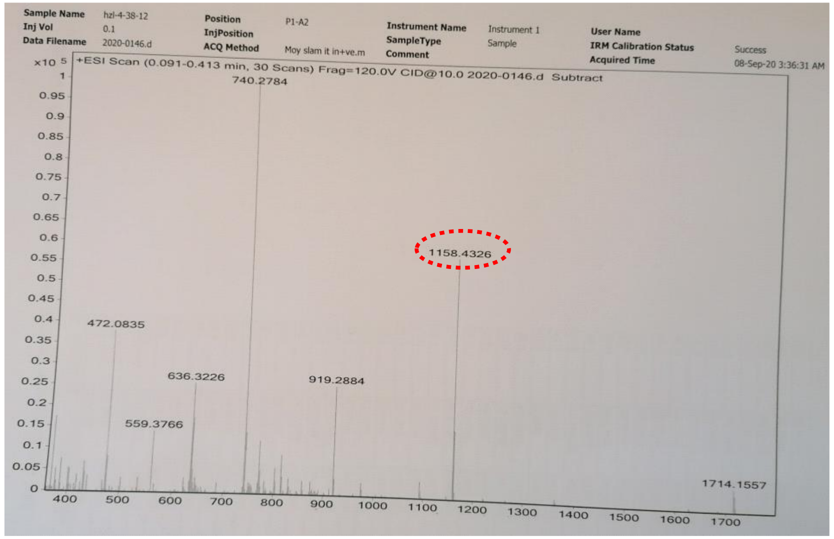

(A)

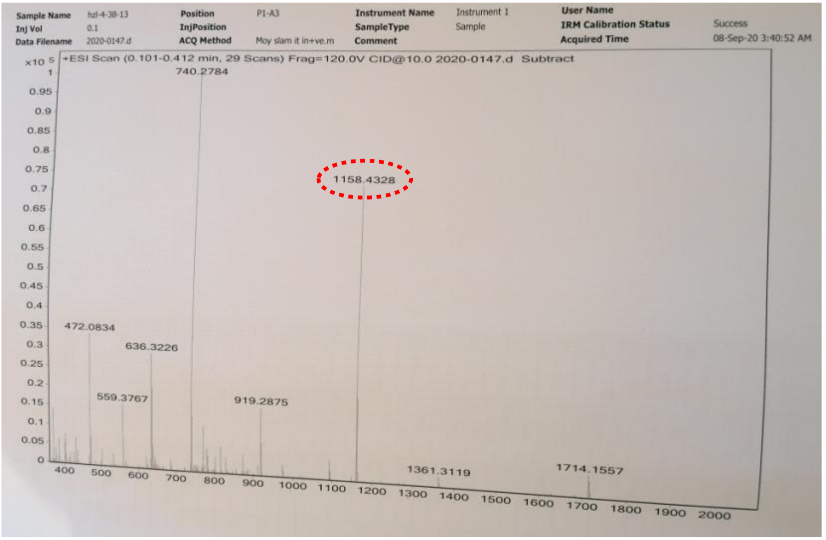

(B)

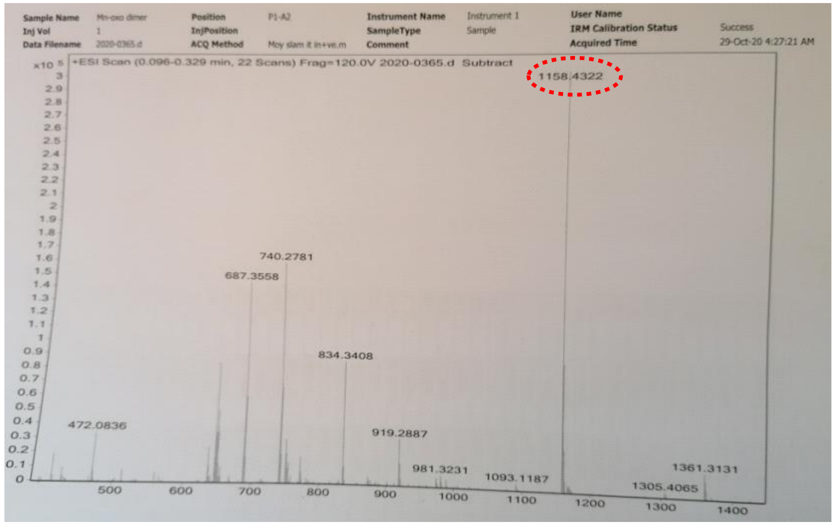

(C)

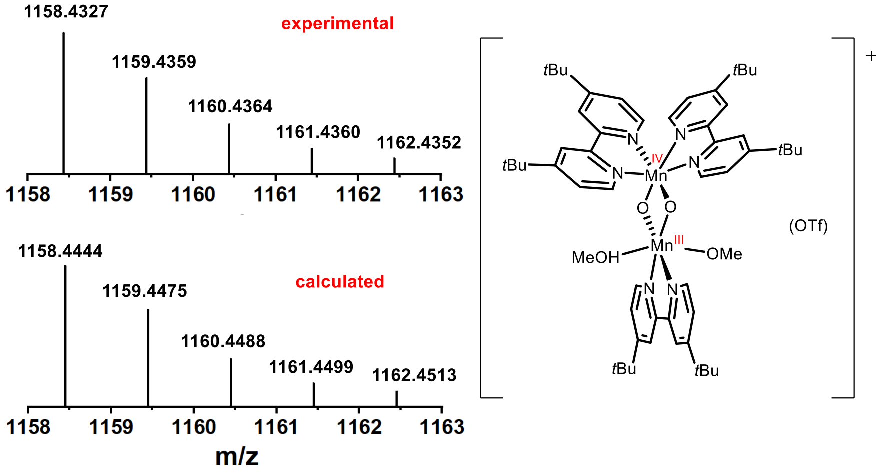

(D)

Fig. S8 HRMS spectra of **bis-μ-O₂-Mn₂** complex in MeOH (**A**. in-situ formed via O_2 activation, **B**. in-situ formed via PhIO oxidation, **C**. isolated crystals, **D**. experimental spectrum and calculated spetrum).

The formation of the same **bis-μ-O_2-Mn_2** complex by photo-induced O_2 activation and PhIO oxidation was demonstrated by HRMS analysis (Fig. S8). As shown in Fig. S8D, the identical m/z signal could be assigned to the fragment peak of the **bis-μ-O_2-Mn_2** complex by losing a HOTf and a $^{-}$OTf during ionization.

5.3 Kinetic investigation

5.3.1 Kinetics of oxidative cleavage of α-methylstyrene by Mn(dtbpy)$_2$(OTf)$_2$

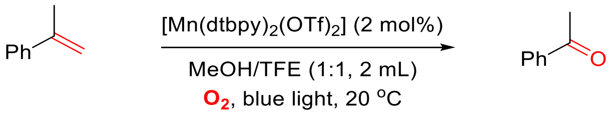

To an oven dried Schlenk tube, [Mn(dtbpy)$_2$(OTf)$_2$] (8.9 mg, 2 mol%) and biphenyl (24.6 mg) as internal standard were added. The reaction tube was vacuumed and purged with oxygen via an oxygen balloon. Methanol (1 mL) and 2,2,2-trifluoroethanol (1 mL) was injected through a syringe. Finally, after the addition of *α*-methylstyrene (59 mg, 0.5 mmol), the reaction tube was allowed to stir at 20 °C under blue light. Then, 0.1 mL of the reaction mixture was taken out for GC analysis every 10 mins.

5.3.2 Kinetic study of oxidative cleavage of α-methylstyrene by pre-activated Mn-catalyst via O_2 activation

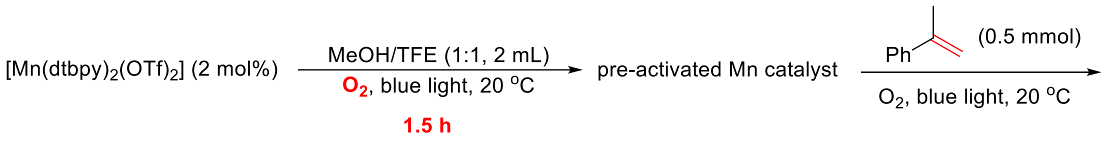

To an oven dried Schlenk tube, [Mn(dtbpy)$_2$(OTf)$_2$] (8.9 mg, 2 mol%) and biphenyl (24.6 mg) as internal standard were added. The reaction tube was vacuumed and purged with oxygen via an oxygen balloon. Methanol (1 mL) and 2,2,2-trifluoroethanol (1 mL) was injected through a syringe. Then the reaction mixture was stirred for 1.5 h at 20 °C under blue light. After that, *α*-methylstyrene (59 mg, 0.5 mmol) was added, and the reaction tube was allowed to stir at 20 °C under blue light again. 0.1 mL of the reaction mixture was taken out for GC analysis every 10 mins.

5.3.3 Kinetic study of oxidative cleavage of α-methylstyrene by pre-activated Mn-catalyst via PhIO oxidation

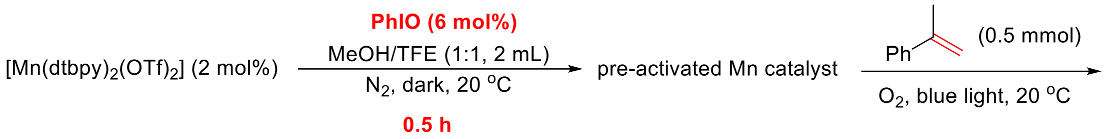

To an oven dried Schlenk tube, [Mn(dtbpy)$_2$(OTf)$_2$] (8.9 mg, 2 mol%) and biphenyl (24.6 mg) as internal standard were added. The reaction tube was vacuumed and purged with nitrogen three times. Methanol (1 mL) and 2,2,2-trifluoroethanol (1 mL) was injected through a syringe. Then PhIO (6.6 mg, 6 mol%) was added under nitrogen, and the reaction mixture was stirred for 0.5 h at 20 °C in dark. After that, an oxygen balloon was connected and *α*-methylstyrene (59 mg, 0.5 mmol) was added. Finally, the reaction tube was allowed to stir at 20 °C under blue light. 0.1 mL of the reaction mixture was taken out for GC analysis every 10 mins.

5.3.4 Kinetic study of oxidative cleavage of α-methylstyrene by bis-μ-O$_2$-Mn$_2$ complex

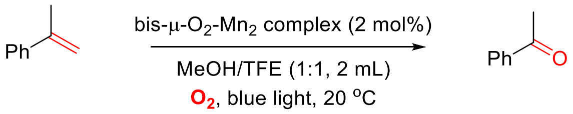

To an oven dried Schlenk tube, the **bis-μ-O$_2$-Mn$_2$** complex (14.6 mg, 2 mol%) and biphenyl (24.6 mg) as internal standard were added. The reaction tube was vacuumed and purged with oxygen via an oxygen balloon. Methanol (1 mL) and 2,2,2-trifluoroethanol (1 mL) was injected through a syringe. Finally, after the addition of *α*-methylstyrene (59 mg, 0.5 mmol), the reaction tube was allowed to stir at 20 °C under blue light. Then, 0.1 mL of the reaction mixture was taken out for GC analysis every 10 mins.

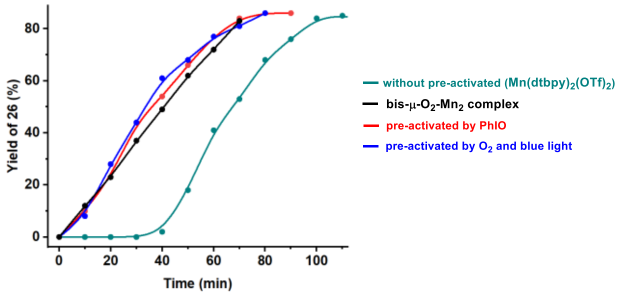

Fig. S9 A. Kinetic behavior of the aerobic oxidation of α-methylstyrene catalyzed by 2 mol% [Mn(dtbpy)$_2$(OTf)$_2$], **bis-μ-O$_2$-Mn$_2$** complex or pre-activated Mn-catalyst via PhIO oxidation or O$_2$-activation.

5.3.5 Kinetic isotope effect investigation

5.3.5.1 Kinetic study of oxidative cleavage of α-methylstyrene using CD_3OD and CF_3CH_2OH

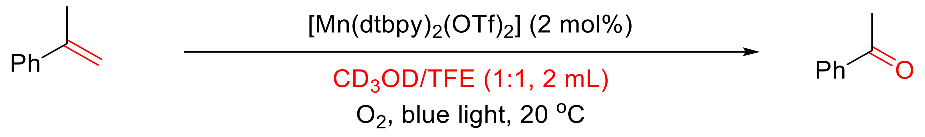

To an oven dried Schlenk tube, [Mn(dtbpy)$_2$(OTf)$_2$] (8.9 mg, 2 mol%) and biphenyl (24.6 mg) as internal standard were added. The reaction tube was vacuumed and purged with oxygen via an oxygen balloon. Methanol-d4 (1 mL) and 2,2,2-trifluoroethanol (1 mL) was injected through a syringe. Finally, after the addition of *α*-methylstyrene (59 mg, 0.5 mmol), the reaction tube was allowed to stir at 20 °C under blue light. Then, 0.1 mL of the reaction mixture was taken out for GC analysis every 10 mins.

5.3.5.2 Kinetic study of oxidative cleavage of α-methylstyrene using CH_3OD and CF_3CH_2OH

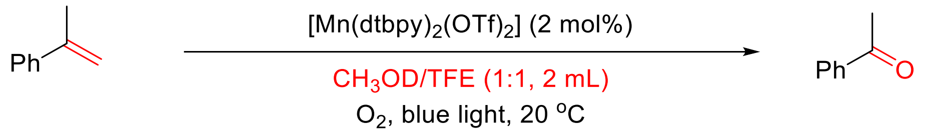

To an oven dried Schlenk tube, [Mn(dtbpy)$_2$(OTf)$_2$] (2 mol%, 8.9 mg) and biphenyl (24.6 mg) as internal standard were added. The reaction tube was vacuumed and purged with oxygen via an oxygen balloon. Methan(ol-d) (1 mL) and 2,2,2-trifluoroethanol (1 mL) was injected through a syringe. Finally, after the addition of α-methylstyrene (59 mg, 0.5 mmol), the reaction tube was allowed to stir at 20 °C under blue light. Then, 0.1 mL of the reaction mixture was taken out for GC analysis every 10 mins.

The results of kinetic isotope effect investigations are shown in Fig. S10. CH_3OH and CH_3OD gave the same introduction period and reaction rate; however, CD_3OD afforded an induction period shorter by ca 10 min but a similar rate of oxidizing *α*-methylstyrene. This somewhat surprising inverse kinetic isotope effect indicates that methanol is likely to be involved in the O_2 activation; but the cleavage of C-H and O-H bond of methanol

is not the rate-determining step.

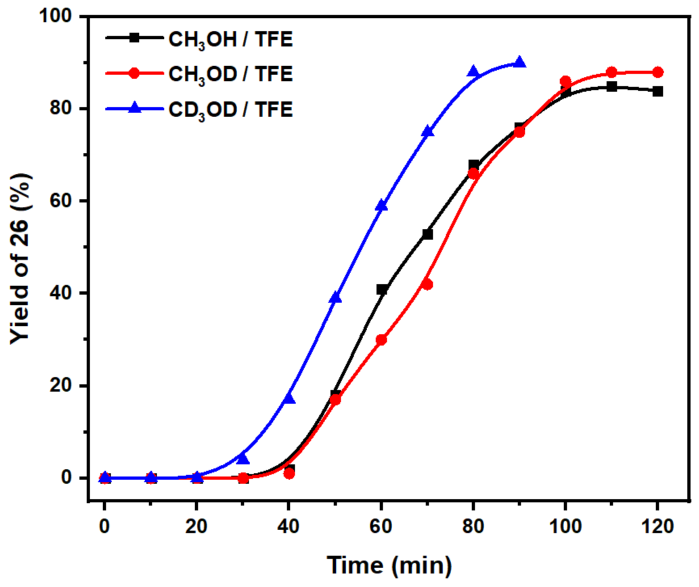

Fig. S10 Kinetic isotope effect investigations.

5.3.6 Kinetic behavior of the oxidative cleavage of α-methylstyrene with the addition of formaldehyde

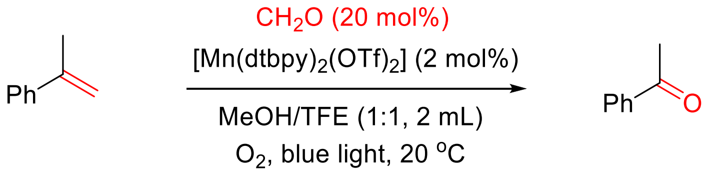

To an oven dried Schlenk tube, [Mn(dtbpy)$_2$(OTf)$_2$] (8.9 mg, 2 mol%) and biphenyl (23.1 mg) as internal standard were added. The reaction tube was vacuumed and purged with oxygen via an oxygen balloon. Methanol (1 mL) and 2,2,2-trifluoroethanol (1 mL) was injected through a syringe. Finally, after the addition of formaldehyde (0.1 mmol, 40 wt. % in water) and α-methylstyrene (59 mg, 0.5 mmol), the reaction tube was allowed to stir at 20 °C under blue light. Then, 0.1 mL of the reaction mixture was taken out for GC analysis every 10 mins.

Since formaldehyde used in the above experiment is a 40 wt. % water solution, the effect of water was also examined as a control reaction. The experimental procedure is as follows: To an oven dried Schlenk tube, [Mn(dtbpy)$_2$(OTf)$_2$] (8.9 mg, 2 mol%) and biphenyl (23.1 mg) as internal standard were added. The reaction tube was vacuumed and purged with oxygen via an oxygen balloon. Methanol (1 mL) and 2,2,2-

trifluoroethanol (1 mL) was injected through a syringe. Finally, after the addition of H_2O (4.5 mg) and α-methylstyrene (59 mg, 0.5 mmol), the reaction tube was allowed to stir at 20 °C under blue light. Then, 0.1 mL of the reaction mixture was taken out for GC analysis every 10 mins.

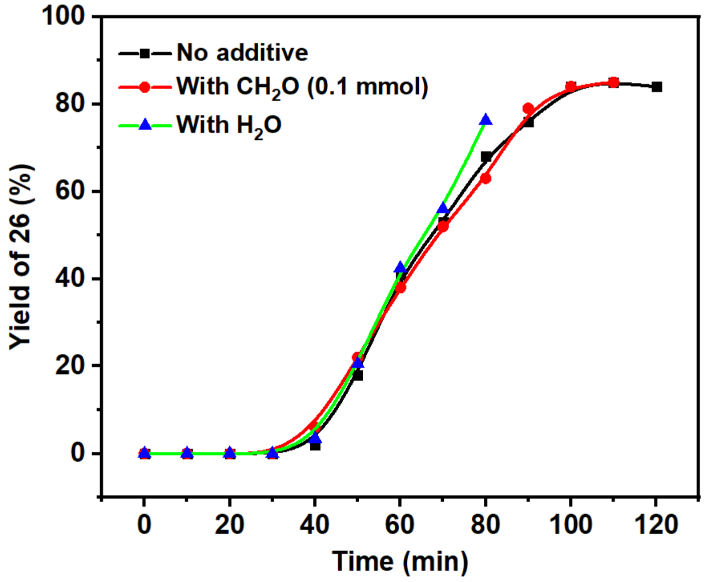

Fig. S11 Kinetic behavior of the oxidation of α-methylstyrene with or without formaldehyde.

The kinetic behaviors of the oxidation of α-methylstyrene with or without formaldehyde are shown in Fig. S11, indicating that the addition of formaldehyde does not affect the induction time and reaction rate.

5.3.7 Light on/off experiment

Oxidation with [Mn(dtbpy)$_2$(OTf)$_2$] under light on/off conditions

To an oven dried Schlenk tube, [Mn(dtbpy)$_2$(OTf)$_2$] (8.9 mg, 2 mol%) and biphenyl (24.6 mg) as internal standard were added. The reaction tube was vacuumed and purged with oxygen via an oxygen balloon. Methanol (1 mL) and 2,2,2-trifluoroethanol (1 mL) was injected through a syringe. Finally, after the addition of α-methylstyrene (59 mg, 0.5 mmol), the reaction tube was allowed to stir at 20 °C under blue light. After 60 mins, 0.1 mL of the reaction mixture was taken out for GC analysis, and in the meantime, the light was switched off and the reaction tube was further stirred in dark. After 30 mins, 0.1 mL of the reaction mixture was taken out for GC analysis, and the reaction tube was exposed to blue light again for another 30 mins. Finally, 0.1 mL of the reaction mixture

was taken out for GC analysis.

As shown in Fig. S12, comparing with the reaction under the standard conditions, the reaction in the absence of blue light showed an extremely slow reaction rate, which highlights the importance of light irradiation.

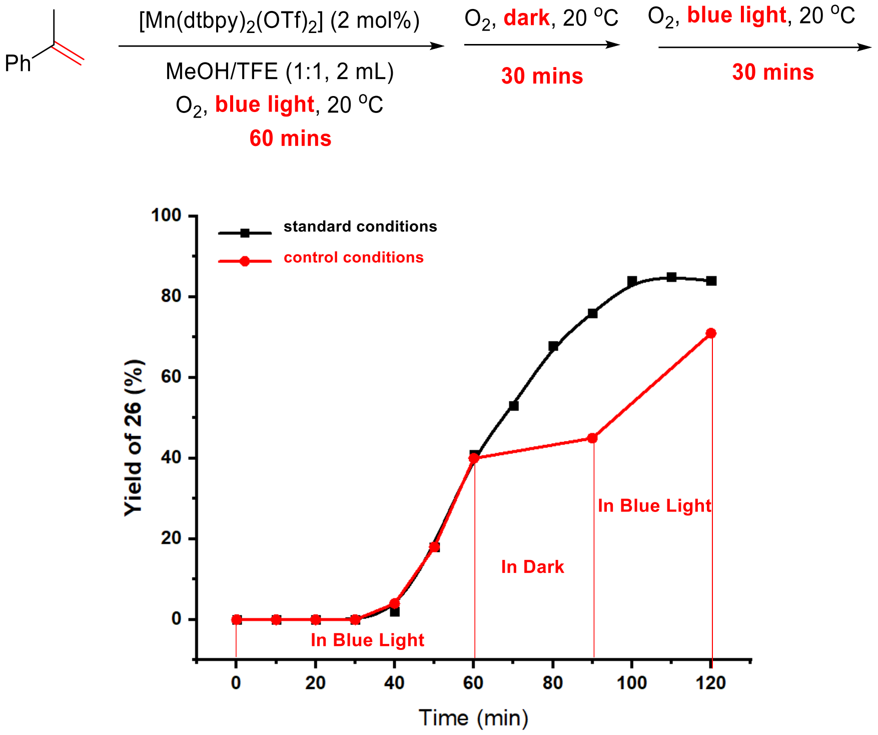

Fig. S12 Kinetic profile of oxidative cleavage of α-methylstyrene under light on/off conditions.

Oxidation with [Mn(dtbpy)$_2$(OTf)$_2$] and bis-μ-O_2-Mn_2 with light off

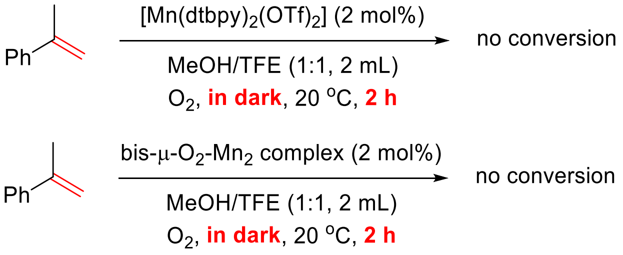

Scheme S4. Oxidative cleavage of α-methylstyrene by [Mn(dtbpy)$_2$(OTf)$_2$] and **bis-μ-O_2-Mn_2** in dark.

To an oven dried Schlenk tube, [Mn(dtbpy)$_2$(OTf)$_2$] (8.9 mg, 2 mol%) or **bis-μ-O_2-Mn_2** complex (14.6 mg, 2 mol%) and biphenyl (24.6 mg) as internal standard were added. The reaction tube was vacuumed and purged with oxygen via an oxygen balloon. Methanol (1 mL) and 2,2,2-trifluoroethanol (1 mL) was injected through a syringe. Finally, after the addition of *α*-methylstyrene (59 mg, 0.5 mmol), the reaction tube was

allowed to stir at 20 °C in dark. After 2 h, 0.1 mL of the reaction mixture was taken out for GC analysis. The GC result showed that acetophenone was not produced, and *α*-methylstyrene was wholly recovered.

5.4 UV-Vis experiments

PhIO oxidation: To an oven dried Schlenk tube, [Mn(dtbpy)$_2$(OTf)$_2$] (8.9 mg, 0.01 mmol) was added. The reaction tube was vacuumed and purged with nitrogen three times. Methanol (2 mL) was injected through a syringe. Then PhIO (6.6 mg, 0.03 mmol) was added under nitrogen, and the reaction mixture was stirred for 5 mins at 20 °C in dark. After that, 0.2 mL of the mixture was taken out and diluted with MeOH (3.8 mL). The diluted solution was then directly used for UV-Vis analysis.

O_2 activation: To an oven dried Schlenk tube, [Mn(dtbpy)$_2$(OTf)$_2$] (8.9 mg, 0.01 mmol) was added. The reaction tube was vacuumed and purged with oxygen via an oxygen balloon. Then methanol (2 mL) was injected through a syringe, and the reaction mixture was stirred for 1 h at 20 °C under blue light. After that, 0.2 mL of the mixture was taken out and diluted with MeOH (3.8 mL). The diluted solution was then directly used for UV-Vis analysis.

UV-Vis spectrum of* bis-μ-O_2-Mn_2 *complex: To an oven dried Schlenk tube, the **bis-μ-O_2-Mn_2** complex (1.5 mg, 0.001 mmol) was dissolved in methanol (4 mL). Then, the solution was directly used for UV-Vis analysis.

5.5 EPR experiments

5.5.1 Experimental methods: All solvents were of analytic grade and were purchased from Sigma-Aldrich (Dorset, UK). All EPR samples were prepared in methanol and acetonitrile solvents in an aerobic condition. Samples containing ~ 300 μM of [Mn(dtbpy)$_2$(OTf)$_2$] and **bis-μ-O_2-Mn_2** dimer complexes were transferred into 4 mm outer diameter/3 mm inner diameter Suprasil quartz EPR tubes (Wilmad LabGlass) and frozen in liquid N_2. The photoactivation of the **bis-μ-O_2-Mn_2** dimer in methanol and acetonitrile solvents was carried out at the specified temperature by placing the sample in a 1-propanol and dry-ice/liquid nitrogen solvent mixture. Optical irradiation at 455

nm was accomplished (for the specified duration described in the text/legend) using a Thorlabs Mounted High Power LED (M455L3) [https://www.thorlabs.com/thorproduct.cfm?partnumber=M455L3] with the output beam collimated using a Thorlabs collimation adaptor (SM2F32-A) [https://www.thorlabs.com/thorproduct.cfm?partnumber=SM2F32-A]. Optimal output (1 W typical) was maintained by driving with a constant current of 1A from a Thorlabs LED Driver [https://www.thorlabs.com/thorproduct.cfm?partnumber=LEDD1B]. All EPR samples were measured on a Bruker EMXplus EPR spectrometer equipped with a Bruker ER 4112SHQ/dual-mode X-band resonators. Sample cooling was achieved using a Bruker Stinger[21] cryogen free system mated to an Oxford Instruments ESR900 cryostat, and temperature control was maintained using an Oxford Instruments MercuryITC. The optimum conditions used for recording the spectra are given below; microwave power 10 dB (21.9 mW), modulation amplitude 5 G, time constant 82 ms, conversion time 10 ms, sweep time 120 s, receiver gain 30 dB and an average microwave frequency of 9.385 GHz. All annealing experiments were performed using a 1-propanol and dry-ice/liquid nitrogen solvent mixture, and the **bis-μ-O_2-Mn_2** dimer was annealed for the specified time duration (see main text/legend) at the temperature stated. All EPR spectra were measured as a frozen solution at 8 K/20 K, respectively. The analysis of the continuous wave EPR spectra were performed using EasySpin toolbox (5.2.28) for the Matlab program package.[22]

5.5.2 Results and discussion

Methanol solutions of [Mn(dtbpy)$_2$(OTf)$_2$] and **bis-μ-O_2-Mn_2** before and after blue light irradiation were monitored by electron paramagnetic resonance (EPR) spectroscopy. Low temperature EPR spectra of [Mn(dtbpy)$_2$(OTf)$_2$] in MeOH are dominated by a six-line spectrum at g = 2.0 and ^{55}Mn hyperfine coupling of 90 G (Fig. S13A), with much weaker features centred around 600 (g_{eff} ca. 10) and 1600 G (g_{eff} ca. 4) (Fig. S13D). The spectra are comparable in spread to those reported for the related *cis*-{$Mn^{II}N_4O_2$} complex [Mn(bipy)$_2$(CF_3CO_2)$_2$], for which the ZFS parameters $|D|$ = 0.038 cm^{-1}, E/D = 0.13 have been obtained from solid state X-band spectra.[23] In

parallel mode, the EPR spectrum is dominated by a feature from 1000-1700 G (alongside residual features of the g = 2 region), also consistent with the ZFS parameters above (Fig. S14A, C).

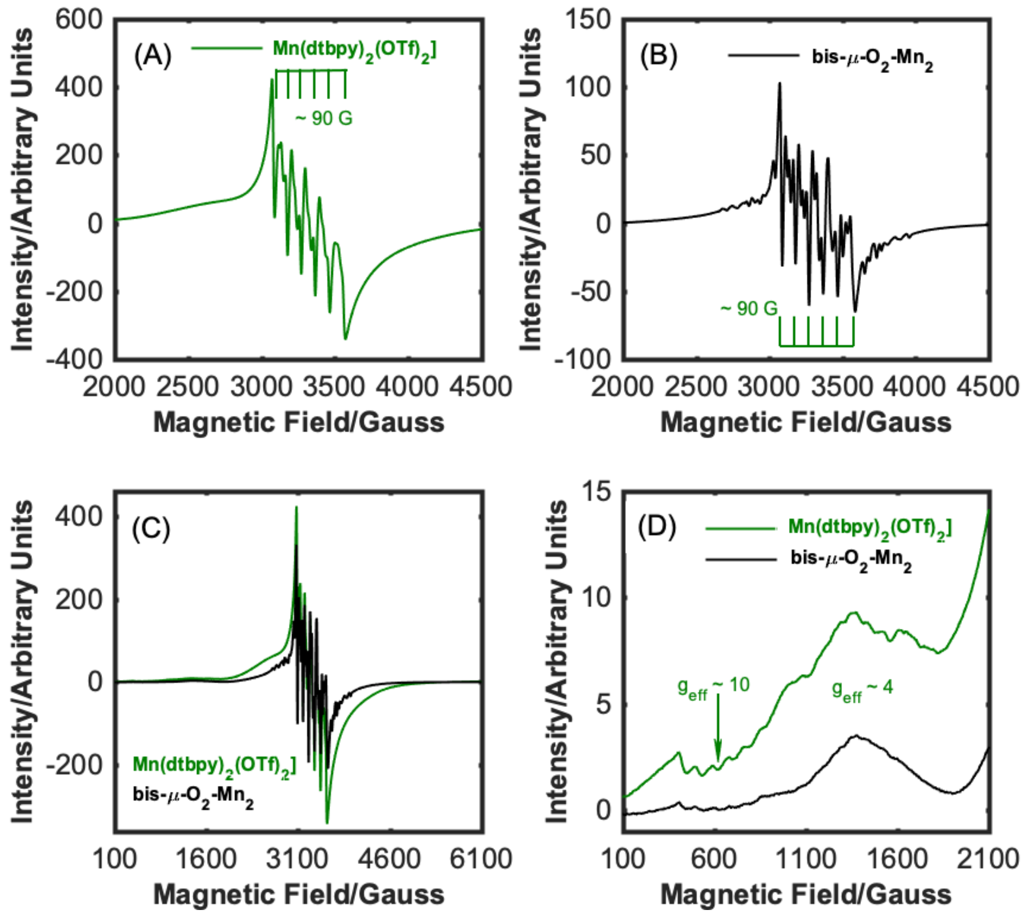

Fig. S13 Perpendicular mode cw-EPR spectra of [Mn(dtbpy)$_2$(OTf)$_2$] monomer in MeOH (A), and **bis-μ-O$_2$-Mn$_2$** dimer dissolved in cold (208 K) MeOH (B), and overlaid (C). The bottom right (D) panel is an expansion of the g_{eff} = 10 and g_{eff} = 4 regions of (C). *Conditions*; MW power 20 dB (monomer)/10 dB (dimer), MA 5G, time constant 82 ms, conversion time 10 ms, sweep time 120 s, receiver gain 30 dB, average microwave frequency 9.385 GHz, temperature 20 K.

A sample of **bis-μ-O$_2$-Mn$_2$** dissolved in cold (208 K) methanol gives a complex multi-line spectrum (Fig. S13B), which is consistent with the *S* = ½ ground state of an antiferromagnetically coupled Mn(III)Mn(IV) dimer.[24] The spectrum also has features consistent with that of [Mn(dtbpy)$_2$(OTf)$_2$] (Fig. S13B; green "goal-post"). Similar low-field features (Fig. S13D) are also observed, confirming a mixture of species in

solution. The sample of **bis-µ-O2-Mn2** in MeOH also has additional features observed in parallel-mode EPR spectra which are not present in spectra of $[Mn(dtbpy)_2(OTf)_2]$ (Fig. S14B). These are centred at ca. 800 G (g_{eff} ca. 8) with a small ^{55}Mn hyperfine coupling constant of ca. 42 G, which are values commonly observed for the $S = 2$, Mn(III) ion with large $|D|$.[25,26]

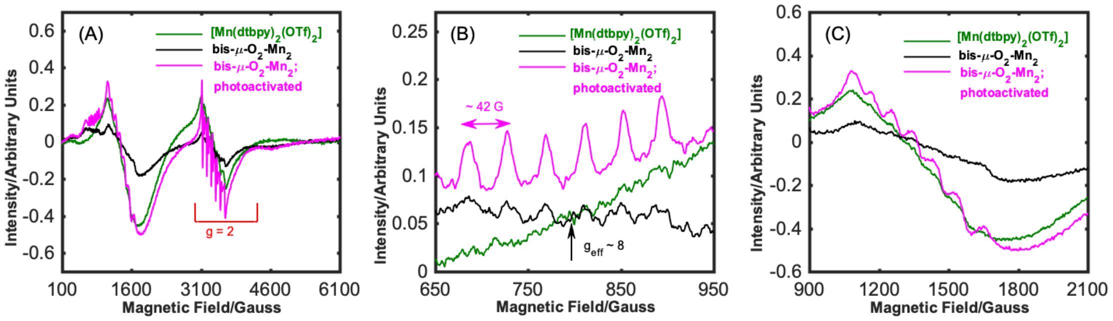

Fig. S14 Parallel mode cw-EPR spectra of $[Mn(dtbpy)_2(OTf)_2]$ (green traces) monomer in MeOH, **bis-µ-O2-Mn2** dimer dissolved in RT MeOH (black traces), and the latter after photoactivation with blue light at RT under air (magenta traces). The middle and right panels are an expansion of the $g_{eff} = 8$ and $g_{eff} = 4$ regions of the left-hand panel. *Conditions*; as described in **Fig. S13**, MW 23 db (1.1 mW), temperature 7 K.

Notably, irradiating/photoactivating the **bis-µ-O2-Mn2** sample (dissolved in cold-MeOH) with blue light at different temperatures between 210-275 K (5-40 minutes) increases the intensity of the $g_{eff} = 4$ signal, with a noticeable enhancement of hyperfine structure (ca. 90 G), along with the development of a pronounced shoulder at ca. 1000 G (indicated by the magenta arrow on the LHP in Fig. S15), whilst the multi-line structure in the $g = 2$ region is lost (indicated by the down and upwards magenta arrows on RHP in Fig. S15). This could be consistent with generation of high-valent species from the **bis-µ-O2-Mn2** dimer. A Mn(IV) monomer ($S = 3/2$) with large (>> 9 GHz, the microwave frequency) ZFS ($|D|$) would be expected to give signals in the $g_{eff} = 4$-6 region depending on the E/D ratio and similar signals have previously been assigned to Mn(IV).[27,28] The $g_{eff} = 8$, Mn(III) species is also observed in (parallel-mode) EPR spectra of irradiated samples with enhanced resolution (magenta trace in Fig. S14B) compared to the non-irradiated sample (black trace in Fig. S14B).

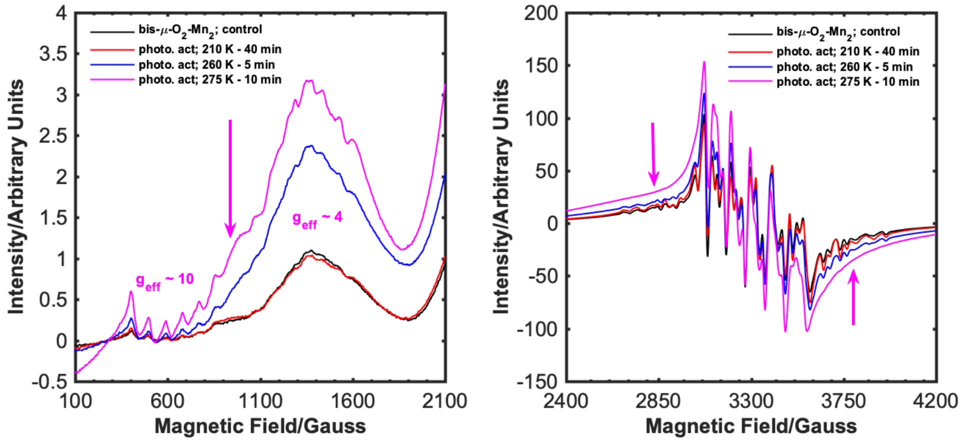

Fig. S15 Perpendicular mode, 20 K cw-EPR spectra of **bis-μ-O_2-Mn_2** dimer (black trace) dissolved in cold (208 K) MeOH, followed by irradiation with blue light under air for 40 (red trace), 5 (blue) and 10 minutes (magenta) at the specified temperatures. The left-hand panel shows the zoomed-in g_{eff} = 10 and g_{eff} = 4-6 regions of the spectra in the right-hand panel. *Conditions*; as described in Fig. S13.

Comparative studies in MeCN: The **bis-μ-O_2-Mn_2** dimer appears to be more robust in MeCN solution, giving very similar solution and powder spectra (Fig. S16). These spectra have weak low-field features at ca. 1600 G (g_{eff} ca. 4; middle panel in Fig. S16) in perpendicular mode, and from ca. 1000-1600 G in parallel mode: this again indicates that these low-field signals result from several species in solution. There are only subtle changes in the g_{eff} ca. 4-6 region under irradiation, and there is no evidence of the Mn(III) signature in the parallel mode spectra of any these samples.

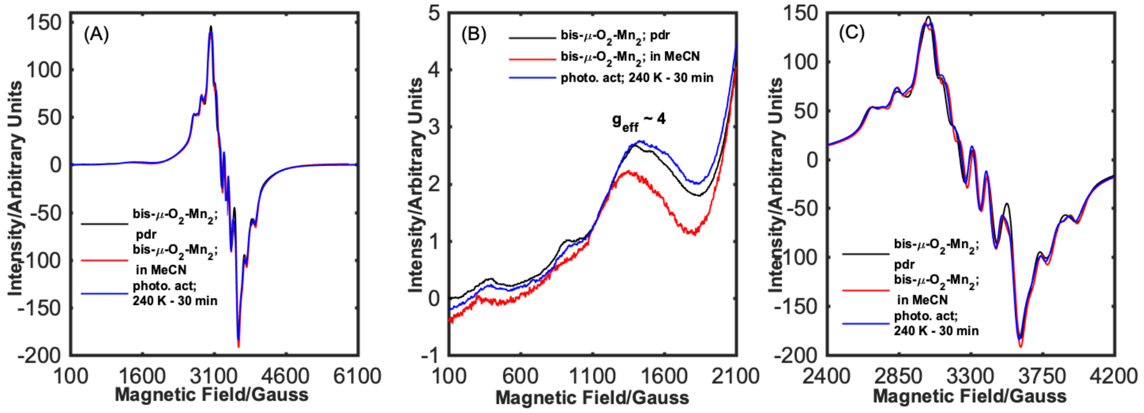

Fig. S16 Perpendicular mode cw-EPR spectra of **bis-μ-O_2-Mn_2** dimer as polycrystalline powders (black trace), dissolved in RT MeCN (red), followed by irradiation with blue light for 30 minutes at 240 K (blue). (B) and (C) show the zoomed-in g_{eff} = 4 and g = 2 regions of the spectra shown in (A). *Conditions*; as described in Fig. S13.

5.6 Additional information for suppoting the mechanism

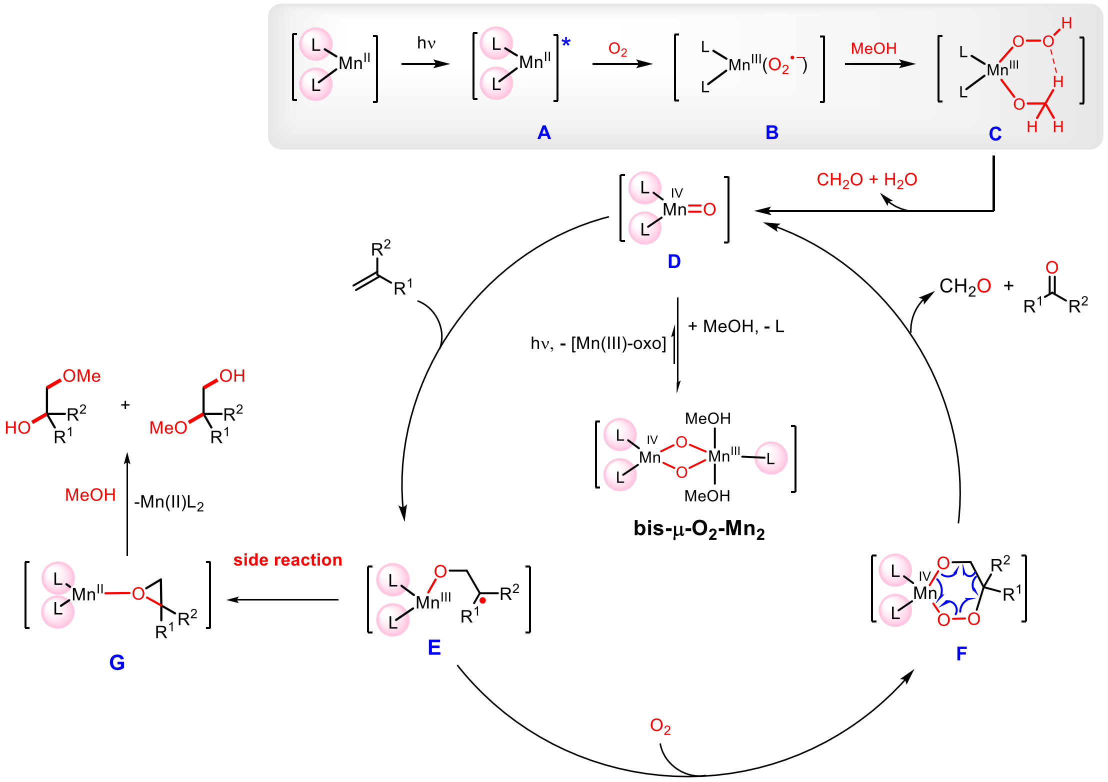

Scheme S5. Proposed mechanism. The mechanism includes an explanation for the formation of ring-opened side products. See below for the experimental observations (Sections 5.6.2-5.6.4).

5.6.1 Detection of formaldehyde

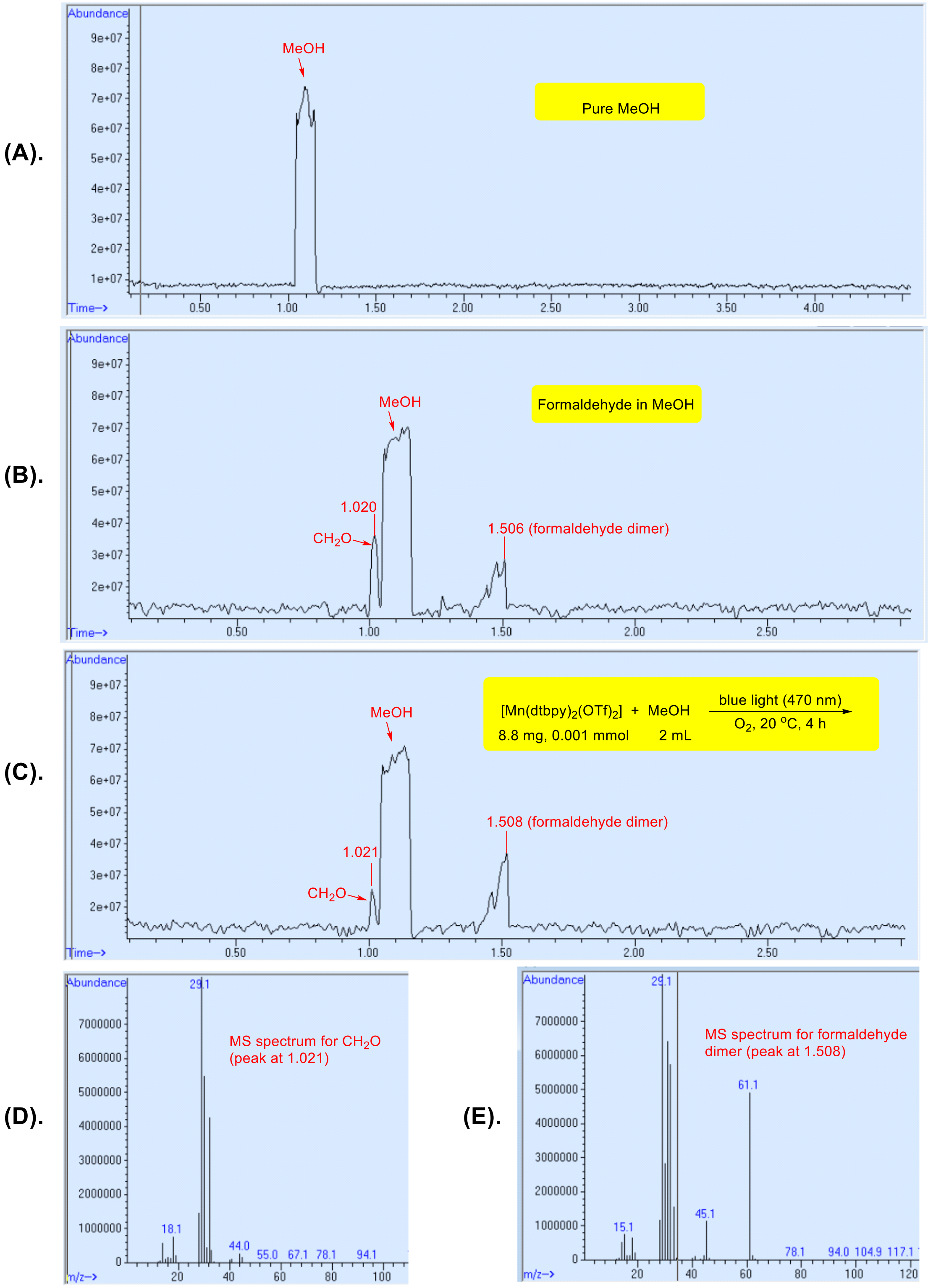

Fig. S17 (A) GC spectrum of pure MeOH; (B) GC spectrum of formaldehyde (CH_2O) in MeOH; (C) GC spectrum for the reaction solution of [Mn(dtbpy)$_2$(OTf)$_2$] in MeOH under O_2 and blue light for 4 hours at 20 °C; (D) MS spectrum of CH_2O; (E) MS spectrum of formaldehyde dimer.

5.6.2 Examples of the observation of side products

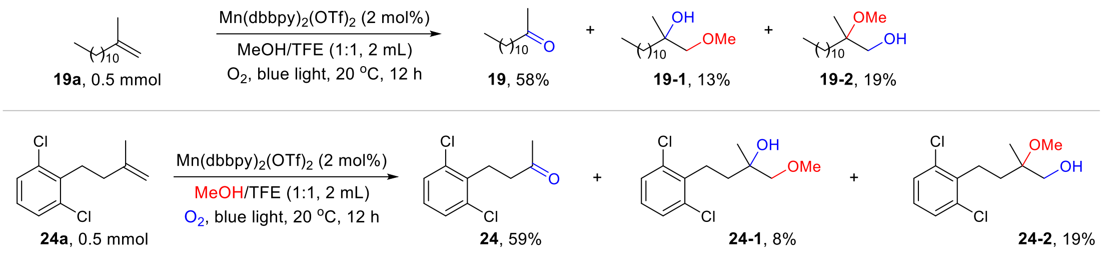

Formation of alkene hydroxymethoxylation side products was experimentally observed, which might result from the ring-opening of the corresponding epoxide intermediate with methanol (see Section 5.6.4). Their formation in the oxidation reaction of alkenes is indirect evidence to support the formation of intermediate **D/E** (Scheme S5).

5.6.3 Observation of epoxide formation

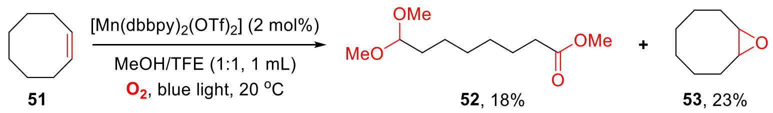

The formation of epoxide **53** in the oxidation reaction of cyclooctene **51** is indirect evidence to support the formation of intermediate **D/E** (Scheme S5).

5.6.4 Oxidation of epoxides

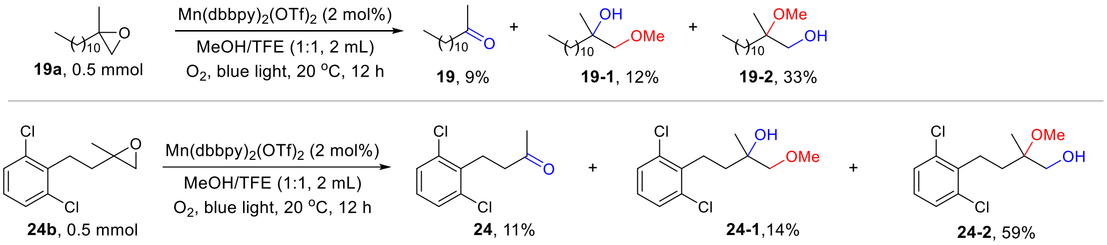

To an oven dried Schlenk tube, [Mn(dtbpy)$_2$(OTf)$_2$] (2 mol%, 8.8 mg) was added. Then the reaction tube was vacuumed and purged with oxygen via an oxygen balloon. Methanol (1 mL) and 2,2,2-trifluoroethanol (1 mL) was injected through a syringe. Finally, after the addition of epoxide (0.5 mmol), the reaction tube was allowed to stir at 20 °C under blue light for 12 h. After the crude mixture was concentrated under vacuum, the pure product was obtained by flash chromatography on silica gel with

hexane/ethyl acetate. The low yield of **19/24** suggests that epoxide may not be an intermediate during the Mn-catalyzed aerobic oxidative cleavage of alkenes.

5.6.5 The $^{16}O_2$-$^{18}O_2$ tracer experiments

The proposed mechanism is also supported by $^{16}O_2$-$^{18}O_2$ tracer experiments. As shown in Scheme S6, when the alkene **62** was oxidized with pure $^{16}O_2$ or $^{18}O_2$, only ^{16}O or ^{18}O labeled cleavage product was observed; but when a mixture of $^{16}O_2$-$^{18}O_2$ was used, all statistically possible products were formed. The result is consistent with the proposed mechanism, in which the two oxygen atoms in the product arise from two different O_2 molecules, rather one O_2 molecule.

$^{16}O_2$ tracer experiment: To an oven dried Schlenk tube, [Mn(dtbpy)$_2$(OTf)$_2$] (4.4 mg, 2 mol%) was added. Then the reaction tube was vacuumed and filled with $^{16}O_2$. Methanol (0.5 mL) and 2,2,2-trifluoroethanol (0.5 mL) was injected through a syringe under N_2. Finally, after the addition of alkene **62** (33.6 mg, 0.25 mmol), the reaction tube was allowed to stir at 20 ºC under blue light for 2 h. Then, degassed EtOAc (5 mL) was added to stop the reaction, and the diluted solution was analyzed by mass spectrometry.

$^{18}O_2$ tracer experiment: To an oven dried Schlenk tube, Mn(dtbpy)$_2$(OTf)$_2$ (4.4 mg, 2 mol%) was added. Then the reaction tube was vacuumed and filled with $^{18}O_2$. Methanol (0.5 mL) and 2,2,2-trifluoroethanol (0.5 mL) was injected through a syringe under N_2. Finally, after the addition of alkene **62** (33.6 mg, 0.25 mmol), the reaction tube was allowed to stir at 20 ºC under blue light for 2 h. Then, degassed EtOAc (5 mL) was added to stop the reaction, and the diluted solution was analyzed by mass spectrometry.

$^{16}O_2$-$^{18}O_2$ tracer experiment: To an oven dried Schlenk tube, Mn(dtbpy)$_2$(OTf)$_2$ (4.4 mg, 2 mol%) was added. Then the reaction tube was vacuumed and filled with $^{16}O_2$/$^{18}O_2$ (1:1 mixed, in volume). Methanol (0.5 mL) and 2,2,2-trifluoroethanol (0.5 mL) was injected through a syringe under N_2. Finally, after the addition of alkene **62** (33.6 mg, 0.25 mmol), the reaction tube was allowed to stir at 20 ºC under blue light for 2 h. Then, degassed EtOAc (5 mL) was added to stop the reaction, and the diluted solution was

analyzed by mass spectrometry.

$H_2{}^{18}O$ tracer experiment: To an oven dried Schlenk tube, Mn(dtbpy)$_2$(OTf)$_2$ (4.4 mg, 2 mol%) was added. Then the reaction tube was vacuumed and filled with $^{16}O_2$. Methanol (0.5 mL) and 2,2,2-trifluoroethanol (0.5 mL) was injected through a syringe under N_2. Finally, after the addition of alkene **62** (33.6 mg, 0.25 mmol) and $H_2{}^{18}O$ (10 uL), the reaction tube was allowed to stir at 20 °C under blue light for 2 h. Then, degassed EtOAc (5 mL) was added to stop the reaction, and the diluted solution was analyzed by mass spectrometry.

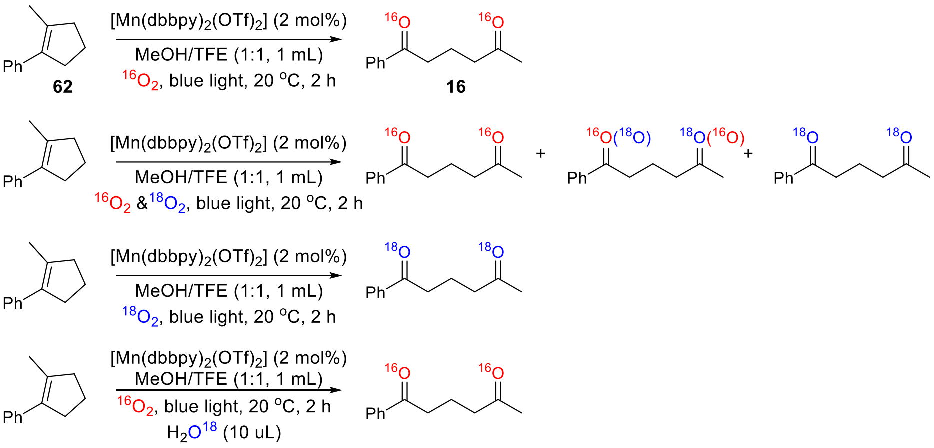

Scheme S6. $^{16}O_2$-$^{18}O_2$ tracer experiment.

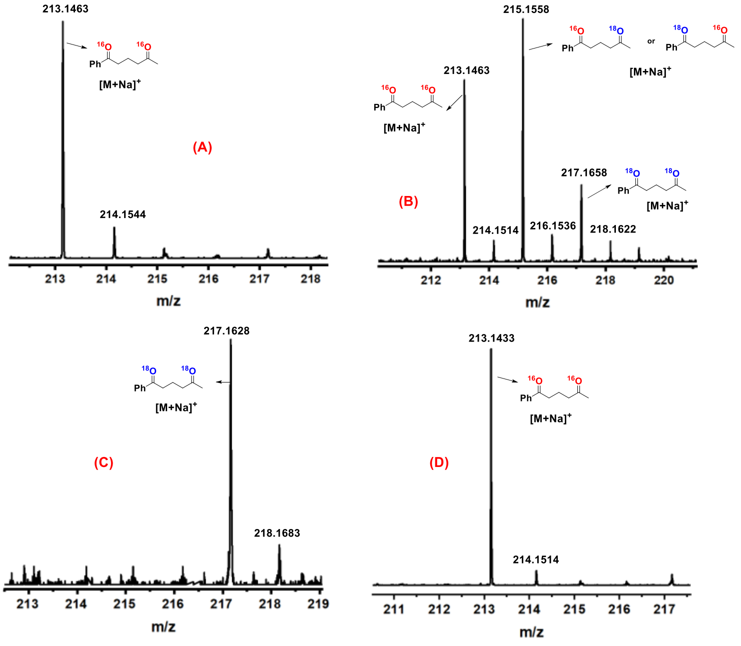

Fig. S18 MS spectra of ketone **16** from oxidative cleavage of alkene **62** under $^{16}O_2$ (**A**), under $^{16}O_2$/$^{18}O_2$ mixture (**B**), under $^{18}O_2$ (**C**), under $^{16}O_2$ with H_2O^{18} (**D**).

6. Analytical data of products

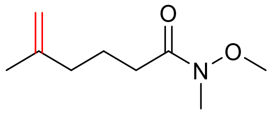

***N*-methoxy-*N*,5-dimethylhex-5-enamide (1a)**[29]

^{1}H NMR (400 MHz, $CDCl_3$) δ 4.73 (s, 1H), 4.70 (s, 1H),, 3.68 (s, 3H), 3.18 (s, 3H), 2.41 (t, *J* = 7.5 Hz, 2H), 2.07 (t, *J* = 7.5 Hz, 2H), 1.84 – 1.68 (m, 5H).

^{13}C NMR (101 MHz, $CDCl_3$) δ 174.52, 145.13, 110.31, 61.14, 37.25, 32.14, 31.23, 22.30, 22.19.

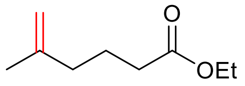

Ethyl 5-methylhex-5-enoate (2a)[30]

^{1}H NMR (400 MHz, $CDCl_3$) δ 4.73 (s, 1H), 4.69 (s, 1H), 4.13 (q, *J* = 7.1 Hz, 2H), 2.29 (t, *J* = 7.5 Hz, 2H), 2.05 (t, *J* = 7.5 Hz, 2H), 1.83 – 1.69 (m, 5H), 1.26 (t, *J* = 7.1 Hz, 3H).

^{13}C NMR (101 MHz, $CDCl_3$) δ 173.63, 144.77, 110.57, 60.17, 37.03, 33.70, 22.75, 22.15, 14.21.

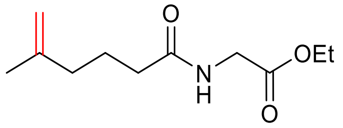

Ethyl (5-methylhex-5-enoyl)glycinate (3a)

^{1}H NMR (400 MHz, $CDCl_3$) δ 6.02 (s, 1H), 4.74 (s, 1H), 4.70 (s, 1H), 4.22 (q, *J* = 7.1 Hz, 2H), 4.04 (d, *J* = 5.1 Hz, 2H), 2.24 (t, *J* = 7.5 Hz, 2H), 2.06 (t, *J* = 7.4 Hz, 2H), 1.85 – 1.76 (m, 2H), 1.72 (s, 3H), 1.29 (t, *J* = 7.1 Hz, 3H).

^{13}C NMR (101 MHz, $CDCl_3$) δ 173.04, 170.11, 144.85, 110.69, 61.52, 41.34, 37.03, 35.58, 23.20, 22.16, 14.13.

HRMS (CI) calcd for $C_{11}H_{19}NO_3$ $[M+H]^+$: 214.1438; found: 214.1441.

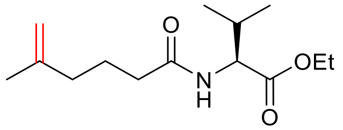

Ethyl (5-methylhex-5-enoyl)-*L*-valinate (4a)

^{1}H NMR (400 MHz, $CDCl_3$) δ 5.99 (s, 1H), 4.74 (s, 1H), 4.70 (s, 1H), 4.57 (dd, *J* = 8.7, 4.8 Hz, 1H), 4.26 – 4.13 (m, 2H), 2.28 – 2.00 (m, 5H), 1.83 – 1.76 (m, 2H), 1.71 (s, 3H), 1.29 (t, *J* = 7.1 Hz, 3H), 0.97 – 0.88 (m, 6H).

^{13}C NMR (101 MHz, $CDCl_3$) δ 172.77, 172.18, 144.85, 110.66, 61.19, 56.78, 37.02, 35.85, 31.31, 23.31, 22.10, 18.88, 17.74, 14.17.

HRMS (CI) calcd for $C_{14}H_{25}NO_3$ $[M+H]^+$: 256.1907; found: 256.1918.

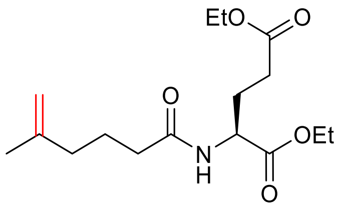

Diethyl (5-methylhex-5-enoyl)-*L*-glutamate (5a)

^{1}H NMR (400 MHz, $CDCl_3$) δ 6.23 (s, 1H), 4.73 (s, 1H), 4.69 (s, 1H), 4.64 – 4.55 (m, 1H), 4.26 – 4.07 (m, 4H), 2.50 – 2.29 (m, 2H), 2.26 – 1.94 (m, 6H), 1.87 – 1.75 (m, 2H), 1.71 (s, 3H), 1.34 – 1.22 (m, 6H).

^{13}C NMR (101 MHz, $CDCl_3$) δ 172.92, 172.84, 172.02, 144.82, 110.69, 61.61, 60.71, 51.62, 37.04, 35.71, 30.37, 27.39, 23.20, 22.14, 14.15, 14.11.

HRMS (ESI) calcd for $C_{16}H_{27}NO_5$ $[M+Na]^+$: 336.1781; found: 336.1784.

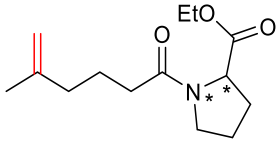

Ethyl (5-methylhex-5-enoyl)prolinate (6a, dr = 79 :21)

^{1}H NMR (400 MHz, $CDCl_3$) δ 4.78 – 4.63 (m, 2H), 4.47 (dd, *J* = 8.5, 3.6 Hz, 0.79H), 4.38 (dd, *J* = 8.5, 2.5 Hz, 0.21H), 4.24 – 4.13 (m, 2H), 3.69 – 3.45 (m, 2H), 2.40 – 1.75 (m, 10H), 1.74 – 1.67 (m, 3H), 1.33 – 1.23 (m, 3H).

^{13}C NMR (101 MHz, $CDCl_3$) δ 172.47, 172.33, 171.95, 171.80, 145.21, 110.39, 110.34, 61.51, 60.99, 59.51, 58.72, 46.98, 46.33, 37.22, 37.16, 33.62, 33.59, 31.49, 29.23, 24.75, 22.57, 22.34, 22.22, 22.19, 14.18, 14.14.

HRMS (CI) calcd for $C_{14}H_{23}NO_3$ $[M+H]^+$: 254.1751; found: 254.1761.

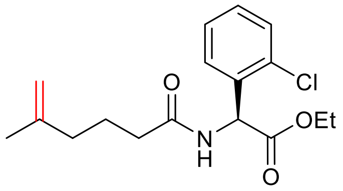

Ethyl (*S*)-2-(2-chlorophenyl)-2-(5-methylhex-5-enamido)acetate (7a)

^{1}H NMR (400 MHz, $CDCl_3$) δ 7.47 – 7.33 (m, 2H), 7.31 – 7.21 (m, 2H), 6.56 (d, *J* = 6.8 Hz, 1H), 5.90 (d, *J* = 7.4 Hz, 1H), 4.71 (s, 1H), 4.66 (s, 1H), 4.29 – 4.12 (m, 2H), 2.30 – 2.16 (m, 2H), 2.02 (t, *J* = 7.4 Hz, 2H), 1.78 (dt, *J* = 14.2, 7.2 Hz, 2H), 1.68 (s, 3H), 1.20 (t, *J* = 7.1 Hz, 3H).

^{13}C NMR (101 MHz, $CDCl_3$) δ 172.17, 170.34, 144.80, 134.98, 133.46, 130.39, 130.08, 129.59, 127.19, 110.68, 62.10, 54.72, 36.90, 35.43, 23.02, 22.08, 13.96.

HRMS (CI) calcd for $C_{17}H_{22}ClNO_3$ $[M+H]^+$: 324.1361; found: 324.1369.

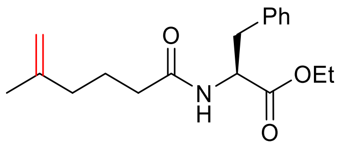

Ethyl (5-methylhex-5-enoyl)-*L*-phenylalaninate (8a)

^{1}H NMR (400 MHz, $CDCl_3$) δ 7.34 – 7.20 (m, 3H), 7.11 (d, *J* = 7.0 Hz, 2H), 5.89 (d, *J* = 6.3 Hz, 1H), 4.88 (dd, *J* = 12.9, 6.0 Hz, 1H), 4.72 (s, 1H), 4.65 (s, 1H), 4.17 (q, *J* = 7.1 Hz, 2H), 3.22 – 3.02 (m, 2H), 2.16 (t, *J* = 7.4 Hz, 2H), 2.01 (t, *J* = 7.3 Hz, 2H), 1.82 – 1.61 (m, 5H), 1.25 (t, *J* = 7.1 Hz, 3H).

^{13}C NMR (101 MHz, $CDCl_3$) δ 172.36, 171.69, 144.80, 135.91, 129.28, 128.47, 127.04, 110.63, 61.47, 52.92, 37.96, 36.97, 35.73, 23.18, 22.14, 14.09.

HRMS (ESI) calcd for $C_{18}H_{25}NO_3$ $[M+Na]^+$: 326.1727; found: 326.1730.

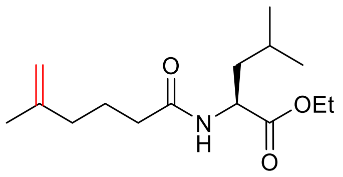

Ethyl (5-methylhex-5-enoyl)-*L*-leucinate (9a)

^{1}H NMR (400 MHz, $CDCl_3$) δ 5.89 (d, *J* = 7.8 Hz, 1H), 4.73 (s, 1H), 4.69 (s, 1H), 4.64 (td, *J* = 8.6, 4.9 Hz, 1H), 4.19 (q, *J* = 7.1 Hz, 2H), 2.21 (t, *J* = 7.5 Hz, 2H), 2.05 (t, *J* = 7.5 Hz, 2H), 1.86 – 1.74 (m, 2H), 1.71 (s, 3H), 1.68 – 1.59 (m, 2H), 1.58 – 1.46 (m, 1H), 1.28 (t, *J* = 7.1 Hz, 3H), 1.00 – 0.91 (m, 6H).

^{13}C NMR (101 MHz, $CDCl_3$) δ 173.26, 172.60, 144.87, 110.64, 61.26, 50.58, 41.80, 36.99, 35.71, 24.86, 23.20, 22.75, 22.10, 21.97, 14.10.

HRMS (CI) calcd for $C_{15}H_{27}NO_3$ $[M+H]^+$: 270.2064; found: 270.2073.

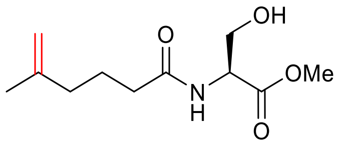

Methyl (5-methylhex-5-enoyl)-*L*-serinate (10a)

^{1}H NMR (400 MHz, $CDCl_3$) δ 6.65 (d, *J* = 7.1 Hz, 1H), 4.79 – 4.61 (m, 3H), 4.04 – 3.85 (m, 2H), 3.78 (s, 3H), 3.29 (br, 1H), 2.26 (t, *J* = 7.5 Hz, 2H), 2.06 (t, *J* = 7.4 Hz, 2H), 1.86 – 1.75 (m, 2H), 1.72 (s, 3H).

^{13}C NMR (101 MHz, $CDCl_3$) δ 173.59, 171.08, 144.79, 110.74, 63.27, 54.62, 52.68, 37.01, 35.65, 23.20, 22.15.

HRMS (CI) calcd for $C_{11}H_{19}NO_4$ $[M+H]^+$: 230.1387; found: 230.1394.

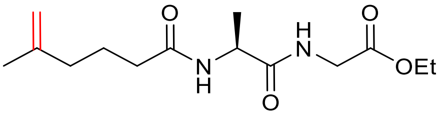

Ethyl (5-methylhex-5-enoyl)-*L*-alanylglycinate (11a)

^{1}H NMR (400 MHz, $CDCl_3$) δ 6.92 (s, 1H), 6.24 (d, *J* = 7.2 Hz, 1H), 4.73 (s, 1H), 4.68 (s, 1H), 4.58 (dd, *J* = 14.2, 7.1 Hz, 1H), 4.20 (q, *J* = 7.1 Hz, 2H), 4.01 (d, *J* = 5.4 Hz, 2H), 2.20 (t, *J* = 7.6 Hz, 2H), 2.04 (t, *J* = 7.5 Hz, 2H), 1.84 – 1.73 (m, 2H), 1.71 (s, 3H), 1.40 (d, *J* = 7.0 Hz, 3H), 1.28 (t, *J* = 7.1 Hz, 3H).

^{13}C NMR (101 MHz, $CDCl_3$) δ 172.95, 172.62, 169.51, 144.77, 110.70, 61.48, 48.47, 41.30, 37.02, 35.68, 23.16, 22.12, 18.21, 14.10.

HRMS (ESI) calcd for $C_{14}H_{24}N_2O_4$ $[M+Na]^+$: 307.1628; found: 307.1633.

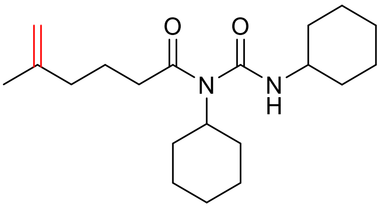

***N*-cyclohexyl-*N*-(cyclohexylcarbamoyl)-5-methylhex-5-enamide (12a)**

^{1}H NMR (400 MHz, $CDCl_3$) δ 7.03 (s, 1H), 4.73 (s, 1H), 4.68 (s, 1H), 4.02 – 3.81 (m, 1H), 3.76 – 3.58 (m, 1H), 2.40 (t, *J* = 7.5 Hz, 2H), 2.06 (t, *J* = 7.4 Hz, 2H), 2.00 – 1.54 (m, 17H), 1.46 – 1.06 (m, 8H).

^{13}C NMR (101 MHz, $CDCl_3$) δ 173.26, 172.60, 144.87, 110.64, 61.26, 50.58, 41.80, 36.99, 35.71, 24.86, 23.20, 22.75, 22.10, 21.97, 14.10.

HRMS (ESI) calcd for $C_{20}H_{34}N_2O_2$ $[M+Na]^+$: 357.2512; found: 357.2517.

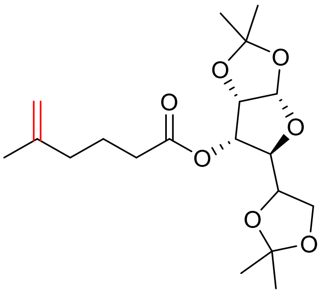

(3a*S*,5*S*,6*S*,6a*S*)-5-(2,2-dimethyl-1,3-dioxolan-4-yl)-2,2-dimethyltetrahydrofuro[2,3-d][1,3]dioxol-6-yl 5-methylhex-5-enoate (13a)

^{1}H NMR (400 MHz, $CDCl_3$) δ 5.87 (d, J = 3.6 Hz, 1H), 5.28 (s, 1H), 4.74 (s, 1H), 4.68 (s, 1H), 4.48 (d, J = 3.6 Hz, 1H), 4.21 (s, 2H), 4.12 – 3.98 (m, 2H), 2.35 (td, J = 7.3, 1.9 Hz, 2H), 2.06 (t, J = 7.5 Hz, 2H), 1.79 (p, J = 7.5 Hz, 2H), 1.71 (s, 3H), 1.52 (s, 3H), 1.41 (s, 3H), 1.31 (s, 6H).

^{13}C NMR (101 MHz, $CDCl_3$) δ 172.17, 144.50, 112.27, 110.83, 109.31, 105.05, 83.37, 79.87, 75.88, 72.42, 67.27, 36.88, 33.53, 26.80, 26.72, 26.18, 25.22, 22.59, 22.14.

HRMS (ESI) calcd for $C_{19}H_{30}O_7$ $[M+Na]^+$: 393.1884; found: 393.1889.

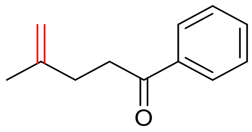

4-Methyl-1-phenylpent-4-en-1-one (15a)[31]

^{1}H NMR (400 MHz, $CDCl_3$) δ 7.97 (d, J = 7.4 Hz, 2H), 7.56 (t, J = 7.3 Hz, 1H), 7.46 (t, J = 7.5 Hz, 2H), 4.77 (s, 1H), 4.72 (s, 1H), 3.15 – 3.11 (m, 2H), 2.47 – 2.43 (m, 2H), 1.79 (s, 3H).

^{13}C NMR (101 MHz, $CDCl_3$) δ 199.64, 144.63, 136.91, 132.93, 128.54, 127.98, 110.15, 36.78, 31.83, 22.70.

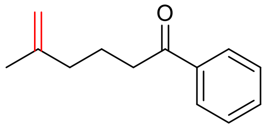

5-Methyl-1-phenylhex-5-en-1-one (16a)[29]

^{1}H NMR (400 MHz, $CDCl_3$) δ 7.89 (d, J = 7.8 Hz, 2H), 7.49 (t, J = 7.2 Hz, 1H), 7.39 (t, J = 7.6 Hz, 2H), 4.68 (s, 1H), 4.64 (s, 1H), 2.90 (t, J = 7.3 Hz, 2H), 2.05 (t, J = 7.5 Hz, 2H), 1.83 (p, J = 7.3 Hz, 2H), 1.67 (s, 3H).

^{13}C NMR (101 MHz, $CDCl_3$) δ 200.30, 145.10, 137.09, 132.90, 128.55, 128.01, 110.60, 37.82, 37.18, 22.23, 21.92.

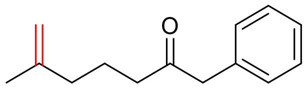

6-Methyl-1-phenylhept-6-en-2-one (17a)[32]

^{1}H NMR (400 MHz, $CDCl_3$) δ 7.36 – 7.18 (m, 5H), 4.69 (s, 1H), 4.61 (s, 1H), 3.67 (s, 2H), 2.44 (t, J = 7.3 Hz, 2H), 1.95 (t, J = 7.5 Hz, 2H), 1.78 – 1.60 (m, 5H).

^{13}C NMR (101 MHz, $CDCl_3$) δ 208.23, 144.94, 134.28, 129.35, 128.64, 126.92, 110.48, 50.16, 41.16, 36.90, 22.08, 21.33.

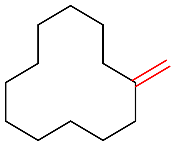

Methylenecyclododecane (18a)[33]

^{1}H NMR (400 MHz, $CDCl_3$) δ 4.79 (s, 2H), 2.11 – 2.00 (m, 4H), 1.57 – 1.43 (m, 4H), 1.38 – 1.22 (m, 14H).

^{13}C NMR (101 MHz, $CDCl_3$) δ 147.45, 110.33, 33.07, 24.46, 24.14, 23.72, 23.28, 22.63.

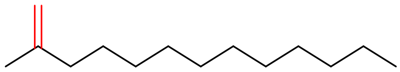

2-Methyltridec-1-ene (19a)[34]

^{1}H NMR (400 MHz, $CDCl_3$) δ4.74 – 4.63 (m, 2H), 2.00 (t, J = 7.6 Hz, 2H), 1.71 (s, 3H), 1.47 – 1.36 (m, 2H), 1.26 (s, 16H), 0.88 (t, J = 6.7 Hz, 3H).

^{13}C NMR (101 MHz, $CDCl_3$) δ 146.32, 109.50, 37.87, 31.95, 29.71, 29.66, 29.58, 29.38, 29.36, 27.67, 22.71, 22.38, 14.11.

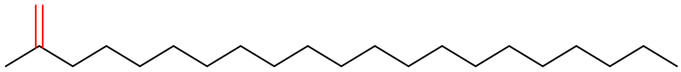

2-Methylhenicos-1-ene (20a)

^{1}H NMR (400 MHz, $CDCl_3$) δ 4.72 – 4.61 (m, 2H), 2.00 (t, J = 7.6 Hz, 2H), 1.71 (s, 3H), 1.46 – 1.36 (m, 2H), 1.32 – 1.22 (m, 32H), 0.88 (t, J = 6.7 Hz, 3H).

^{13}C NMR (101 MHz, $CDCl_3$) δ 146.36, 109.48, 37.86, 31.94, 29.71, 29.67, 29.65, 29.56, 29.37, 29.35, 27.66, 22.70, 22.39, 14.12.

HRMS (CI) calcd for $C_{22}H_{44}$ $[M+NH_4]^+$: 326.3781; found: 326.3784.

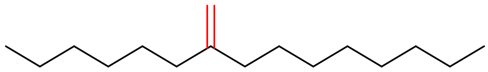

7-Methylenepentadecane (21a)[35]

^{1}H NMR (400 MHz, $CDCl_3$) δ 4.72 (s, 2H), 2.07 – 1.97 (m, 4H), 1.51 – 1.21 (m, 20H), 0.96 – 0.86 (m, 6H).

^{13}C NMR (101 MHz, $CDCl_3$) δ 150.42, 108.32, 36.11, 31.93, 31.82, 29.56, 29.49, 29.33, 29.15, 27.86, 27.82, 22.70, 22.67, 14.10.

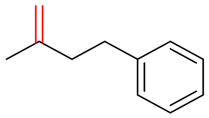

(3-Methylbut-3-en-1-yl)benzene (23a)[36]

^{1}H NMR (400 MHz, $CDCl_3$) δ 7.27 (t, *J* = 7.5 Hz, 2H), 7.24 – 7.14 (m, 3H), 4.74 (s, 1H), 4.71 (s, 1H), 2.82 – 2.69 (m, 2H), 2.38 – 2.25 (m, 2H), 1.77 (s, 3H).

^{13}C NMR (101 MHz, $CDCl_3$) δ 145.36, 142.20, 128.30, 128.27, 125.74, 110.18, 39.59, 34.24, 22.59.

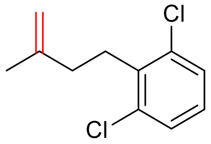

1,3-Dichloro-2-(3-methylbut-3-en-1-yl)benzene (24a)

^{1}H NMR (400 MHz, $CDCl_3$) δ 7.25 (d, *J* = 8.0 Hz, 2H), 7.04 (t, *J* = 8.0 Hz, 1H), 4.78 (s, 2H), 3.04 (dd, *J* = 10.0, 6.8 Hz, 2H), 2.34 – 2.15 (m, 2H), 1.83 (s, 3H).

^{13}C NMR (101 MHz, $CDCl_3$) δ 145.08, 137.98, 135.26, 128.08, 127.45, 110.40, 35.89, 30.17, 22.36.

HRMS (CI) calcd for $C_{11}H_{12}Cl_2$ $[M+NH_4]^+$: 232.0654; found: 232.0647.

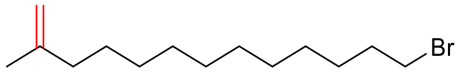

13-Bromo-2-methyltridec-1-ene (25a)

^{1}H NMR (400 MHz, $CDCl_3$) δ 4.61 (s, 1H), 4.59 (s, 1H), 3.34 (t, *J* = 6.9 Hz, 2H), 1.93 (t, *J* = 7.5 Hz, 2H), 1.83 – 1.73 (m, 2H), 1.64 (s, 3H), 1.41 – 1.29 (m, 4H), 1.28 – 1.14 (m, 12H).

^{13}C NMR (101 MHz, $CDCl_3$) δ 146.33, 109.50, 37.83, 34.04, 32.84, 29.55, 29.51, 29.50, 29.42, 29.30, 28.76, 28.17, 27.62, 22.39.

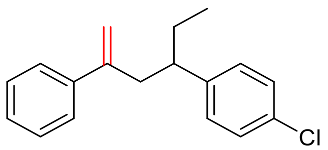

1-Chloro-4-(5-phenylhex-5-en-3-yl)benzene (44a)

^{1}H NMR (400 MHz, $CDCl_3$) δ 7.36 – 7.24 (m, 5H), 7.21 (d, *J* = 8.3 Hz, 2H), 6.96 (d, *J* = 8.2 Hz, 2H), 5.13 (d, *J* = 1.3 Hz, 1H), 4.85 (s, 1H), 2.92 – 2.80 (m, 1H), 2.72 – 2.61 (m, 1H), 2.57 – 2.43 (m, 1H), 1.89 – 1.60 (m, 1H), 1.61 – 1.41 (m, 1H), 0.78 – 0.62 (m, 3H).

^{13}C NMR (101 MHz, $CDCl_3$) δ 146.58, 143.49, 141.02, 131.42, 129.08, 128.28, 128.21, 127.33, 126.32, 114.46, 45.24, 42.88, 28.74, 11.93.

HRMS (CI) calcd for $C_{18}H_{19}Cl$ $[M+H]^+$: 271.1248; found: 271.1255.

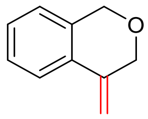

4-Methyleneisochromane (45a)[24]

^{1}H NMR (400 MHz, $CDCl_3$) δ 7.60 (dd, *J* = 5.7, 3.5 Hz, 1H), 7.20 – 7.08 (m, 2H), 6.94 (dd, *J* = 5.4, 3.6 Hz, 1H), 5.52 (s, 1H), 4.93 (s, 1H), 4.72 (s, 2H), 4.36 (s, 2H).

^{13}C NMR (101 MHz, $CDCl_3$) δ 138.22, 134.49, 130.97, 127.98, 126.88, 124.57, 123.35, 106.80, 70.91, 68.88.

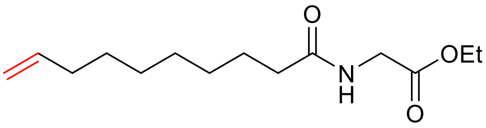

Ethyl dec-9-enoylglycinate (49)

^{1}H NMR (400 MHz, $CDCl_3$) δ 6.01 (s, 1H), 5.80 (ddt, *J* = 16.9, 10.2, 6.7 Hz, 1H), 5.03 - 4.88 (m, 2H), 4.22 (q, *J* = 7.1 Hz, 2H), 4.03 (d, *J* = 5.1 Hz, 2H), 2.24 (t, *J* = 7.6 Hz, 2H), 2.03 (q, *J* = 6.9 Hz, 2H), 1.73 – 1.59 (m, 2H), 1.44 – 1.22 (m, 11H).

^{13}C NMR (101 MHz, $CDCl_3$) δ 173.21, 170.13, 139.07, 114.14, 61.47, 41.30, 36.37, 33.70, 29.14, 29.12, 28.88, 28.80, 25.50, 14.09.

HRMS (ESI) calcd for $C_{14}H_{25}NO_3$ $[M+Na]^+$: 278.1727; found: 278.1728.

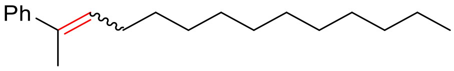

Tetradec-2-en-2-ylbenzene (E & Z, 54)[37]

^{1}H NMR (400 MHz, $CDCl_3$) δ 7.41 – 7.13 (m, 5H), 5.78 (t, *J* = 7.2 Hz, 0.15H), 5.45 (t, *J* = 7.3 Hz, 0.80H), 2.19 (q, *J* = 7.3 Hz, 0.37H), 2.02 (s, 3H), 1.95 (q, *J* = 7.3 Hz, 1.58H), 1.33 – 1.17 (m, 18H), 0.88 (t, *J* = 6.7 Hz, 3H).

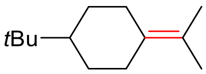

1-(*tert*-Butyl)-4-(propan-2-ylidene)cyclohexane (59)[38]

^{1}H NMR (400 MHz, $CDCl_3$) δ 2.74 (d, *J* = 14.0 Hz, 2H), 1.84 (d, *J* = 12.7 Hz, 2H), 1.77 – 1.60 (m, 8H), 1.24 – 1.12 (m, 1H), 1.06 – 0.92 (m, 2H), 0.87 (s, 9H).

^{13}C NMR (101 MHz, $CDCl_3$) δ 131.98, 119.85, 48.43, 32.45, 30.17, 28.55, 27.64, 19.91.

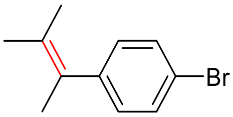

1-Bromo-4-(3-methylbut-2-en-2-yl)benzene (61)

^{1}H NMR (400 MHz, $CDCl_3$) δ 7.41 (d, *J* = 8.3 Hz, 2H), 6.99 (d, *J* = 8.3 Hz, 2H), 1.92 (s, 3H), 1.79 (s, 3H), 1.57 (s, 3H).

^{13}C NMR (101 MHz, $CDCl_3$) δ 144.16, 131.02, 130.20, 128.85, 128.02, 119.48, 22.02, 20.61, 20.55.

HRMS (CI) calcd for $C_{11}H_{13}Br$ $[M+H]^+$: 225.0273; found: 225.0265.

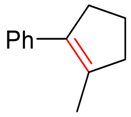

(2-Methylcyclopent-1-en-1-yl)benzene (62)[39]

^{1}H NMR (400 MHz, $CDCl_3$) δ 7.40 – 7.22 (m, 5H), 2.78 – 2.66 (m, 2H), 2.49 (t, *J* = 7.4 Hz, 2H), 1.96 – 1.80 (m, 5H).

^{13}C NMR (101 MHz, $CDCl_3$) δ 138.79, 135.24, 134.82, 128.02, 127.64, 126.03, 40.15, 37.30, 21.91, 15.51.

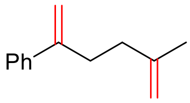

(5-Methylhexa-1,5-dien-2-yl)benzene (63)[40]

^{1}H NMR (400 MHz, $CDCl_3$) δ 7.43 – 7.37 (m, 2H), 7.32 (t, *J* = 7.3 Hz, 2H), 7.29 – 7.21 (m, 1H), 5.27 (s, 1H), 5.07 (s, 1H), 4.73 (s, 1H), 4.68 (s, 1H), 2.70 – 2.58 (m, 2H), 2.23 – 2.11 (m, 2H), 1.74 (s, 3H).

^{13}C NMR (101 MHz, $CDCl_3$) δ 148.26, 145.42, 141.23, 128.25, 127.31, 126.09, 112.26, 110.01, 36.53, 33.61, 22.52.

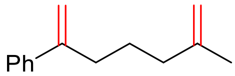

(6-Methylhepta-1,6-dien-2-yl)benzene (64)

^{1}H NMR (400 MHz, $CDCl_3$) δ 7.40 (d, *J* = 7.5 Hz, 2H), 7.32 (t, *J* = 7.4 Hz, 2H), 7.28 – 7.21 (m, 1H), 5.27 (s, 1H), 5.06 (s, 1H), 4.70 (s, 1H), 4.66 (s, 1H), 2.54 – 2.43 (m, 2H), 2.04 (t, *J* = 7.6 Hz, 2H), 1.69 (s, 3H), 1.65 – 1.53 (m, 2H).

^{13}C NMR (101 MHz, $CDCl_3$) δ 148.49, 145.64, 141.37, 128.23, 127.26, 126.10, 112.25, 110.03, 37.34, 34.85, 26.10, 22.34.

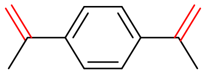

1,4-Di(prop-1-en-2-yl)benzene (65)[41]

^{1}H NMR (400 MHz, $CDCl_3$) δ 7.44 (s, 4H), 5.38 (s, 2H), 5.08 (s, 2H), 2.15 (s, 6H).

^{13}C NMR (101 MHz, $CDCl_3$) δ 142.80, 140.20, 125.31, 112.25, 21.73.

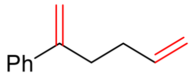

Hexa-1,5-dien-2-ylbenzene (67)[42]

^{1}H NMR (400 MHz, $CDCl_3$) δ 7.39 (d, J = 7.4 Hz, 2H), 7.31 (t, J = 7.5 Hz, 2H), 7.28 – 7.19 (m, 1H), 5.83 (ddt, J = 16.9, 10.2, 6.6 Hz, 1H), 5.28 (s, 1H), 5.06 (s, 1H), 4.98 (t, J = 15.1 Hz, 2H), 2.67 – 2.54 (m, 2H), 2.30 – 2.12 (m, 2H).

^{13}C NMR (101 MHz, $CDCl_3$) δ 147.89, 141.21, 138.11, 128.25, 127.32, 126.11, 114.70, 112.44, 34.71, 32.43.

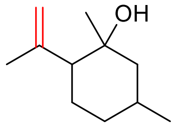

1,5-Dimethyl-2-(prop-1-en-2-yl)cyclohexan-1-ol (71)[43]

^{1}H NMR (400 MHz, $CDCl_3$) δ 4.89 (s, 1H), 4.76 (s, 1H), 1.91 – 1.64 (m, 9H), 1.53 (s, 1H), 1.49 – 1.41 (m, 1H), 1.14 (s, 3H), 1.02 (t, J = 12.8 Hz, 1H), 0.87 (d, J = 6.4 Hz, 3H).

^{13}C NMR (101 MHz, $CDCl_3$) δ 148.30, 111.88, 70.84, 53.10, 48.74, 34.96, 29.91, 27.82, 27.79, 24.75, 22.24.

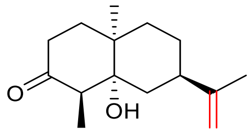

(1R,4aR,7R,8aR)-8a-hydroxy-1,4a-dimethyl-7-(prop-1-en-2-yl)octahydronaphthalen-2(1H)-one (75)[19]

^{1}H NMR (400 MHz, $CDCl_3$) δ 4.76 – 4.63 (m, 2H), 2.88 (q, J = 6.7 Hz, 1H), 2.66 – 2.50 (m, 1H), 2.40 – 2.19 (m, 2H), 2.10 (td, J = 14.1, 5.1 Hz, 1H), 1.89 (td, J = 14.1, 3.9 Hz, 1H), 1.69 (s, 4H), 1.64 – 1.51 (m, 2H), 1.51 – 1.34 (m, 3H), 1.25 (s, 3H), 1.04 (d, J = 6.6 Hz, 3H).

^{13}C NMR (101 MHz, $CDCl_3$) δ 210.69, 149.24, 108.99, 77.91, 51.72, 39.66, 37.62, 37.52, 35.35, 33.36, 31.54, 25.74, 21.66, 20.78, 6.51.

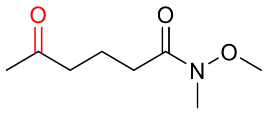

***N*-methoxy-*N*-methyl-5-oxohexanamide (1)**

^{1}H NMR (400 MHz, $CDCl_3$) δ 3.68 (s, 3H), 3.18 (s, 3H), 2.54 (t, *J* = 7.1 Hz, 2H), 2.46 (t, *J* = 7.2 Hz, 2H), 2.14 (s, 3H), 1.91 (p, *J* = 7.2 Hz, 2H).

^{13}C NMR (101 MHz, $CDCl_3$) δ 208.45, 173.92, 61.14, 42.66, 32.08, 30.72, 29.82, 18.56.

HRMS (CI) calcd for $C_8H_{15}NO_3$ $[M+H]^+$: 174.1130; found: 174.1133.

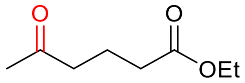

Ethyl 5-oxohexanoate (2)[44]

^{1}H NMR (400 MHz, $CDCl_3$) δ 4.13 (q, *J* = 7.1 Hz, 2H), 2.51 (t, *J* = 7.2 Hz, 2H), 2.33 (t, *J* = 7.3 Hz, 2H), 2.14 (s, 3H), 1.89 (p, *J* = 7.3 Hz, 2H), 1.26 (t, *J* = 7.1 Hz, 3H).

^{13}C NMR (101 MHz, $CDCl_3$) δ 207.97, 173.11, 60.31, 42.44, 33.22, 29.88, 18.85, 14.18.

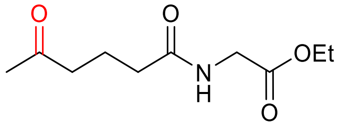

Ethyl (5-oxohexanoyl)glycinate (3)

^{1}H NMR (400 MHz, $CDCl_3$) δ 6.21 (s, 1H), 4.21 (q, *J* = 7.1 Hz, 2H), 4.02 (d, *J* = 5.3 Hz, 2H), 2.54 (t, *J* = 7.0 Hz, 2H), 2.28 (t, *J* = 7.2 Hz, 2H), 2.15 (s, 3H), 1.96 – 1.87 (m, 2H), 1.29 (t, *J* = 7.1 Hz, 3H).

^{13}C NMR (101 MHz, $CDCl_3$) δ 208.59, 172.61, 169.98, 61.45, 42.23, 41.25, 34.89, 29.91, 19.42, 14.07.

HRMS (CI) calcd for $C_{10}H_{17}NO_4$ $[M+H]^+$: 216.1230; found: 216.1231.

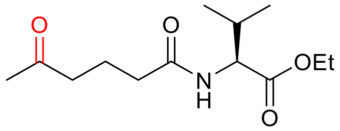

Ethyl (5-oxohexanoyl)-*L*-valinate (4)

^{1}H NMR (400 MHz, $CDCl_3$) δ 6.27 (d, *J* = 8.8 Hz, 1H), 4.53 (dd, *J* = 8.7, 4.9 Hz, 1H), 4.32 – 4.06 (m, 2H), 2.54 (t, *J* = 7.0 Hz, 2H), 2.29 (t, *J* = 7.2 Hz, 2H), 2.15 (s, 3H), 1.91 (p, *J* = 7.1 Hz, 2H), 1.29 (t, *J* = 7.1 Hz, 3H), 1.02 - 0.82 (m, 6H).

^{13}C NMR (101 MHz, $CDCl_3$) δ 208.59, 172.45, 172.01, 61.10, 56.87, 42.22, 35.05, 31.01, 29.81, 19.52, 18.81, 17.65, 14.06.

HRMS (CI) calcd for $C_{13}H_{23}NO_4$ $[M+H]^+$: 258.1700; found: 258.1708.

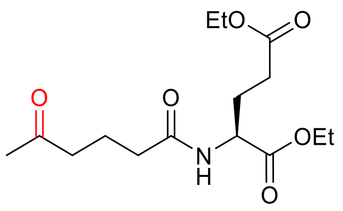

Diethyl (5-oxohexanoyl)-*L*-glutamate (5)

^{1}H NMR (400 MHz, $CDCl_3$) δ 6.42 (d, *J* = 7.6 Hz, 1H), 4.58 (td, *J* = 7.9, 5.2 Hz, 1H), 4.20 (q, *J* = 7.2 Hz, 2H), 4.14 (q, *J* = 7.1 Hz, 2H), 2.53 (t, *J* = 7.0 Hz, 2H), 2.48 – 2.31 (m, 2H), 2.26 (t, *J* = 7.3 Hz, 2H), 2.23 – 2.12 (m, 4H), 2.06 – 1.95 (m, 1H), 1.90 (p, *J* = 7.1 Hz, 2H), 1.36 – 1.19 (m, 6H).

^{13}C NMR (101 MHz, $CDCl_3$) δ 208.52, 172.84, 172.44, 171.84, 61.56, 60.69, 51.64, 42.24, 34.98, 30.30, 29.86, 27.12, 19.38, 14.08, 14.04.

HRMS (CI) calcd for $C_{15}H_{25}NO_6$ $[M+H]^+$: 316.1755; found: 316.1754.

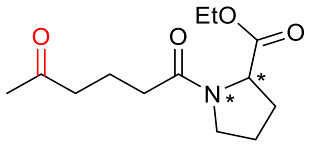

Ethyl (5-oxohexanoyl)-*D*-prolinate (6, dr = 81 : 19**)**

^{1}H NMR (400 MHz, $CDCl_3$) δ 4.54 – 4.31 (m, 1H), 4.27 – 4.09 (m, 2H), 3.70 – 3.27 (m, 2H), 2.63 – 2.48 (m, 2H), 2.44 – 1.82 (m, 11H), 1.33 – 1.23 (m, 3H).

^{13}C NMR (101 MHz, $CDCl_3$) δ 208.78, 208.63, 172.32, 172.17, 171.30, 171.16, 61.51, 60.94, 59.32, 58.65, 46.93, 46.23, 42.44, 33.05, 32.86, 31.37, 29.86, 29.81, 29.16, 24.69, 22.45, 18.71, 18.59, 14.11, 14.08.

HRMS (CI) calcd for $C_{13}H_{22}NO_4$ $[M+H]^+$: 256.1543; found: 256.1552.

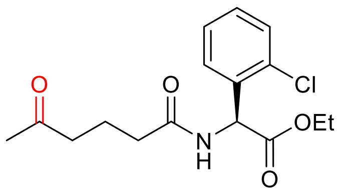

Ethyl (S)-2-(2-chlorophenyl)-2-(5-oxohexanamido)acetate (7)

^{1}H NMR (400 MHz, $CDCl_3$) δ 7.41 - 7.33 (m, 2H), 7.51 - 7.23 (m, 2H), 6.59 (d, *J* = 6.9 Hz, 1H), 5.89 (d, *J* = 7.4 Hz, 1H), 4.34 – 4.07 (m, 2H), 2.50 (t, *J* = 7.0 Hz, 2H), 2.32 - 2.24 (m, 2H), 2.11 (s, 3H), 1.89 (p, *J* = 7.1 Hz, 2H), 1.20 (t, *J* = 7.1 Hz, 3H).

^{13}C NMR (101 MHz, $CDCl_3$) δ 208.43, 170.23, 134.84, 133.49, 130.23, 130.10, 129.65, 127.23, 62.11, 54.65, 42.16, 34.83, 29.92, 19.33, 13.95.

HRMS (CI) calcd for $C_{16}H_{20}ClNO_4$ $[M+H]^+$: 326.1154; found: 326.1161.

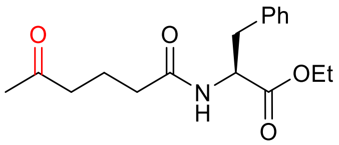

Ethyl (5-oxohexanoyl)-*L*-phenylalaninate (8)

^{1}H NMR (400 MHz, $CDCl_3$) δ 7.33 – 7.20 (m, 3H), 7.14 – 7.09 (m, 2H), 5.96 (d, *J* = 7.6 Hz, 1H), 4.86 (dt, *J* = 7.8, 6.1 Hz, 1H), 4.17 (q, *J* = 7.1 Hz, 2H), 3.20 - 3.01 (m, 2H), 2.44 (t, *J* = 6.8 Hz, 2H), 2.20 (t, *J* = 7.2 Hz, 2H), 2.11 (s, 3H), 1.85 (p, *J* = 7.1 Hz, 2H), 1.25 (t, *J* = 7.1 Hz, 3H).

^{13}C NMR (101 MHz, $CDCl_3$) δ 208.35, 171.88, 171.62, 135.89, 129.23, 128.49, 127.04, 61.48, 52.94, 42.23, 37.93, 35.08, 29.91, 19.39, 14.07.

HRMS (CI) calcd for $C_{17}H_{23}NO_4$ $[M+H]^+$: 306.1700; found: 306.1702.

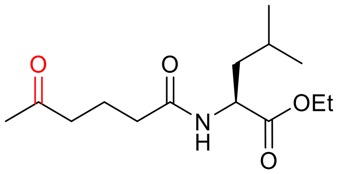

Ethyl (5-oxohexanoyl)-*L*-leucinate (9)

^{1}H NMR (400 MHz, $CDCl_3$) δ 5.99 (d, *J* = 8.4 Hz, 1H), 4.60 (td, *J* = 8.6, 4.9 Hz, 1H), 4.18 (q, *J* = 7.1 Hz, 2H), 2.53 (t, *J* = 7.0 Hz, 2H), 2.26 (t, *J* = 7.2 Hz, 2H), 2.15 (s, 3H), 1.91 (p, *J* = 7.1 Hz, 2H), 1.72 – 1.58 (m, 2H), 1.58 – 1.47 (m, 1H), 1.28 (t, *J* = 7.1 Hz, 3H), 0.99 – 0.89 (m, 6H).

^{13}C NMR (101 MHz, $CDCl_3$) δ 208.52, 173.10, 172.13, 61.24, 50.64, 42.24, 41.57, 35.06, 29.91, 24.85, 22.74, 21.90, 19.48, 14.09.

HRMS (CI) calcd for $C_{14}H_{25}NO_4$ $[M+H]^+$: 272.1856; found: 272.1857.

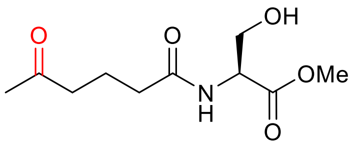

Methyl (5-oxohexanoyl)-L-serinate (10)

^{1}H NMR (400 MHz, $CDCl_3$) δ 6.72 (d, *J* = 7.2 Hz, 1H), 4.64 (dt, *J* = 7.3, 3.5 Hz, 1H), 4.04 - 3.84 (m, 2H), 3.78 (s, 3H), 3.46 – 3.24 (m, 1H), 2.55 (t, *J* = 6.6 Hz, 2H), 2.30 (t, *J* = 7.2 Hz, 2H), 2.16 (s, 3H), 1.93 (p, *J* = 7.0 Hz, 2H).

^{13}C NMR (101 MHz, $CDCl_3$) δ 209.14, 172.89, 170.98, 62.94, 54.64, 52.63, 42.32, 35.08, 29.91, 19.47.

HRMS (CI) calcd for $C_{10}H_{17}NO_5$ $[M+H]^+$: 232.1179; found: 232.1186.

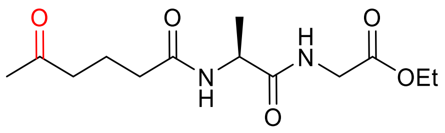

Ethyl (5-oxohexanoyl)-*L*-alanylglycinate (11)

^{1}H NMR (400 MHz, $CDCl_3$) δ 7.07 (t, *J* = 5.5 Hz, 1H), 6.48 (d, *J* = 7.5 Hz, 1H), 4.65 – 4.50 (m, 1H), 4.20 (q, *J* = 7.1 Hz, 2H), 4.01 (dd, *J* = 5.5, 2.4 Hz, 2H), 2.52 (t, *J* = 7.0 Hz, 2H), 2.25 (td, *J* = 7.2, 2.3 Hz, 2H), 2.14 (s, 3H), 1.90 (p, *J* = 7.1 Hz, 2H), 1.39 (d, *J* = 7.0 Hz, 3H), 1.28 (t, *J* = 7.1 Hz, 3H).

^{13}C NMR (101 MHz, $CDCl_3$) δ 208.55, 172.63, 172.46, 169.57, 61.40, 48.50, 42.34, 41.25, 35.05, 29.88, 19.39, 18.07, 14.07.

HRMS (CI) calcd for $C_{13}H_{22}N_2O_5$ $[M+H]^+$: 287.1601; found: 287.1609.

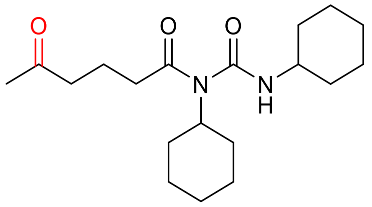

***N*-cyclohexyl-*N*-(cyclohexylcarbamoyl)-5-oxohexanamide (12)**

^{1}H NMR (400 MHz, $CDCl_3$) δ 7.08 (s, 1H), 4.01 – 3.83 (m, 1H), 3.77 – 3.60 (m, 1H), 2.54 (t, *J* = 6.8 Hz, 2H), 2.42 (t, *J* = 7.0 Hz, 2H), 2.15 (s, 3H), 2.02 – 1.89 (m, 4H), 1.85 – 1.68 (m, 8H), 1.67 – 1.56 (m, 2H), 1.42 – 1.12 (m, 8H).

^{13}C NMR (101 MHz, $CDCl_3$) δ 209.10, 172.15, 153.89, 55.33, 49.82, 42.31, 34.27, 32.58, 30.81, 29.87, 26.13, 25.41, 25.28, 24.65, 19.42.

HRMS (ESI) calcd for $C_{19}H_{32}N_2O_3$ $[M+Na]^+$: 359.2305; found: 359.2305.

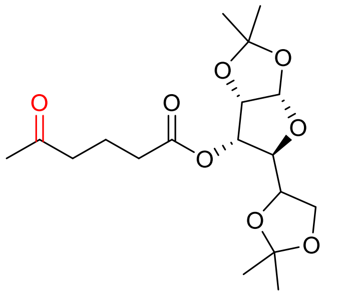

(3a*S*,5*S*,6*S*,6a*S*)-5-(2,2-dimethyl-1,3-dioxolan-4-yl)-2,2-dimethyltetrahydrofuro[2,3-*d*][1,3]dioxol-6-yl 5-oxohexanoate (13)

^{1}H NMR (400 MHz, $CDCl_3$) δ 5.87 (d, *J* = 3.7 Hz, 1H), 5.27 (d, *J* = 2.1 Hz, 1H), 4.48 (d, *J* = 3.7 Hz, 1H), 4.22 – 4.17 (m, 2H), 4.11 – 3.98 (m, 2H), 2.53 (t, *J* = 7.1 Hz, 2H), 2.39 (td, *J* = 7.3, 1.9 Hz, 2H),

2.14 (s, 3H), 1.91 (p, J = 7.2 Hz, 2H), 1.52 (s, 3H), 1.41 (s, 3H), 1.36 – 1.27 (m, 6H).

^{13}C NMR (101 MHz, CDCl$_3$) δ 207.72, 171.72, 112.27, 109.33, 105.05, 83.36, 79.82, 76.00, 72.43, 67.31, 42.19, 33.13, 29.95, 26.86, 26.71, 26.19, 25.27, 18.72.

HRMS (ESI) calcd for $C_{18}H_{28}O_8$ [M+Na]$^+$: 395.1676; found: 395.1676.

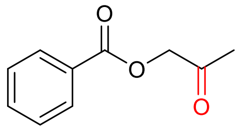

2-Oxopropyl benzoate (14)[45]

^{1}H NMR (400 MHz, CDCl$_3$) δ 8.15 – 8.03 (m, 2H), 7.59 (t, J = 7.4 Hz, 1H), 7.46 (t, J = 7.7 Hz, 2H), 4.88 (s, 2H), 2.23 (s, 3H).

^{13}C NMR (101 MHz, CDCl$_3$) δ 201.75, 165.78, 133.40, 129.81, 129.11, 128.43, 68.67, 26.14.

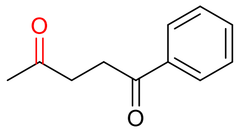

1-Phenylpentane-1,4-dione (15)

^{1}H NMR (400 MHz, CDCl$_3$) δ 8.03 – 7.92 (m, 2H), 7.56 (t, J = 7.4 Hz, 1H), 7.46 (t, J = 7.6 Hz, 2H), 3.28 (t, J = 6.4 Hz, 2H), 2.88 (t, J = 6.3 Hz, 2H), 2.26 (s, 3H).

^{13}C NMR (101 MHz, CDCl$_3$) δ 207.22, 198.42, 136.56, 133.08, 128.50, 127.96, 36.98, 32.35, 30.03.

HRMS (CI) calcd for $C_{10}H_{12}O_2$ [M+H]$^+$: 177.0910; found: 177.0918.

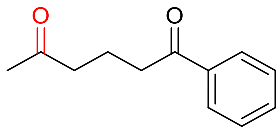

1-Phenylhexane-1,5-dione (16)

^{1}H NMR (400 MHz, CDCl$_3$) δ 7.96 (dd, J = 8.4, 1.3 Hz, 2H), 7.59 – 7.52 (m, 1H), 7.49 – 7.41 (m, 2H), 3.02 (t, J = 7.0 Hz, 2H), 2.57 (t, J = 7.0 Hz, 2H), 2.15 (s, 3H), 2.10 – 1.93 (m, 2H).

^{13}C NMR (101 MHz, CDCl$_3$) δ 208.43, 199.68, 136.75, 133.03, 128.55, 127.98, 42.54, 37.34, 29.90, 18.15.

HRMS (CI) calcd for $C_{12}H_{14}O_2$ [M+H]$^+$: 191.1067; found: 191.1072.

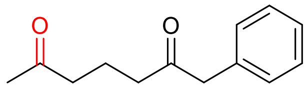

1-Phenylheptane-2,6-dione (17)

^{1}H NMR (400 MHz, $CDCl_3$) δ 7.36 – 7.29 (m, 2H), 7.29 – 7.23 (m, 1H), 7.22 – 7.16 (m, 2H), 3.67 (s, 2H), 2.49 (t, *J* = 7.0 Hz, 2H), 2.39 (t, *J* = 7.1 Hz, 2H), 2.07 (s, 3H), 1.81 (p, *J* = 7.0 Hz, 2H).

^{13}C NMR (101 MHz, $CDCl_3$) δ 208.28, 207.79, 134.06, 129.33, 128.70, 126.99, 50.12, 42.27, 40.60, 29.81, 17.56.

HRMS (CI) calcd for $C_{13}H_{16}O_2$ $[M+H]^+$: 205.1223; found: 205.1226.

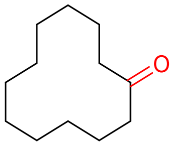

Cyclododecanone (18)[46]

^{1}H NMR (400 MHz, $CDCl_3$) δ 2.56 – 2.38 (m, 4H), 1.87 – 1.58 (m, 4H), 1.44 – 1.09 (m, 14H).

^{13}C NMR (101 MHz, $CDCl_3$) δ 212.81, 40.33, 24.71, 24.57, 24.19, 22.52, 22.31.

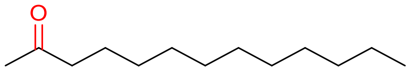

Tridecan-2-one (19)[47]

^{1}H NMR (400 MHz, $CDCl_3$) δ 2.41 (t, *J* = 7.5 Hz, 2H), 2.13 (s, 3H), 1.63 – 1.48 (m, 2H), 1.41 – 1.15 (s, 16H), 0.88 (t, *J* = 6.7 Hz, 3H).

^{13}C NMR (101 MHz, $CDCl_3$) δ 209.25, 43.77, 31.86, 29.77, 29.56, 29.43, 29.36, 29.29, 29.15, 23.84, 22.64, 14.05.

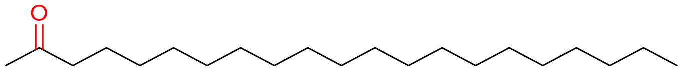

Henicosan-2-one (20)

^{1}H NMR (400 MHz, $CDCl_3$) δ 2.41 (t, *J* = 7.5 Hz, 2H), 2.13 (s, 3H), 1.64 - 1.51 (m, 2H), 1.37 – 1.19 (m, 32H), 0.88 (t, *J* = 6.8 Hz, 3H).

^{13}C NMR (101 MHz, $CDCl_3$) δ 209.36, 43.83, 31.92, 29.83, 29.69, 29.65, 29.60, 29.47, 29.36, 29.19, 23.88, 22.69, 14.11.

HRMS (CI) calcd for $C_{21}H_{42}O$ $[M+H]^+$: 311.3308; found: 311.3308.

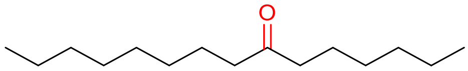

Pentadecan-7-one (21)[48]

^{1}H NMR (400 MHz, $CDCl_3$) δ 2.38 (t, *J* = 7.5 Hz, 4H), 1.62 – 1.47 (m, 4H), 1.38 – 1.17 (m, 16H), 0.93 – 0.81 (m, 6H).

^{13}C NMR (101 MHz, $CDCl_3$) δ 210.70, 41.80, 30.81, 30.60, 28.37, 28.27, 28.13,, 27.93, 22.89, 22.85, 21.63, 21.49, 13.07, 13.01.

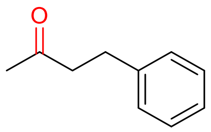

4-Phenylbutan-2-one (23)[49]

^{1}H NMR (400 MHz, $CDCl_3$) δ 7.27 (t, J = 7.5 Hz, 2H), 7.22 - 7.14 (m, 3H), 2.89 (t, J = 7.6 Hz, 2H), 2.75 (t, J = 7.6 Hz, 2H), 2.13 (s, 3H).

^{13}C NMR (101 MHz, $CDCl_3$) δ 207.83, 140.92, 128.43, 128.22, 126.04, 45.10, 30.00, 29.68.

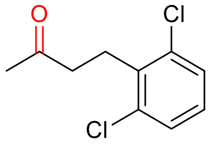

4-(2,6-Dichlorophenyl)butan-2-one (24)[50]

^{1}H NMR (400 MHz, $CDCl_3$) δ 7.33 – 7.20 (m, 2H), 7.08 (t, J = 8.0 Hz, 1H), 3.19 (t, J = 8.2 Hz, 2H), 2.69 (t, J = 8.4 Hz, 2H), 2.20 (s, 3H).

^{13}C NMR (101 MHz, $CDCl_3$) δ 207.17, 136.74, 135.19, 128.15, 127.86, 41.37, 29.70, 25.53.

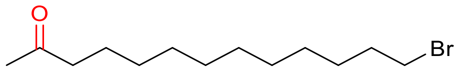

13-Bromotridecan-2-one (25)[51]

^{1}H NMR (400 MHz, $CDCl_3$) δ 3.40 (t, J = 6.9 Hz, 2H), 2.42 (t, J = 7.5 Hz, 2H), 2.13 (s, 3H), 1.91 – 1.80 (m, 2H), 1.62 – 1.50 (m, 2H), 1.49 – 1.37 (m, 7.0 Hz, 2H), 1.34 - 1.22 (m, 12H).

^{13}C NMR (101 MHz, $CDCl_3$) δ 209.20, 43.71, 33.95, 32.75, 29.77, 29.36, 29.31, 29.28, 29.07, 28.66, 28.07, 23.76.

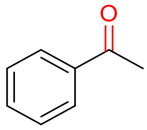

Acetophenone (26)[7]

^{1}H NMR (400 MHz, $CDCl_3$) δ 8.02 – 7.89 (m, 2H), 7.61 – 7.52 (m, 1H), 7.47 (t, J = 7.6 Hz, 2H), 2.61 (s, 3H).

^{13}C NMR (101 MHz, $CDCl_3$) δ 198.12, 137.10, 133.07, 128.53, 128.27, 26.59.

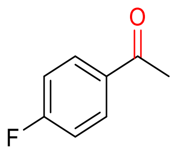

1-(4-Fluorophenyl)ethan-1-one (27)[7]

^{1}H NMR (400 MHz, $CDCl_3$) δ 8.02 - 7.95 (m, 2H), 7.13 (t, *J* = 8.6 Hz, 2H), 2.59 (s, 3H).

^{13}C NMR (101 MHz, $CDCl_3$) δ 196.43, 165.73 (d, *J* = 255.53 Hz), 133.56 (d, *J* = 3.0 Hz), 130.90 (d, *J* = 9.4 Hz), 115.61(d, *J* = 22.2 Hz), 26.50.

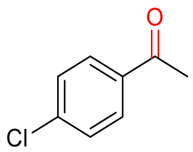

1-(4-Chlorophenyl)ethan-1-one (28)[7]

^{1}H NMR (400 MHz, $CDCl_3$) δ 7.90 (d, *J* = 8.7 Hz, 2H), 7.44 (d, *J* = 8.7 Hz, 2H), 2.59 (s, 3H).

^{13}C NMR (101 MHz, $CDCl_3$) δ 196.77, 139.53, 135.40, 129.69, 128.85, 26.52.

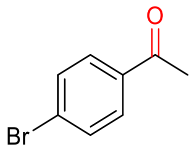

1-(4-Bromophenyl)ethan-1-one (29)[7]

^{1}H NMR (400 MHz, $CDCl_3$) δ 7.82 (d, *J* = 8.6 Hz, 2H), 7.60 (d, *J* = 8.6 Hz, 2H), 2.58 (s, 3H).

^{13}C NMR (101 MHz, $CDCl_3$) δ 196.93, 135.78, 131.84, 129.79, 128.25, 26.49.

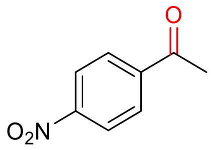

1-(4-Nitrophenyl)ethan-1-one (30)[52]

^{1}H NMR (400 MHz, $CDCl_3$) δ 8.32 (d, *J* = 8.8 Hz, 2H), 8.12 (d, *J* = 8.8 Hz, 2H), 2.69 (s, 3H).

^{13}C NMR (101 MHz, $CDCl_3$) δ 196.25, 150.32, 141.34, 129.26, 123.81, 26.94.

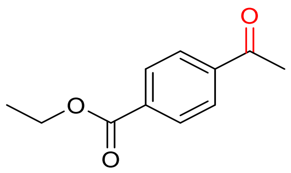

Ethyl 4-acetylbenzoate (31)[53]

^{1}H NMR (400 MHz, $CDCl_3$) δ 8.12 (d, *J* = 8.4 Hz, 2H), 8.00 (d, *J* = 8.4 Hz, 2H), 4.41 (q, *J* = 7.1 Hz, 2H), 2.64 (s, 3H), 1.42 (t, *J* = 7.1 Hz, 3H).

^{13}C NMR (101 MHz, $CDCl_3$) δ 197.35, 165.54, 140.00, 134.10, 129.61, 128.01, 61.29, 26.72, 14.14.

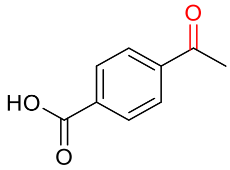

4-Acetylbenzoic acid (32)[54]

^{1}H NMR (400 MHz, DMSO) δ 13.28 (s, 1H), 8.04 (s, 4H), 2.61 (s, 3H).

^{13}C NMR (101 MHz, DMSO) δ 197.73, 166.67, 139.85, 134.52, 129.57, 128.33, 27.01.

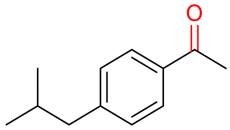

1-(4-Isobutylphenyl)ethan-1-one (33)[55]

^{1}H NMR (400 MHz, $CDCl_3$) δ 7.88 (d, J = 8.2 Hz, 2H), 7.23 (d, J = 8.3 Hz, 2H), 2.58 (s, 3H), 2.53 (d, J = 7.2 Hz, 2H), 1.97 – 1.80 (m, 1H), 0.91 (d, J = 6.7 Hz, 6H).

^{13}C NMR (101 MHz, $CDCl_3$) δ 197.84, 147.54, 134.92, 129.23, 128.26, 45.34, 30.07, 26.49, 22.29.

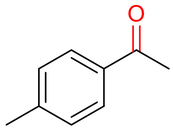

1-(*p*-Tolyl)ethan-1-one (34)[7]

^{1}H NMR (400 MHz, $CDCl_3$) δ 7.78 (d, J = 8.2 Hz, 2H), 7.18 (d, J = 8.0 Hz, 2H), 2.50 (s, 3H), 2.33 (s, 3H).

^{13}C NMR (101 MHz, $CDCl_3$) δ 197.80, 143.82, 134.66, 129.18, 128.39, 26.47, 21.58.

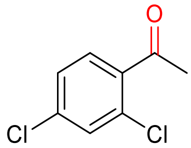

1-(2,4-Dichlorophenyl)ethan-1-one (35)[56]

^{1}H NMR (400 MHz, $CDCl_3$) δ 7.54 (d, J = 8.4 Hz, 1H), 7.45 (d, J = 2.0 Hz, 1H), 7.32 (dd, J = 8.4, 2.0 Hz, 1H), 2.64 (s, 3H).

^{13}C NMR (101 MHz, $CDCl_3$) δ 198.91, 137.69, 137.16, 132.51, 130.68, 130.52, 127.33, 30.67.

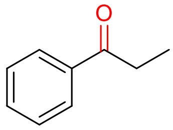

Propiophenone (36)[52]

^{1}H NMR (400 MHz, $CDCl_3$) δ 7.97 (dd, *J* = 8.3, 1.3 Hz, 2H), 7.55 (t, *J* = 7.4 Hz, 1H), 7.46 (t, *J* = 7.5 Hz, 2H), 3.01 (q, *J* = 7.3 Hz, 2H), 1.23 (t, *J* = 7.2 Hz, 3H).

^{13}C NMR (101 MHz, $CDCl_3$) δ 200.81, 136.90, 132.84, 128.52, 127.95, 31.76, 8.22.

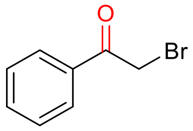

2-Bromo-1-phenylethan-1-one (37)[7]

^{1}H NMR (400 MHz, $CDCl_3$) δ 8.03 – 7.93 (m, 2H), 7.61 (t, *J* = 7.4 Hz, 1H), 7.49 (t, *J* = 7.7 Hz, 2H), 4.46 (s, 2H).

^{13}C NMR (101 MHz, $CDCl_3$) δ 191.19, 133.88, 128.84, 128.79, 30.93.

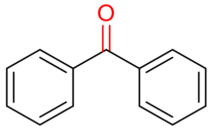

Benzophenone (38)[7]

^{1}H NMR (400 MHz, $CDCl_3$) δ 7.87 – 7.72 (m, 4H), 7.62 – 7.51 (m, 2H), 7.47 (t, *J* = 7.5 Hz, 4H).

^{13}C NMR (101 MHz, $CDCl_3$) δ 196.62, 137.50, 132.32, 129.95, 128.18.

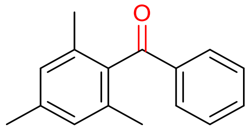

Mesityl(phenyl)methanone (39)[57]

^{1}H NMR (400 MHz, $CDCl_3$) δ 7.80 (dd, *J* = 8.4, 1.4 Hz, 2H), 7.62 – 7.50 (m, 1H), 7.44 (t, *J* = 7.7 Hz, 2H), 6.89 (s, 2H), 2.33 (s, 3H), 2.08 (s, 6H).

^{13}C NMR (101 MHz, $CDCl_3$) δ 200.73, 138.46, 137.29, 136.85, 134.16, 133.50, 129.37, 128.75, 28.29, 21.13, 19.33.

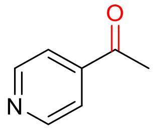

1-(Pyridin-4-yl)ethan-1-one (40)[7]

^{1}H NMR (400 MHz, $CDCl_3$) δ 8.82 (d, *J* = 5.9 Hz, 2H), 7.73 (dd, *J* = 4.6, 1.3 Hz, 2H), 2.64 (s, 3H).

^{13}C NMR (101 MHz, $CDCl_3$) δ 197.22, 150.89, 142.65, 121.14, 26.57.

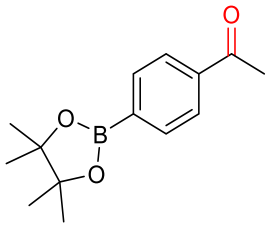

1-(4-(4,4,5,5-Tetramethyl-1,3,2-dioxaborolan-2-yl)phenyl)ethan-1-one (41)[58]

^{1}H NMR (400 MHz, $CDCl_3$) δ 8.01 – 7.85 (m, 4H), 2.62 (s, 3H), 1.36 (s, 12H).

^{13}C NMR (101 MHz, $CDCl_3$) δ 198.42, 138.97, 134.89, 130.68, 127.26, 84.19, 26.75, 24.86.

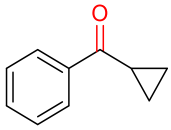

Cyclopropyl(phenyl)methanone (42)[7]

^{1}H NMR (400 MHz, $CDCl_3$) δ 8.01 (dd, J = 8.3, 1.3 Hz, 2H), 7.60 – 7.51 (m, 1H), 7.46 (t, J = 7.5 Hz, 2H), 2.67 (tt, J = 7.8, 4.6 Hz, 1H), 1.31 – 1.18 (m, 2H), 1.07 – 0.97 (m, 2H).

^{13}C NMR (101 MHz, $CDCl_3$) δ 200.54, 137.91, 132.63, 128.40, 127.91, 17.05, 11.56.

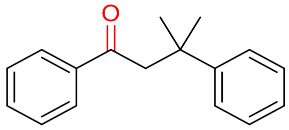

3-Methyl-1,3-diphenylbutan-1-one (43)[59]

^{1}H NMR (400 MHz, $CDCl_3$) δ 7.81 (d, J = 7.0 Hz, 2H), 7.48 (t, J = 7.4 Hz, 1H), 7.36 (t, J = 7.8 Hz, 4H), 7.27 (t, J = 8.0 Hz, 2H), 7.14 (t, J = 7.3 Hz, 1H), 3.30 (s, 2H), 1.50 (s, 6H).

^{13}C NMR (101 MHz, $CDCl_3$) δ 198.93, 148.82, 138.10, 132.59, 128.32, 128.32, 128.11, 125.72, 125.39, 50.81, 37.47, 29.07.

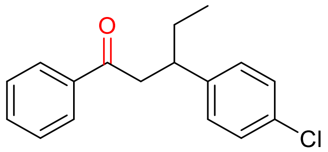

3-(4-Chlorophenyl)-1-phenylpentan-1-one (44)[60]

^{1}H NMR (400 MHz, $CDCl_3$) δ 7.91 - 7.84 (m, 2H), 7.59 – 7.47 (m, 1H), 7.42 (t, J = 7.6 Hz, 2H), 7.24 (d, J = 8.5 Hz, 2H), 7.15 (d, J = 8.5 Hz, 2H), 3.29 – 3.16 (m, 3H), 1.83 – 1.69 (m, 1H), 1.67 – 1.51 (m, 1H), 0.80 (t, J = 7.3 Hz, 3H).

^{13}C NMR (101 MHz, $CDCl_3$) δ 198.74, 143.10, 137.07, 132.98, 131.80, 128.96, 128.52, 128.47, 27.96, 45.34, 42.32, 29.21, 11.97.

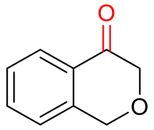

Isochroman-4-one (45)[61]

^{1}H NMR (400 MHz, $CDCl_3$) δ 8.02 (d, *J* = 7.8 Hz, 1H), 7.55 (t, *J* = 7.5 Hz, 1H), 7.40 (t, *J* = 7.6 Hz, 1H), 7.20 (d, *J* = 7.7 Hz, 1H), 4.87 (s, 2H), 4.35 (s, 2H).

^{13}C NMR (101 MHz, $CDCl_3$) δ 193.93, 141.65, 134.12, 129.47, 127.79, 126.26, 124.42, 73.44, 67.80.

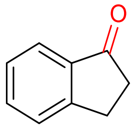

2,3-Dihydro-1*H*-inden-1-one (46)[54]

^{1}H NMR (400 MHz, $CDCl_3$) δ 7.76 (d, *J* = 7.7 Hz, 1H), 7.58 (td, *J* = 7.4, 1.3 Hz, 1H), 7.48 (d, *J* = 7.7 Hz, 1H), 7.37 (t, *J* = 7.5 Hz, 1H), 3.20 – 3.10 (m, 2H), 2.75 – 2.63 (m, 2H).

^{13}C NMR (101 MHz, $CDCl_3$) δ 206.97, 155.08, 137.01, 134.52, 127.20, 126.63, 123.62, 36.15, 25.74.

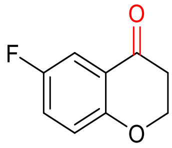

6-Fluorochroman-4-one (47)[62]

^{1}H NMR (400 MHz, $CDCl_3$) δ 7.55 (dd, *J* = 8.3, 3.2 Hz, 1H), 7.24 – 7.15 (m, 1H), 6.96 (dd, *J* = 9.1, 4.2 Hz, 1H), 4.53 (t, *J* = 6.4 Hz, 2H), 2.81 (t, *J* = 6.4 Hz, 2H).

^{13}C NMR (101 MHz, $CDCl_3$) δ 190.99 (d, *J* = 2.0 Hz), 158.10 (d, *J* = 2.0 Hz), 157.18 (d, *J* = 242.4 Hz), 123.49 (d, *J* = 24.2 Hz), 121.70 (d, *J* = 7.1 Hz), 119.55 (d, *J* = 7.3 Hz), 112.07 (d, *J* = 23.2 Hz), 67.15, 37.49.

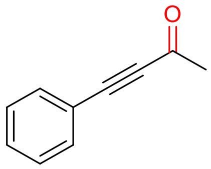

4-Phenylbut-3-yn-2-one (48)[63]

^{1}H NMR (400 MHz, $CDCl_3$) δ 7.62 – 7.54 (m, 2H), 7.48 – 7.43 (m, 1H), 7.38 (t, *J* = 7.4 Hz, 2H), 2.45 (s, 3H).

^{13}C NMR (101 MHz, $CDCl_3$) δ 184.51, 132.95, 130.65, 128.54, 119.80, 90.24, 88.18, 32.65.

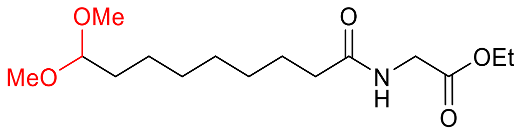

Ethyl (9,9-dimethoxynonanoyl)glycinate (50)

^{1}H NMR (400 MHz, $CDCl_3$) δ 6.00 (s, 1H), 4.35 (t, *J* = 5.7 Hz, 1H), 4.22 (q, *J* = 7.2 Hz, 2H), 4.03 (d, *J* = 5.1 Hz, 2H), 3.31 (s, 6H), 2.27 – 2.19 (m, 2H), 1.69 – 1.54 (m, 4H), 1.42 – 1.22 (m, 11H).

^{13}C NMR (101 MHz, $CDCl_3$) δ 173.16, 170.10, 104.51, 61.46, 52.59, 41.29, 36.35, 32.42, 29.23, 29.20, 29.09, 25.48, 24.49, 14.10.

HRMS (ESI) calcd for $C_{15}H_{29}NO_5$ $[M+Na]^+$: 326.1938; found: 326.1937.

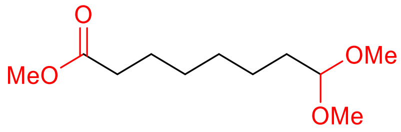

Methyl 8,8-dimethoxyoctanoate (52)[64]

^{1}H NMR (400 MHz, $CDCl_3$) δ 4.34 (t, *J* = 5.7 Hz, 1H), 3.66 (s, 3H), 3.30 (s, 6H), 2.29 (t, *J* = 7.5 Hz, 2H), 1.62 – 1.53 (m, 4H), 1.35 – 1.29 (m, 6H).

^{13}C NMR (101 MHz, $CDCl_3$) δ 174.22, 104.49, 52.61, 51.43, 34.03, 32.40, 29.07, 29.03, 24.82, 24.39.

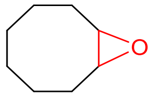

9-Oxabicyclo[6.1.0]nonane (53)[65]

^{1}H NMR (400 MHz, $CDCl_3$) δ 2.99 – 2.79 (m, 2H), 2.20 – 2.08 (m, 2H), 1.63 – 1.27 (m, 10H).

^{13}C NMR (101 MHz, $CDCl_3$) δ 55.66 , 26.55 , 26.28 , 25.59 .

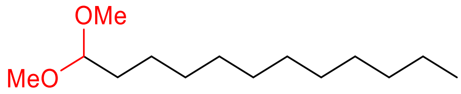

1,1-Dimethoxydodecane (55)[66]

^{1}H NMR (400 MHz, $CDCl_3$) δ 4.36 (t, *J* = 5.8 Hz, 1H), 3.31 (s, 6H), 1.63 – 1.54 (m, 2H), 1.42 – 1.15 (m, 18H), 0.88 (t, *J* = 6.7 Hz, 3H).

^{13}C NMR (101 MHz, $CDCl_3$) δ 104.58, 52.57, 32.49, 31.91, 29.64, 29.62, 29.59, 29.56, 29.48, 29.34, 24.60, 22.68, 14.11.

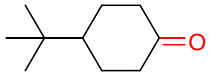

4-(*tert*-Butyl)cyclohexan-1-one (60)[67]

^{1}H NMR (400 MHz, $CDCl_3$) δ 2.48 – 2.19 (m, 4H), 2.13 – 2.00 (m, 2H), 1.56 – 1.32 (m, 3H), 0.96 – 0.87 (m, 9H).

^{13}C NMR (101 MHz, $CDCl_3$) δ 212.57, 46.69, 41.29, 32.44, 27.58.

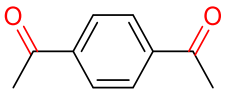

1,4-Diacetylbenzene (66)[7]

^{1}H NMR (400 MHz, $CDCl_3$) δ 8.04 (s, 4H), 2.65 (s, 6H).

^{13}C NMR (101 MHz, $CDCl_3$) δ 197.46, 140.16, 128.45, 26.86.

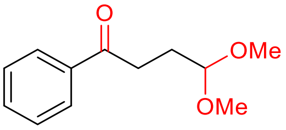

4,4-Dimethoxy-1-phenylbutan-1-one (68)[68]

^{1}H NMR (400 MHz, $CDCl_3$) δ 8.04 – 7.93 (m, 2H), 7.56 (t, J = 7.4 Hz, 1H), 7.46 (t, J = 7.6 Hz, 2H), 4.48 (t, J = 5.5 Hz, 1H), 3.35 (s, 6H), 3.06 (t, J = 7.3 Hz, 2H), 2.14 - 2.01 (m, 2H).

^{13}C NMR (101 MHz, $CDCl_3$) δ 199.49, 136.87, 132.96, 128.52, 127.97, 103.90, 53.25, 33.23, 26.92.

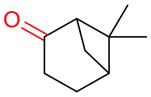

6,6-Dimethylbicyclo[3.1.1]heptan-2-one (70)[59]

^{1}H NMR (400 MHz, $CDCl_3$) δ 2.66 – 2.47 (m, 3H), 2.40 – 2.29 (m, 1H), 2.27 -2.20 (m, 1H), 2.11 – 2.01 (m, 1H), 2.00 – 1.89 (m, 1H), 1.58 (d, J = 10.2 Hz, 1H), 1.33 (s, 3H), 0.86 (s, 3H).

^{13}C NMR (101 MHz, $CDCl_3$) δ 214.98, 57.96, 41.19, 40.38, 32.78, 25.88, 25.26, 22.11, 21.39.

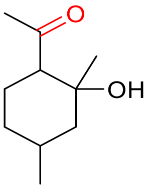

1-(2-Hydroxy-2,4-dimethylcyclohexyl)ethan-1-one (72)

^{1}H NMR (400 MHz, $CDCl_3$) δ 3.96 (d, J = 2.5 Hz, 1H), 2.46 – 2.36 (m, 1H), 2.22 (s, 3H), 1.96 – 1.82 (m, 1H), 1.82 – 1.63 (m, 4H), 1.18 (s, 3H), 0.94 – 0.79 (m, 5H).

^{13}C NMR (101 MHz, $CDCl_3$) δ 216.28, 70.19, 56.90, 47.36, 34.07, 31.13, 29.32, 27.07, 25.68, 21.93.

HRMS (CI) calcd for $C_{10}H_{18}O_2$ $[M+H]^+$: 171.1380; found: 171.1385.

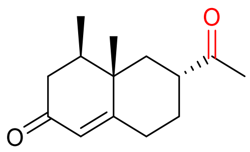

(4R,4aS,6R)-6-acetyl-4,4a-dimethyl-4,4a,5,6,7,8-hexahydronaphthalen-2(3H)-one (74)[69]

^{1}H NMR (400 MHz, $CDCl_3$) δ 5.75 (s, 1H), 2.80 – 2.66 (m, 1H), 2.56 – 2.34 (m, 2H), 2.29 – 1.94 (m, 8H), 1.49 – 1.35 (m, 1H), 1.23 (t, *J* = 13.2 Hz, 1H), 1.09 (s, 3H), 0.96 (d, *J* = 6.8 Hz, 3H).

^{13}C NMR (101 MHz, $CDCl_3$) δ 210.42, 199.20, 168.57, 125.09, 46.68, 41.96, 40.12, 39.86, 38.81, 31.96, 28.40, 28.09, 16.63, 14.81.

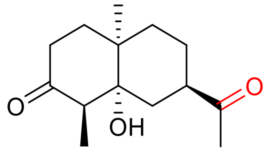

(*1R,4aR,7R,8aR*)-7-acetyl-8a-hydroxy-1,4a-dimethyloctahydronaphthalen-2(1H)-one (76)

^{1}H NMR (400 MHz, $CDCl_3$) δ 2.92 – 2.80 (m, 1H), 2.74 (tt, *J* = 12.7, 3.9 Hz, 1H), 2.56 (td, *J* = 14.2, 7.1 Hz, 1H), 2.33 (ddd, *J* = 14.4, 5.1, 1.8 Hz, 1H), 2.17 – 2.00 (m, 4H), 1.92 (td, *J* = 13.6, 4.2 Hz, 1H), 1.84 (s, 1H), 1.78 – 1.67 (m, 2H), 1.53 (td, *J* = 13.4, 4.1 Hz, 1H), 1.48 – 1.37 (m, 2H), 1.24 (s, 3H), 1.22 – 1.12 (m, 1H), 1.05 (d, *J* = 6.7 Hz, 3H).

^{13}C NMR (101 MHz, $CDCl_3$) δ 211.41, 209.83, 77.37, 51.66, 46.16, 37.46, 37.43, 34.55, 31.15, 29.98, 28.16, 22.75, 21.51, 6.47.

HRMS (ESI) calcd for $C_{14}H_{22}O_3$ $[M+Na]^+$: 261.1461; found: 261.1463.

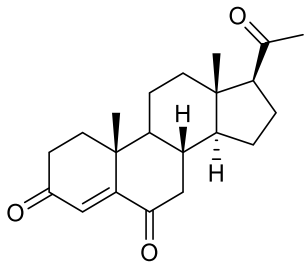

(*8S,10R,13S,14S,17S*)-17-acetyl-10,13-dimethyl-1,7,8,9,10,11,12,13,14,15,16,17-dodecahydro-3H-cyclopenta[a]phenanthrene-3,6 (2H)-dione (78)[70]

^{1}H NMR (400 MHz, $CDCl_3$) δ 6.19 (s, 1H), 2.70 (dd, *J* = 15.9, 4.0 Hz, 1H), 2.62 – 2.40 (m, 3H), 2.17 – 1.87 (m, 8H), 1.81 – 1.67 (m, 3H), 1.55 – 1.22 (m, 6H), 1.17 (s, 3H), 0.69 (s, 3H).

^{13}C NMR (101 MHz, $CDCl_3$) δ 208.79, 201.58, 199.25, 160.44, 125.71, 63.13, 56.52, 50.75, 46.49, 43.86, 39.67, 38.15, 35.54, 34.01, 33.92, 31.44, 24.13, 22.83, 20.84, 17.53, 13.24.

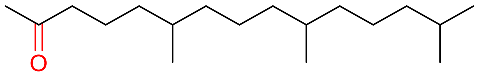

6,10,14-Trimethylpentadecan-2-one (80)[71]

^{1}H NMR (400 MHz, $CDCl_3$) δ 2.39 (t, *J* = 7.5 Hz, 2H), 2.12 (s, 3H), 1.65 – 1.43 (m, 3H), 1.38 – 1.04 (m, 16H), 0.87 – 0.81 (m, 12H).

^{13}C NMR (101 MHz, $CDCl_3$) δ 209.32, 44.13, 39.35, 37.39, 37.37,37.34, 37.27, 37.21, 37.17, 36.57, 36.48, 32.77, 32.75, 32.66, 32.64, 29.83, 27.96, 24.79, 24.78, 24.41, 24.40, 22.70, 22.61, 21.43, 21.42, 19.72, 19.66, 19.56, 19.50.

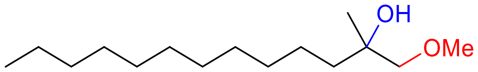

1-Methoxy-2-methyltridecan-2-ol (19-1)

^{1}H NMR (400 MHz, $CDCl_3$) δ 3.41 (s, 3H), 3.24 (dd, J = 22.2, 8.9 Hz, 2H), 1.86 (br, 1H), 1.53 – 1.45 (m, 2H), 1.38 – 1.24 (m, 18H), 1.17 (s, 3H), 0.90 (t, J = 6.6 Hz, 3H).

^{13}C NMR (101 MHz, $CDCl_3$) δ 76.93, 72.10, 59.30, 39.13, 31.91, 30.24, 29.66, 29.62, 29.61, 29.34, 23.74, 23.63, 22.68, 14.11.

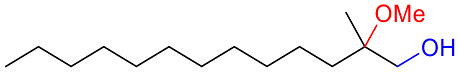

2-Methoxy-2-methyltridecan-1-ol (19-2)

^{1}H NMR (400 MHz, $CDCl_3$) δ 3.48 (d, J = 11.2 Hz, 1H), 3.40 (d, J = 11.3 Hz, 1H), 3.19 (s, 3H), 1.88 (br, 1H), 1.50 – 1.42 (m, 2H), 1.31 – 1.22 (m, 18H), 1.11 (s, 3H), 0.87 (t, J = 6.5 Hz, 3H).

^{13}C NMR (101 MHz, $CDCl_3$) δ 76.98, 66.68, 49.05, 34.75, 31.89, 30.60, 30.27, 29.64, 29.60, 29.58, 29.32, 23.54, 22.67, 14.09.

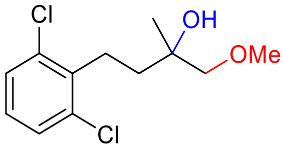

4-(2,6-Dichlorophenyl)-1-methoxy-2-methylbutan-2-ol (24-1)

^{1}H NMR (400 MHz, $CDCl_3$) δ 7.28 (d, J = 7.9 Hz, 2H), 7.07 (t, J = 8.0 Hz, 1H), 3.44 (s, 3H), 3.34 (dd, J = 19.7, 9.0 Hz, 2H), 3.11 – 2.93 (m, 2H), 1.99 (br, 1H), 1.86 – 1.65 (m, 2H), 1.31 (s, 3H).

^{13}C NMR (101 MHz, $CDCl_3$) δ 138.18, 135.19, 128.11, 127.46, 79.78, 71.95, 59.29, 36.83, 25.97, 23.21.

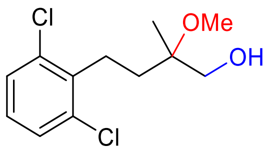

4-(2,6-Dichlorophenyl)-2-methoxy-2-methylbutan-1-ol (24-2)

^{1}H NMR (400 MHz, $CDCl_3$) δ 7.29 (d, J = 8.0 Hz, 2H), 7.08 (t, J = 8.0 Hz, 1H), 3.67 (d, J = 11.2 Hz, 1H), 3.53 (d, J = 11.5 Hz, 1H), 3.34 (s, 3H), 2.97 (t, J = 7.4 Hz, 2H), 2.00 (br, 1H), 1.84 – 1.65 (m, 2H), 1.29 (s, 3H).

^{13}C NMR (101 MHz, $CDCl_3$) δ 137.94, 135.13, 128.16, 127.57, 76.70, 66.85, 49.42, 32.46, 25.77, 19.61.

7. References

1 Le Bourdonnec, B. & Dolle, R. E., III. Novel processes for the preparation of piperidinopropanoic acids and derivatives as peripheral μ opioid antagonist compounds and intermediates for gastrointestinal motility disorders and peripheral opiate-induced side effects. Application: WO patent 2004014310 (2004).

2 Bertrand, X. & Paquin, J.-F. Direct Hydrofluorination of methallyl alkenes using a methanesulfonic acid/triethylamine trihydrofluoride combination. *Org. Lett.* **21**, 9759-9762 (2019).

3 Cabré, A. *et al.* Iridium-catalyzed isomerization of n-sulfonyl aziridines to allyl amines. *Org. Lett.* **20**, 5747-5751 (2018).

4 Yang, B. & Lu, Z. Visible-light-promoted metal-free aerobic hydroxyazidation of alkenes. *ACS Catal.* **7**, 8362-8365 (2017).

5 Yu, Y.-B. *et al.* Enantioselective iridium-catalyzed hydrogenation of β,β-disubstituted nitroalkenes. *Chem. Commun.* **52**, 4812-4815 (2016).

6 Serra, S. Lipase-mediated resolution of substituted 2-aryl-propanols: application to the enantioselective synthesis of phenolic sesquiterpenes. *Tetrahedron: Asymmetry* **22**, 619-628 (2011).

7 Gonzalez-de-Castro, A. & Xiao, J. Green and efficient: iron-catalyzed selective oxidation of olefins to carbonyls with O_2. *J. Am. Chem. Soc.* **137**, 8206-8218 (2015).

8 Tripathi, C. B. & Mukherjee, S. Catalytic enantioselective iodoetherification of oximes. *Angew. Chem., Int. Ed.* **52**, 8450-8453 (2013).

9 Li, R., Wang, S. R. & Lu, W. $FeCl_3$-catalyzed alkenylation of simple arenes with aryl-substituted alkynes. *Org. Lett.* **9**, 2219-2222 (2007).

10 Yichen, W., Tao, l., Dongping, C., Daoming, W. & Peng, W. H_2O as the sole hydrogen source for Ni-catalyzed reduction of alkenes. 10.26434/chemrxiv.8067446 (2019).

11 Hayashi, K. *et al.* Prepn of condensed heterocycle derivatives as NPY Y5 receptor antagonists for treatment of obesity. Application: JP patent 2012167027 (2012).

12 Liwosz, T. W. & Chemler, S. R. Copper-catalyzed oxidative amination and allylic amination of alkenes. *Chem. Eur. J.* **19**, 12771-12777 (2013).

13 Teo, W. J. & Ge, S. Cobalt-catalyzed enantioselective synthesis of chiral gem-bis(boryl)alkanes. *Angew. Chem., Int. Ed.* **57**, 12935-12939 (2018).

14 Fu, N., Sauer, G. S., Saha, A., Loo, A. & Lin, S. Metal-catalyzed electrochemical diazidation of alkenes. *Science* **357**, 575 (2017).

15 Xue, F., Zhao, J. & Hor, T. S. A. Ambient arylmagnesiation of alkynes catalysed by ligandless nickel(II). *Chem. Commun.* **49**, 10121-10123 (2013).

16 Biosca, M. *et al.* Extending the substrate scope in the hydrogenation of unfunctionalized tetrasubstituted olefins with Ir-P stereogenic aminophosphine–oxazoline catalysts. *Org. Lett.* **21**, 807-811 (2019).

17 Perrott, A. & Arnold, D. The importance of conformation in the reactivity of radical cations. Changing configuration at saturated carbon centres. *Can. J. Chem.* **70**, 272-279 (2011).

18 Goseki, R., Onuki, S., Tanaka, S., Ishizone, T. & Hirao, A. Living anionic polymerization of 1,4-diisopropenylbenzene. *Macromolecules* **48**, 3230-3238 (2015).

19 Huang, D., Schuppe, A. W., Liang, M. Z. & Newhouse, T. R. Scalable procedure for the fragmentation of hydroperoxides mediated by copper and iron tetrafluoroborate salts. *Org. Biomol. Chem.* **14**, 6197-6200 (2016).

20 Berliner, D. L. & Monti-Bloch, L. Steroids as neurochemical stimulators of the VNO to alleviate pain. Application: US patent 6331534 (2001).

21 Eaton, S. S., Ngendahimana, T., Eaton, G. R., Jupp, A. R. & Stephan, D. W. Electron paramagnetic resonance of a 10B-containing heterocyclic radical. *J. Magn. Reson.* **290**, 76-84 (2018).

22 Stoll, S. & Schweiger, A. EasySpin, a comprehensive software package for spectral simulation and analysis in EPR. *J. Magn. Reson.* **178**, 42-55 (2006).

23 Rich, J. *et al.* Investigation of the zero-field splitting in six- and seven-coordinate mononuclear Mn^{II} complexes with N/O-based ligands by combining EPR spectroscopy and quantum chemistry. *Eur. J. Inorg. Chem.* **2010**, 3658-3665 (2010).

24 Schäfer, K.-O. *et al.* Electronic structure of antiferromagnetically coupled dinuclear manganese ($Mn^{III}Mn^{IV}$) complexes studied by magnetic resonance techniques. *J. Am. Chem. Soc.* **120**, 13104-13120 (1998).

25 Sheng, Y. *et al.* Comparison of two yeast MnSODs: mitochondrial saccharomyces cerevisiae versus cytosolic candida albicans. *J. Am. Chem. Soc.* **133**, 20878-20889 (2011).

26 Gupta, R. *et al.* High-spin Mn–oxo complexes and their relevance to the oxygen-evolving complex within photosystem II. *PNAS* **112**, 5319-5324 (2015).

27 Stathi, P., Louloudi, M. & Deligiannakis, Y. EPR monitoring of in-situ catalytic oxidative assembly of Mn^{III}-Mn^{IV} dimers via monomeric Mn^{IV} = O. *Chem. Phys. Lett.* **763**, 138255 (2021).

28 Colmer, H. E., Howcroft, A. W. & Jackson, T. A. Formation, characterization, and O–O bond activation of a peroxomanganese(III) complex supported by a cross-clamped cyclam ligand. *Inorg. Chem.* **55**, 2055-2069 (2016).

29 Ghosh, A. K. & Nicponski, D. R. Cu(II)-catalyzed olefin migration and prins cyclization: highly diastereoselective synthesis of substituted tetrahydropyrans. *Org. Lett.* **13**, 4328-4331 (2011).

30 Hendrickson, J. B., Boudreaux, G. J. & Palumbo, P. S. Nuclear synthons: mesyltriflone as an olefin polyanion equivalent. *J. Am. Chem. Soc.* **108**, 2358-2366 (1986).

31 Suresh, R., Simlandy, A. K. & Mukherjee, S. A catalytic enantioselective iodocyclization route to dihydrooxazines. *Org. Lett.* **20**, 1300-1303 (2018).

32 Han, X. & Widenhoefer, R. A. Palladium-mediated cyclization of 6-methyl-1-phenyl-6-hepten-2-one to form 3-methyldiphenylmethane: carbonyl activation by a neutral palladium(ii) complex. *Organometallics* **26**, 4061-4065 (2007).

33 Lebel, H., Davi, M., Díez-González, S. & Nolan, S. P. Copper−carbene complexes as catalysts in the synthesis of functionalized styrenes and aliphatic alkenes. *J. Org. Chem.* **72**, 144-149 (2007).

34 Chen, H., Sun, S. & Liao, X. Nickel-catalyzed decarboxylative alkenylation of anhydrides with vinyl triflates or halides. *Org. Lett.* **21**, 3625-3630 (2019).

35 Nifant'ev, I. E. *et al.* Synthesis of methyl β-alkylcarboxylates by Pd/diphosphine-catalyzed methoxycarbonylation of methylenealkanes $RCH_2CH_2C(R)=CH_2$. *Appl. Catal., A* **581**, 123-132 (2019).

36 Waser, J., Gaspar, B., Nambu, H. & Carreira, E. M. Hydrazines and azides via the metal-catalyzed hydrohydrazination and hydroazidation of olefins. *J. Am. Chem. Soc.* **128**, 11693-11712 (2006).

37 Sha, Q., Ling, Y., Wang, W. & Wei, Y. Capture of in situ generated diazo compounds or copper carbenoids by triphenylphosphine: selective synthesis of trans-alkenes and unsymmetric azines via reaction of aldehydes with ketone-derived N-tosylhydrazones. *Adv. Synth. Catal.* **355**, 2145-2150 (2013).

38 Johnson, C. R. & Elliott, R. C. Synthesis of alkenes with P-(α-lithioalkyl)phosphinothioic amides. *J. Am. Chem. Soc.* **104**, 7041-7044 (1982).

39 Lin, C.-C., Teng, T.-M., Tsai, C.-C., Liao, H.-Y. & Liu, R.-S. Gold-catalyzed deoxygenative nazarov cyclization of 2,4-dien-1-als for stereoselective synthesis of highly substituted cyclopentenes. *J. Am. Chem. Soc.* **130**, 16417-16423 (2008).

40 Tokuyasu, T., Kunikawa, S., McCullough, K. J., Masuyama, A. & Nojima, M. Synthesis of cyclic peroxides by chemo- and regioselective peroxidation of dienes with Co(II)/O_2/Et_3SiH. *J. Org. Chem.* **70**, 251-260 (2005).

41 Wang, X., Wang, Z., Asanuma, Y. & Nishihara, Y. Synthesis of 2-substituted propenes by bidentate phosphine-assisted methylenation of acyl fluorides and acyl chlorides with $AlMe_3$. *Org. Lett.* **21**, 3640-3643 (2019).

42 Zou, S., Gao, B., Huang, Y., Zhang, T. & Huang, H. Palladium-catalyzed hydrocarbonylative cyclization of 1,5-dienes. *Org. Lett.* **21**, 6333-6336 (2019).

43 Davis, C. E. & Coates, R. M. Stereoselective prins cyclizations of δ,ε-unsaturated ketones to cis-3-chlorocyclohexanols with $TiCl_4$. *Angew. Chem., Int. Ed.* **41**, 491-493 (2002).

44 Kulkarni, M. G. & Sebastian, M. T. An efficient synthesis of τ/δ keto esters. *Synth. Commun.* **21**, 581-586 (1991).

45 Jeschke, J., Korb, M., Rüffer, T., Gäbler, C. & Lang, H. Atom economic ruthenium-catalyzed synthesis of bulky β-oxo esters. *Adv. Synth. Catal.* **357**, 4069-4081 (2015).

46 Yang, M., Xing, Z., Fang, B., Xie, X. & She, X. Visible light photoredox catalyzed deprotection of 1,3-oxathiolanes. *Org. Biomol. Chem.* **18**, 288-291 (2020).

47 Ma, S. *et al.* Development of a general and practical iron nitrate/tempo-catalyzed aerobic oxidation of alcohols to aldehydes/ketones: catalysis with table salt. *Adv. Synth. Catal.* **353**, 1005-1017 (2011).

48 Brown, H. C., Kulkarni, S. V., Racherla, U. S. & Dhokte, U. P. Chiral synthesis via organoboranes. 47. efficient synthesis of unsymmetrical ketones and enantiomerically pure spiroketals using (±)-Isopinocampheyldichloroborane. *J. Org. Chem.* **63**, 7030-7036 (1998).

49 Fox, D. J., Pedersen, D. S. & Warren, S. Diphenylphosphinoyl-mediated synthesis of ketones. *Org. Biomol. Chem.* **4**, 3102-3107 (2006).

50 Ho, Y. A. *et al.* Catalytic wacker-type oxidations using visible light photoredox catalysis. *ChemCatChem* **11**, 1889-1892 (2019).

51 Zhang, W.-C. & Li, C.-J. A Direct retro-barbier fragmentation. *J. Org. Chem.* **65**, 5831-5833 (2000).

52 Ruan, J., Li, X., Saidi, O. & Xiao, J. Oxygen and base-free oxidative heck reactions of arylboronic acids with olefins. *J. Am. Chem. Soc.* **130**, 2424-2425 (2008).

53 Cai, C. *et al.* An efficient catalyst for pd-catalyzed carbonylation of aryl arenesulfonates. *Org. Lett.* **8**, 5161-5164 (2006).

54 Dohi, T., Takenaga, N., Goto, A., Fujioka, H. & Kita, Y. Clean and efficient benzylic C-H oxidation in water using a hypervalent iodine reagent: activation of polymeric iodosobenzene with KBr in the presence of montmorillonite-K10. *J. Org. Chem.* **73**, 7365-7368 (2008).

55 Bellale, E. V., Bhalerao, D. S. & Akamanchi, K. G. Oxidative conversion of α,α-disubstituted acetamides to corresponding one-carbon-shorter ketones using hypervalent iodine (λ5) reagents in combination with tetraethylammonium bromide. *J. Org. Chem.* **73**, 9473-9475 (2008).

56 Zhang, G. *et al.* Highly selective Wacker reaction of styrene derivatives: a green and efficient aerobic oxidative process promoted by benzoquinone/$NaNO_2$/$HClO_4$ under mild conditions. *Org. Biomol. Chem.* **11**, 2947-2950 (2013).

57 Li, M., Wang, C. & Ge, H. Pd(II)-catalyzed decarboxylative cross-coupling of potassium aryltrifluoroborates with α-oxocarboxylic acids at room temperature. *Org. Lett.* **13**, 2062-2064 (2011).

58 Andrade, L. H. & Barcellos, T. Lipase-catalyzed highly enantioselective kinetic resolution of boron-containing chiral alcohols. *Org. Lett.* **11**, 3052-3055 (2009).

59 Irfan, M., Glasnov, T. N. & Kappe, C. O. Continuous flow ozonolysis in a laboratory scale reactor. *Org. Lett.* **13**, 984-987 (2011).

60 Tauchman, J., Císařová, I. & Štěpnička, P. Chiral phosphanylferrocenecarboxamides with amino acid pendant groups as ligands for Cu-mediated asymmetric conjugate additions of diethylzinc to

chalcones – structural characterisation of precursors to the Cu catalyst. *Eur. J. Org. Chem.* **2010**, 4276-4287 (2010).

61 Zhao, Q., Henrion, G. & Gagosz, F. Synthesis of isochroman-4-ones and 2H-pyran-3(6H)-ones by gold-catalyzed oxidative cycloalkoxylation of alkynes. *Bioorg. Med. Chem.* **27**, 2616-2620 (2019).

62 Rocaboy, R., Anastasiou, I. & Baudoin, O. Redox-neutral coupling between two C(sp^3)–H bonds enabled by 1,4-palladium shift for the synthesis of fused heterocycles. *Angew. Chem., Int. Ed.* **58**, 14625-14628 (2019).

63 Hanson, S. K., Wu, R. & Silks, L. A. P. Mild and selective vanadium-catalyzed oxidation of benzylic, allylic, and propargylic alcohols using air. *Org. Lett.* **13**, 1908-1911 (2011).

64 Ji, S.-J. & Horiuchi, C. A. Photo-cleavage of carbon-carbon bond of α-iodocycloalkanones giving ω,ω-dialkoxyalkanoic ester in alcohol. *Bull. Chem. Soc. Jpn.* **73**, 1645-1652 (2000).

65 Murphy, A., Pace, A. & Stack, T. D. P. Ligand and pH influence on manganese-mediated peracetic acid epoxidation of terminal olefins. *Org. Lett.* **6**, 3119-3122 (2004).

66 Mei, Y., Bentley, P. A. & Du, J. NCS with thiourea as highly efficient catalysts for acetalization of aldehydes. *Tetrahedron Lett.* **50**, 4199-4200 (2009).

67 Guan, B. *et al.* Highly selective aerobic oxidation of alcohol catalyzed by a gold(i) complex with an anionic ligand. *J. Am. Chem. Soc.* **127**, 18004-18005 (2005).

68 Nikitas, N. F., Triandafillidi, I. & Kokotos, C. G. Photo-organocatalytic synthesis of acetals from aldehydes. *Green Chem.* **21**, 669-674 (2019).

69 Furusawa, M., Hashimoto, T., Noma, Y. & Asakawa, Y. Biotransformation of citrus aromatics nootkatone and valencene by microorganisms. *Chem. Pharm. Bull.* **53**, 1423-1429 (2005).

70 Hunter, A. C. & Priest, S.-M. An efficient one-pot synthesis generating 4-ene-3,6-dione functionalised steroids from steroidal 5-en-3β-ols using a modified Jones oxidation methodology. *Steroids* **71**, 30-33 (2006).

71 Zhao, L. *et al.* Design, synthesis, and estrogenic activity of a novel estrogen receptor modulator - A hybrid structure of 17β-estradiol and vitamin E in hippocampal neurons. *J. Med. Chem.* **50**, 4471-4481 (2007).

72 Dolomanov, O. V., Bourhis, L. J., Gildea, R. J., Howard, J. A. K. & Puschmann, H. OLEX2: a complete structure solution, refinement and analysis program. *J. Appl. Crystallogr.* **42**, 339-341 (2009).

73 Sheldrick, G. M. SHELXT – Integrated space-group and crystal-structure determination. *Acta Cryst.* A7**1**, 3-8 (2015).

74 Sheldrick, G. M. Crystal structure refinement with SHELXL. *Acta Cryst.* C**71**, 3-8 (2015).

8. NMR spectra of products

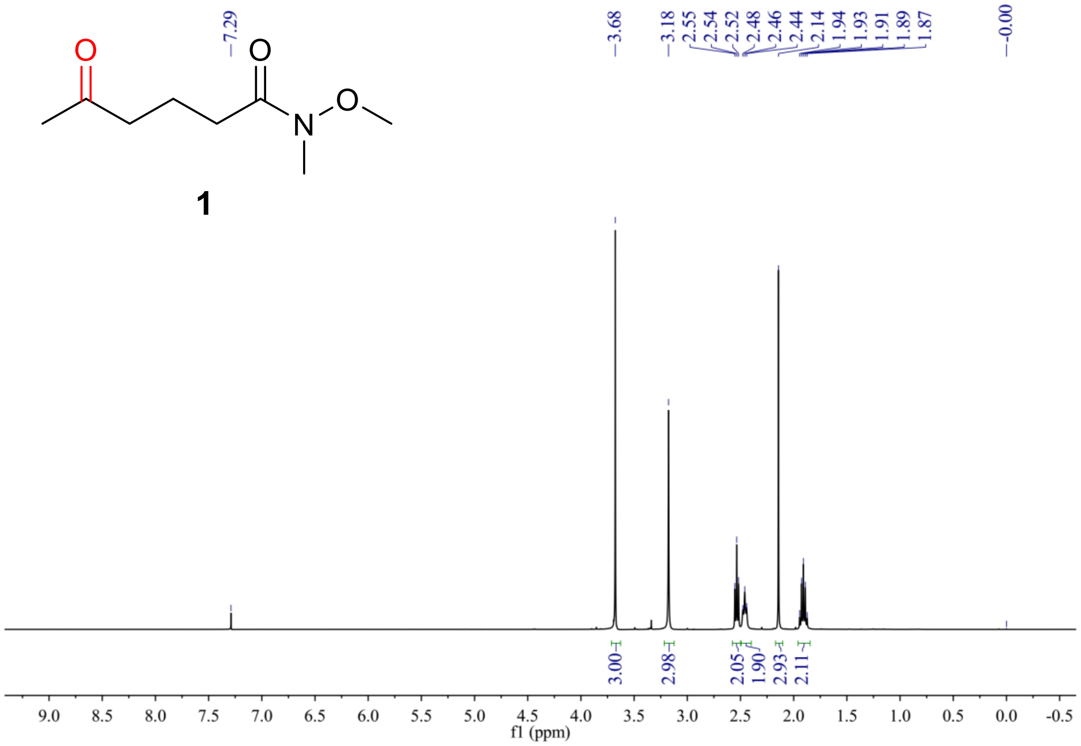

1

^{1}H NMR

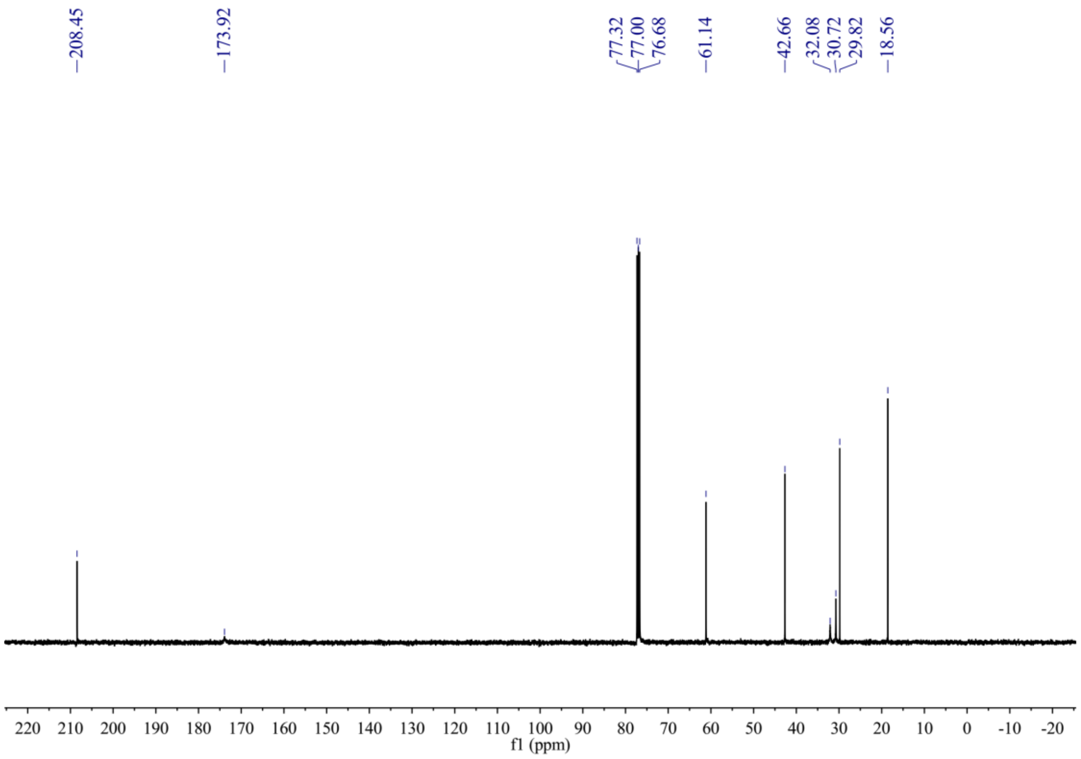

^{13}C NMR

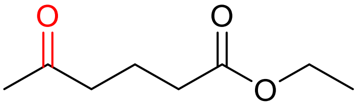

2

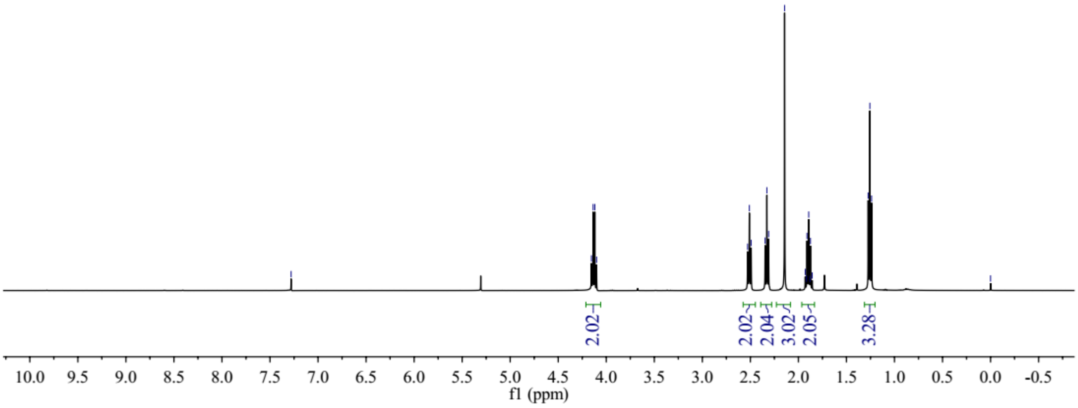

^{1}H NMR

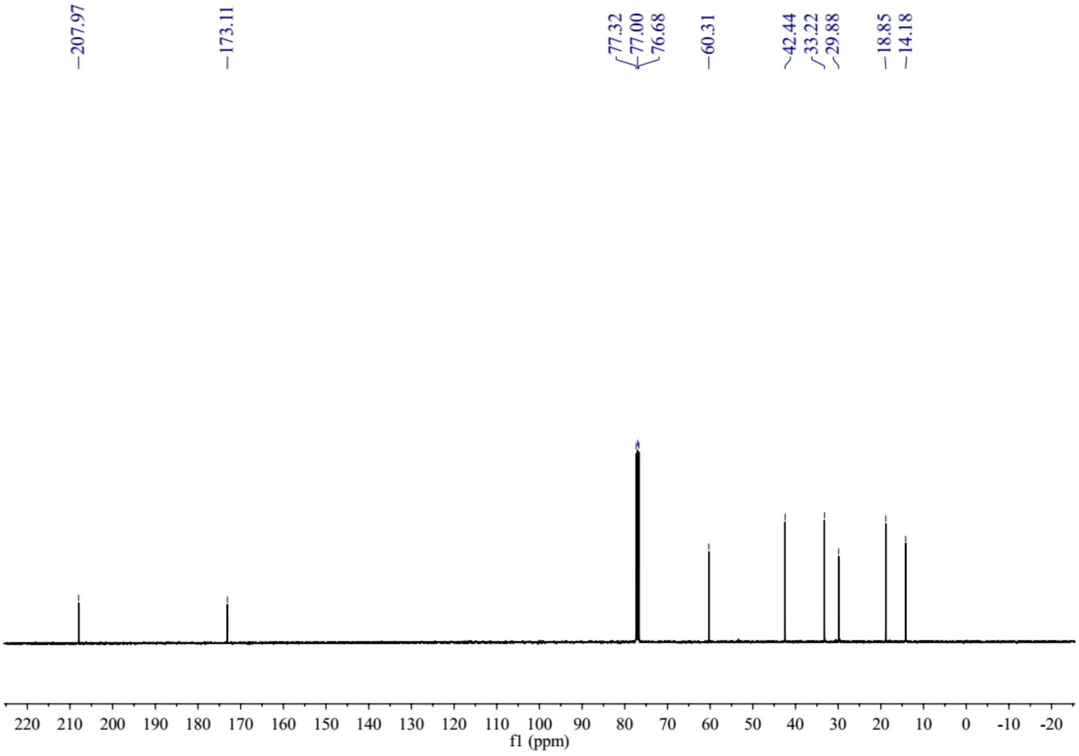

^{13}C NMR

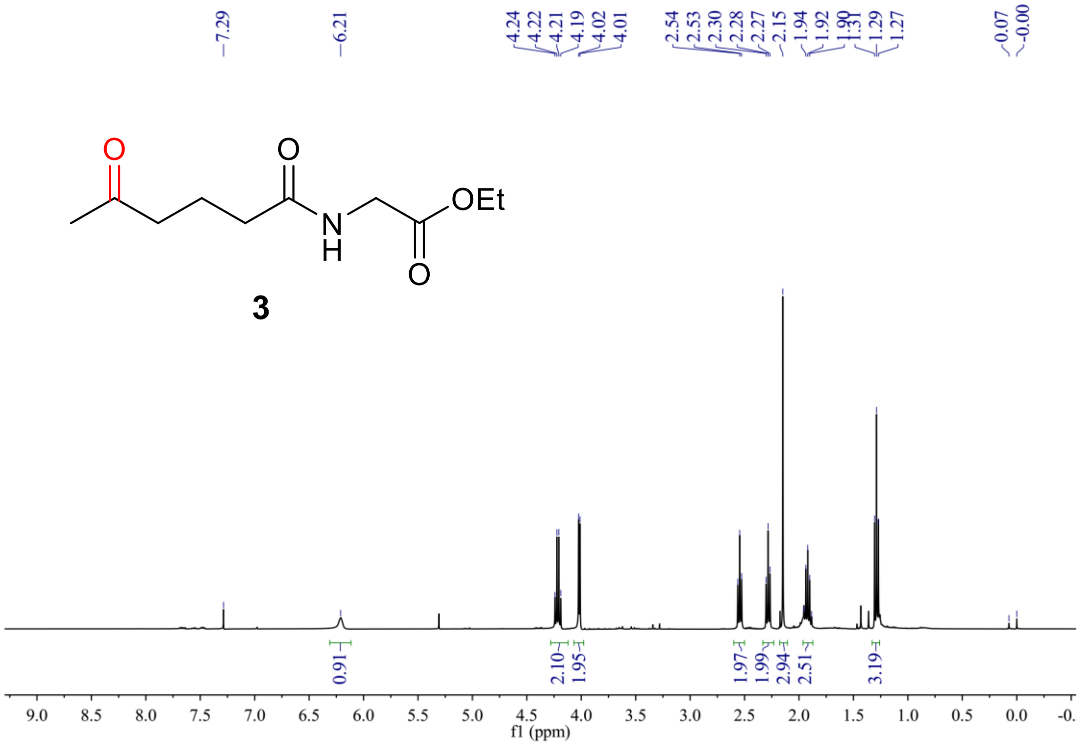

^{1}H NMR

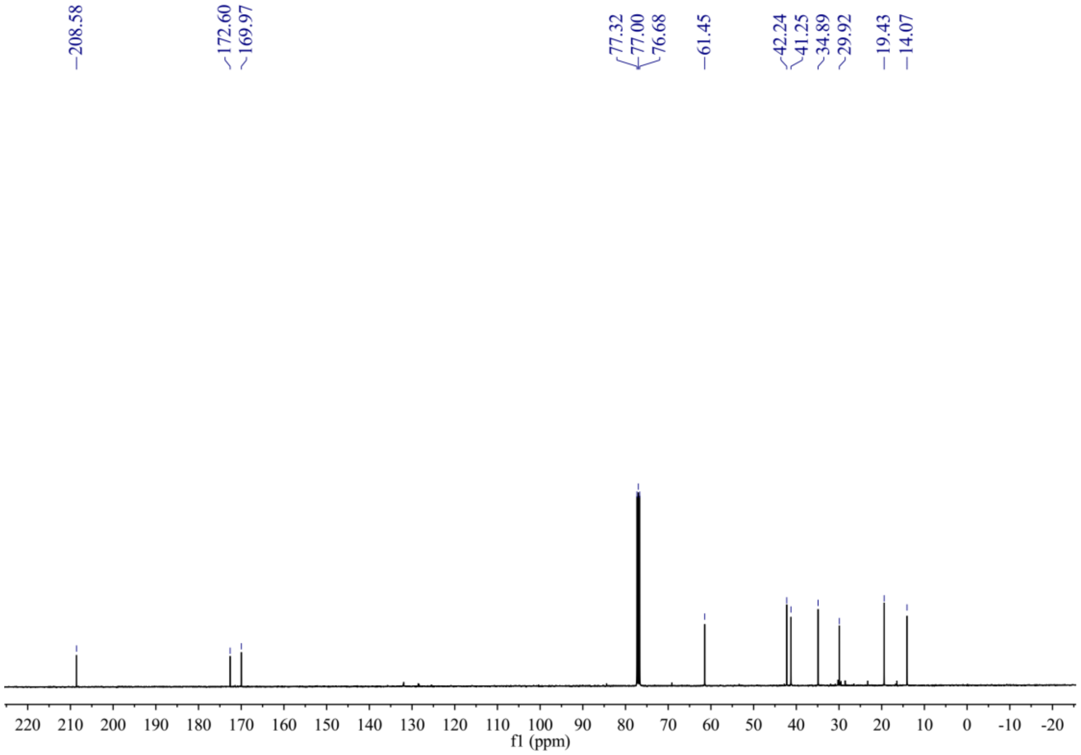

^{13}C NMR

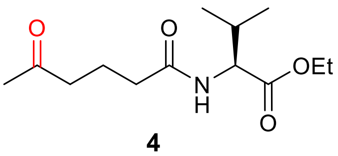

4

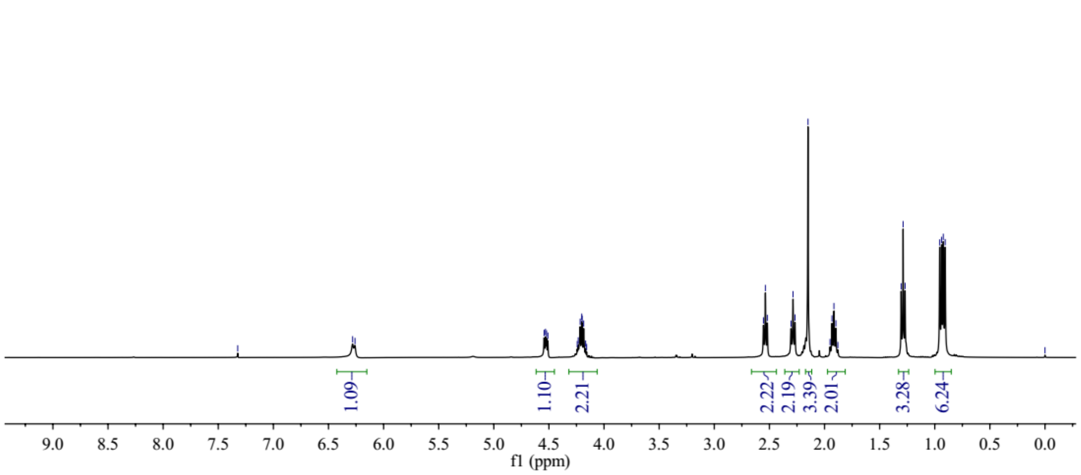

^{1}H NMR

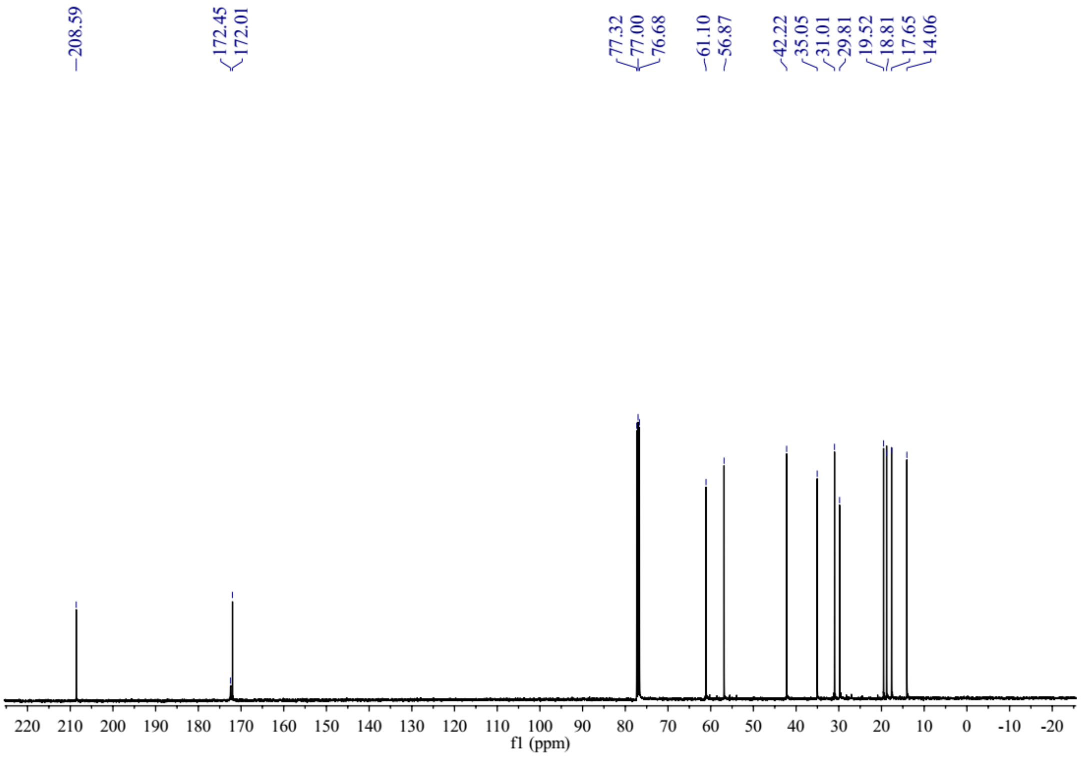

^{13}C NMR

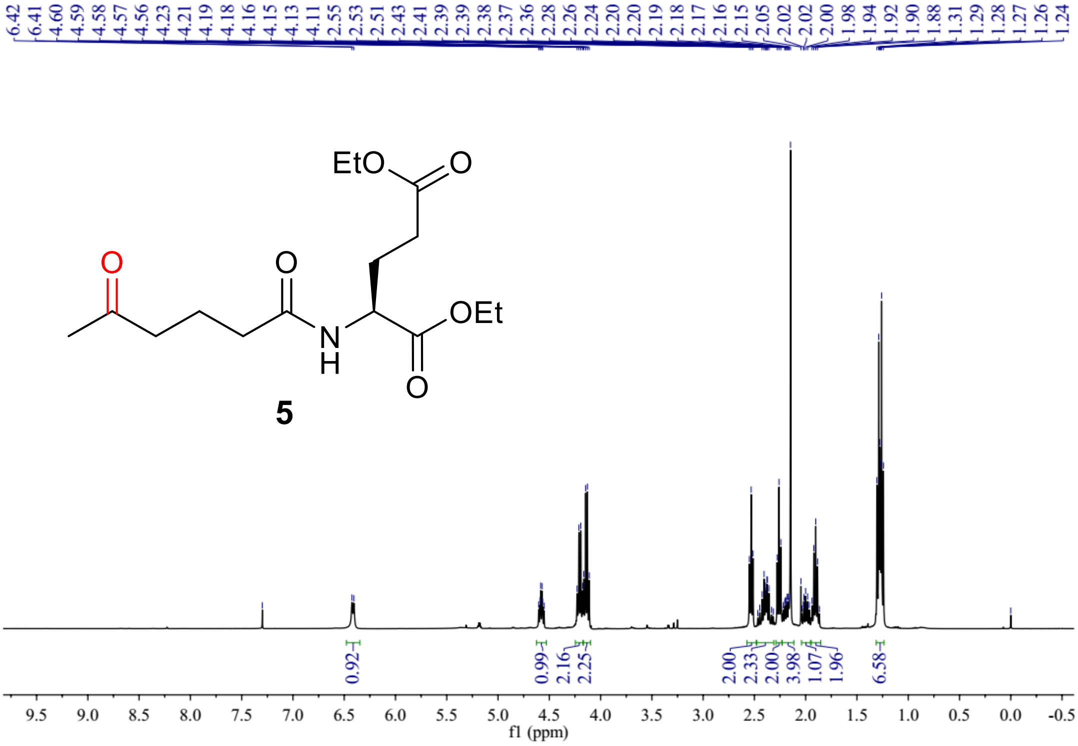

^{1}H NMR

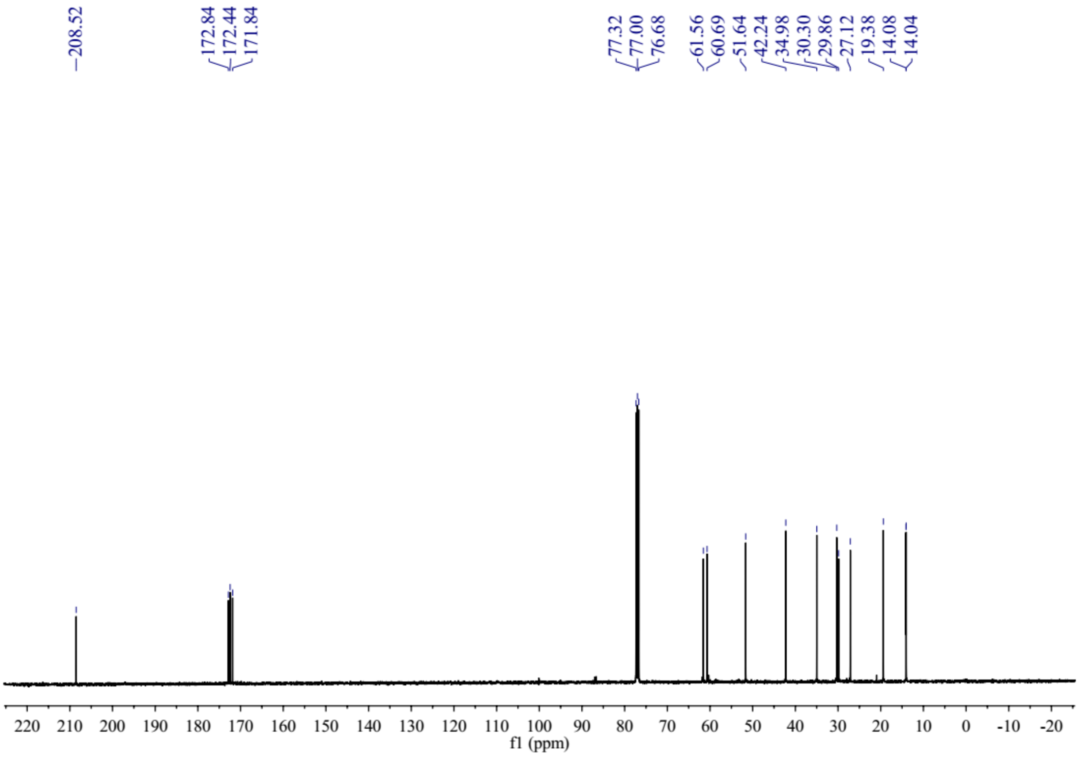

^{13}C NMR

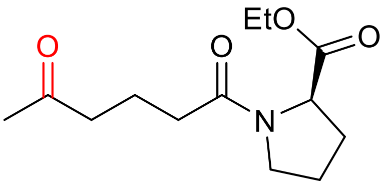

6 (dr = 81:19)

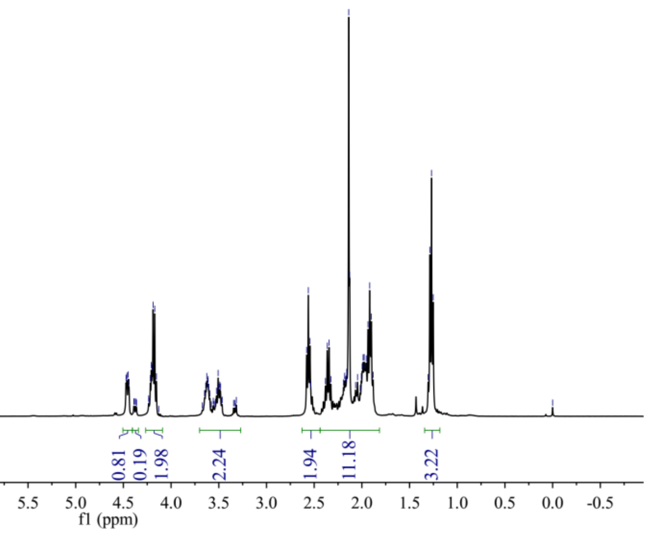

^{1}H NMR

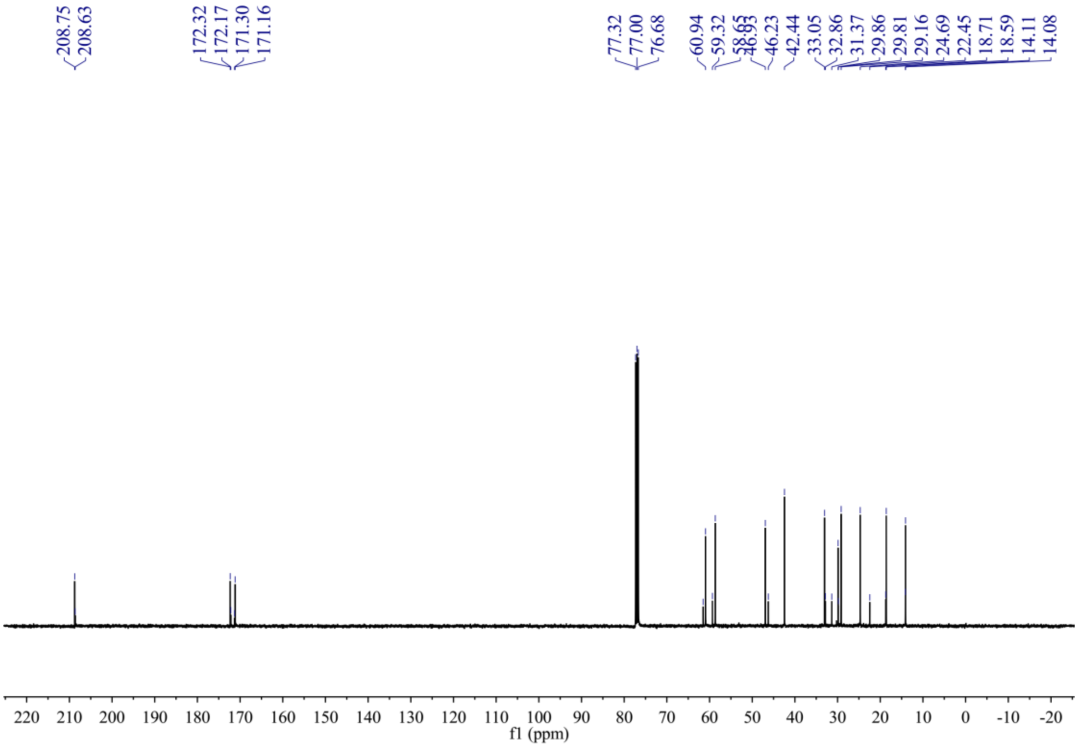

^{13}C NMR

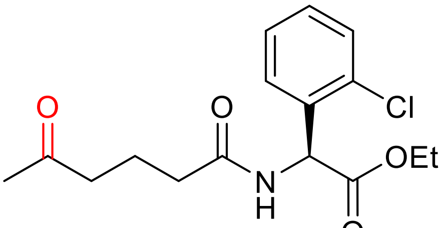

7

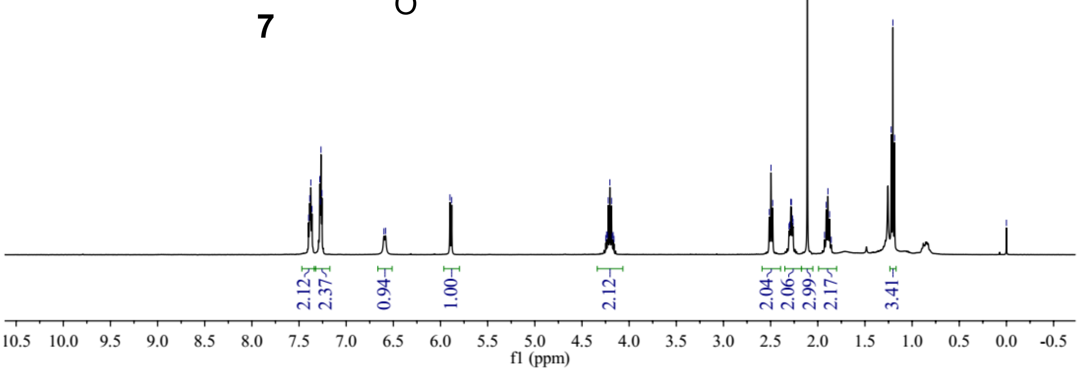

^{1}H NMR

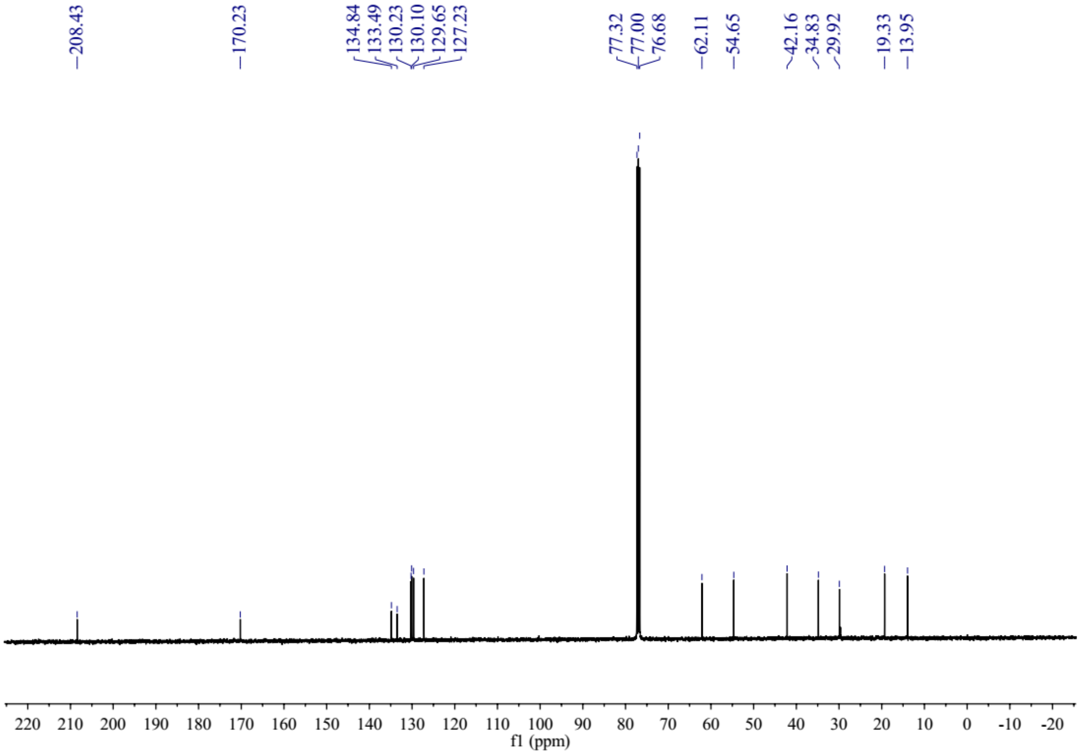

^{13}C NMR

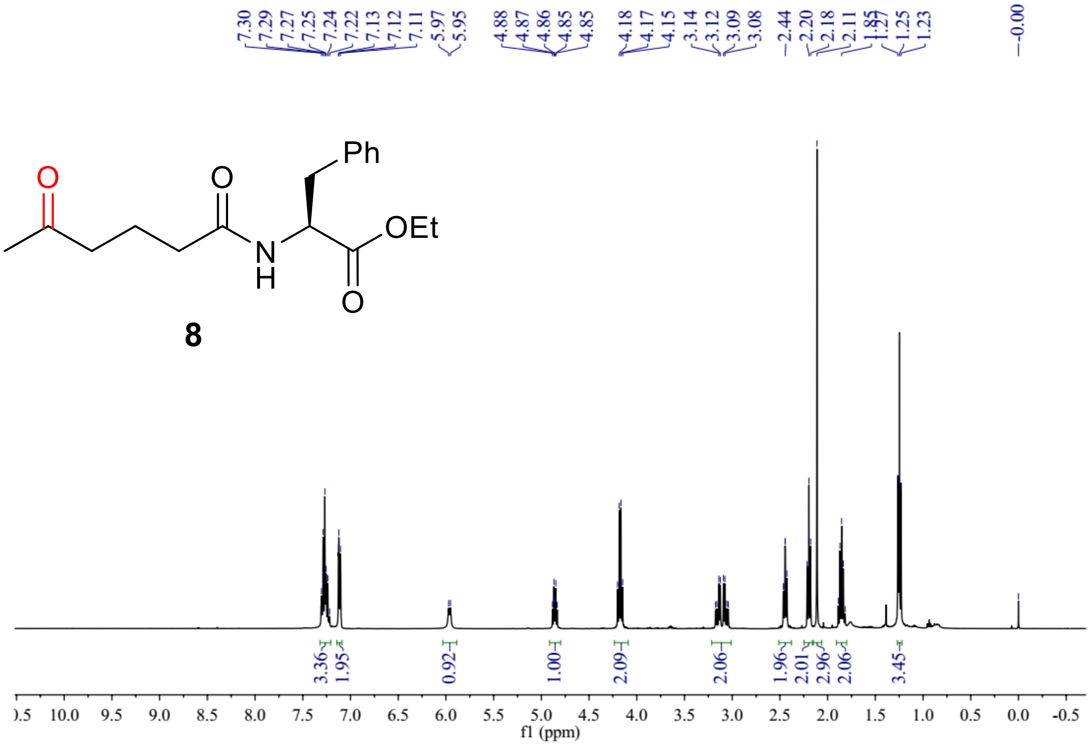

^{1}H NMR

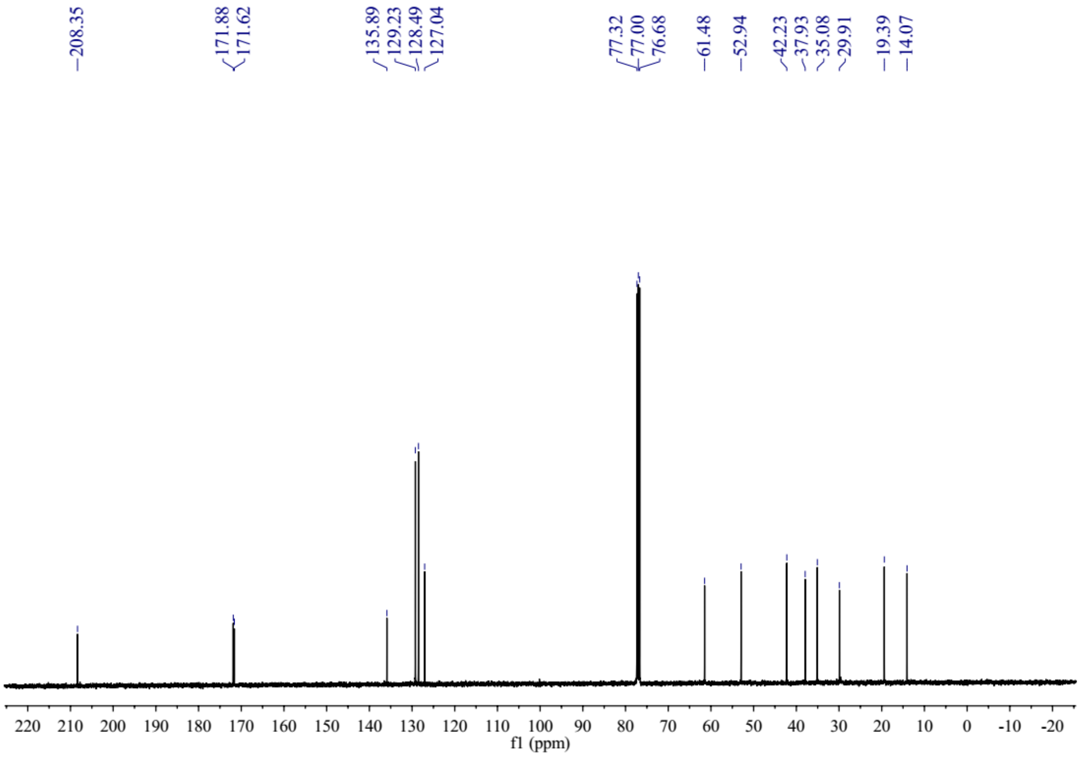

^{13}C NMR

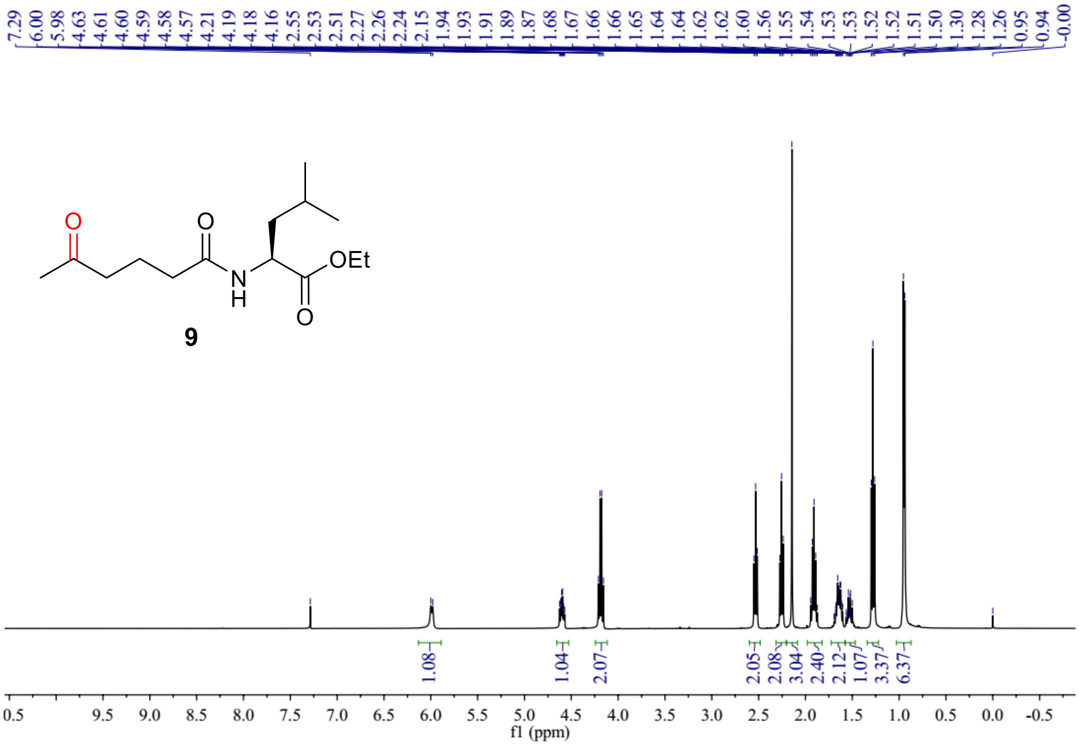

^{1}H NMR

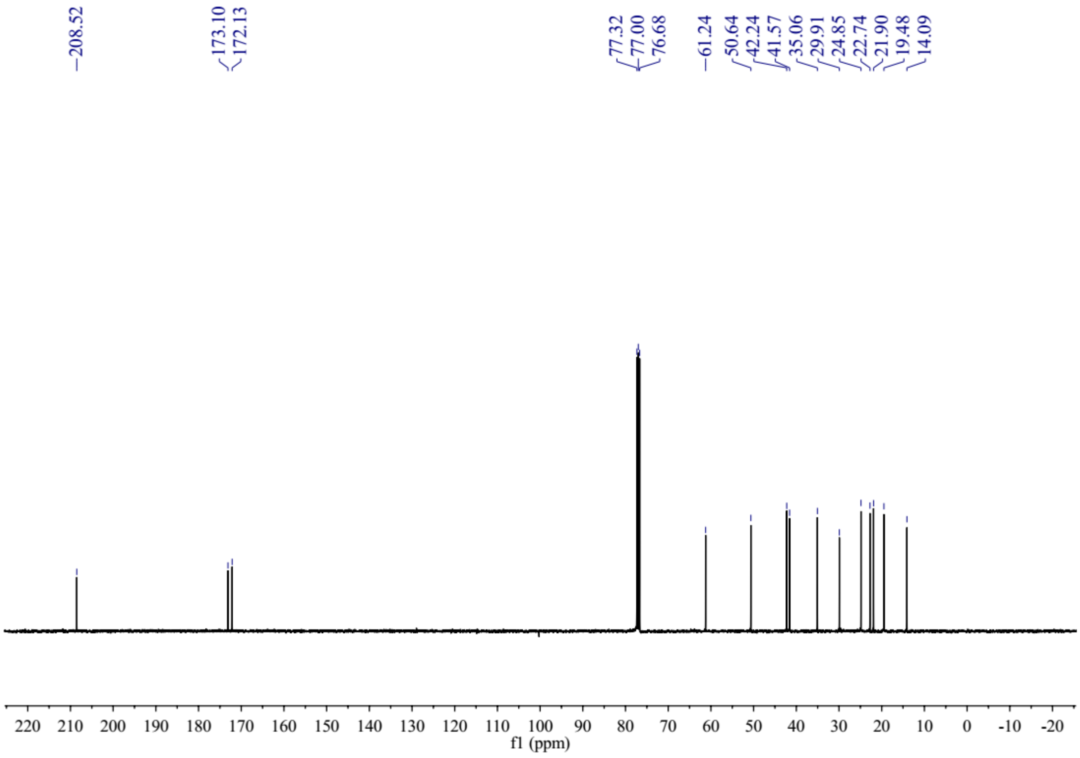

^{13}C NMR

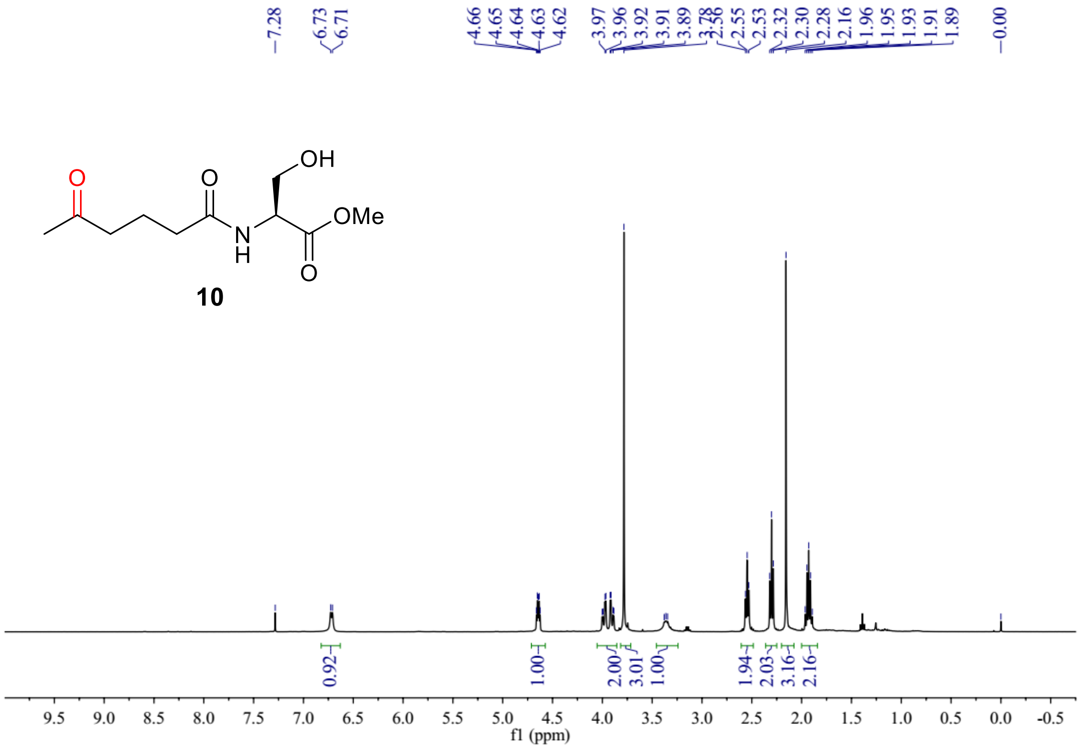

^{1}H NMR

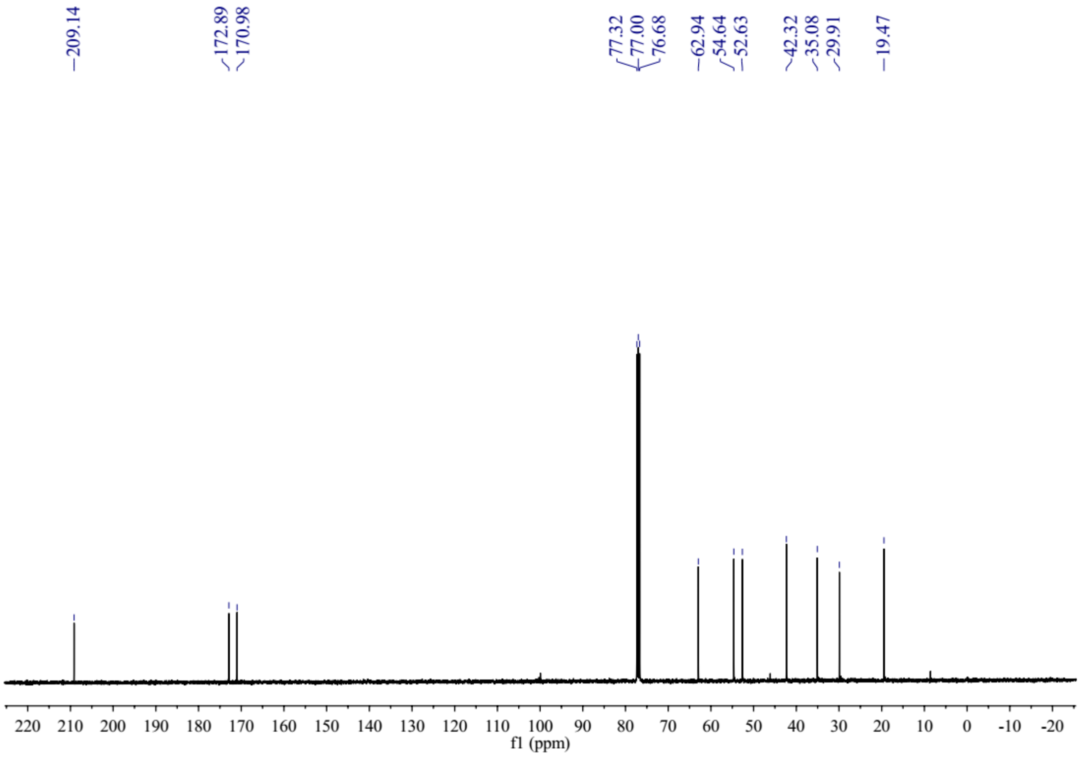

^{13}C NMR

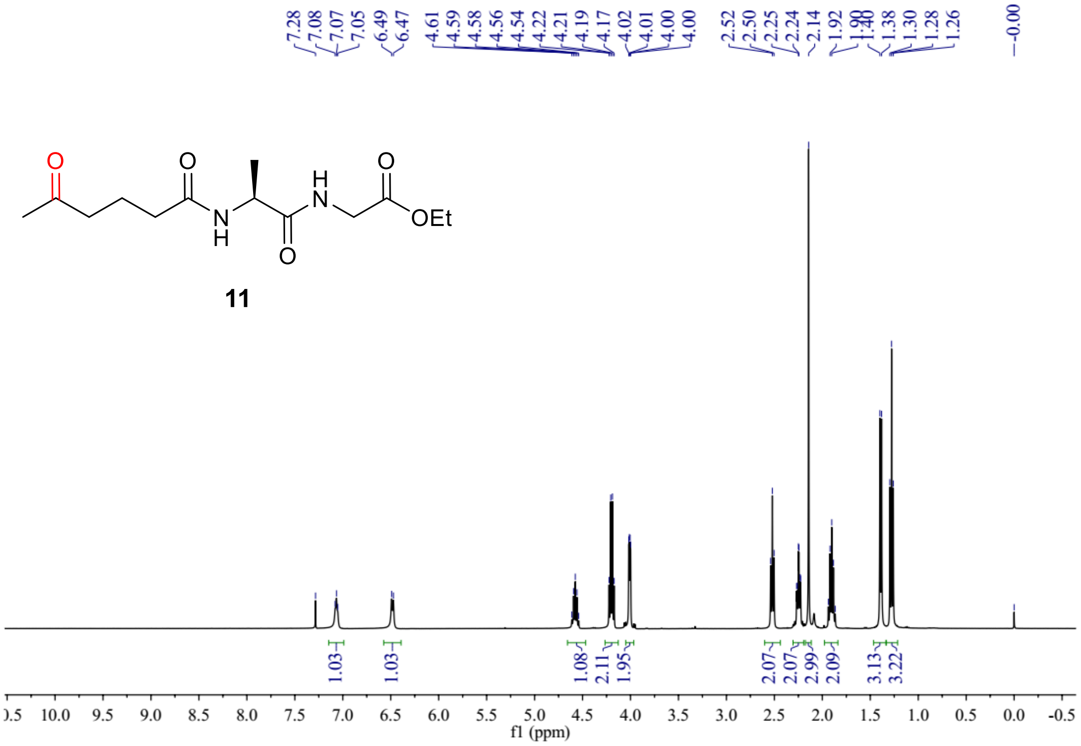

^{1}H NMR

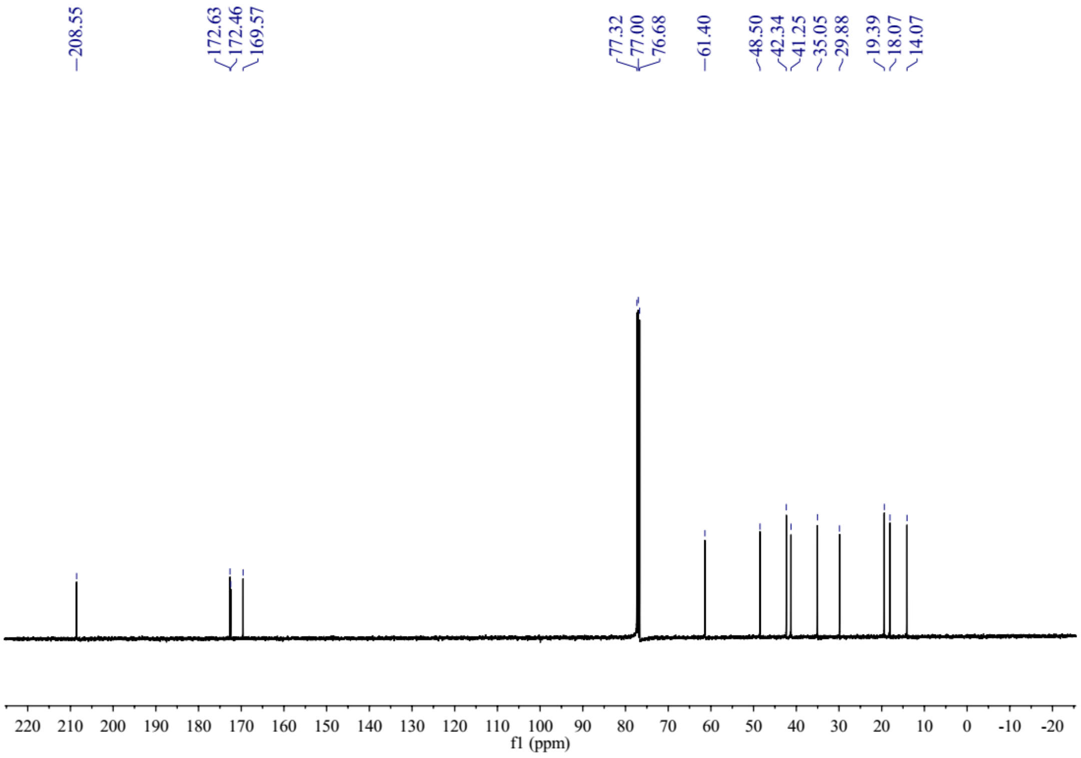

^{13}C NMR

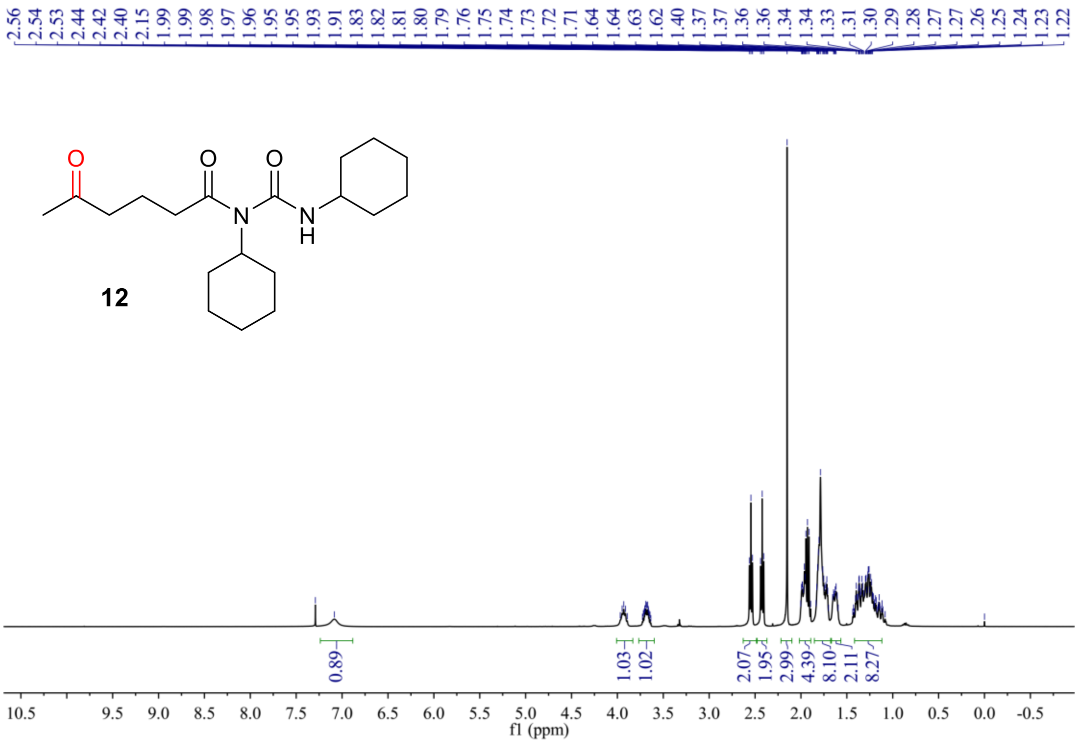

12

^{1}H NMR

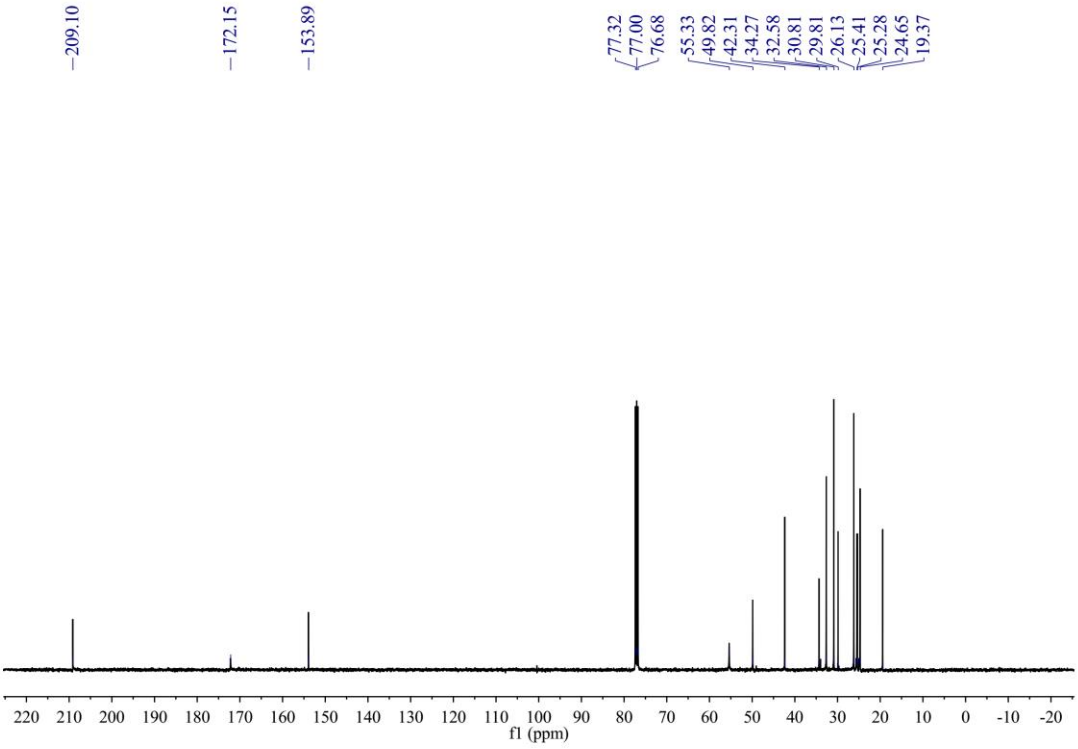

^{13}C NMR

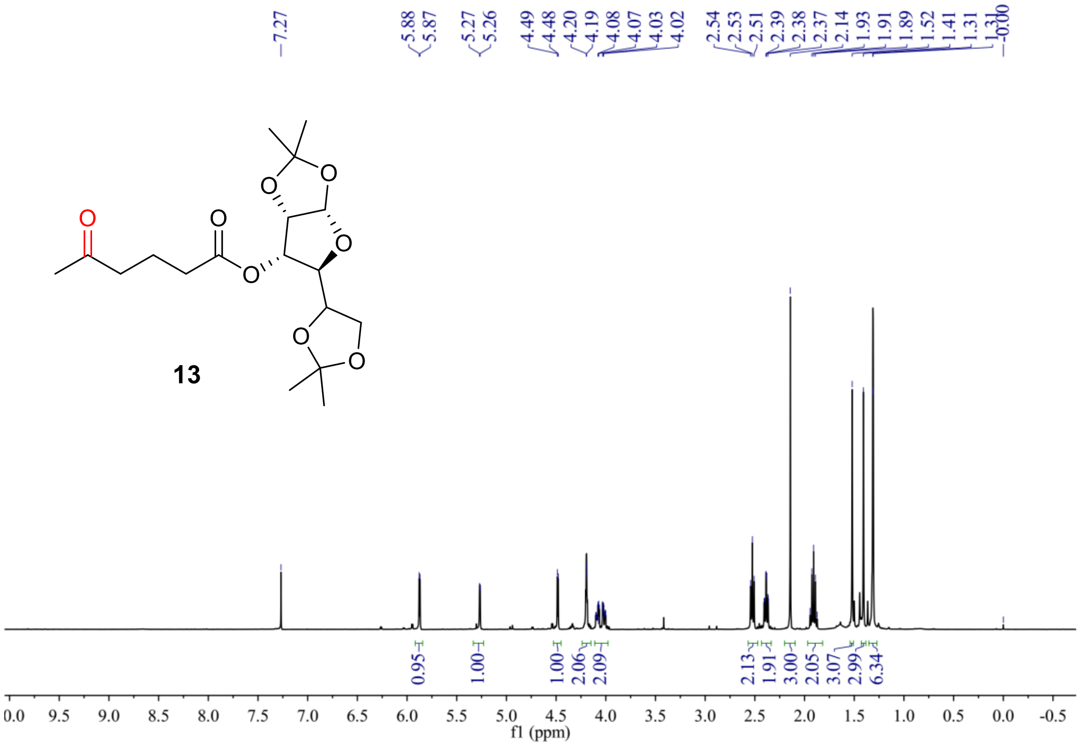

^{1}H NMR

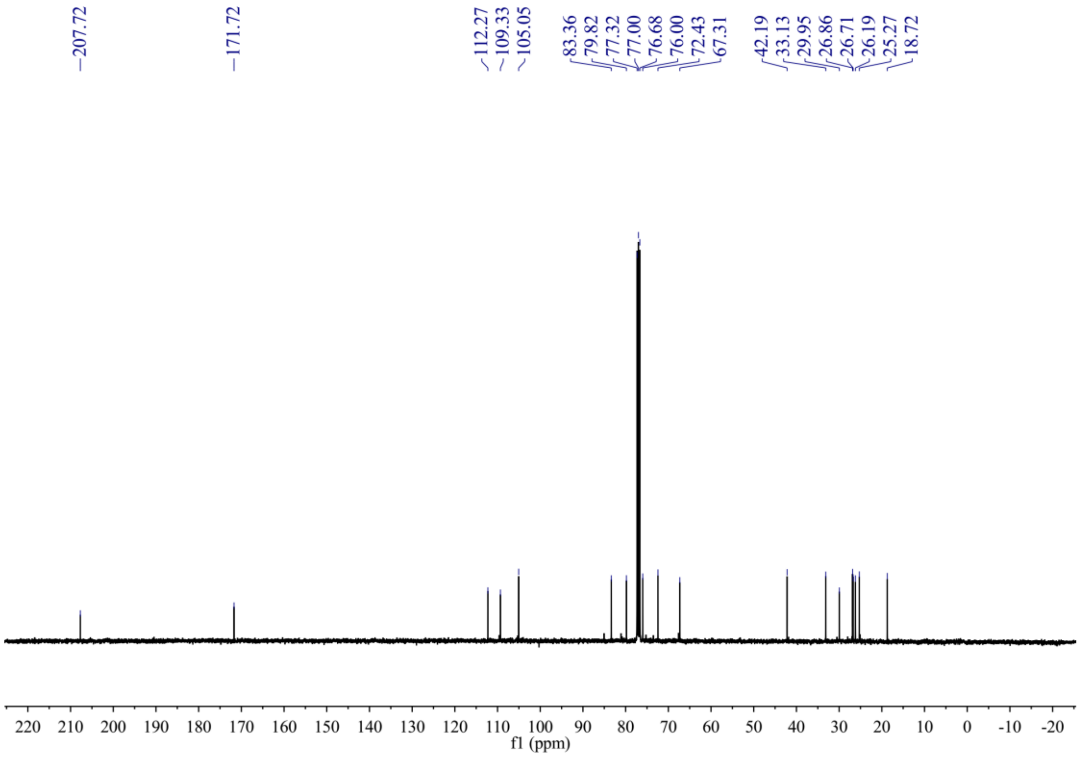

^{13}C NMR

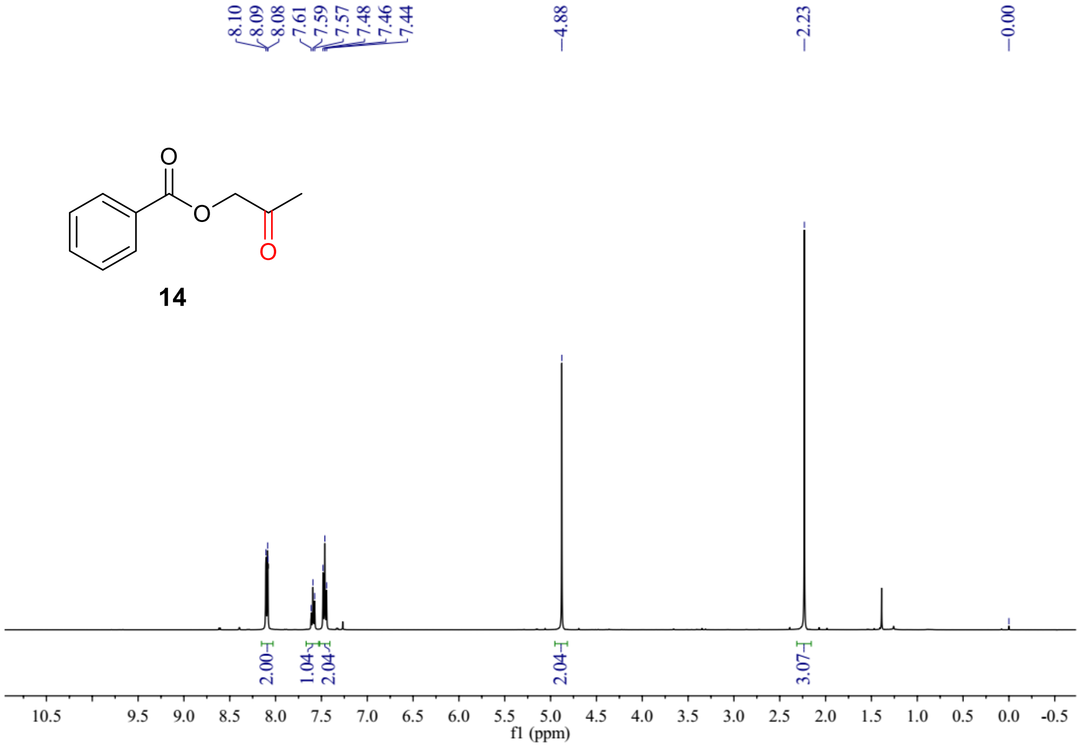

^{1}H NMR

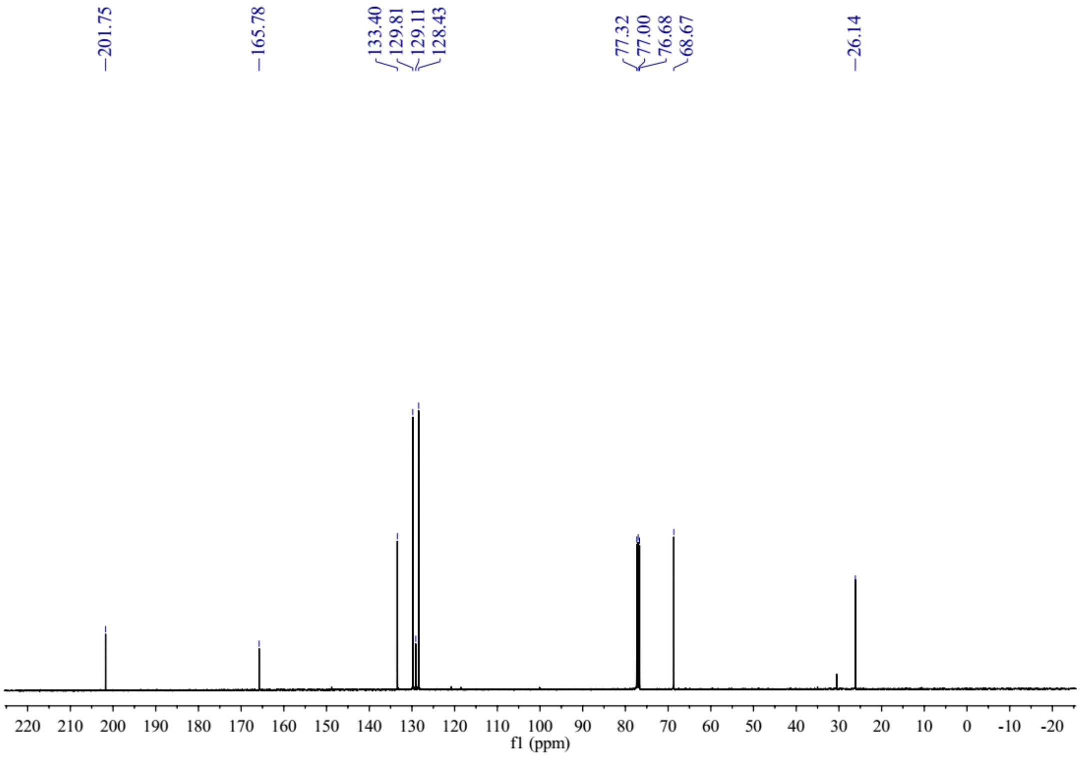

^{13}C NMR

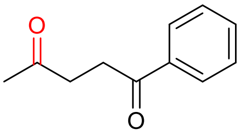

15

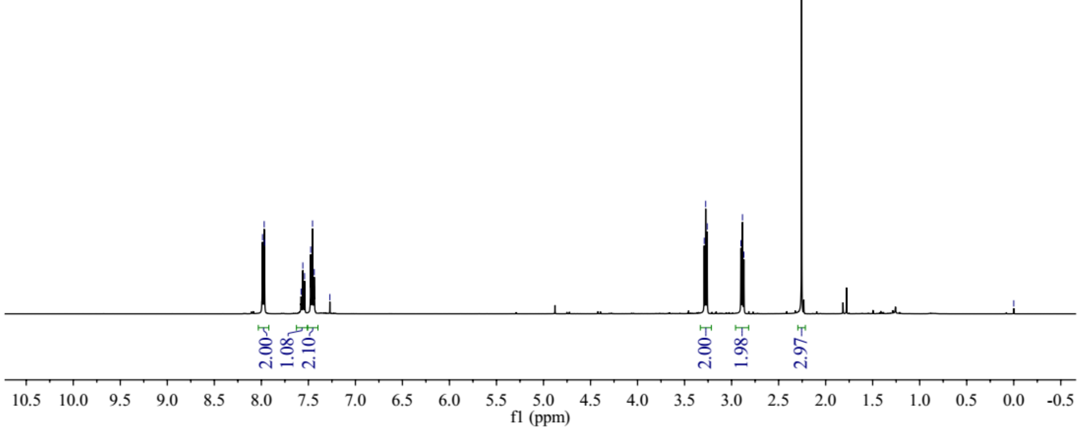

^{1}H NMR

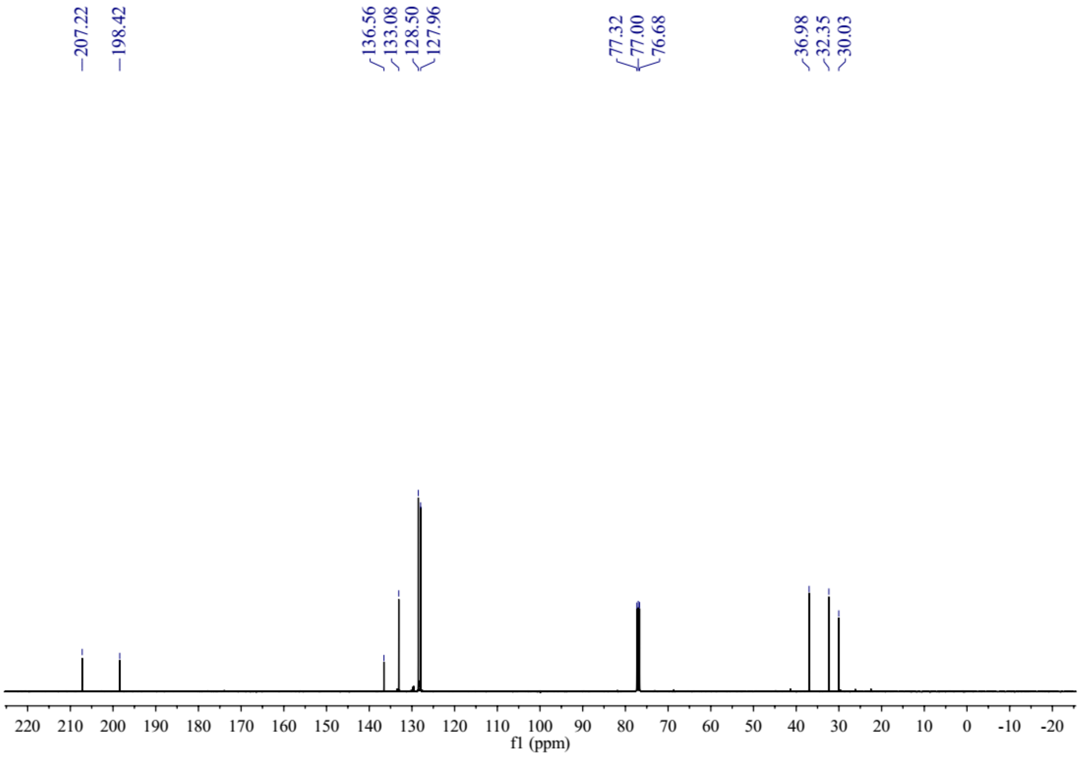

^{13}C NMR

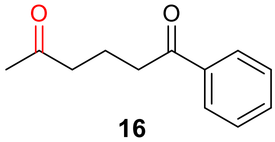

16

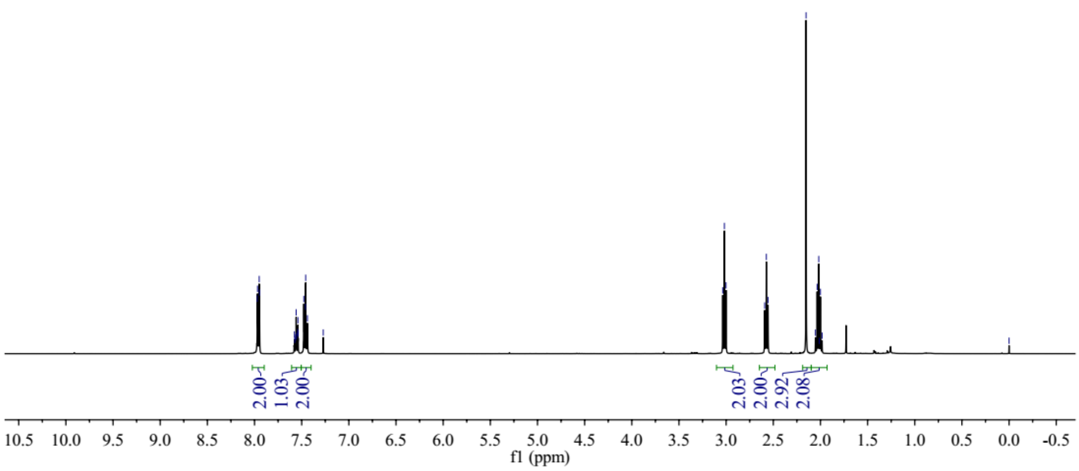

^{1}H NMR

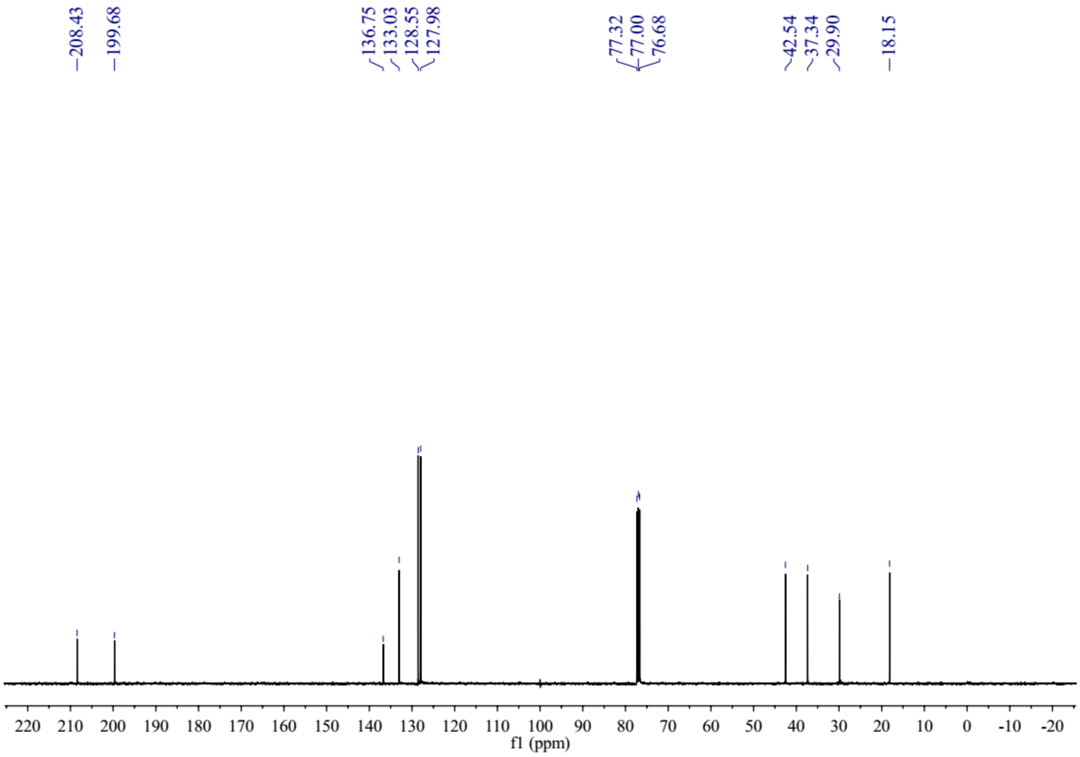

^{13}C NMR

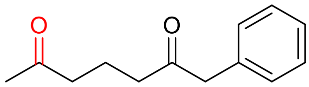

17

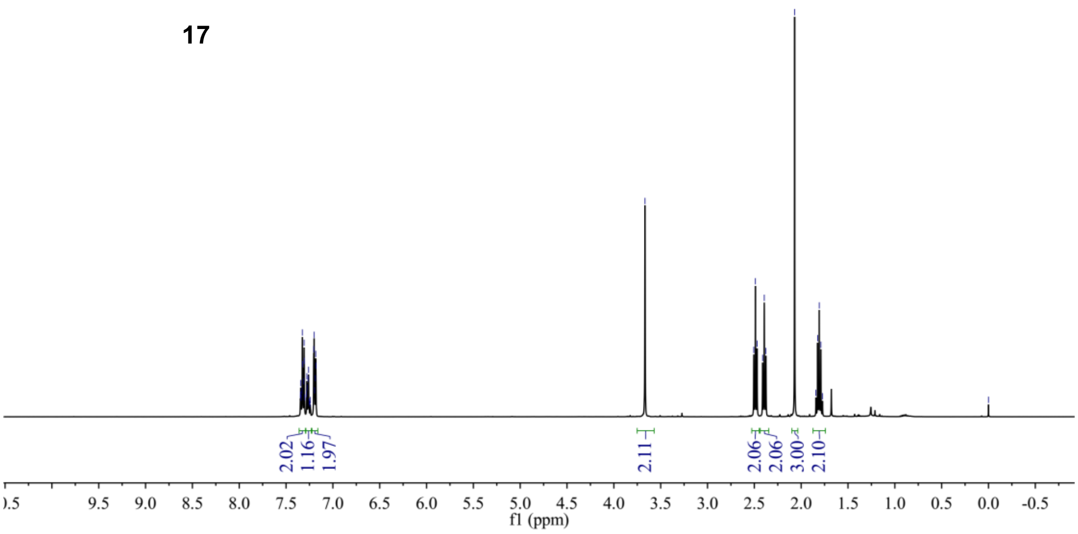

^{1}H NMR

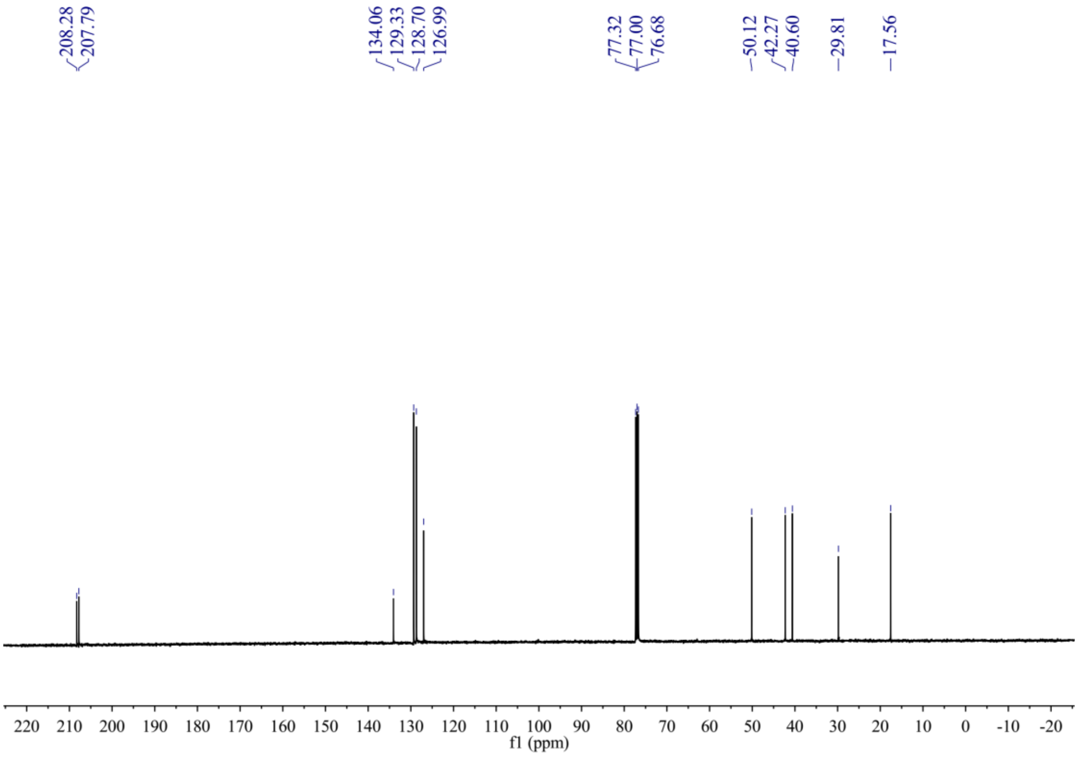

^{13}C NMR

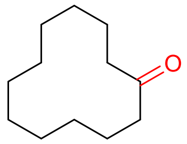

18

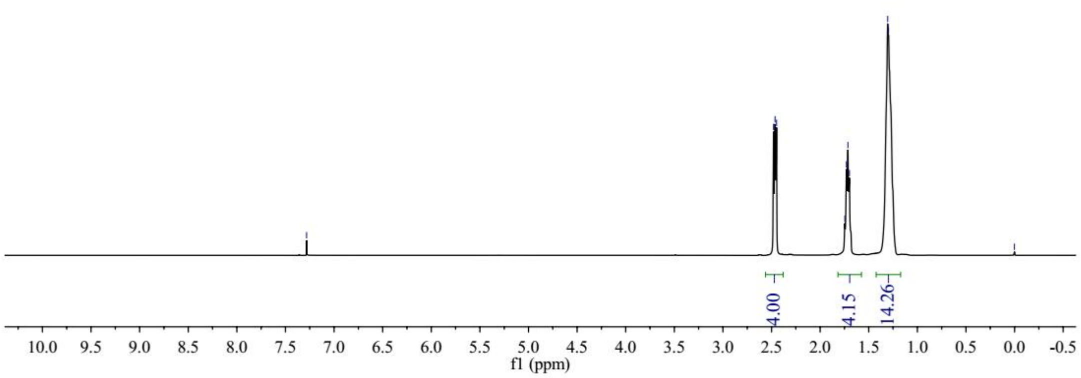

^{1}H NMR

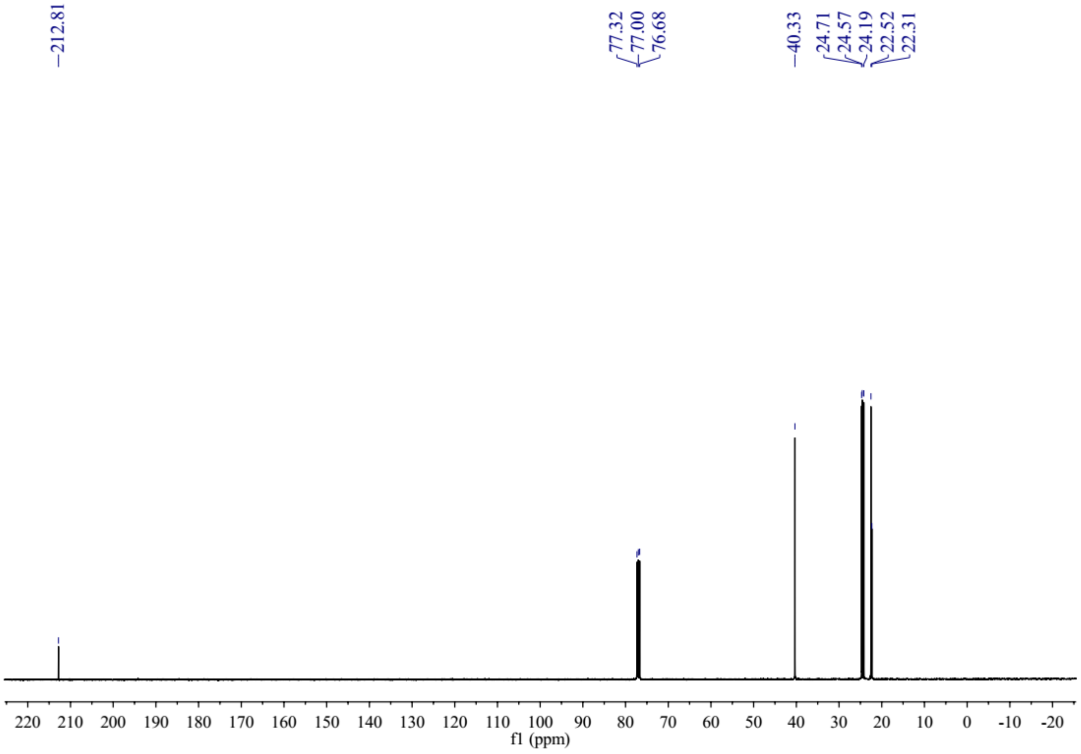

^{13}C NMR

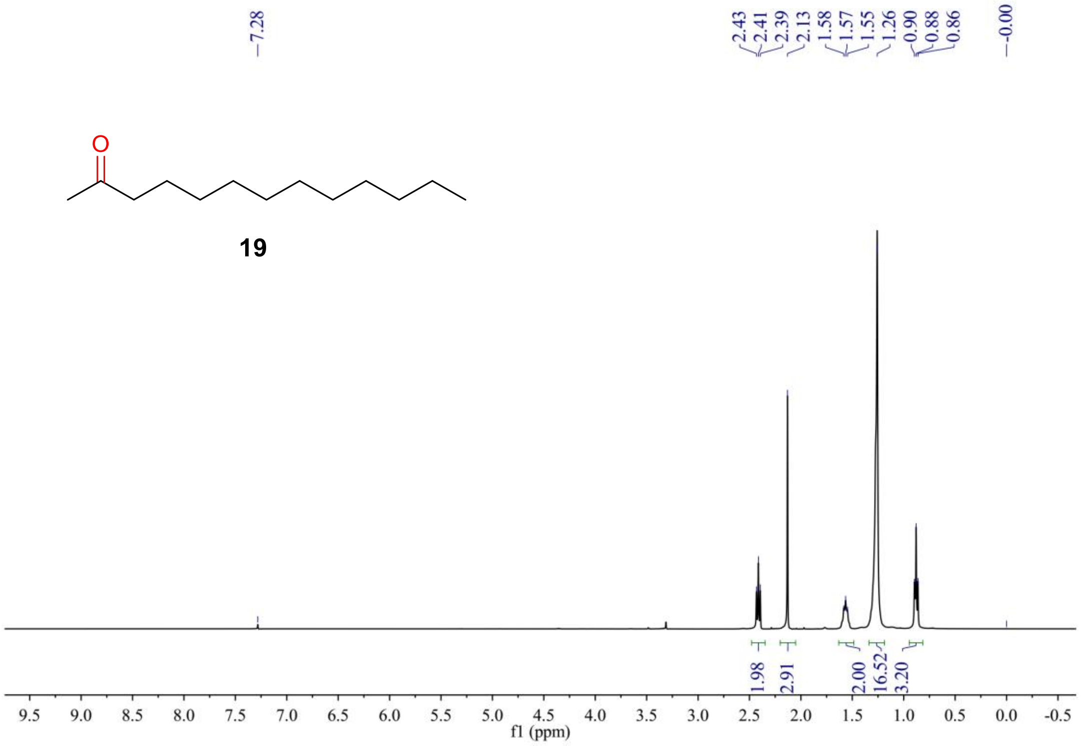

^{1}H NMR

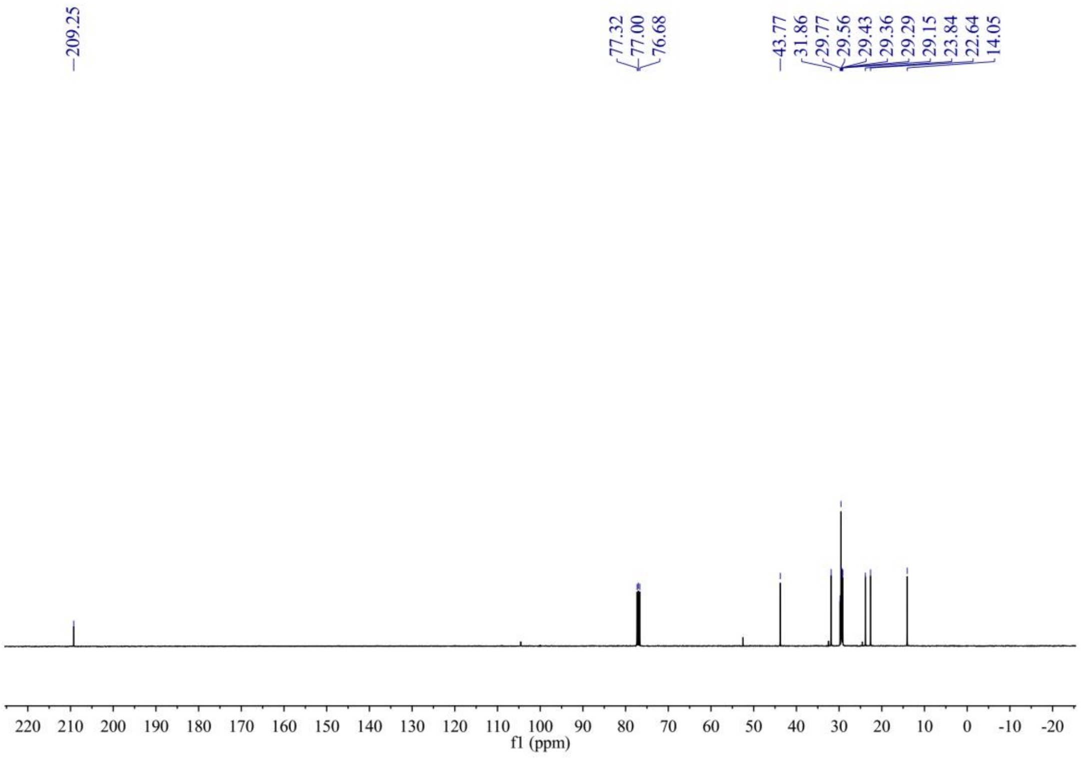

^{13}C NMR

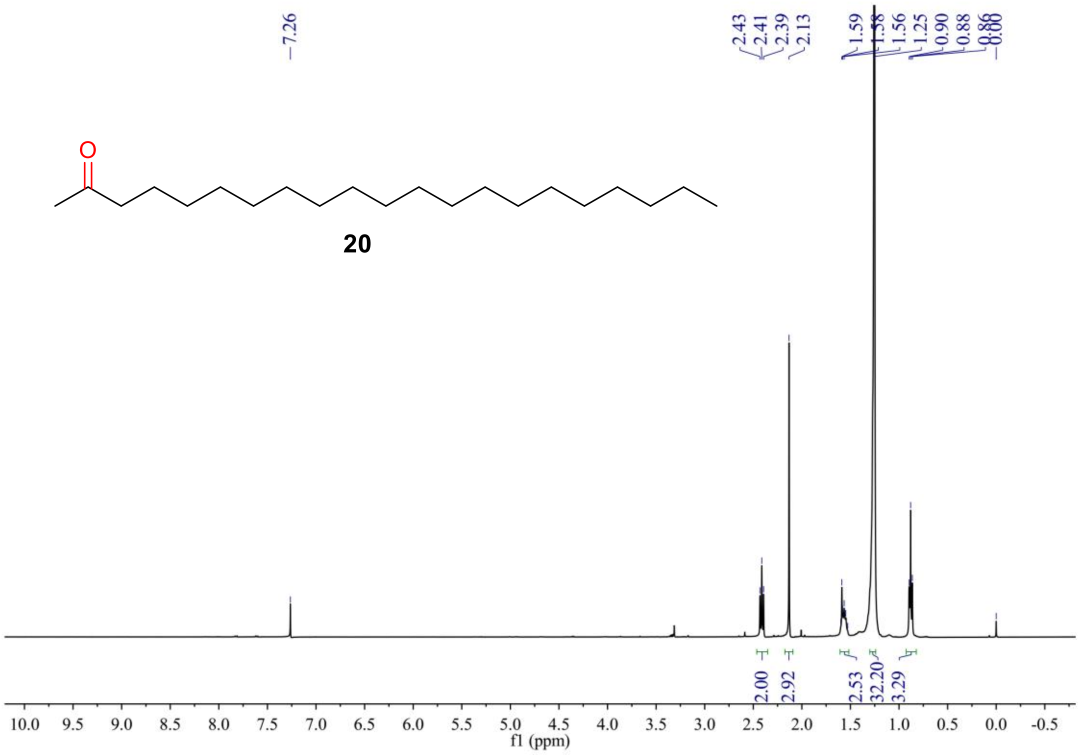

^{1}H NMR

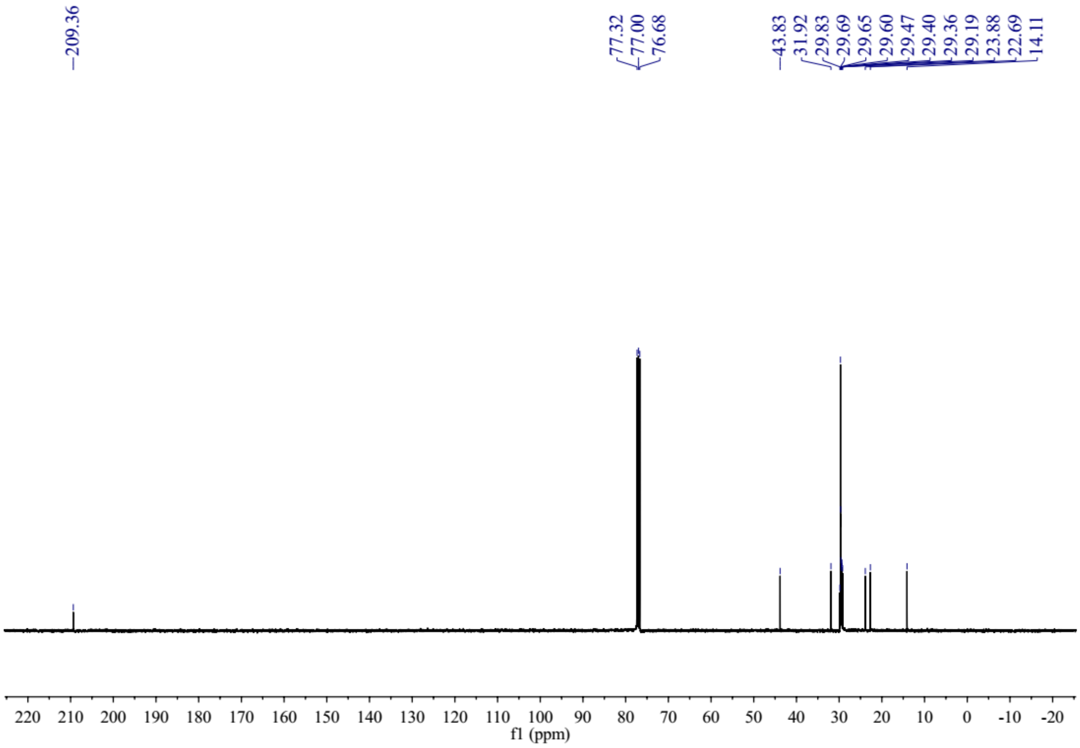

^{13}C NMR

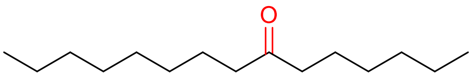

21

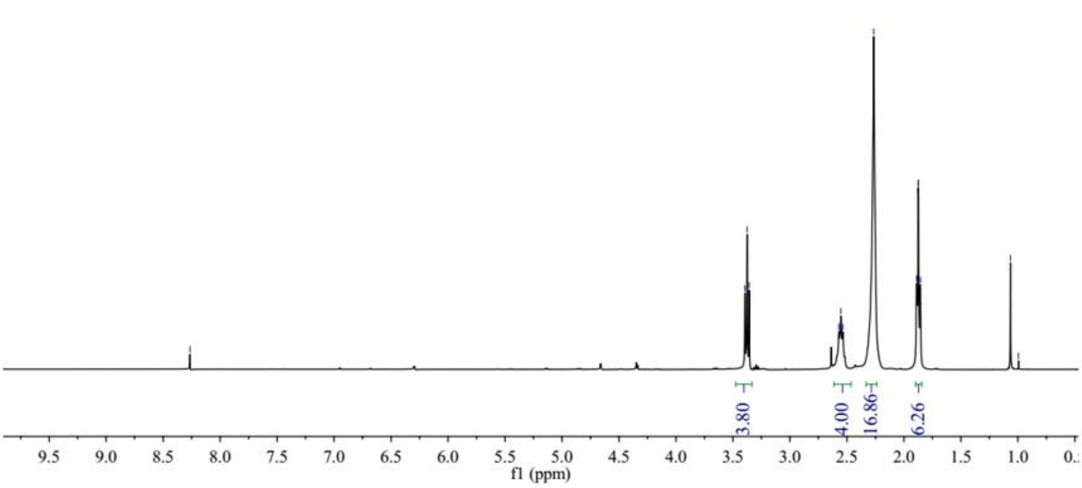

^{1}H NMR

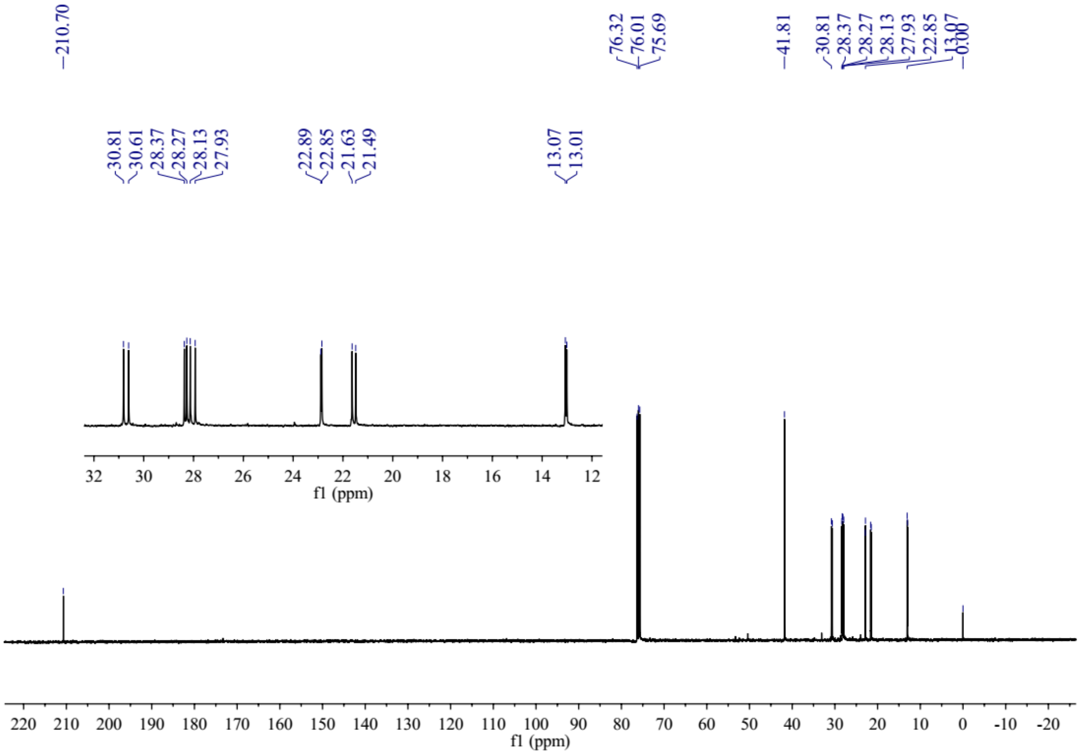

^{13}C NMR

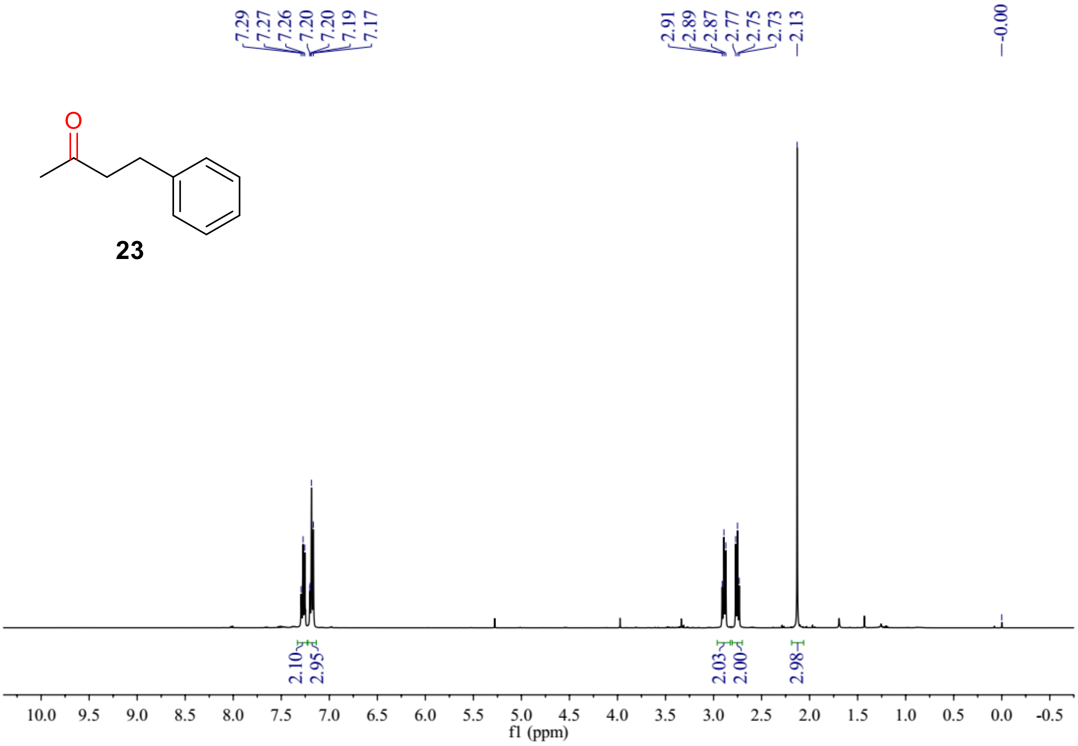

^{1}H NMR

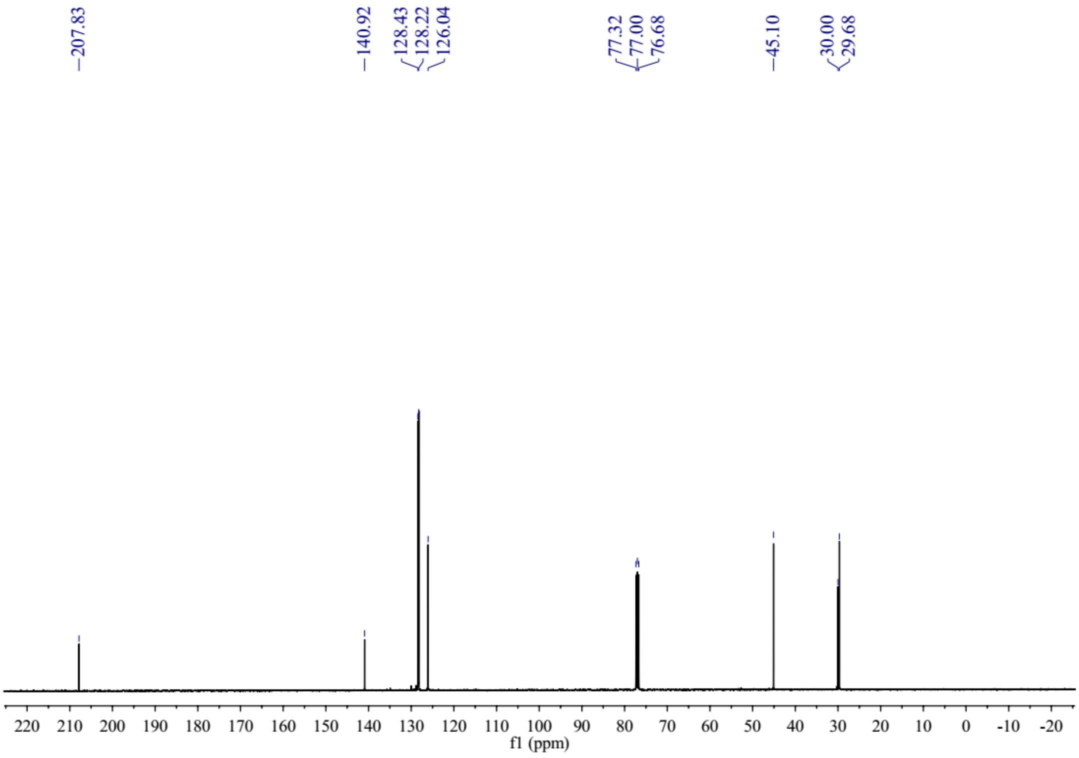

^{13}C NMR

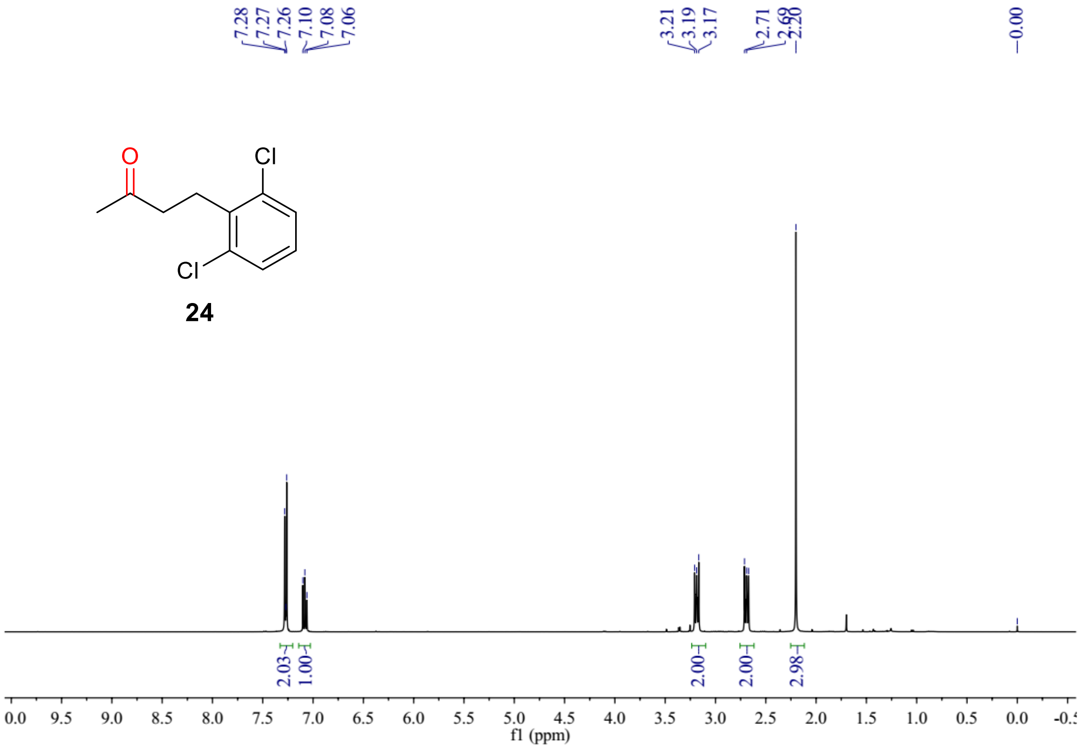

^{1}H NMR

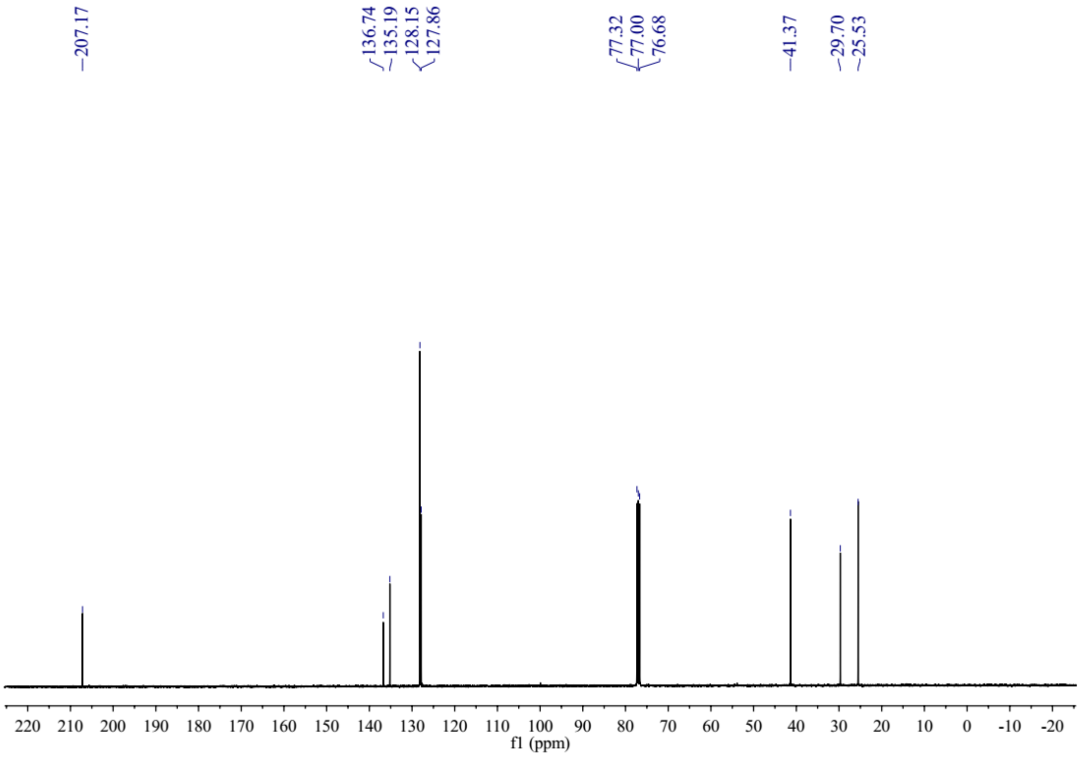

^{13}C NMR

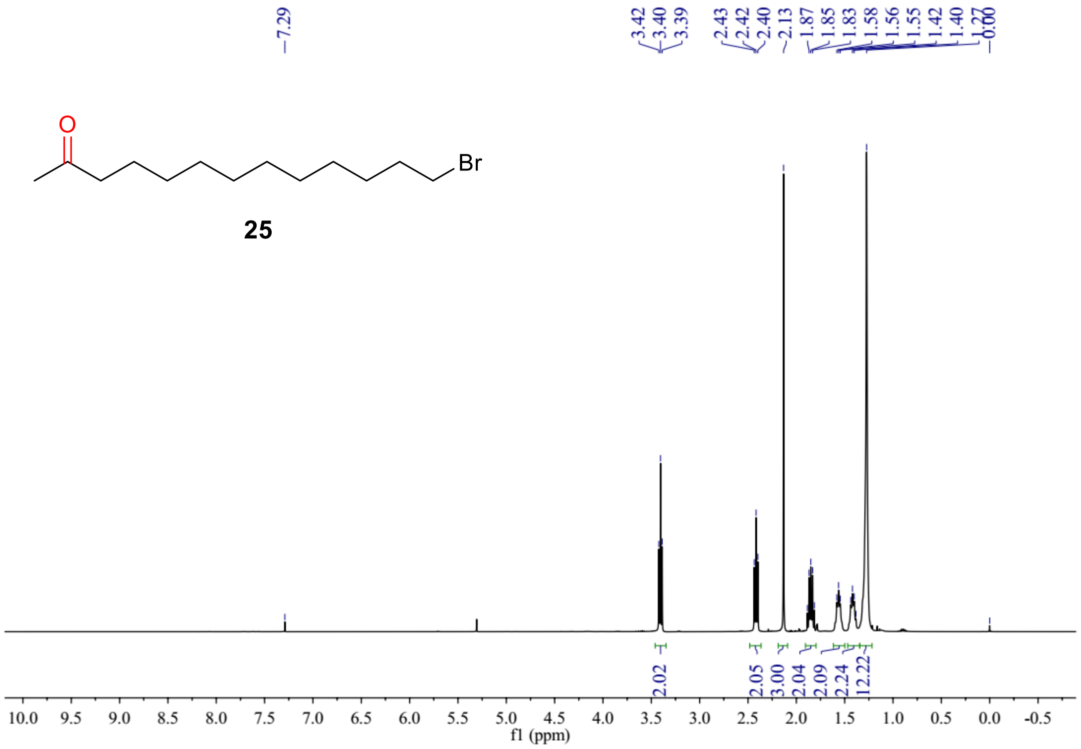

^{1}H NMR

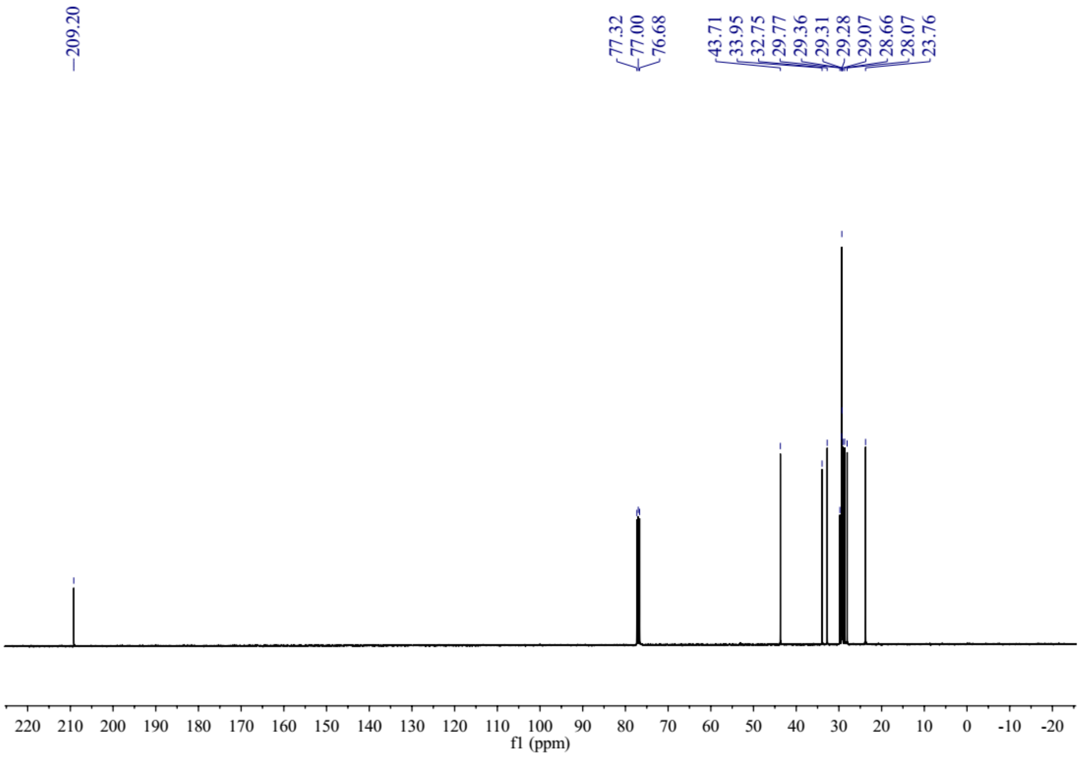

^{13}C NMR

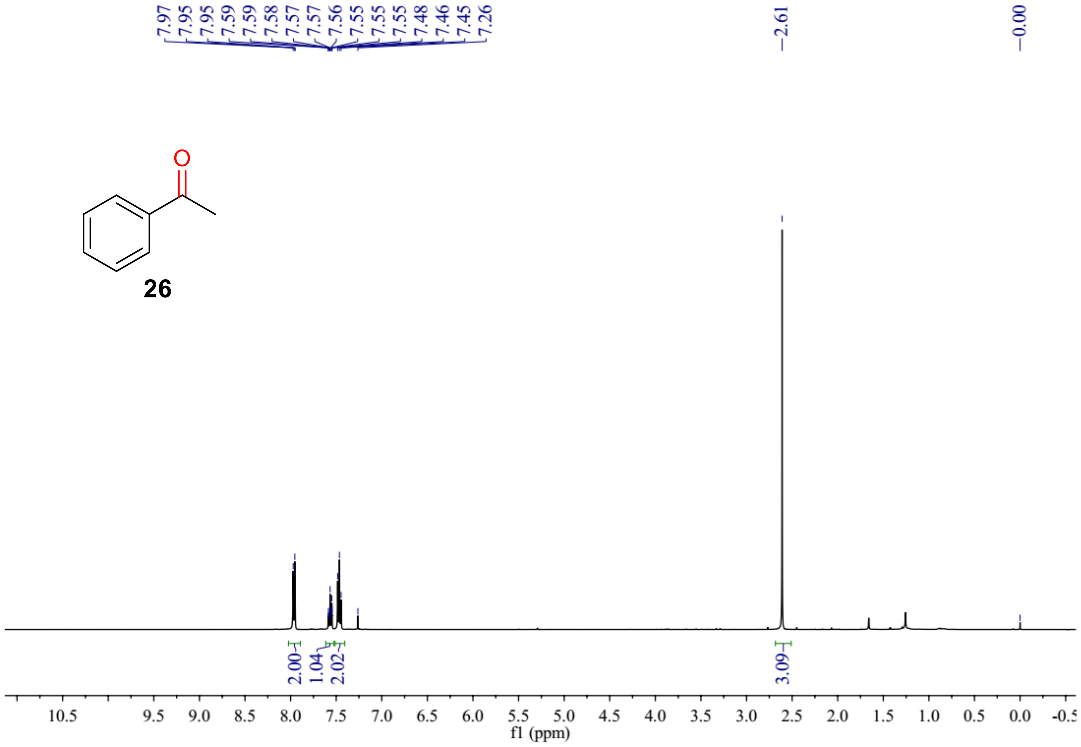

26

^{1}H NMR

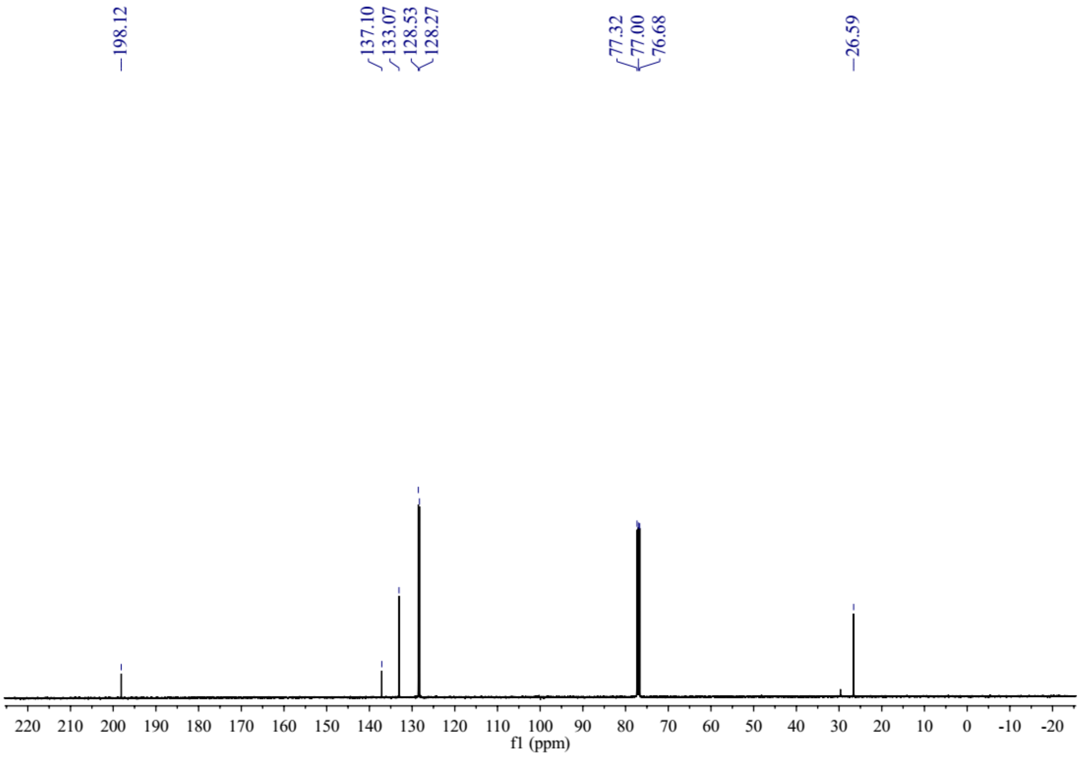

^{13}C NMR

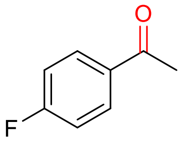

27

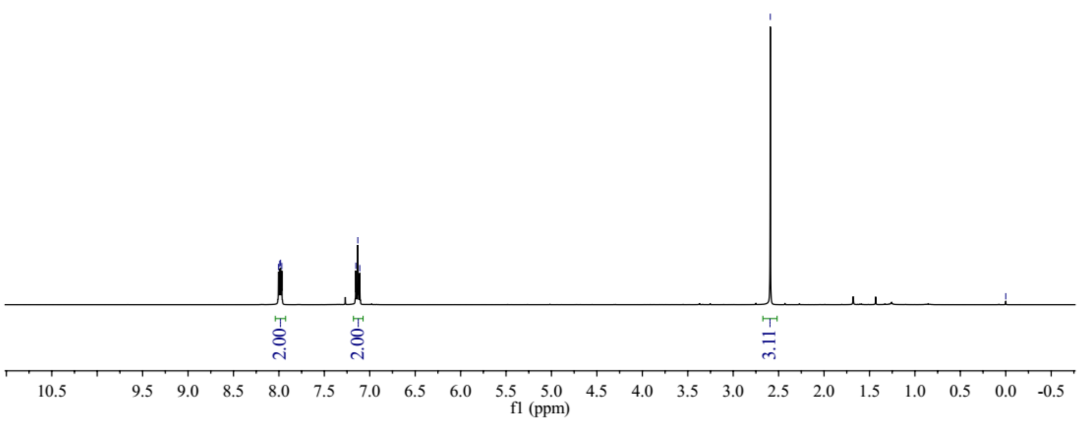

^{1}H NMR

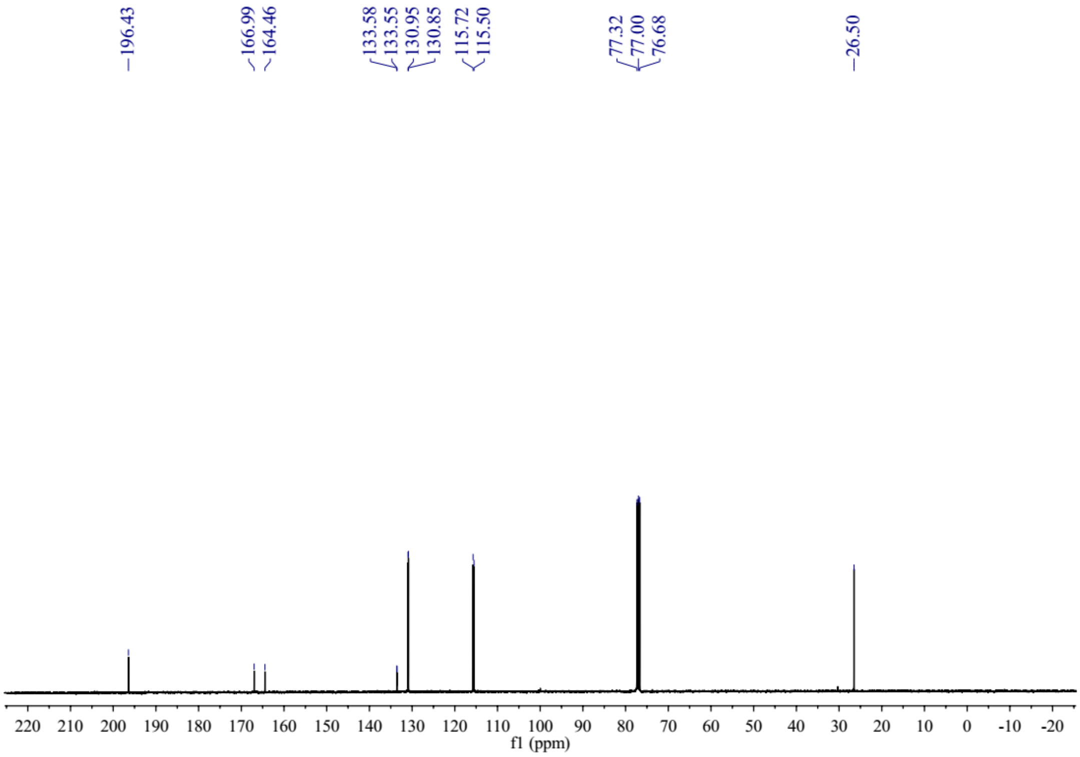

^{13}C NMR

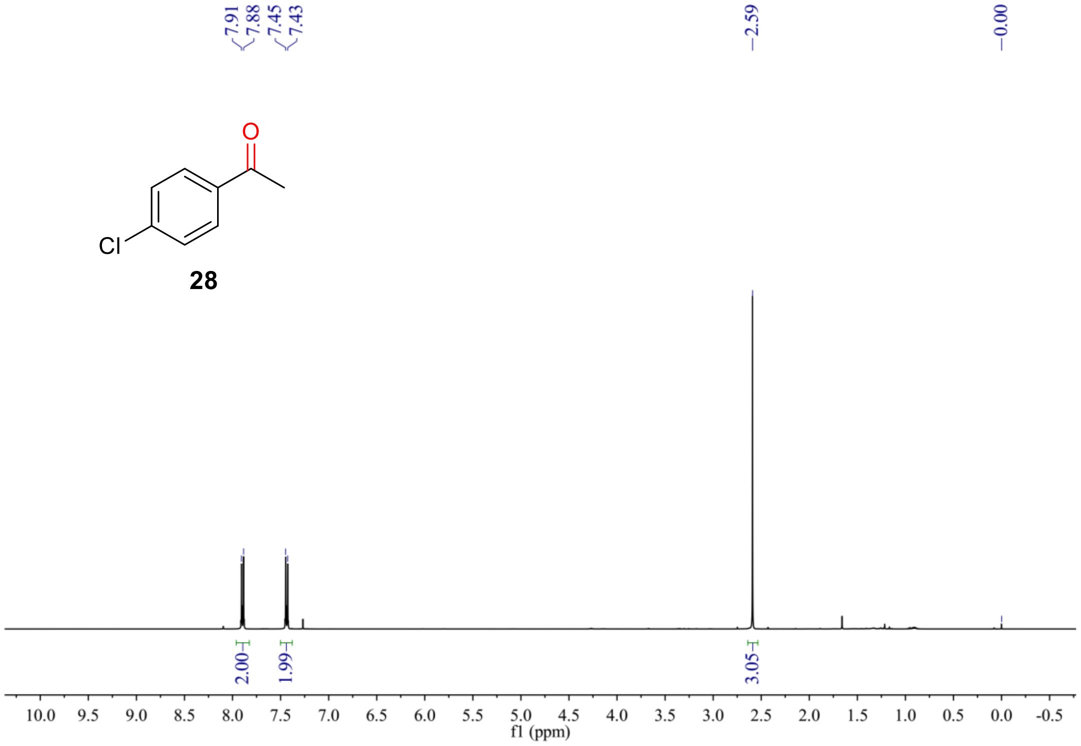

^{1}H NMR

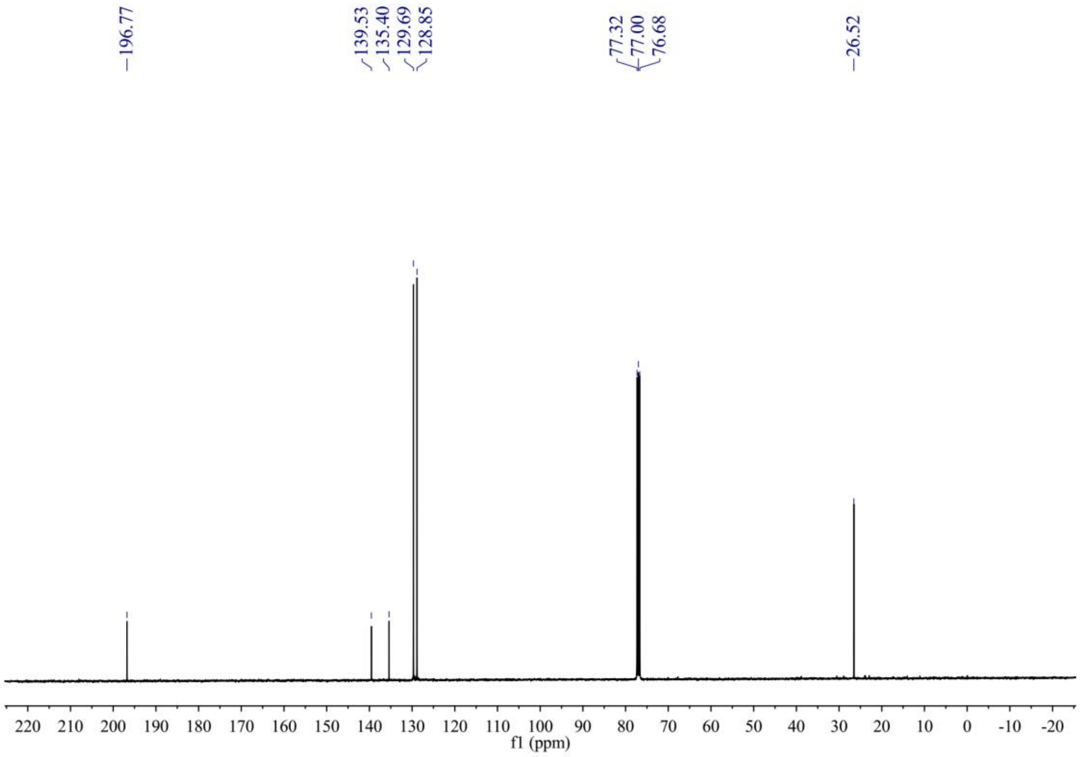

^{13}C NMR

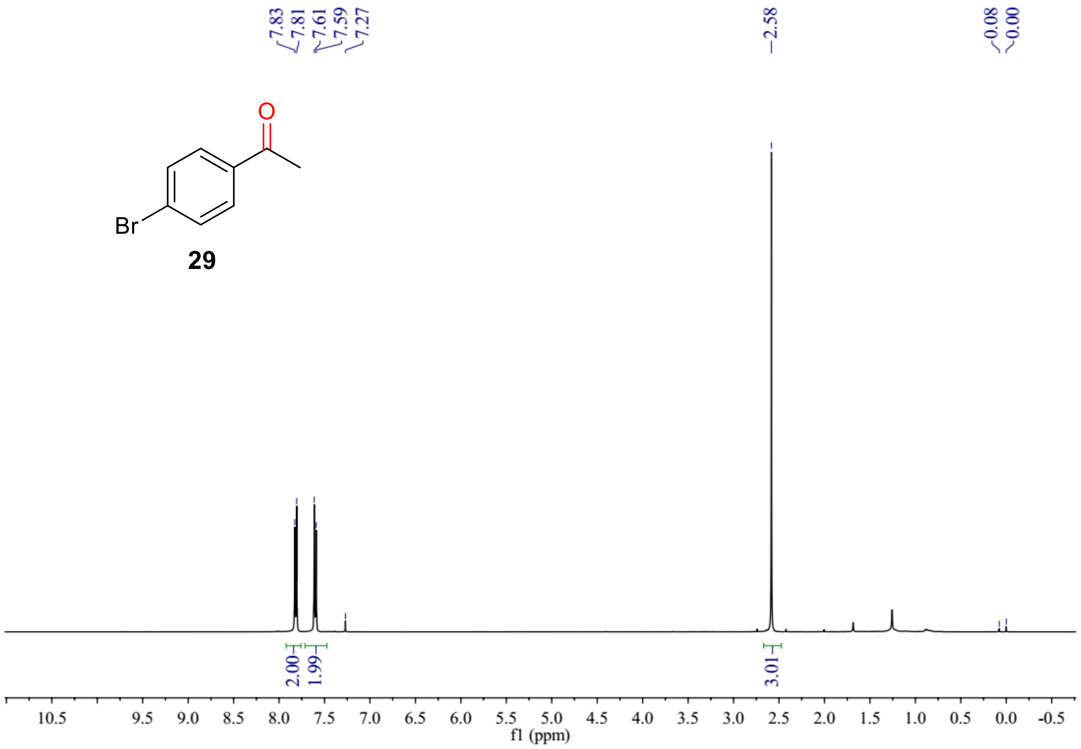

^{1}H NMR

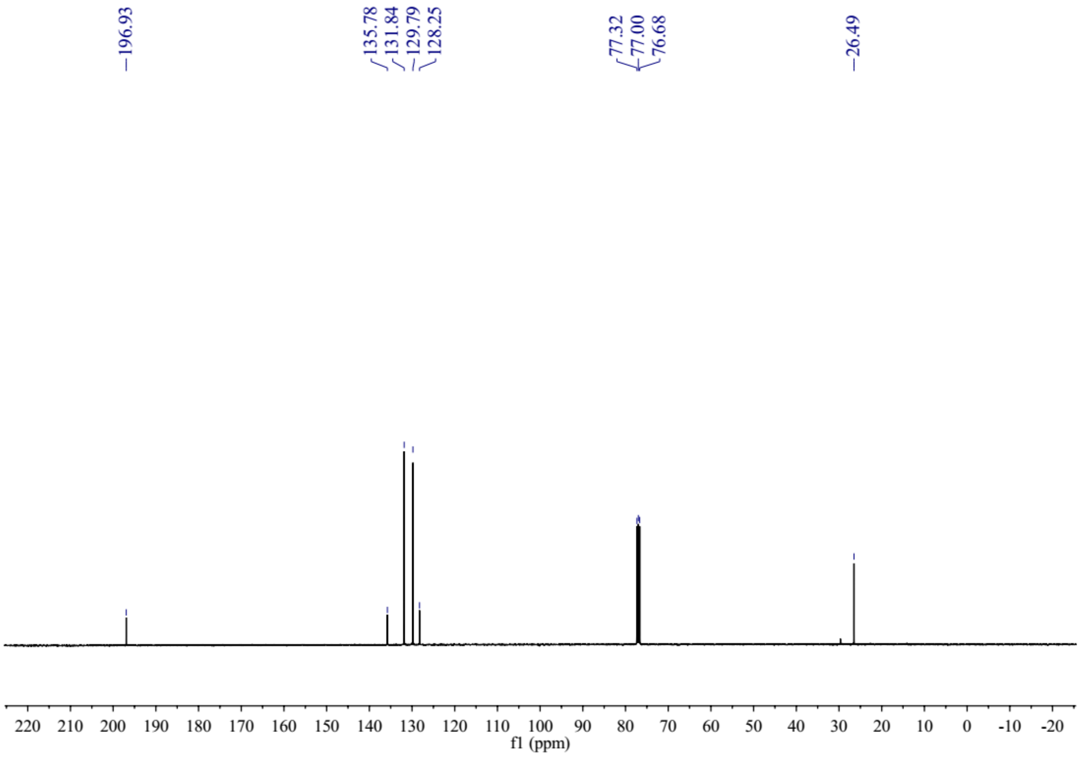

^{13}C NMR

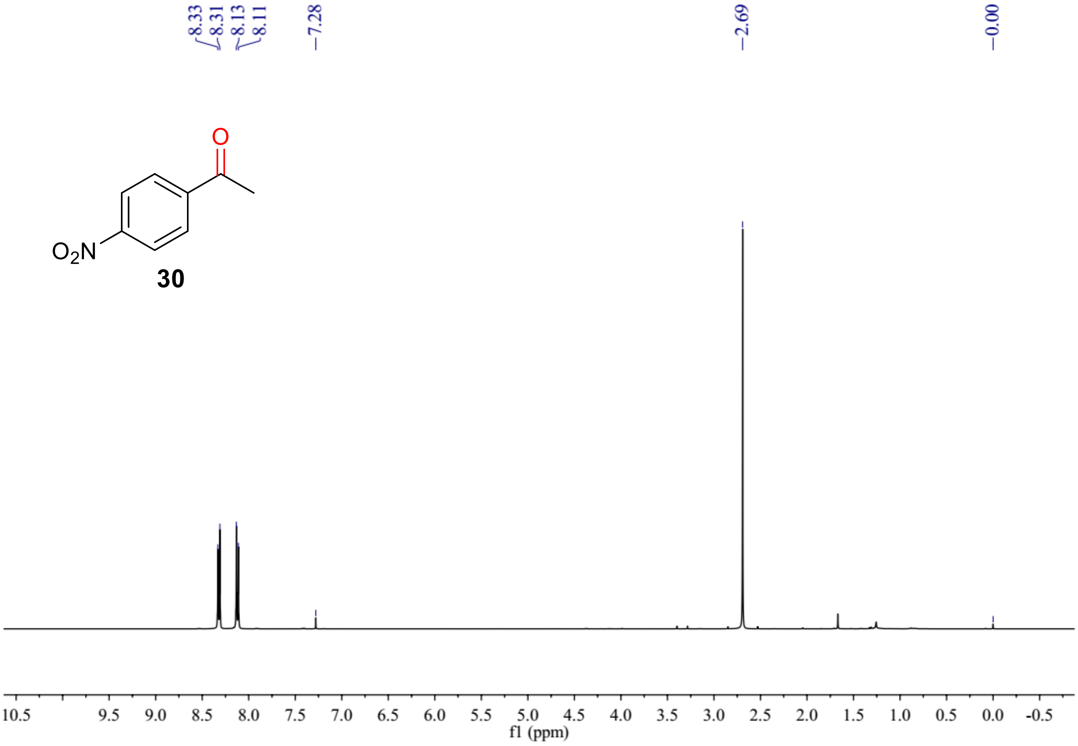

^{1}H NMR

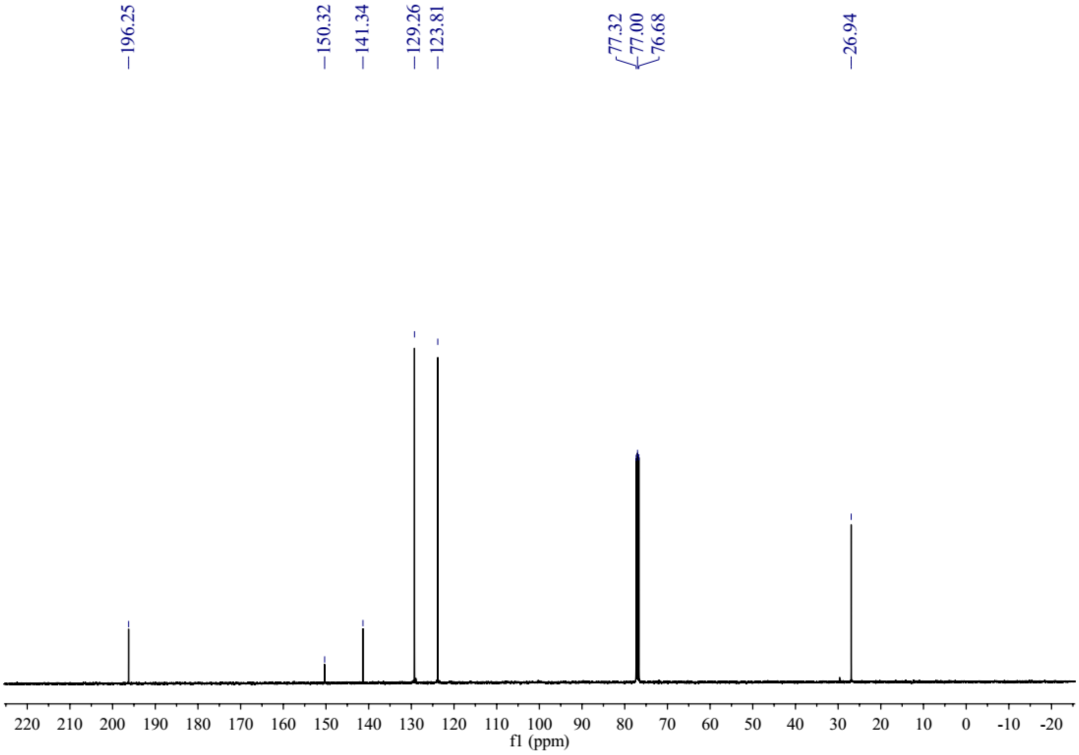

^{13}C NMR

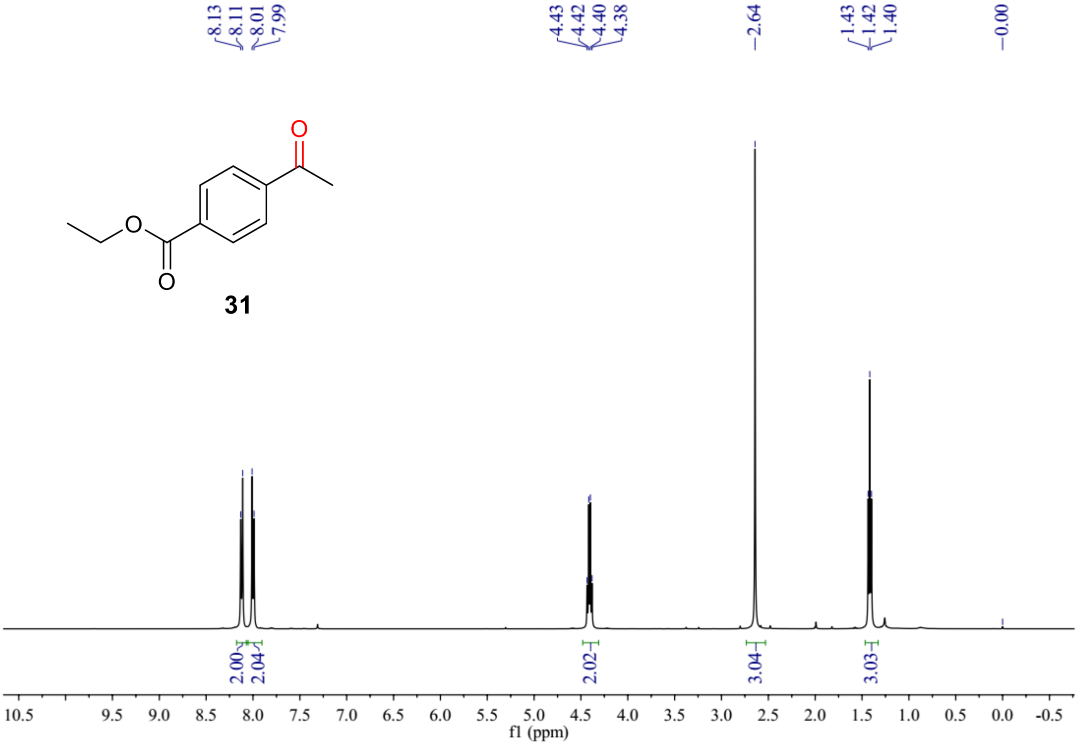

^{1}H NMR

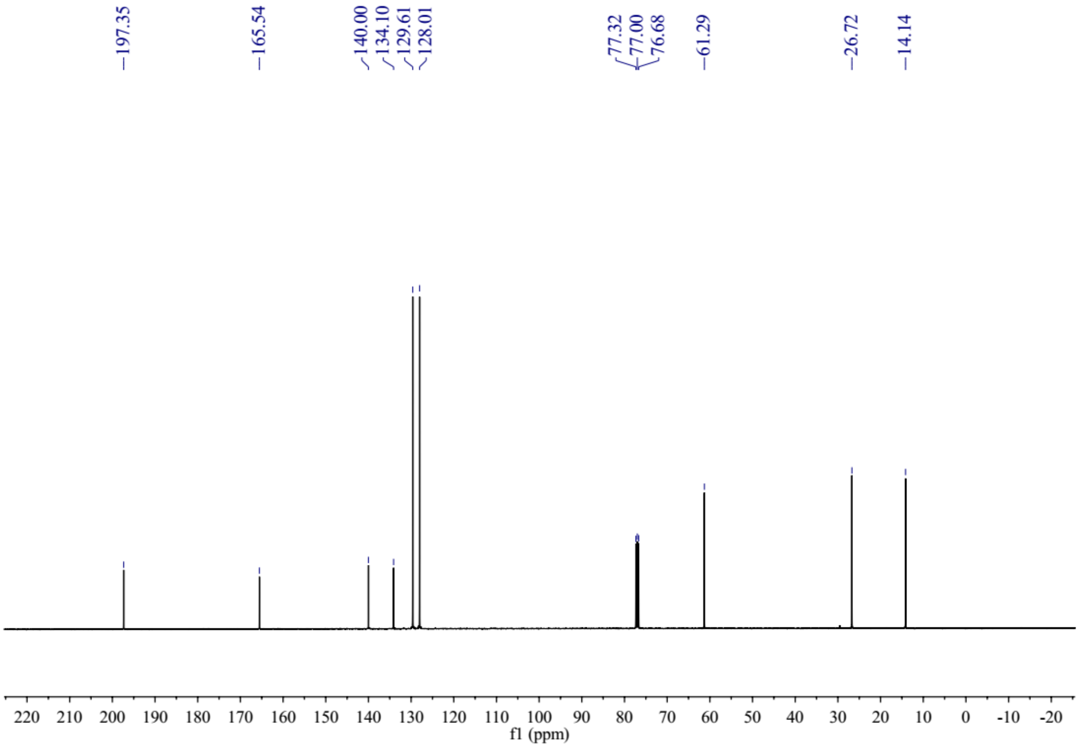

^{13}C NMR

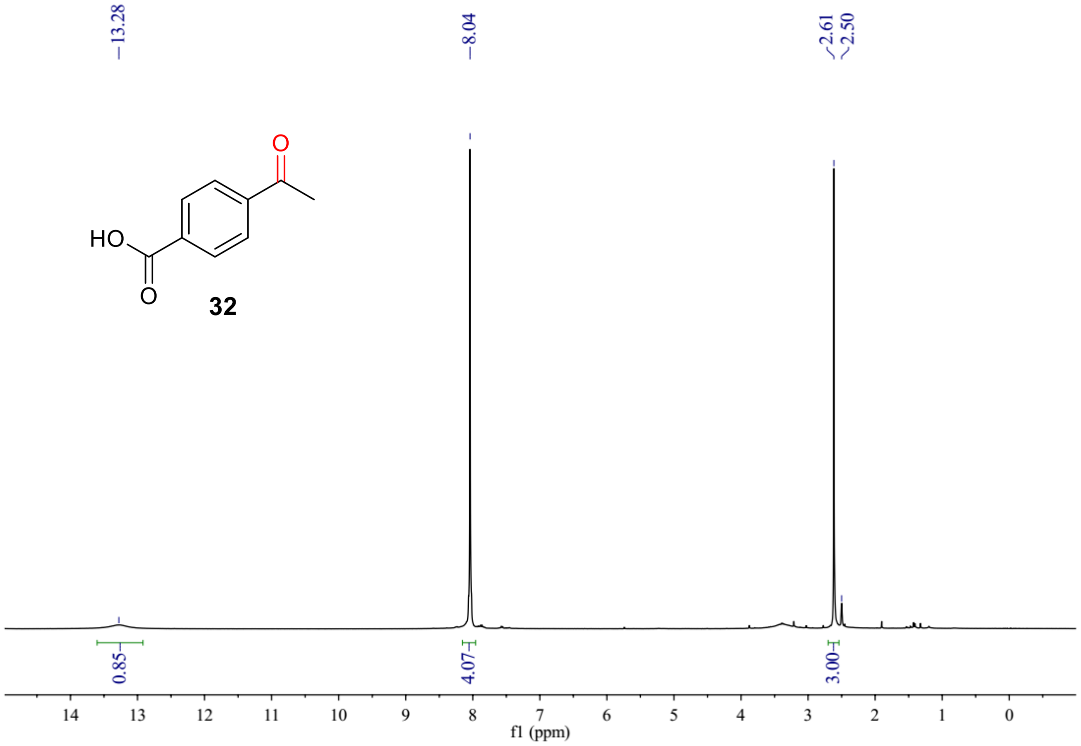

^{1}H NMR

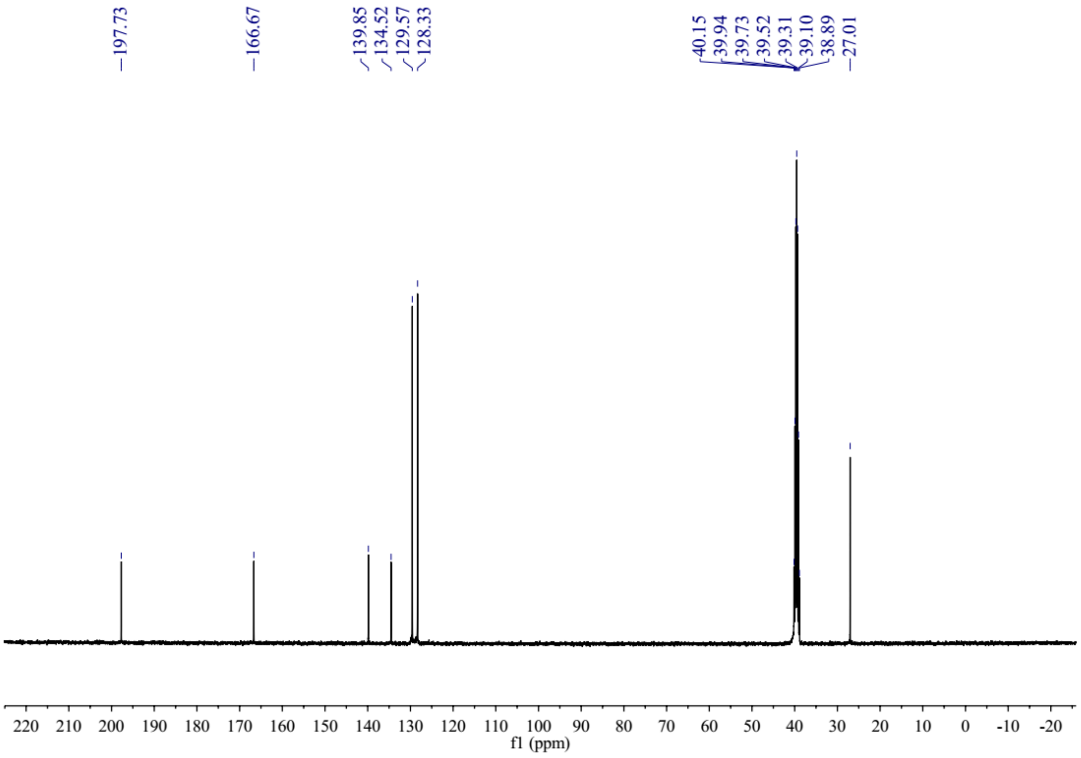

^{13}C NMR

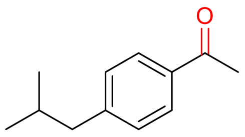

33

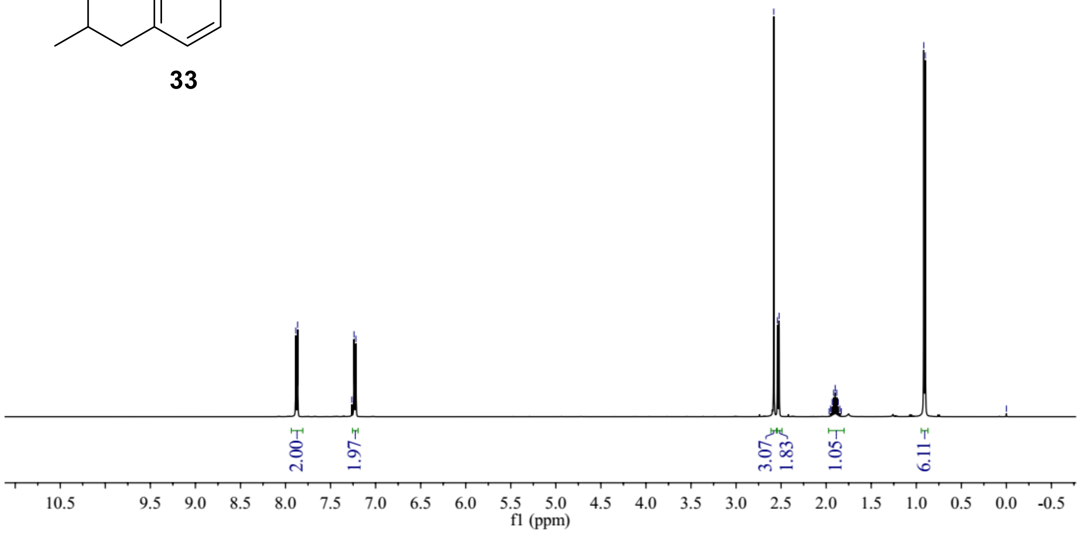

^{1}H NMR

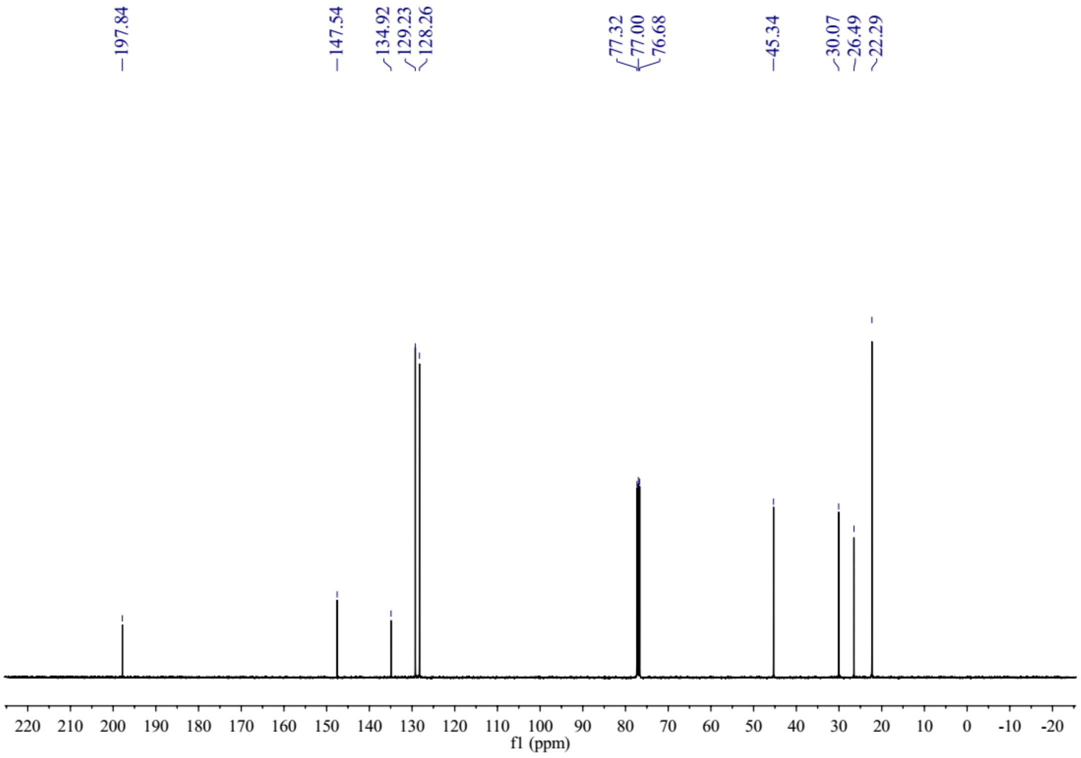

^{13}C NMR

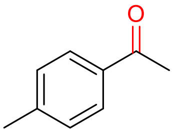

34

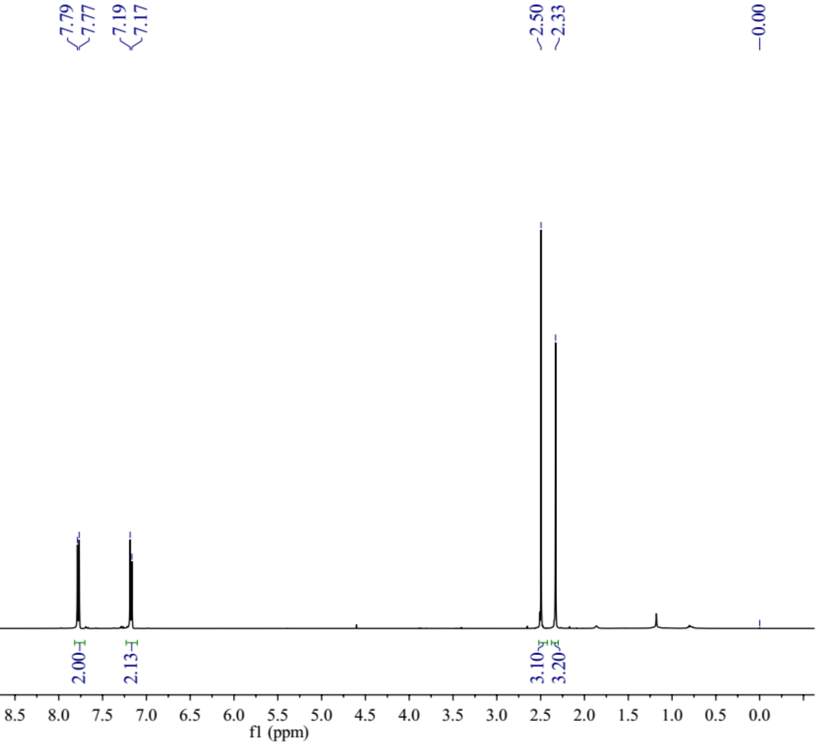

^{1}H NMR

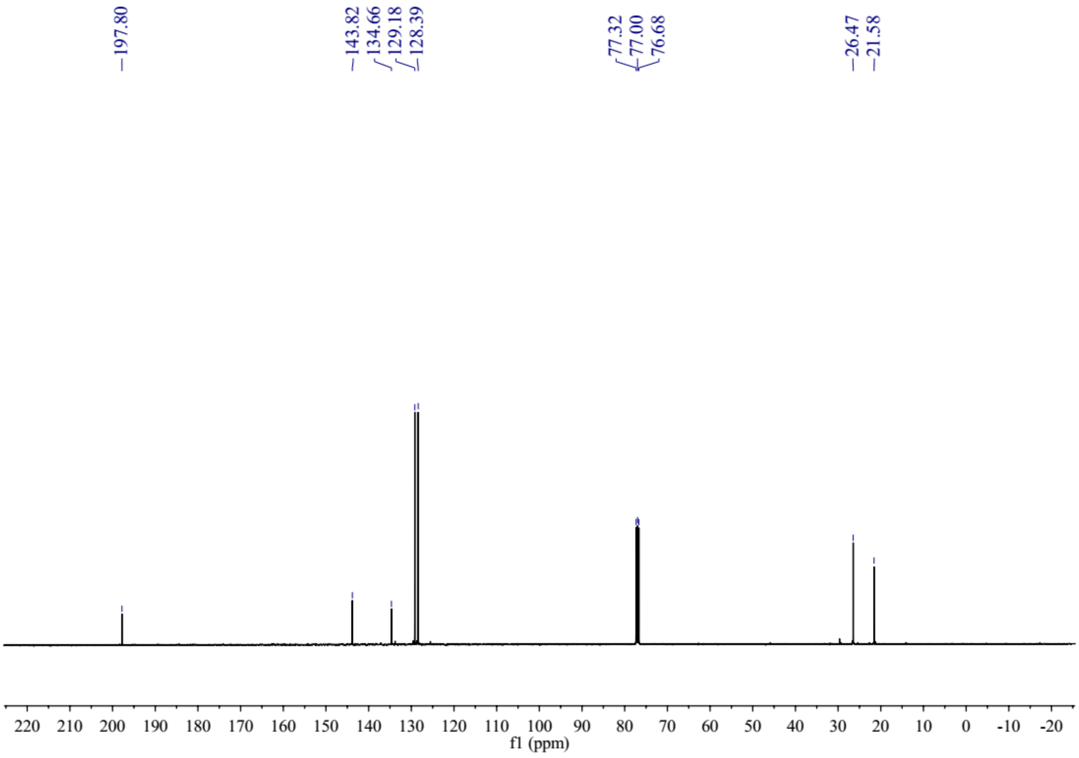

^{13}C NMR

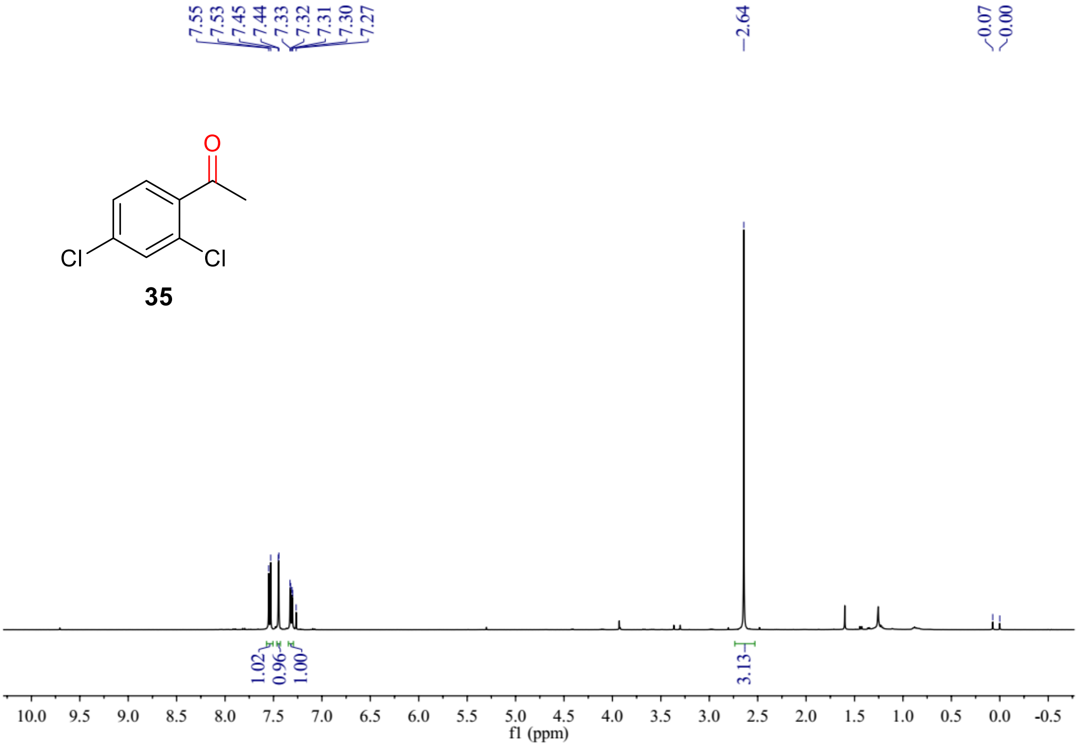

^{1}H NMR

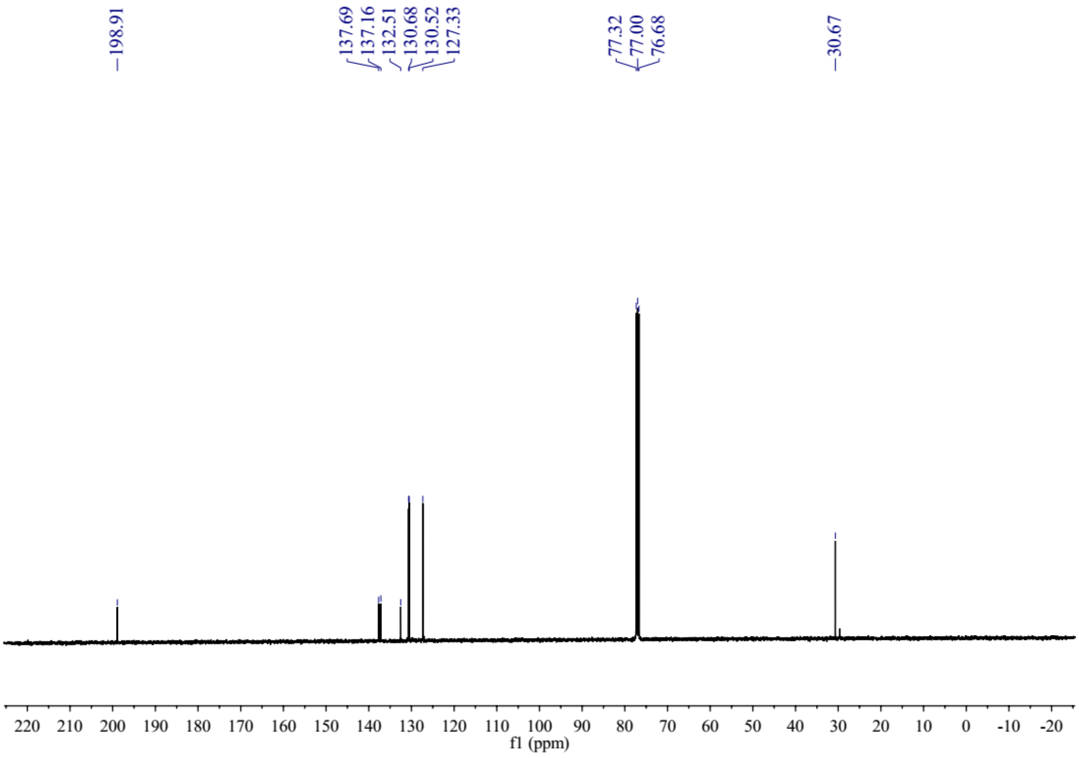

^{13}C NMR

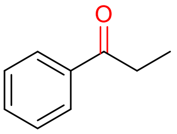

36

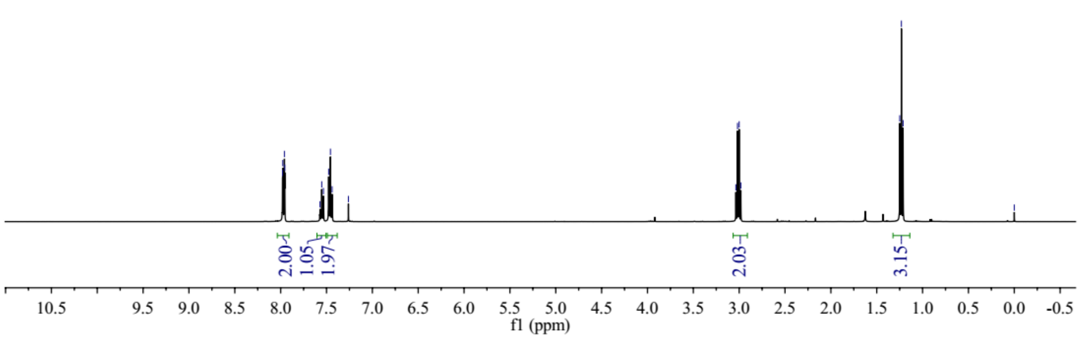

^{1}H NMR

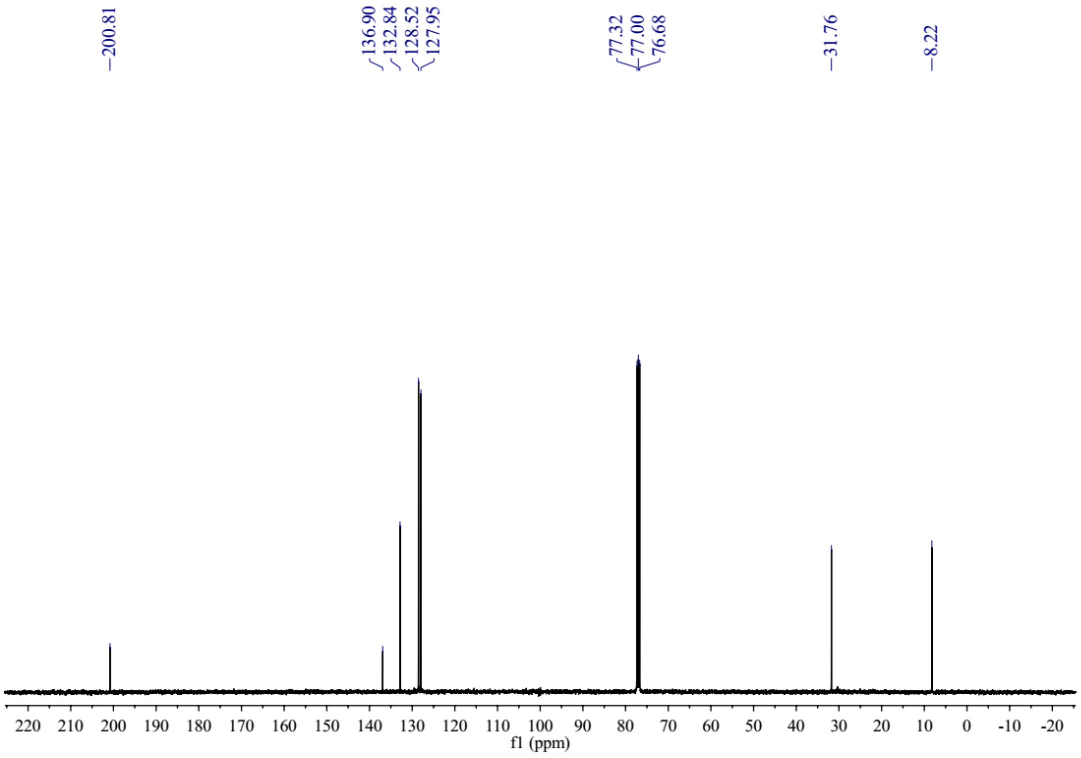

^{13}C NMR

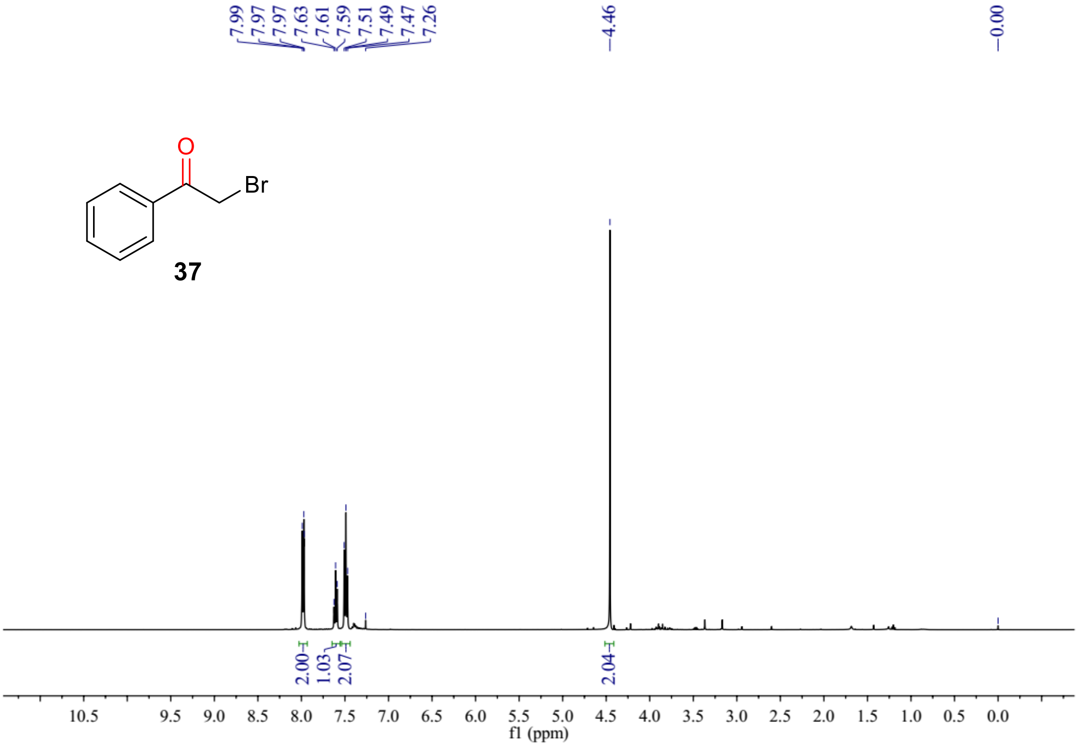

^{1}H NMR

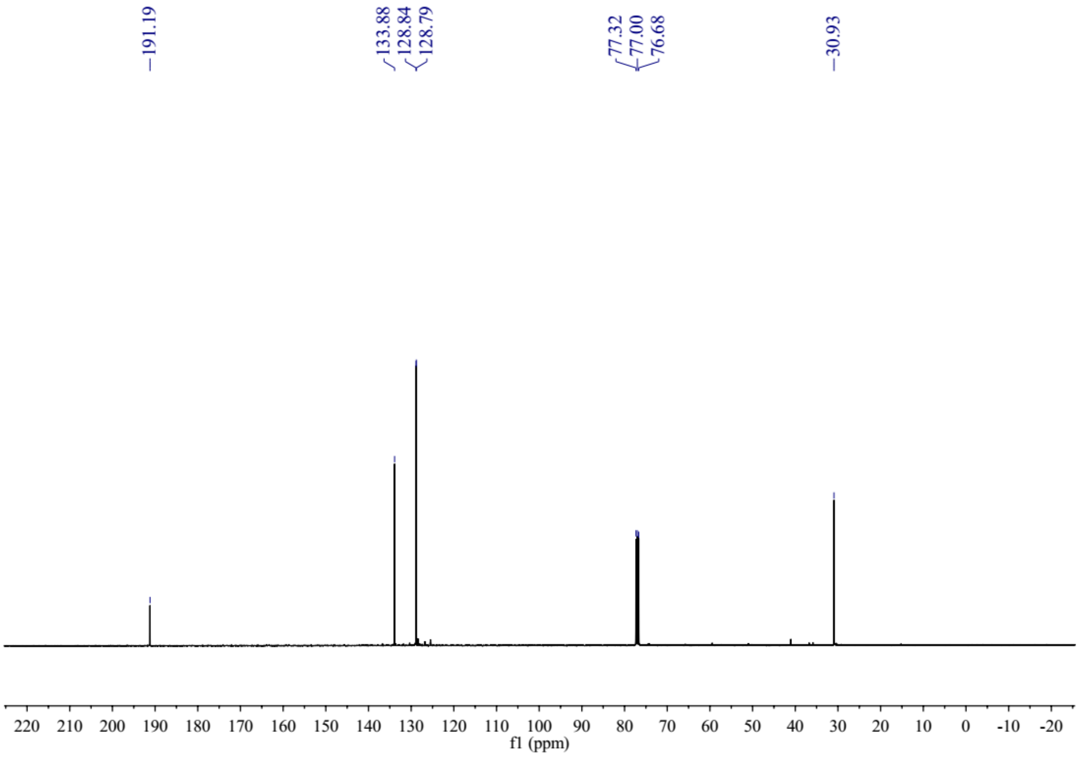

^{13}C NMR

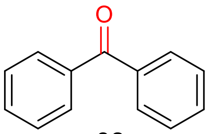

38

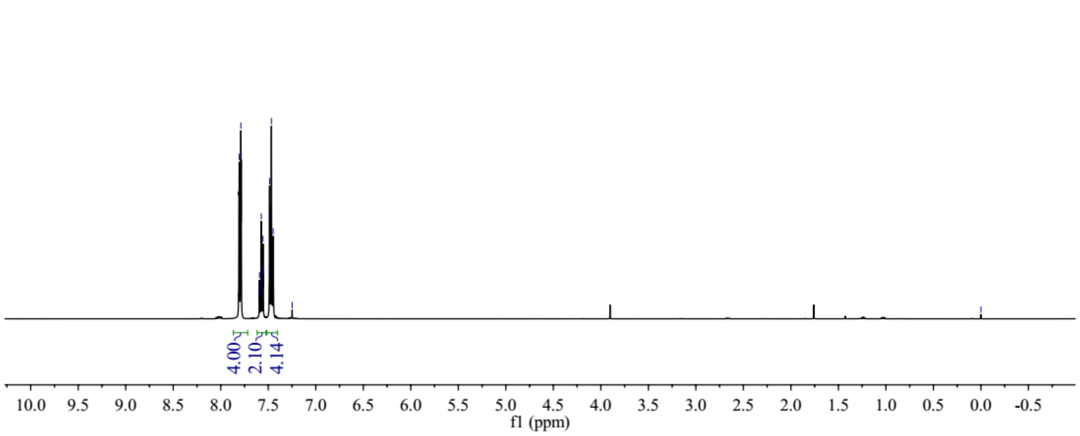

^{1}H NMR

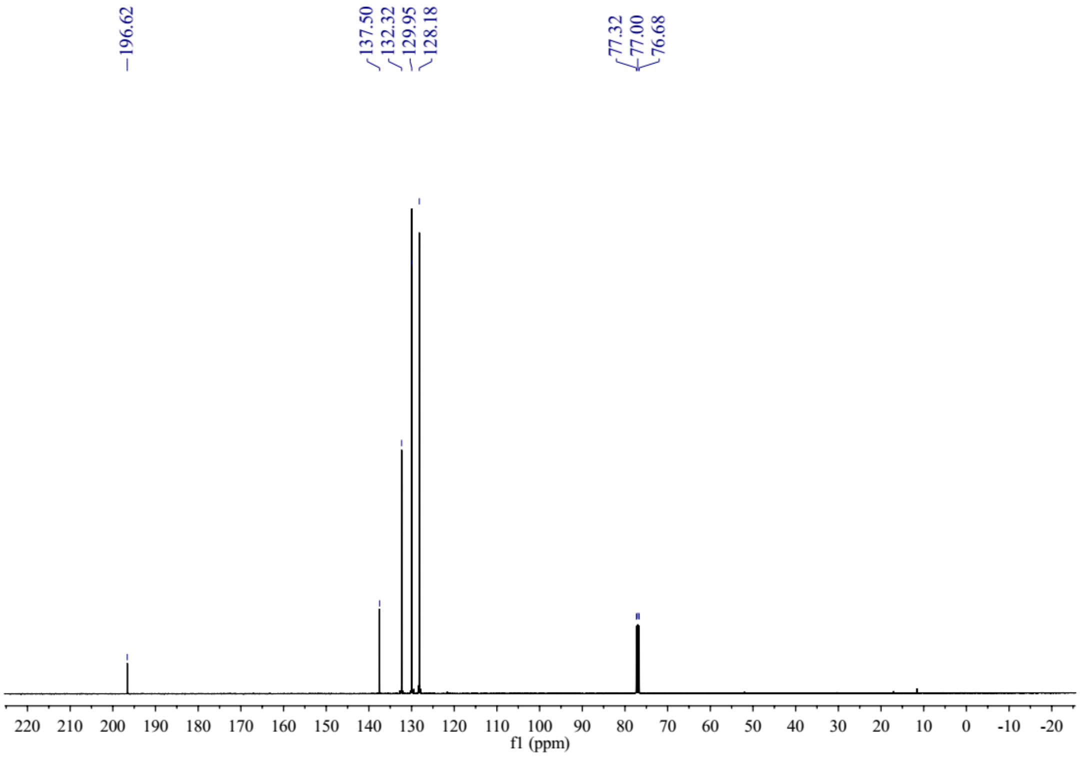

^{13}C NMR

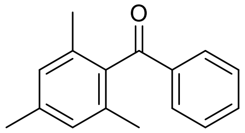

39

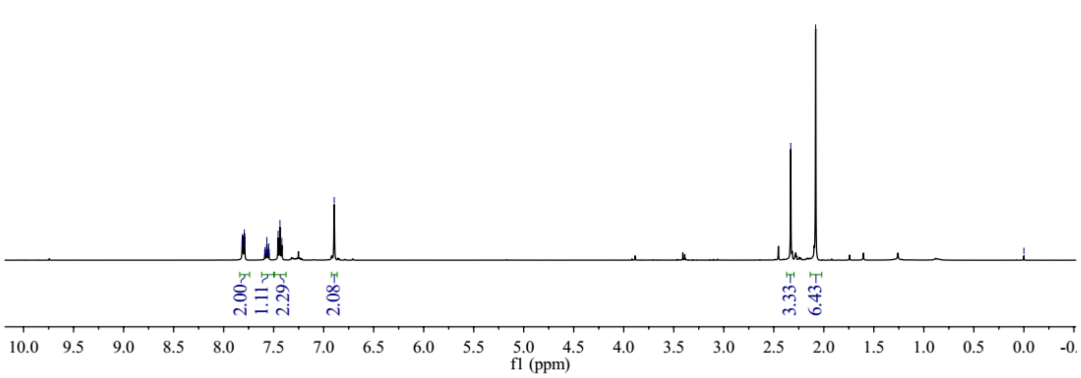

^{1}H NMR

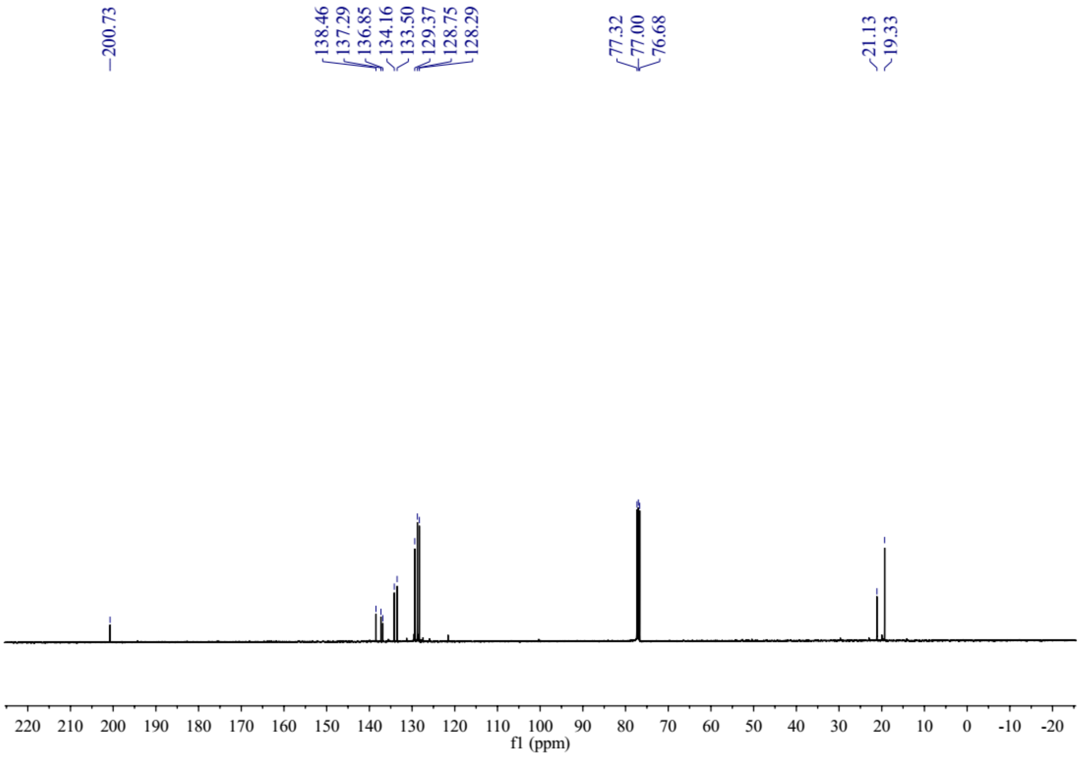

^{13}C NMR

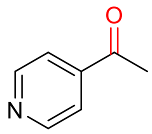

40

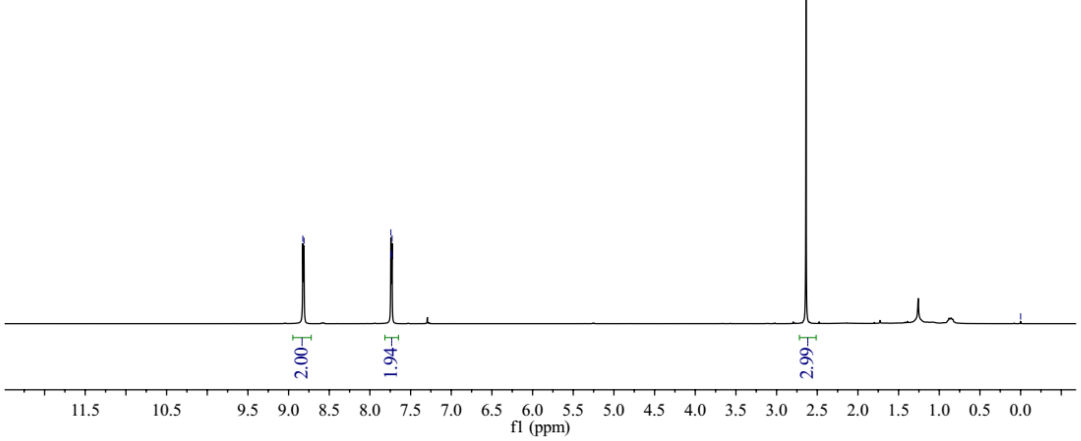

^{1}H NMR

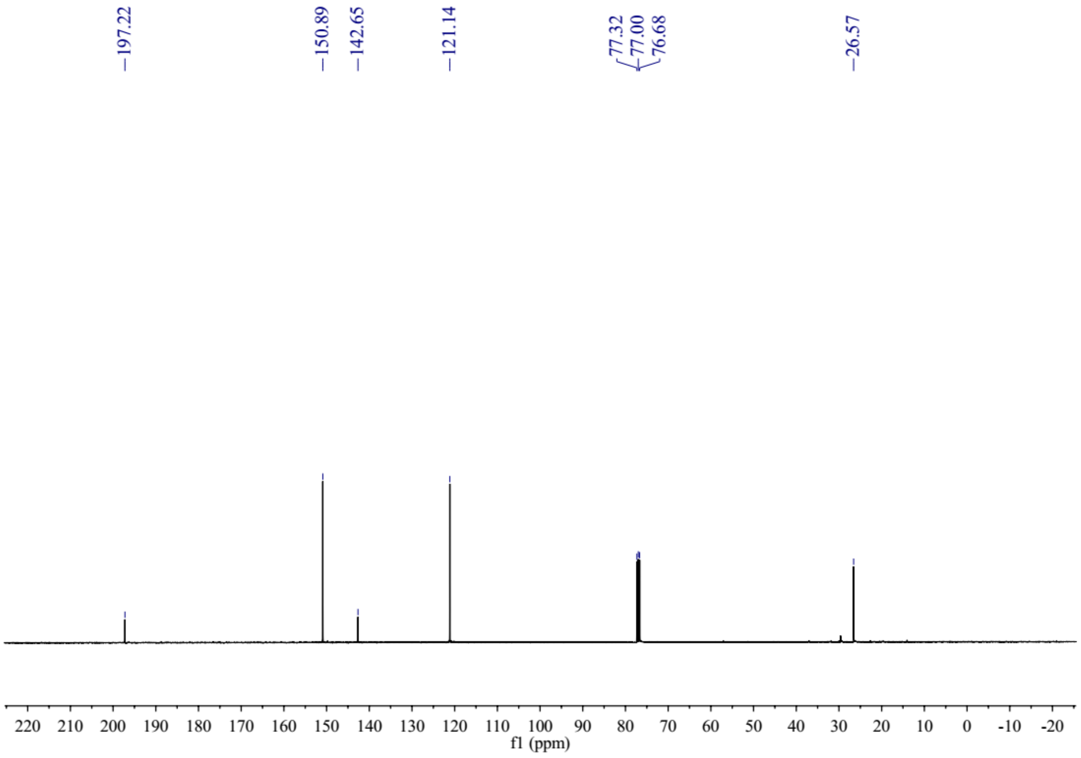

^{13}C NMR

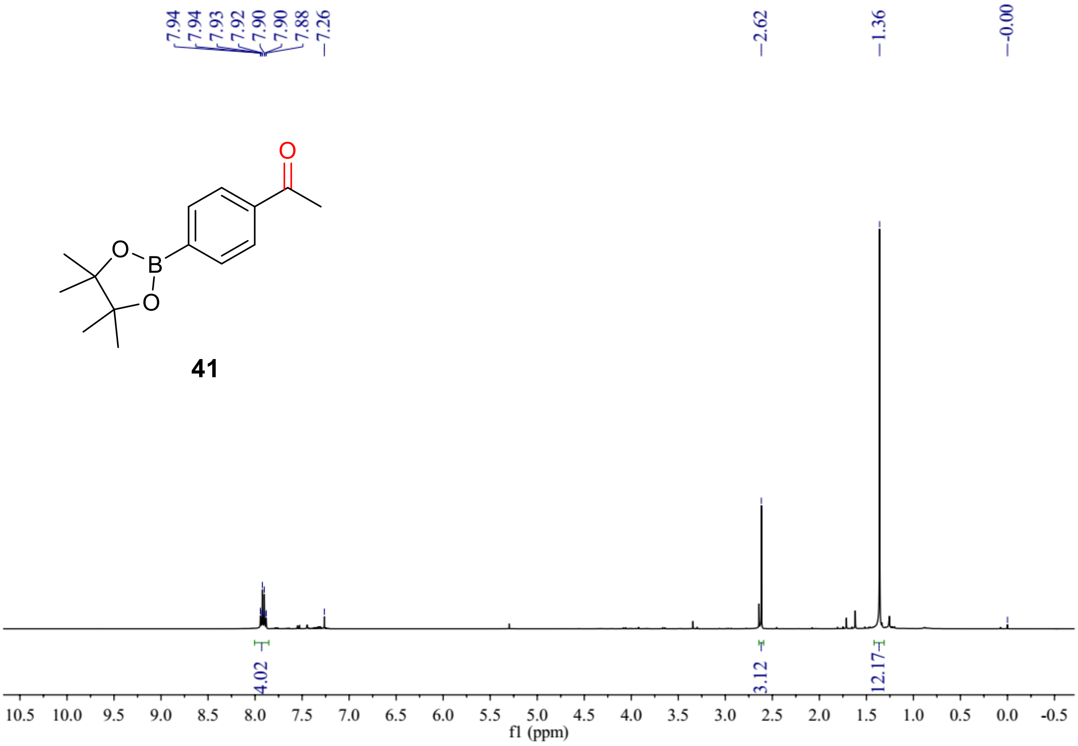

^{1}H NMR

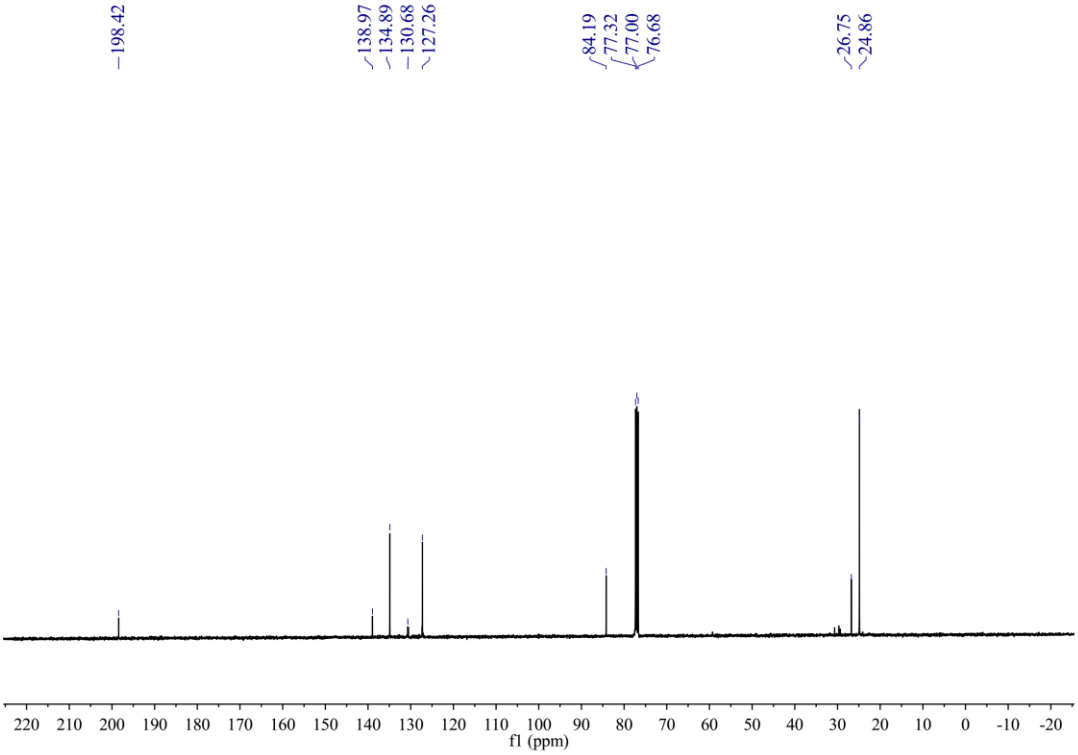

^{13}C NMR

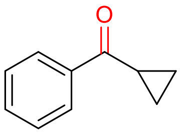

42

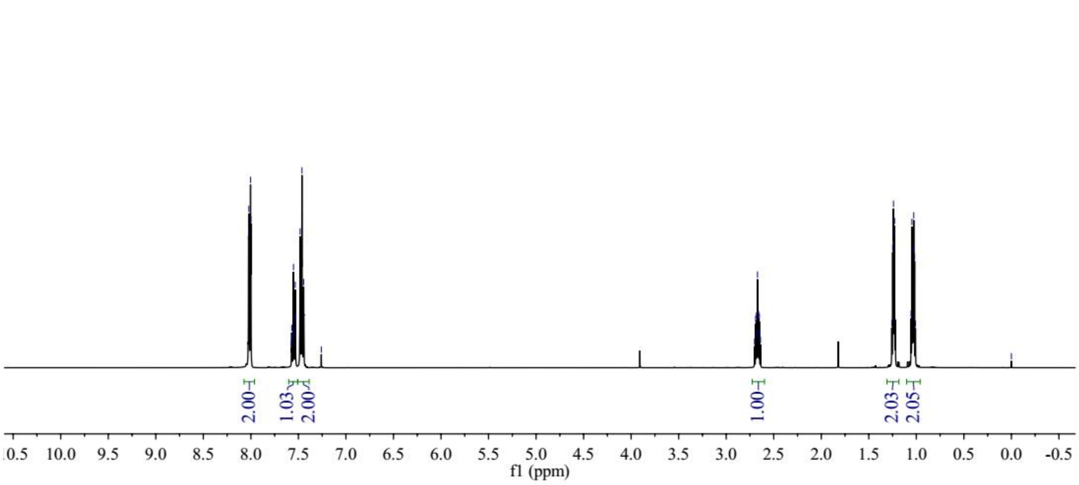

^{1}H NMR

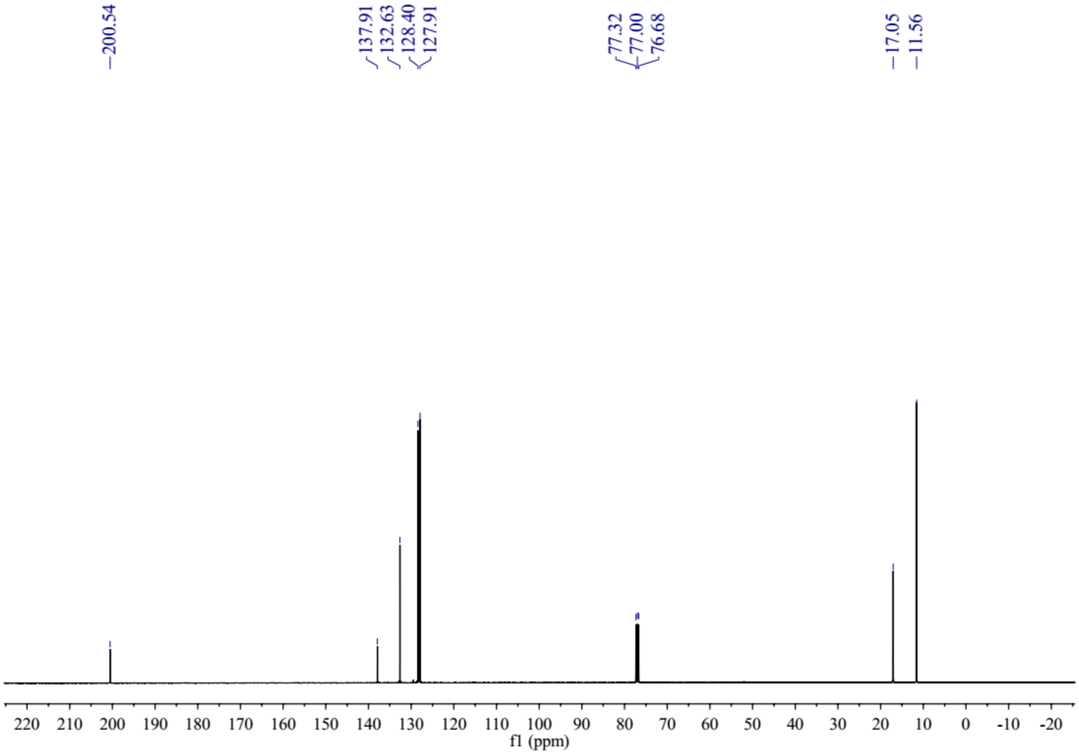

^{13}C NMR

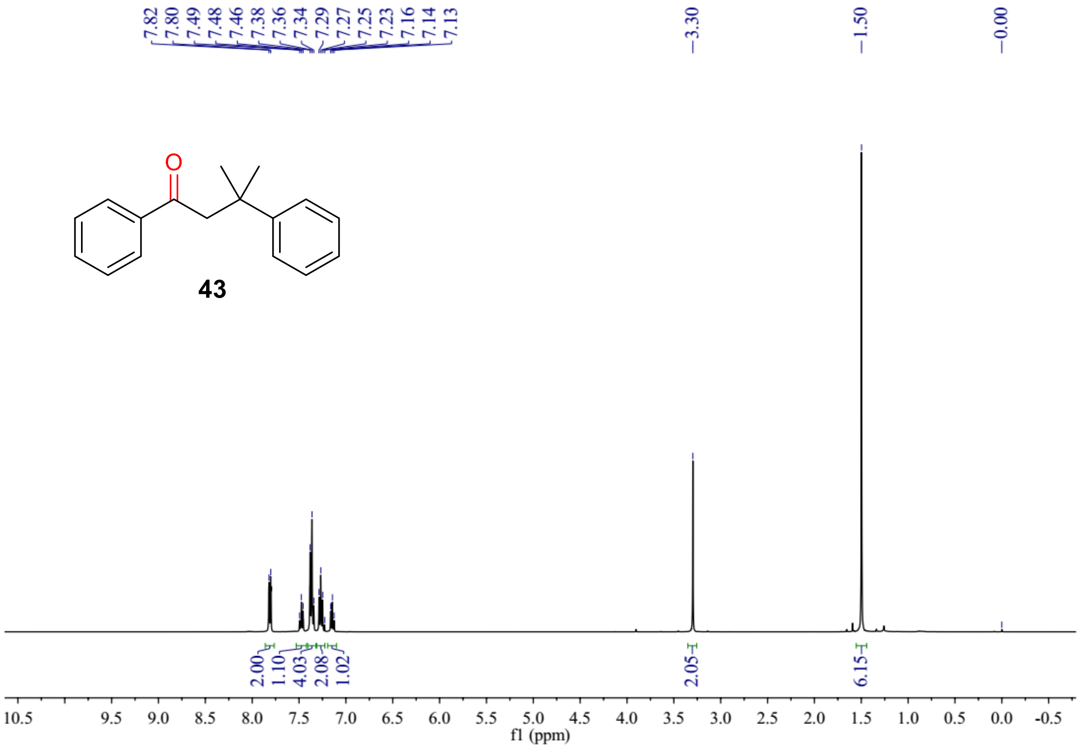

^{1}H NMR

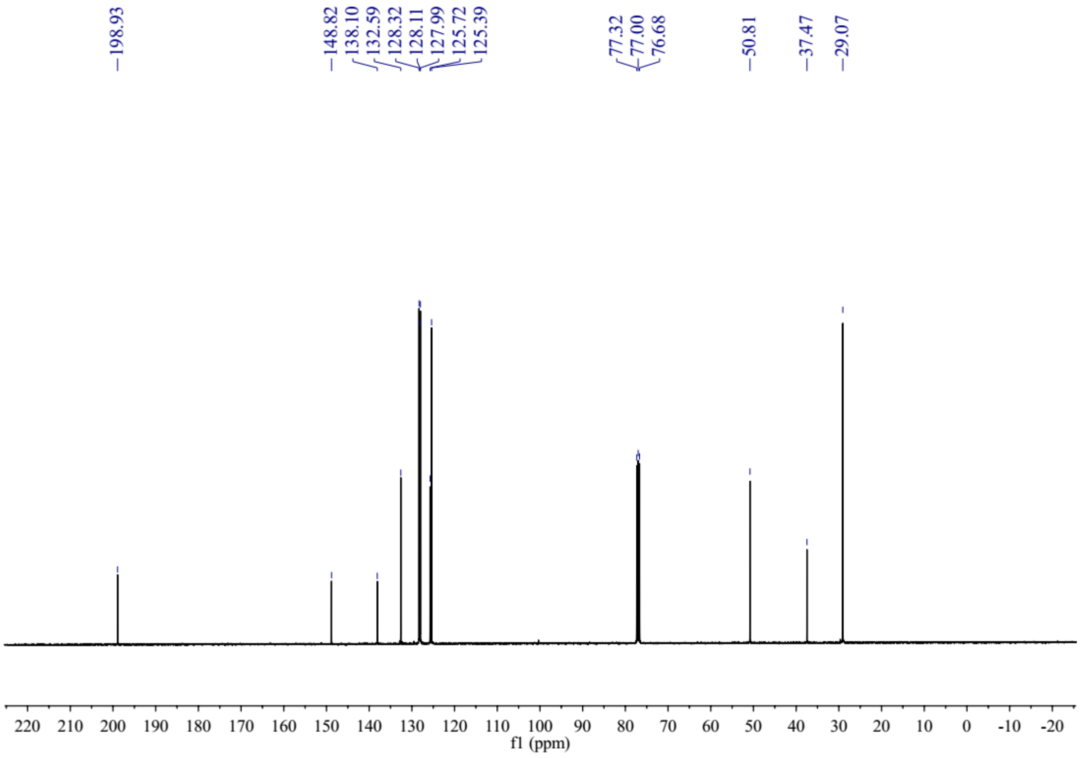

^{13}C NMR

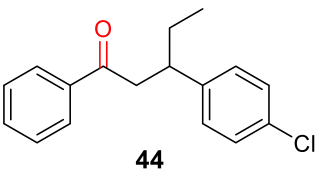

44

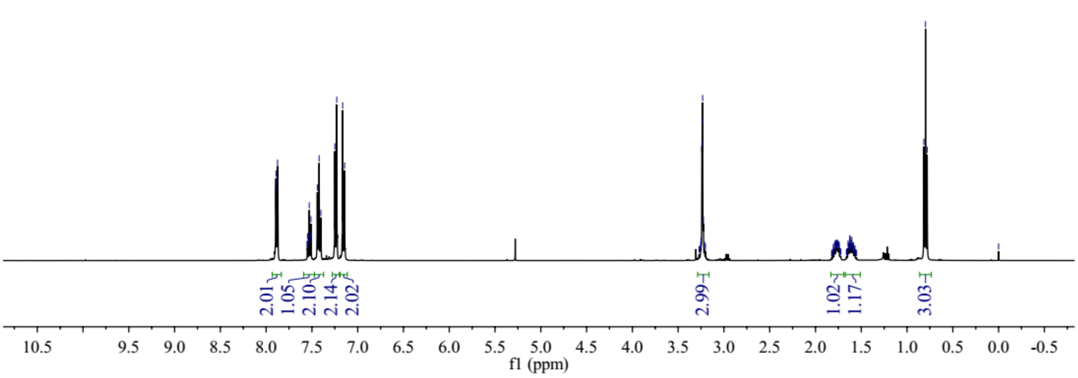

^{1}H NMR

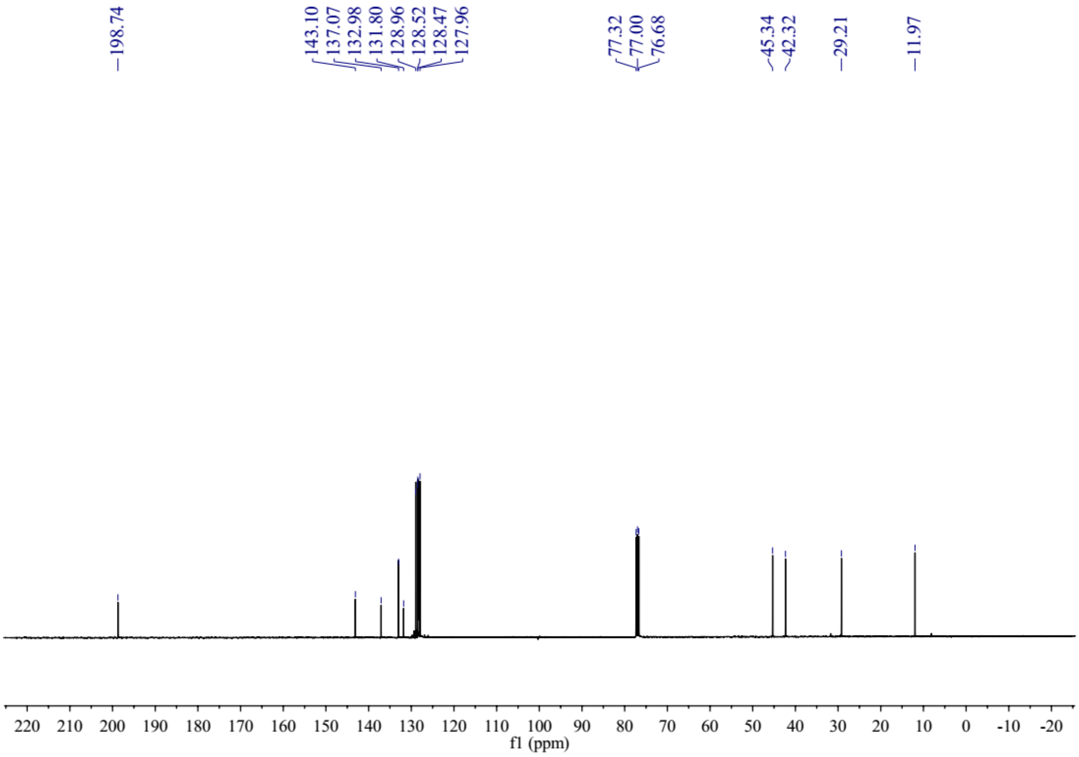

^{13}C NMR

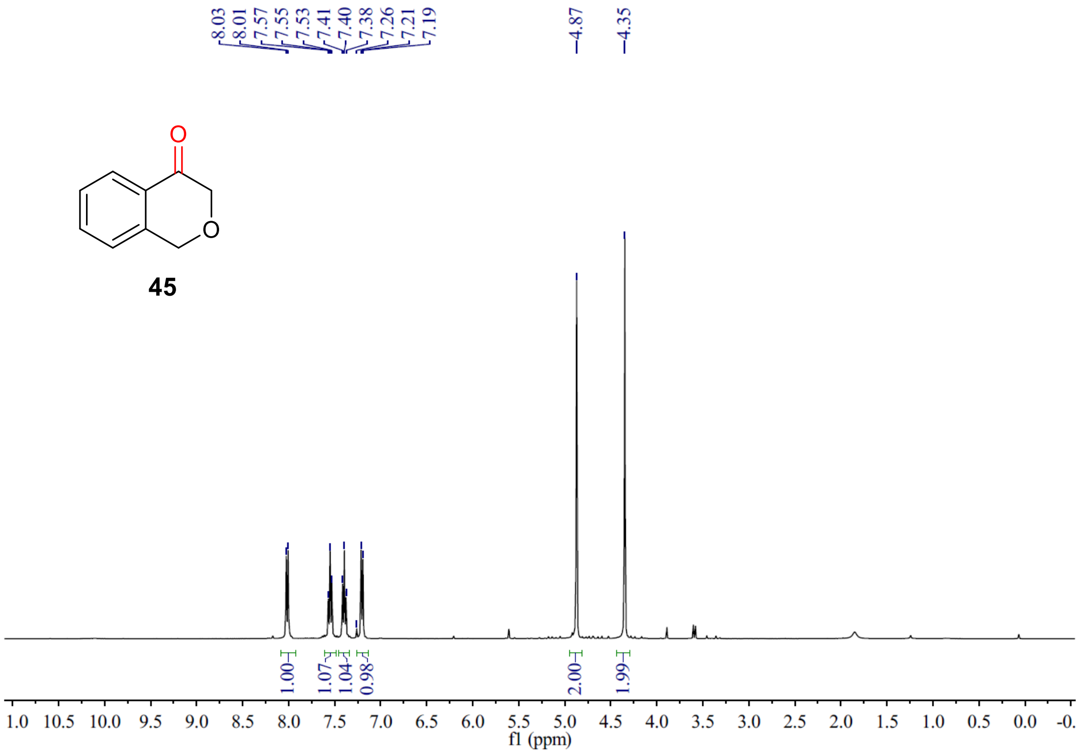

^{1}H NMR

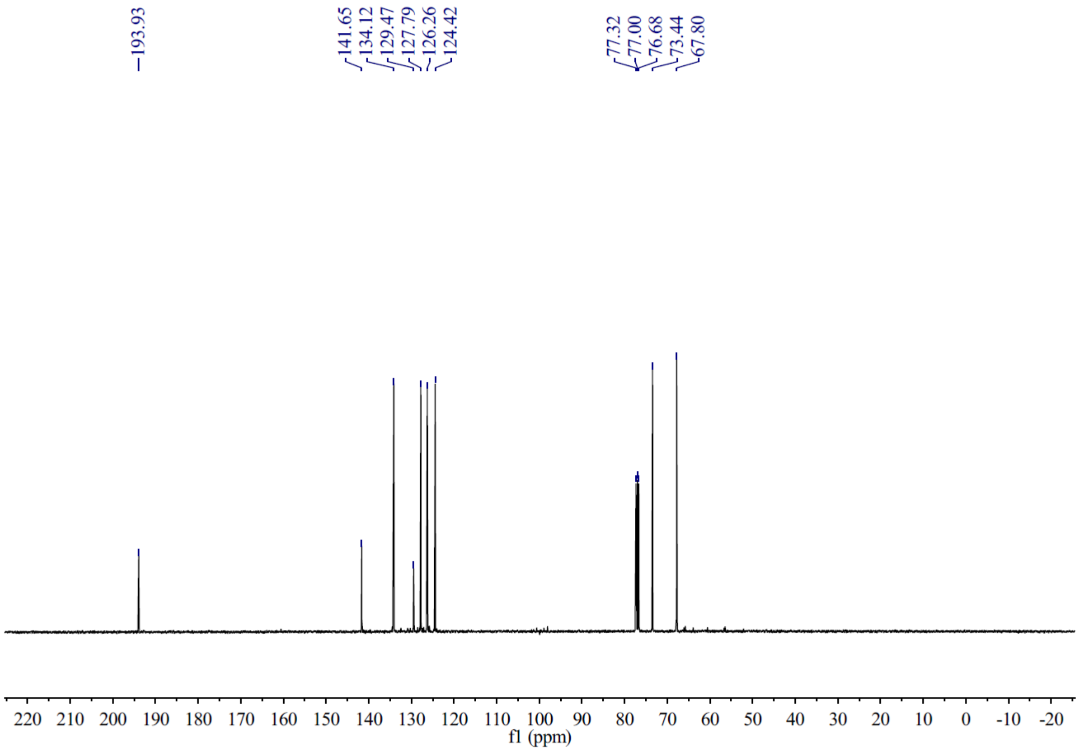

^{13}C NMR

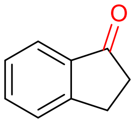

46

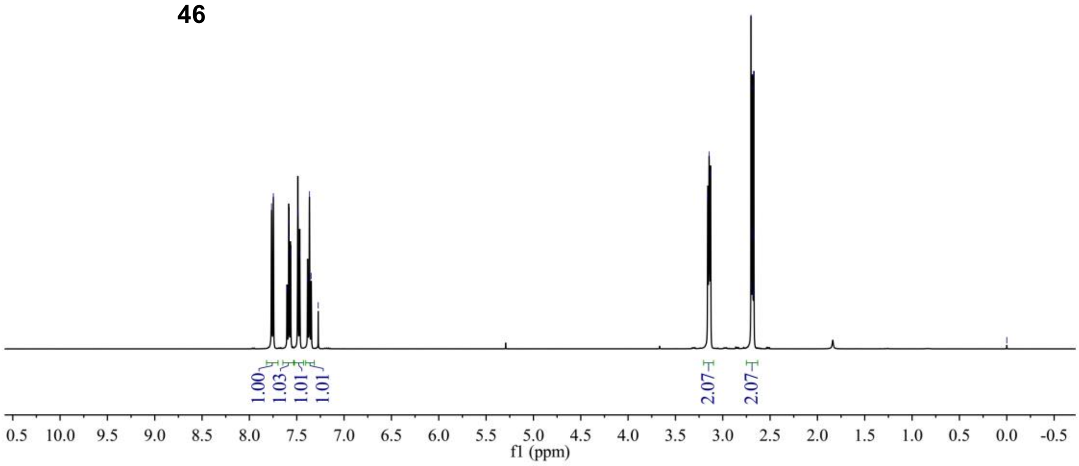

^{1}H NMR

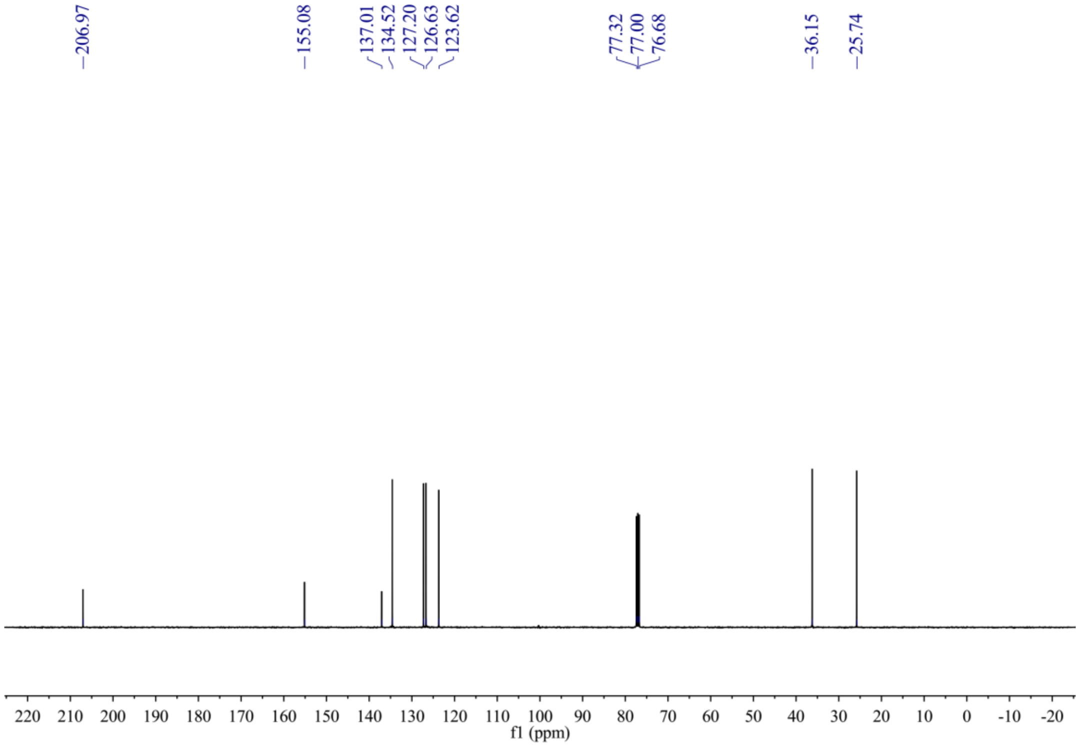

^{13}C NMR

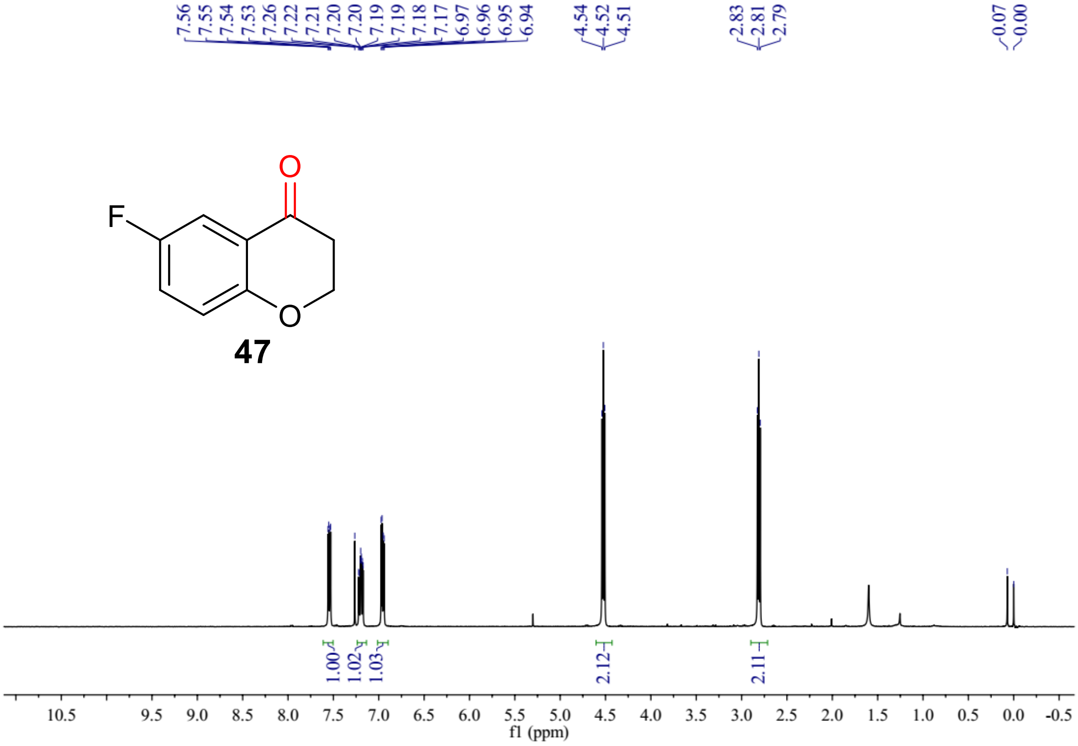

^{1}H NMR

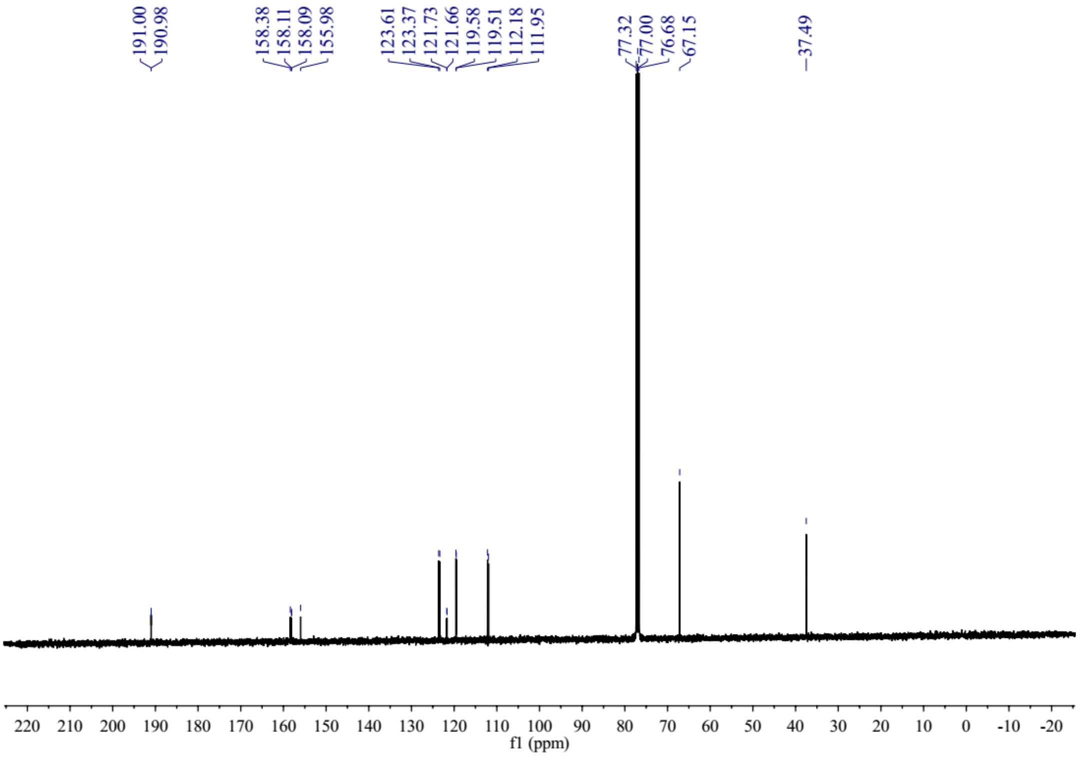

^{13}C NMR

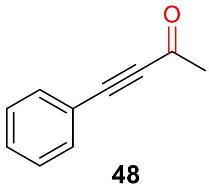

48

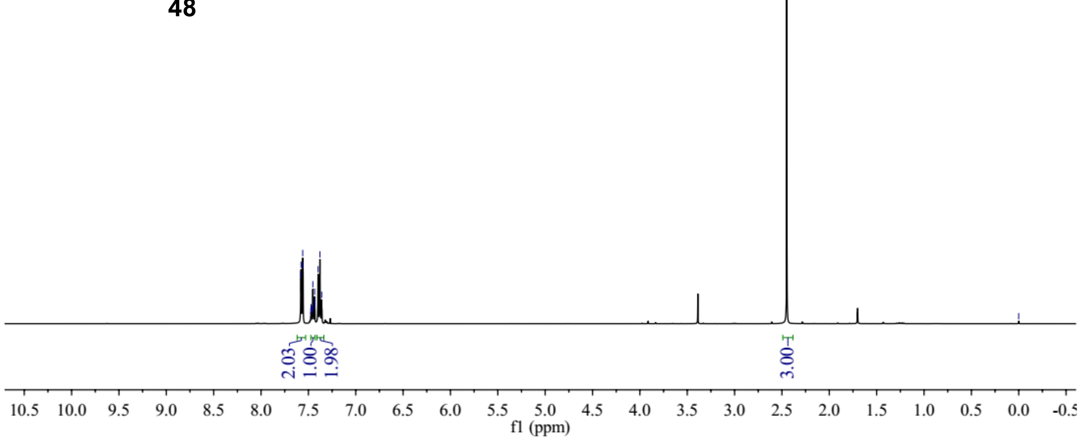

^{1}H NMR

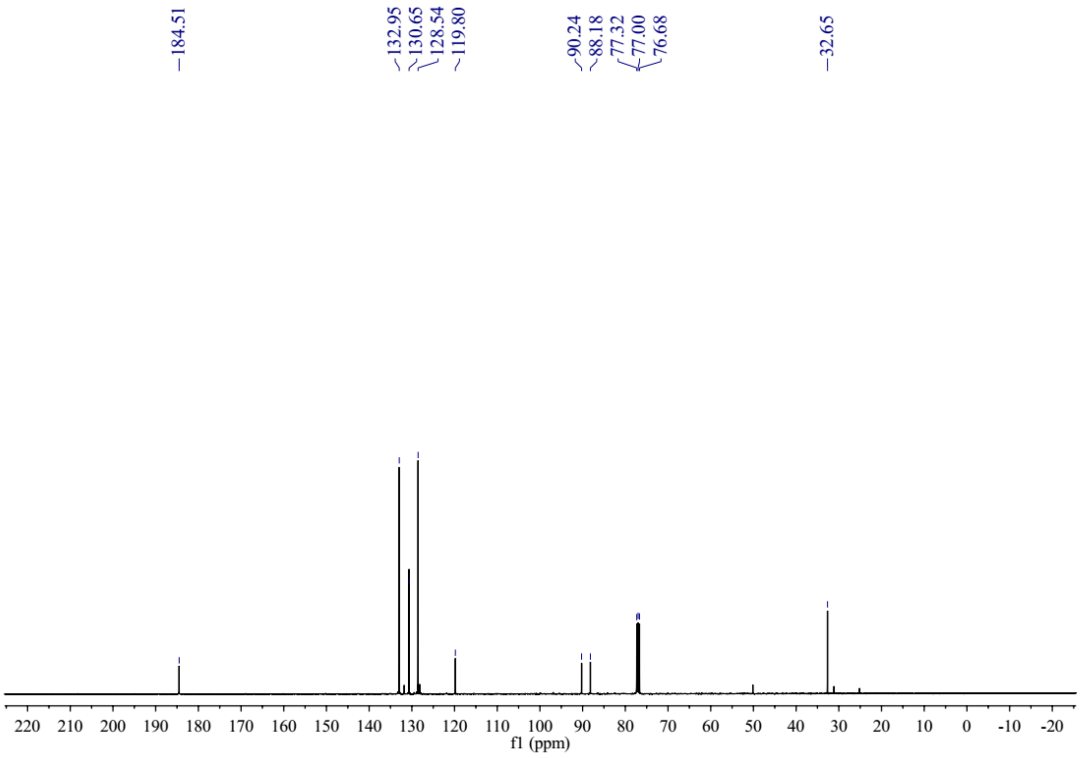

^{13}C NMR

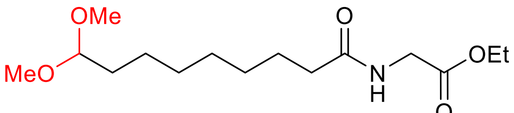

50

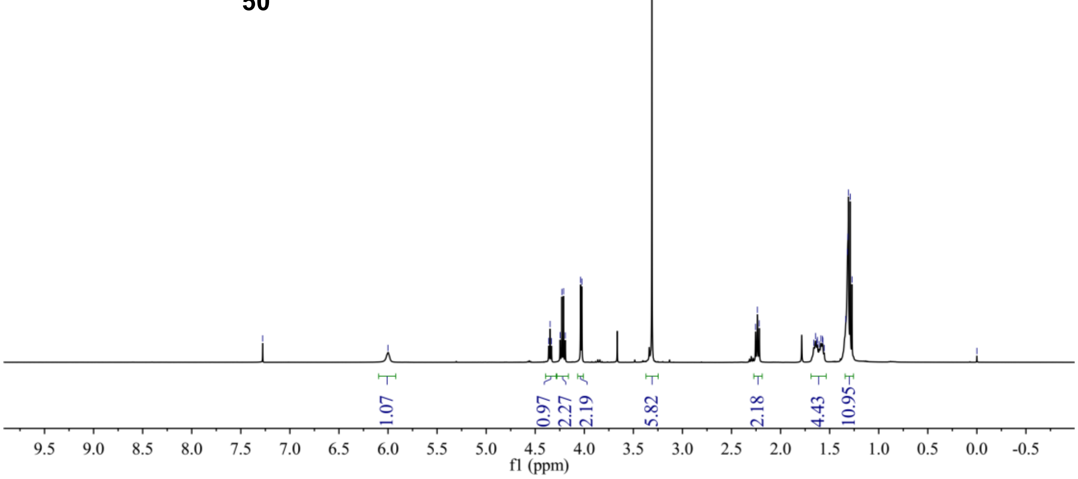

^{1}H NMR

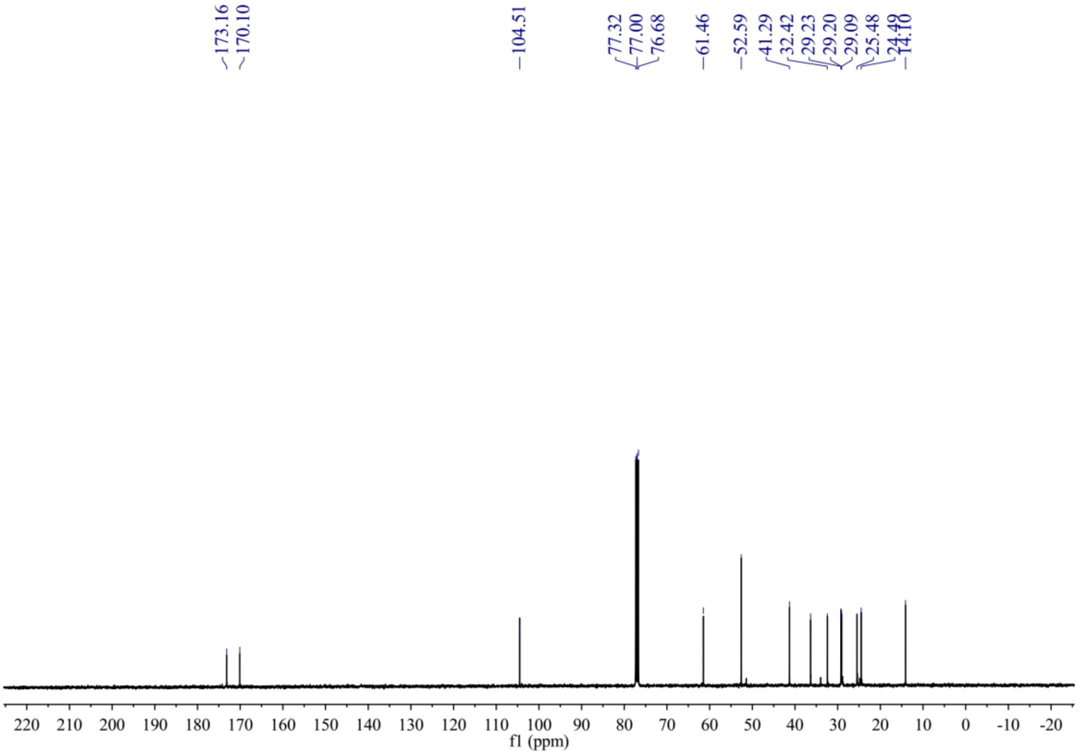

^{13}C NMR

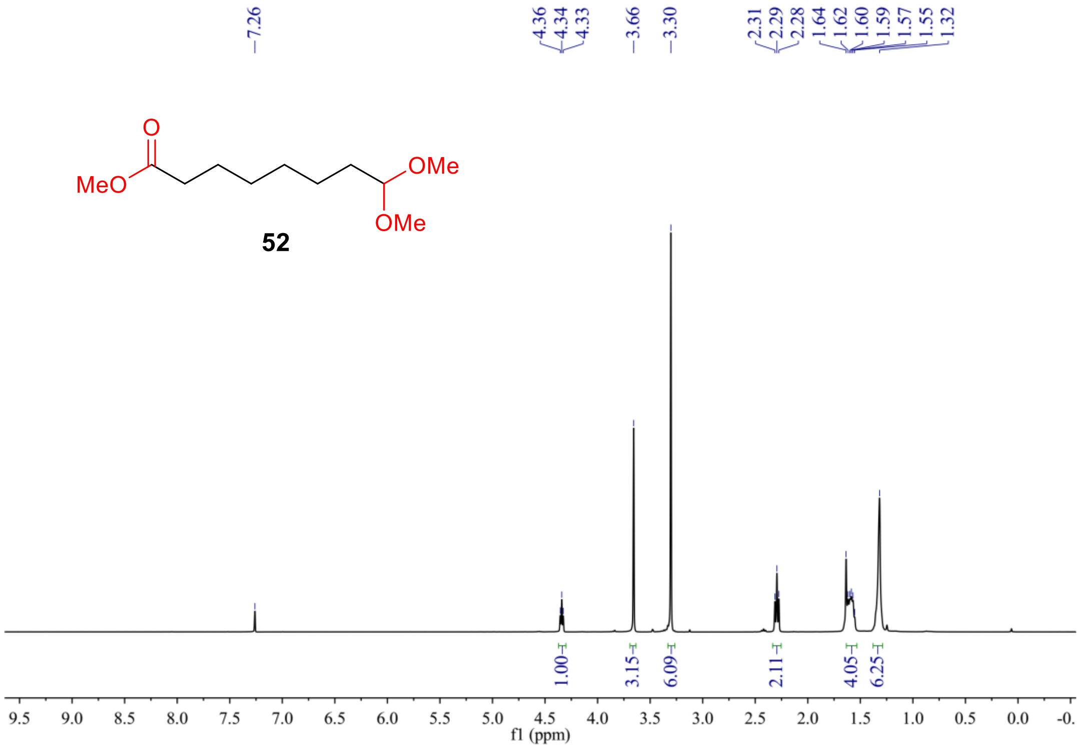

^{1}H NMR

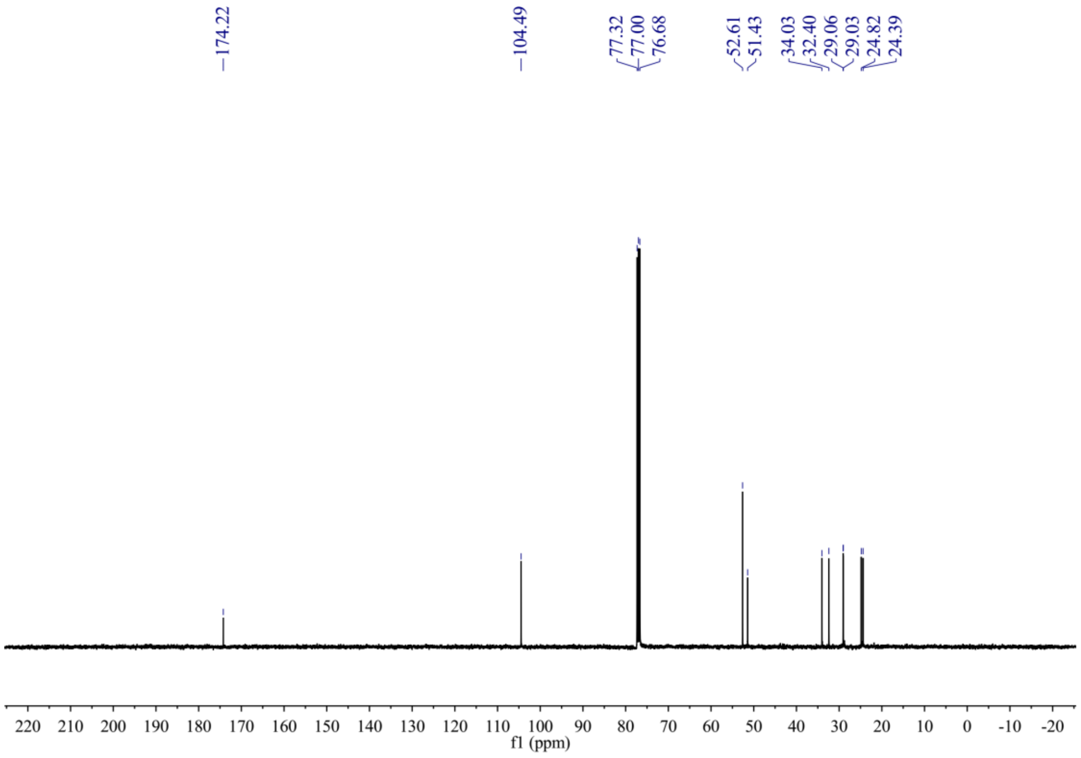

^{13}C NMR

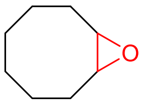

53

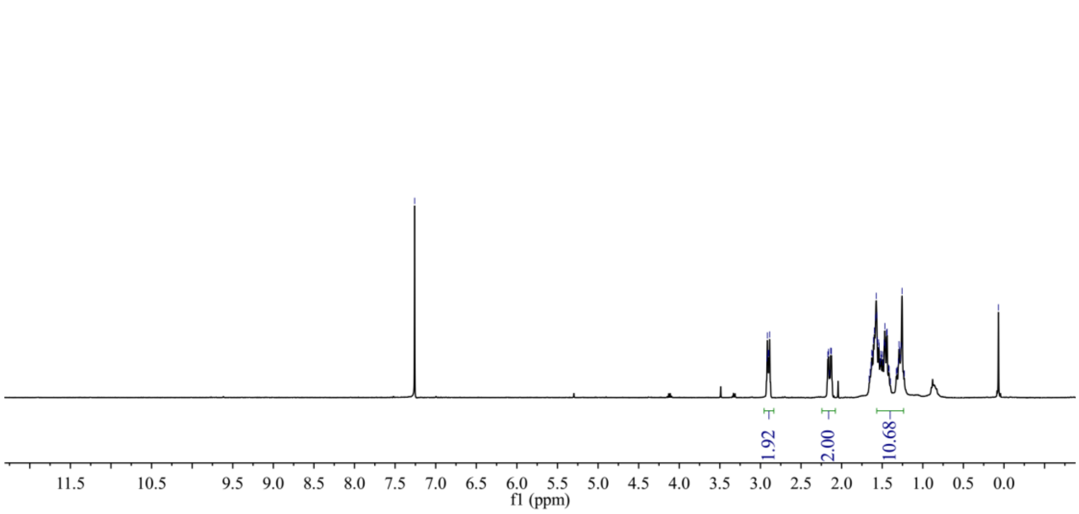

^{1}H NMR

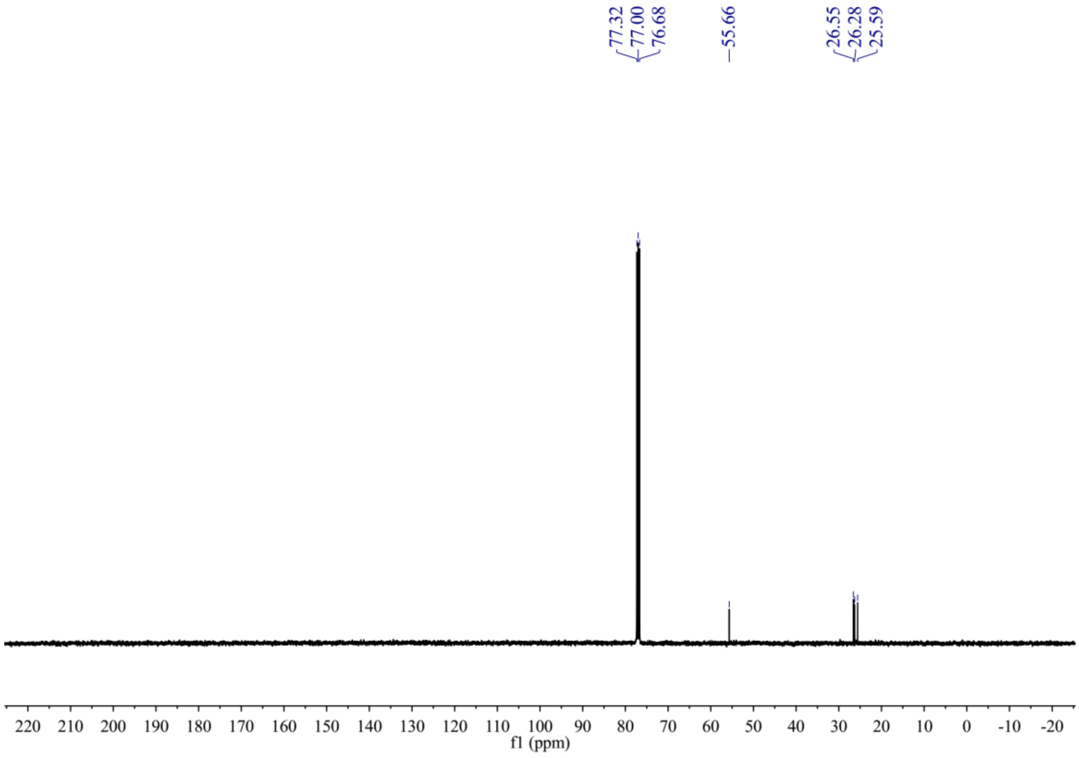

^{13}C NMR

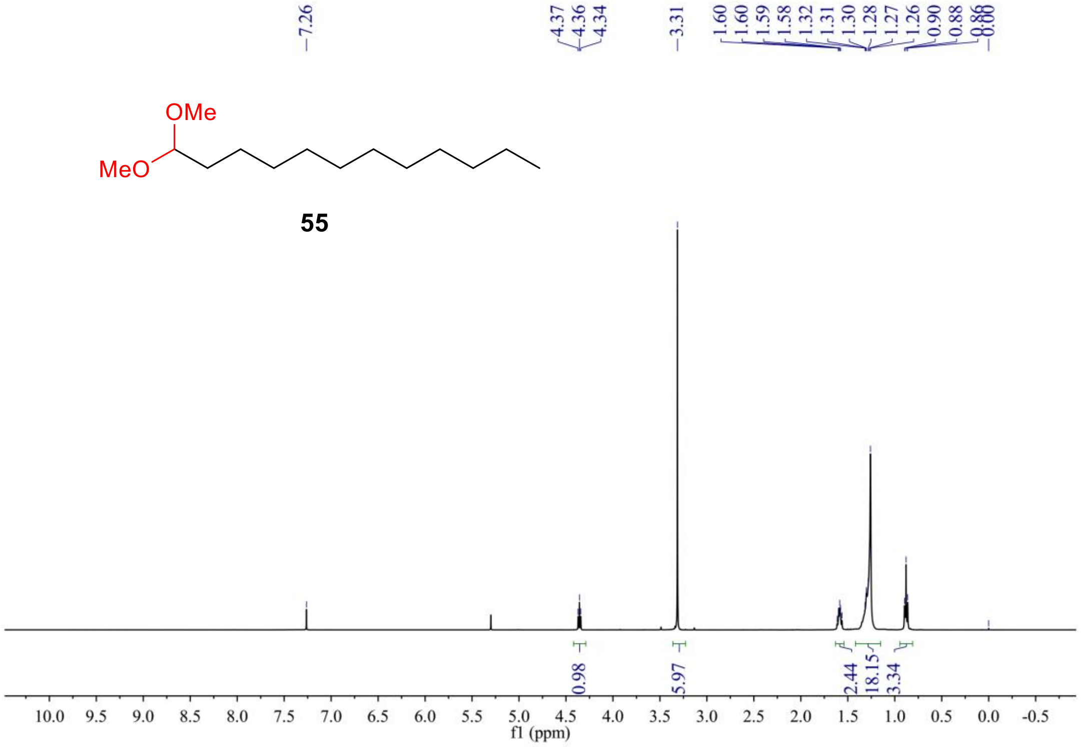

^{1}H NMR

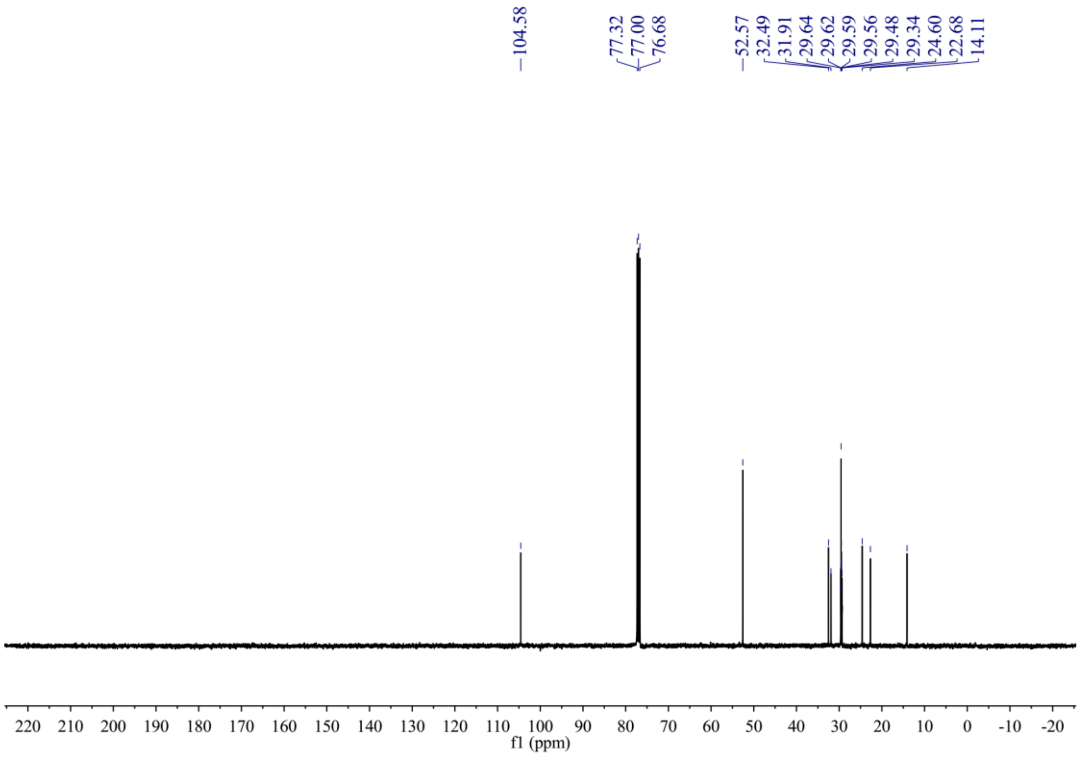

^{13}C NMR

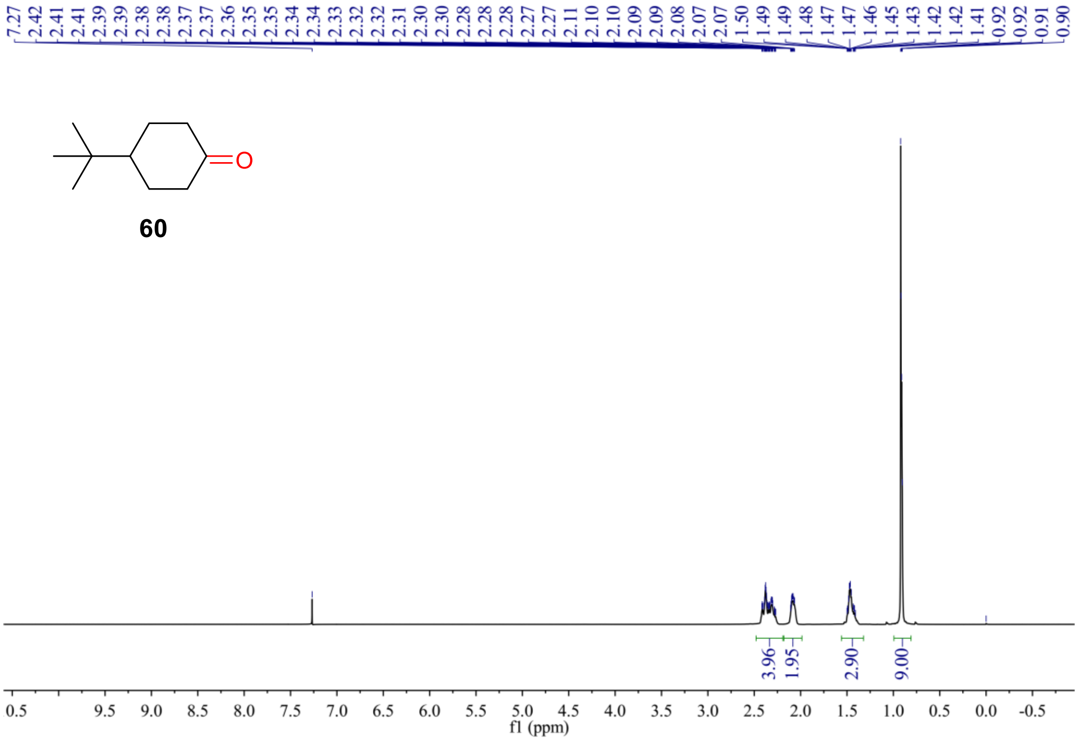

^{1}H NMR

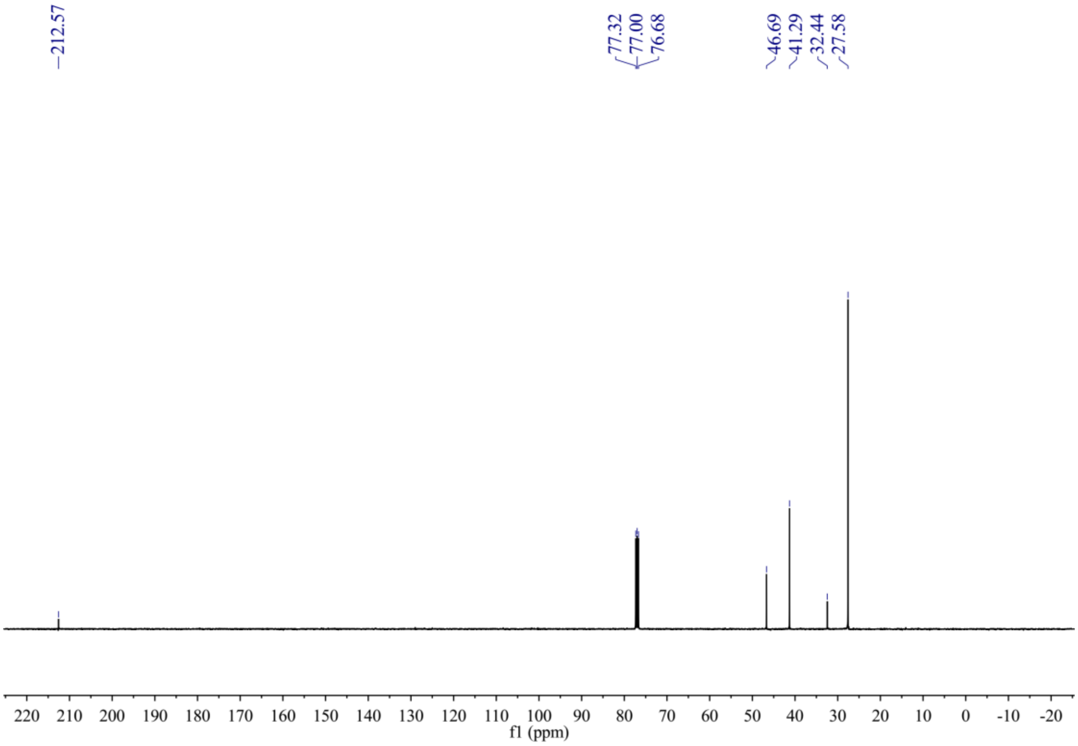

^{13}C NMR

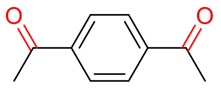

66

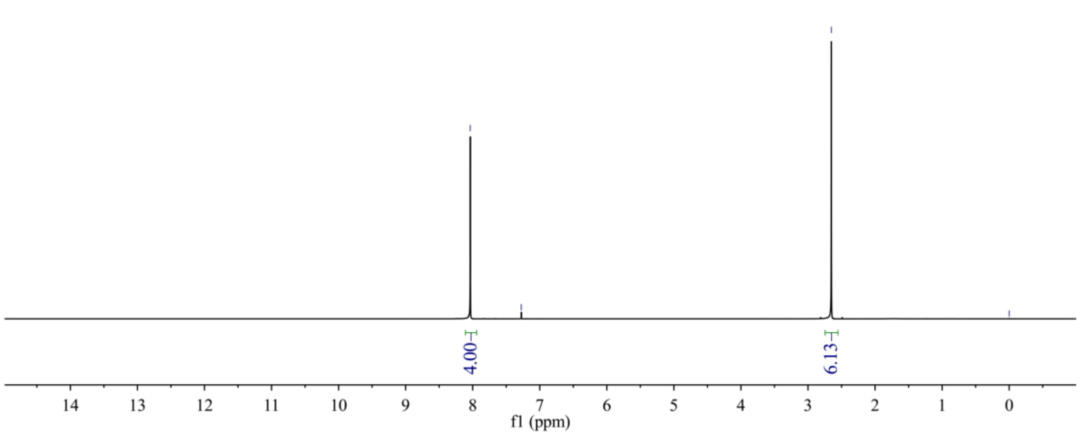

^{1}H NMR

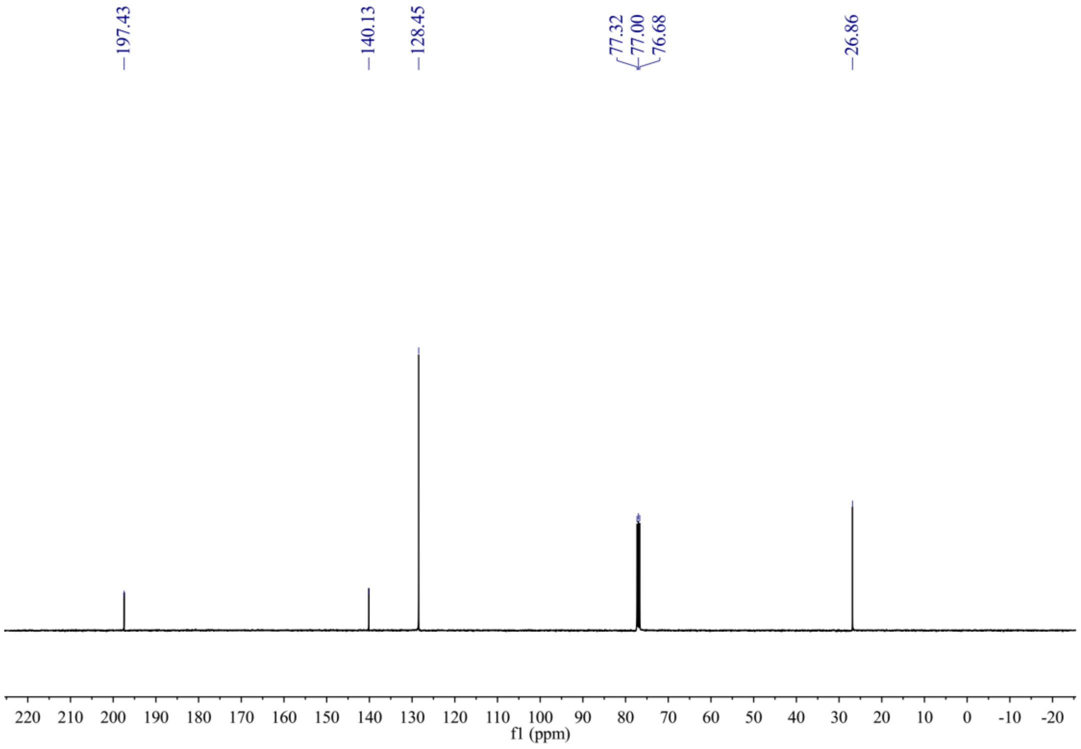

^{13}C NMR

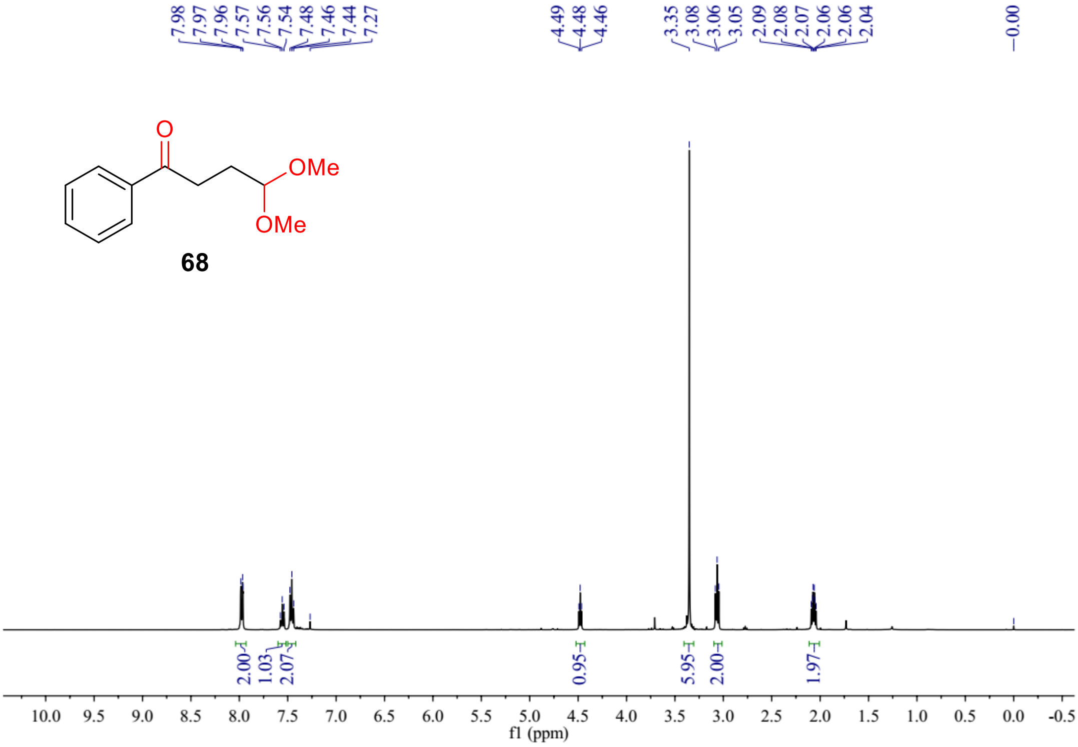

^{1}H NMR

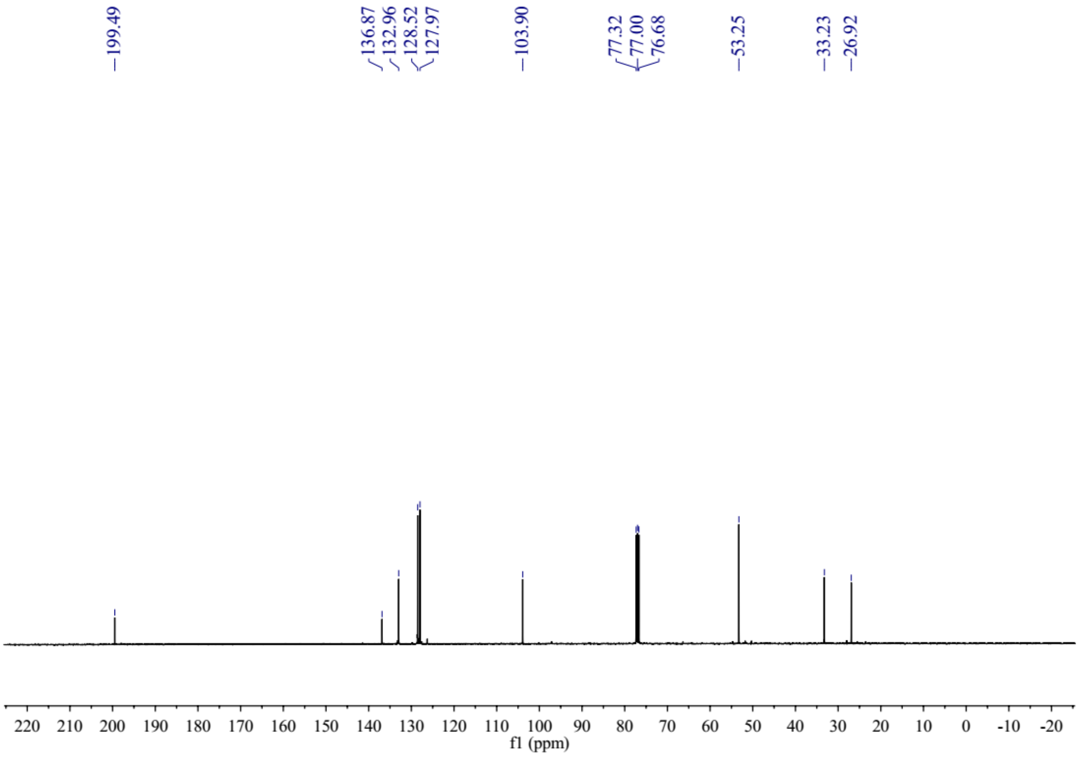

^{13}C NMR

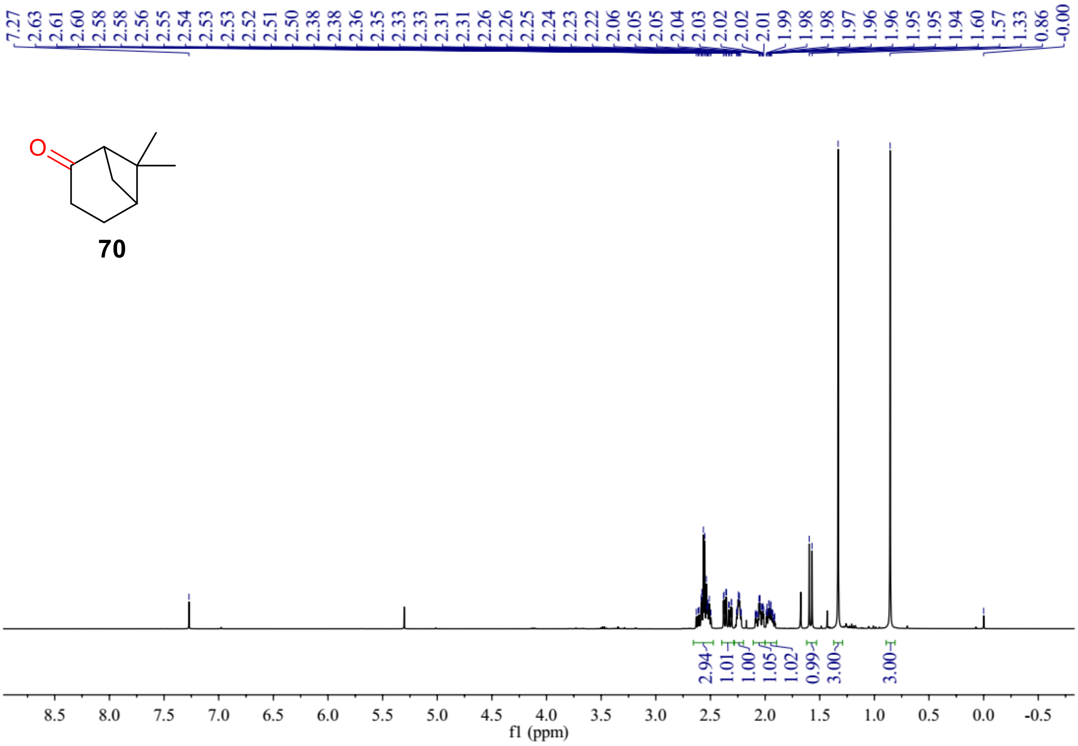

^{1}H NMR

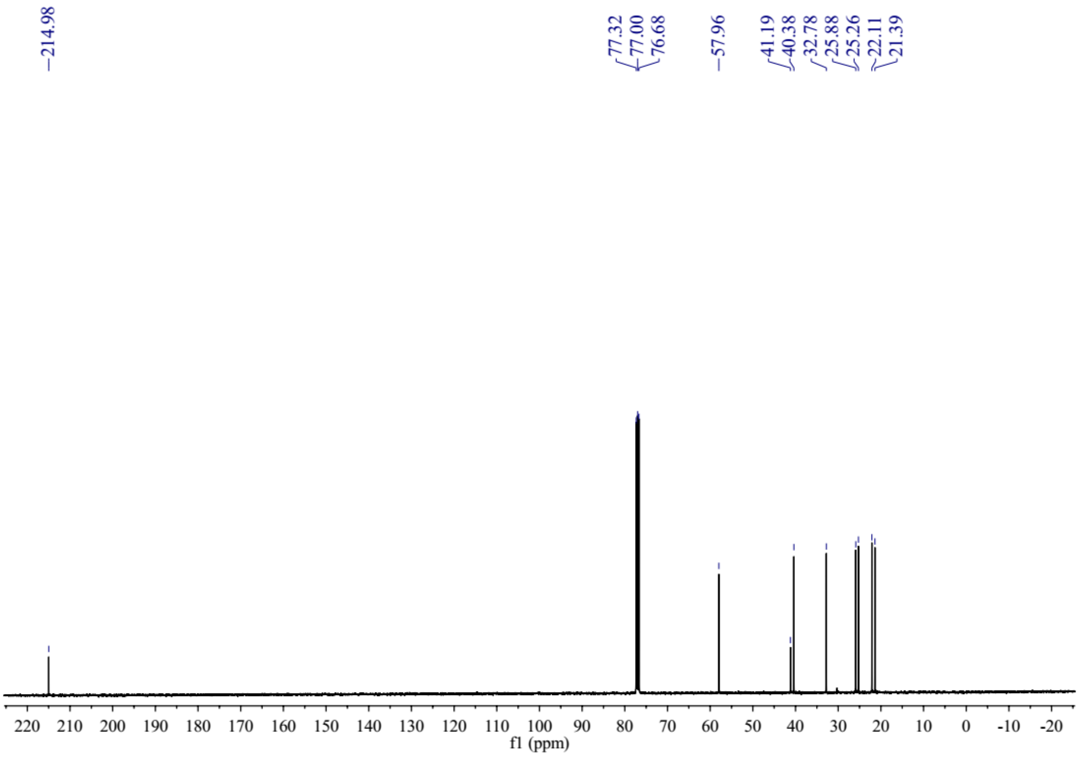

^{13}C NMR

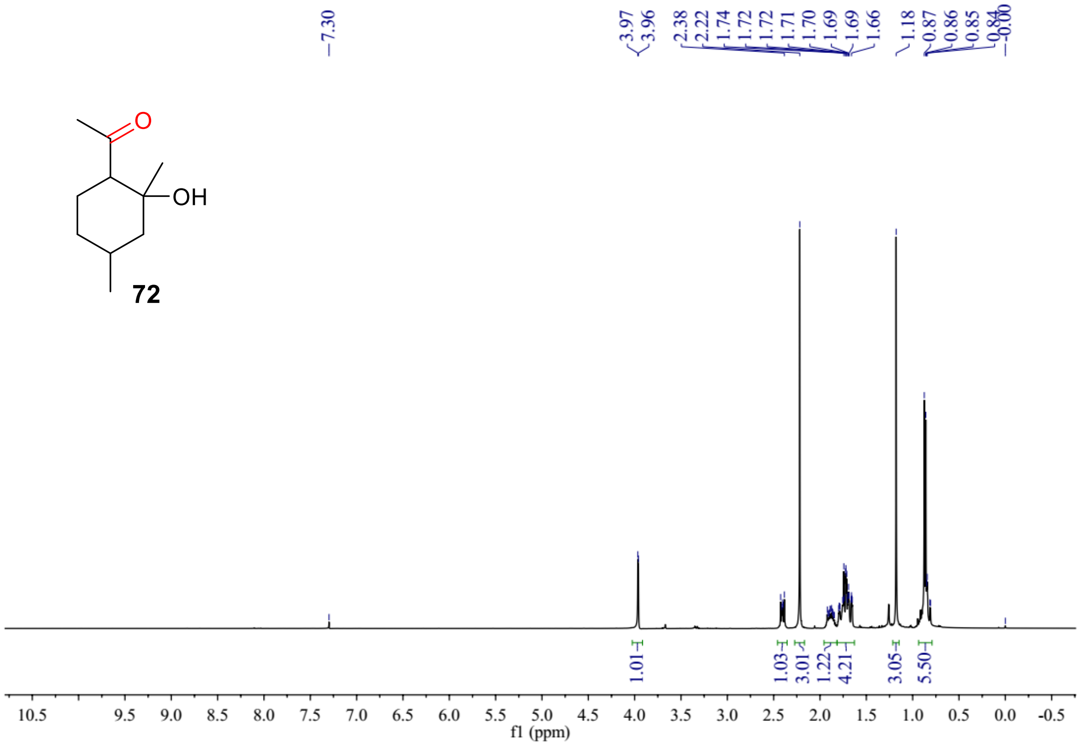

^{1}H NMR

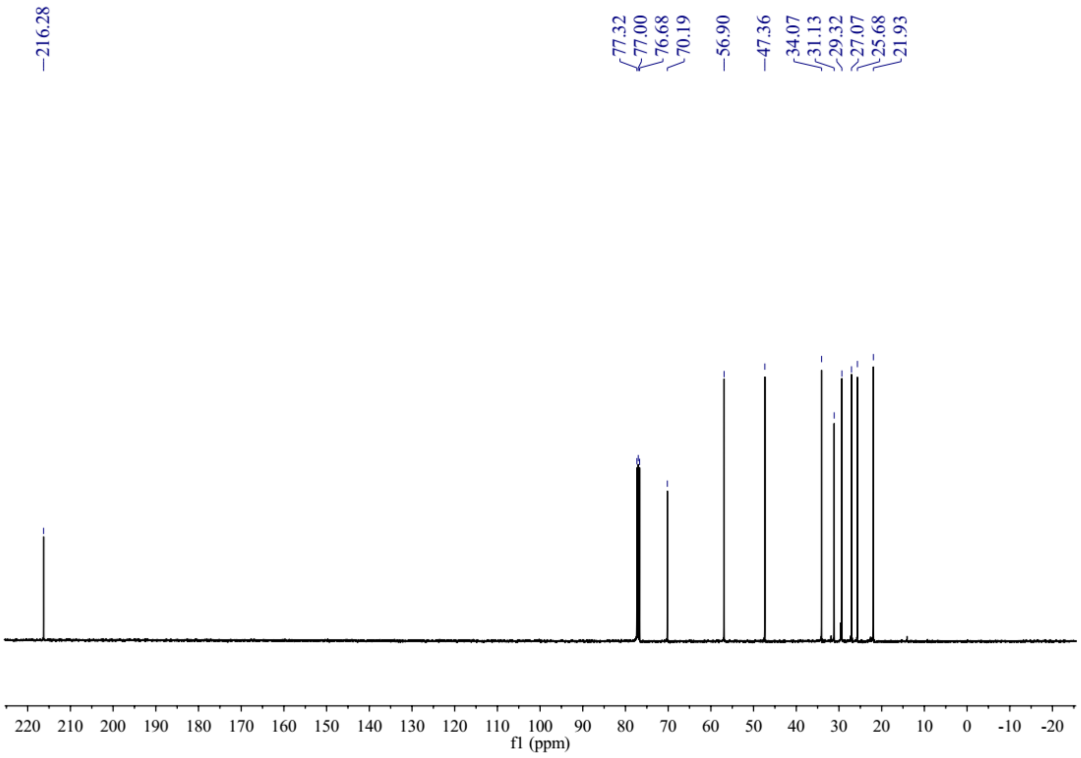

^{13}C NMR

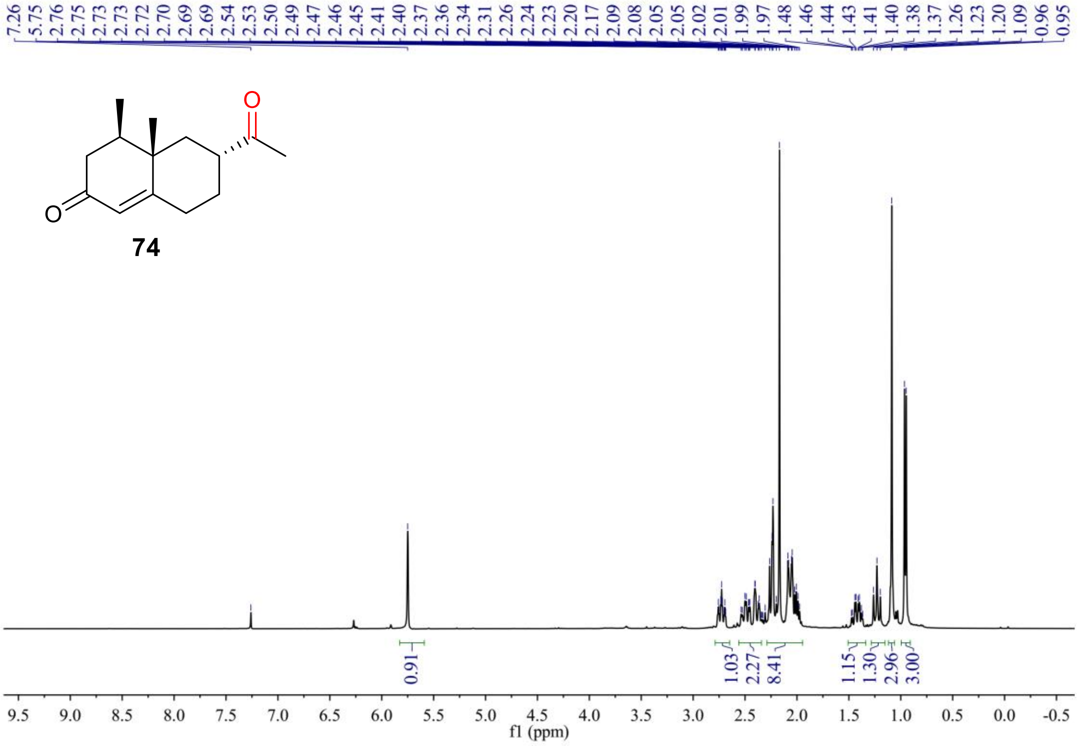

^{1}H NMR

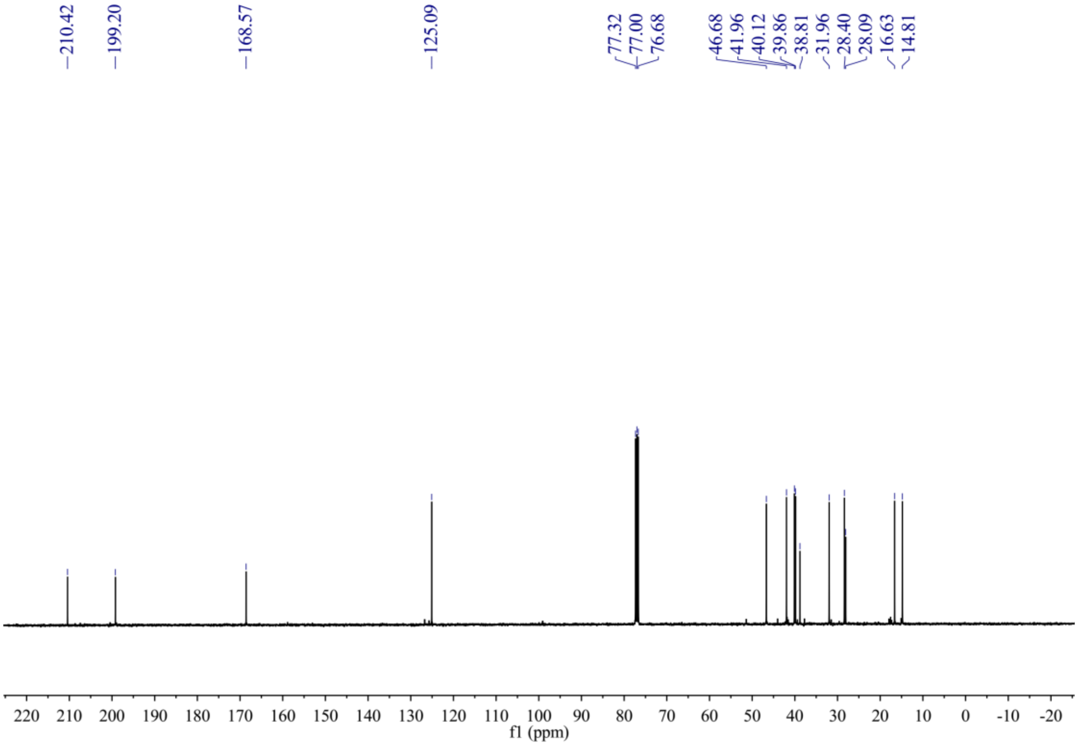

^{13}C NMR

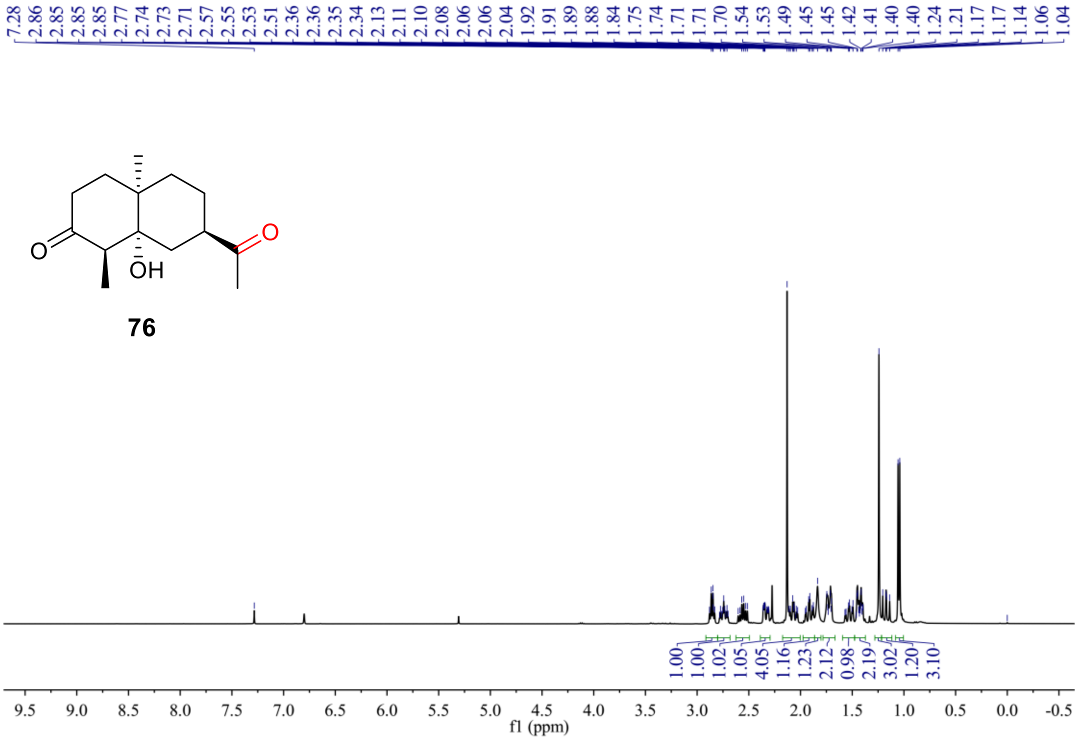

^{1}H NMR

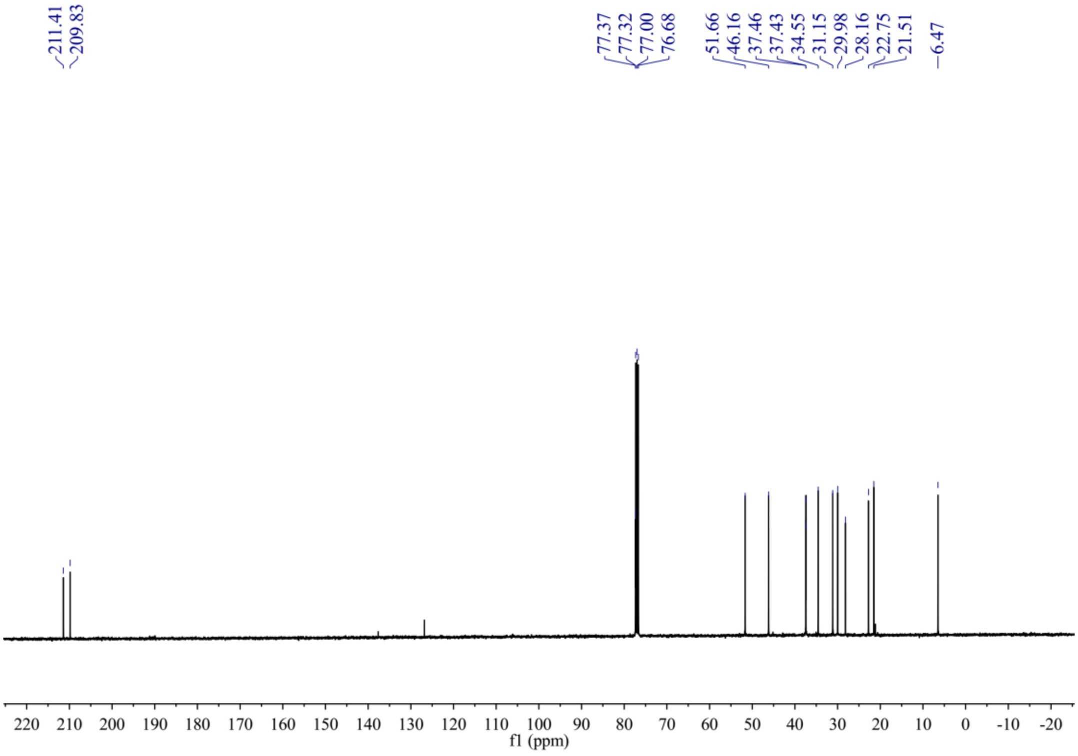

^{13}C NMR

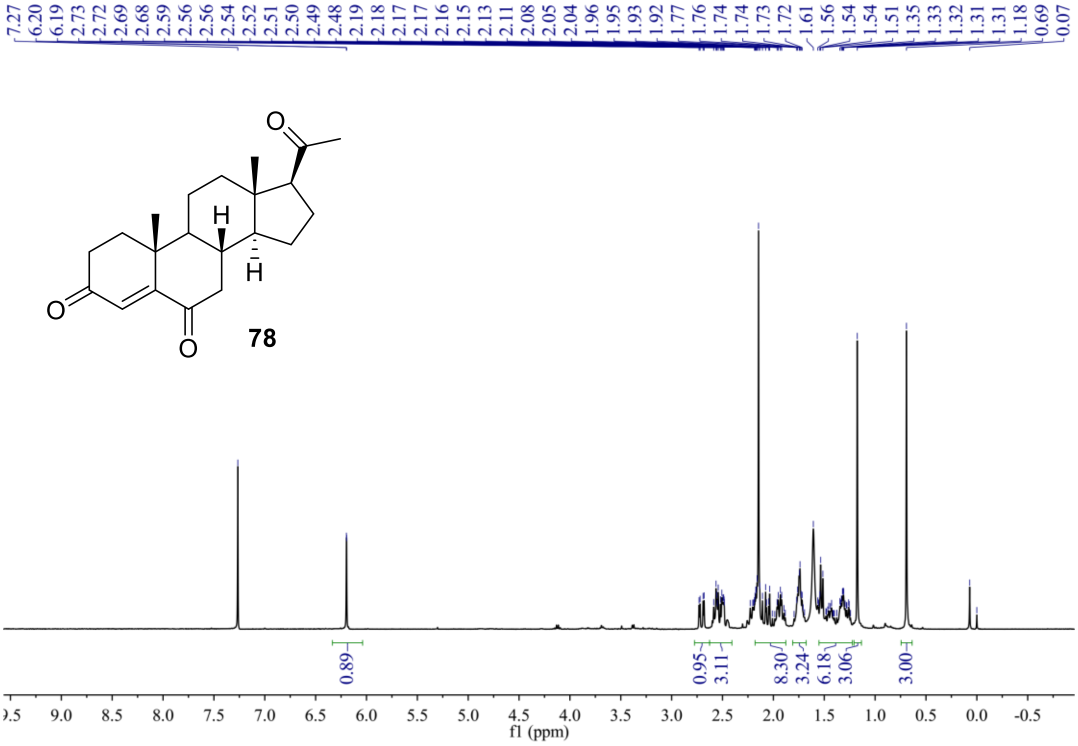

^{1}H NMR

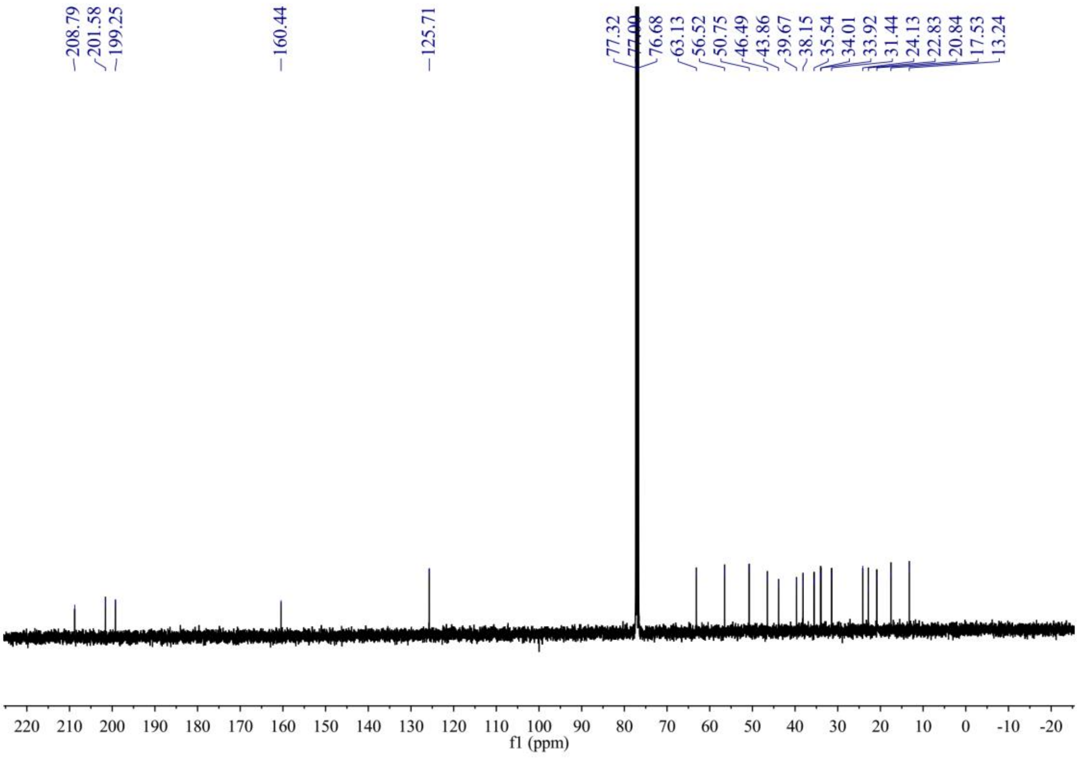

^{13}C NMR

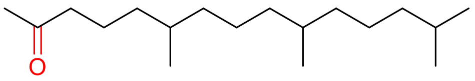

80

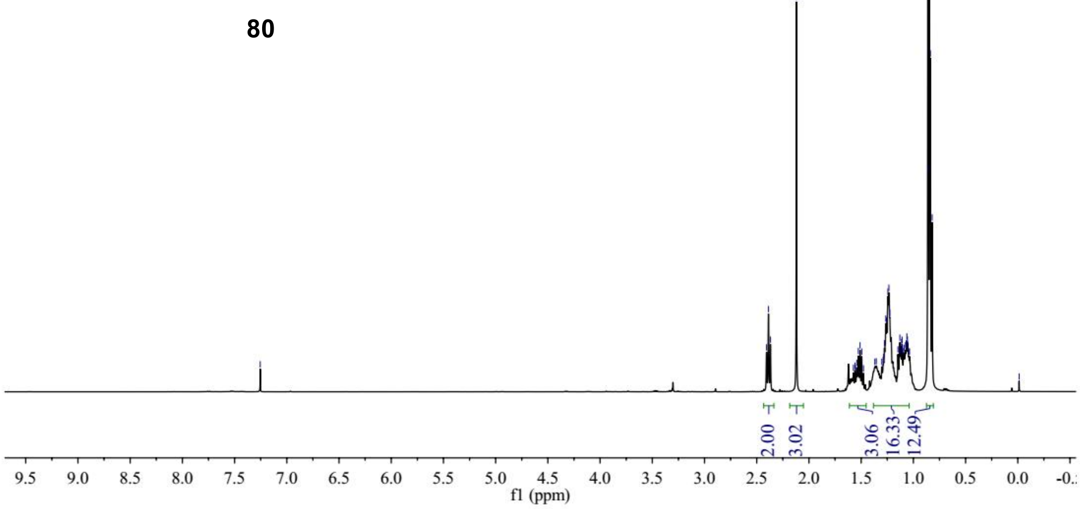

^{1}H NMR

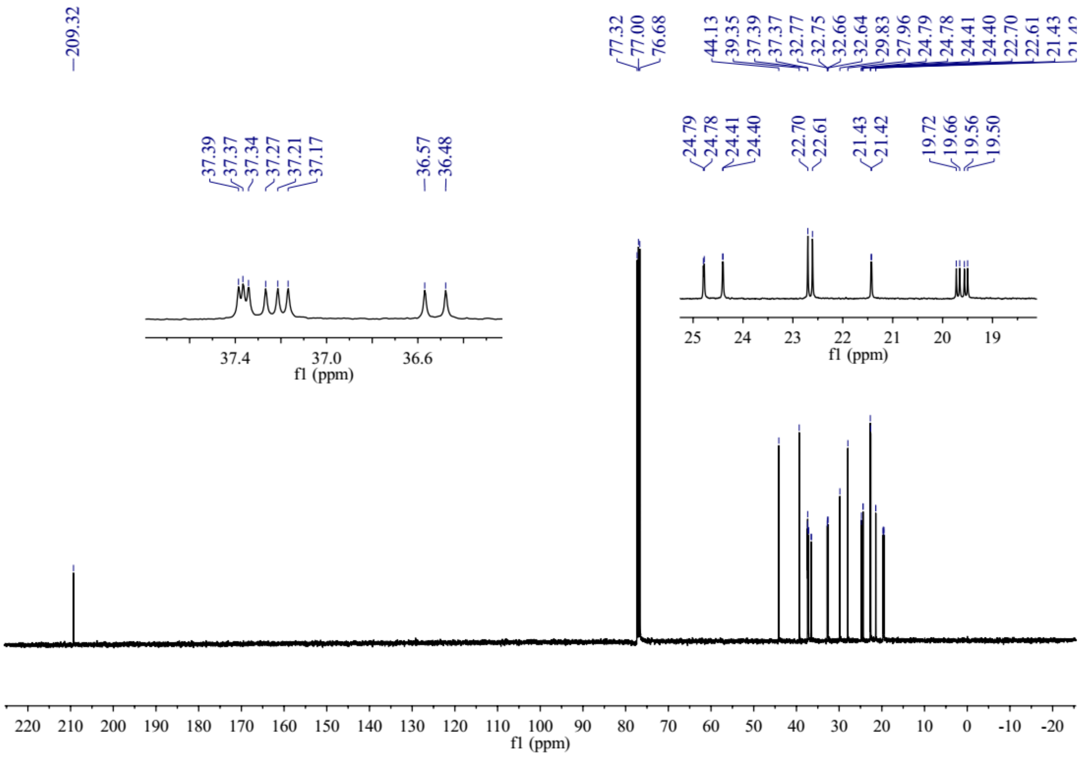

^{13}C NMR

9. Crystallographic data

9.1 Crystallographic data of [Mn(dtbpy)$_2$(OTf)$_2$]

A suitable single crystal of $C_{38}H_{48}F_6MnN_4O_6S_2$ [CCDC 1997610] was selected and mounted on a MiTeGen loop using Parabar oil and placed on a 'Bruker D8 Venture' diffractometer. The crystal was kept at 150.0 K during data collection. Using Olex2[72], the structure was solved with the ShelXS[73] structure solution program using Direct Methods and refined with the ShelXL[74] refinement package using Least Squares minimisation. The data were modelled as a pseudomerohedral twin containing a 180 degree rotation about the c axis with a ca. 60:40 split.

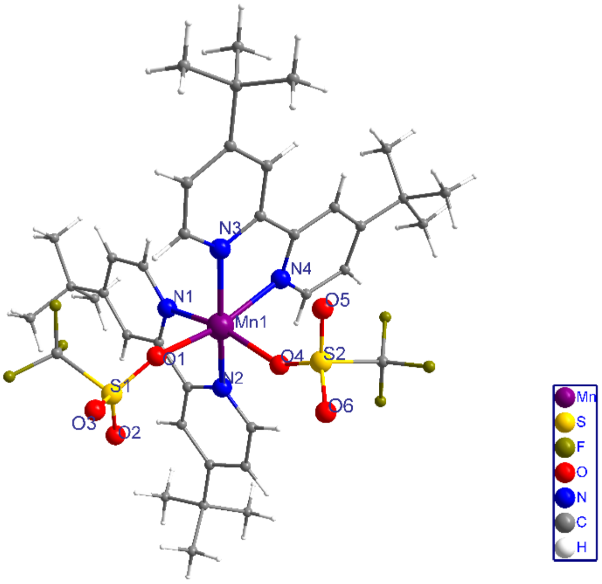

CCDC 1997610 contains the supplementary crystallographic data for this compound. This data can be obtained free of charge from The Cambridge Crystallographic Data Centre via www.ccdc.cam.ac.uk/data_request/cif.

Crystal Data for $C_{38}H_{48}F_6MnN_4O_6S_2$ (M =889.86 g/mol): monoclinic, space group P2$_1$/n (no. 14), a = 10.7999(9) Å, b = 20.6748(14) Å, c = 18.3888(12) Å, β = 90.168(3)°, V = 4105.9(5) Å^3, Z = 4, T = 150.0 K, μ(MoKα) = 0.500 mm^{-1}, $Dcalc$ = 1.440 g/cm^3, 53749 reflections measured (4.256° ≤ 2Θ ≤ 52.892°), 8415 unique (R_{int} = 0.0318, R_{sigma} = 0.0215) which were used in all calculations. The final R_1 was 0.0339 (I > 2σ(I)) and wR_2 was 0.0846 (all data).

Table S4 Crystal data and structure refinement for CCDC 1997610.

Identification code	CCDC 1997610
Empirical formula	$C_{38}H_{48}F_6MnN_4O_6S_2$
Formula weight	889.86
Temperature/K	150.0
Crystal system	monoclinic
Space group	$P2_1/n$
a/Å	10.7999(9)
b/Å	20.6748(14)
c/Å	18.3888(12)
α/°	90
β/°	90.168(3)
γ/°	90
Volume/Å^3	4105.9(5)
Z	4
ρ_{calc}g/cm^3	1.440
μ/mm^{-1}	0.500
F(000)	1852.0
Crystal size/mm^3	0.045 × 0.015 × 0.011
Radiation	MoKα (λ = 0.71073)
2Θ range for data collection/°	4.256 to 52.892
Index ranges	$-13 \leq h \leq 13$, $-25 \leq k \leq 25$, $-22 \leq l \leq 23$
Reflections collected	53749
Independent reflections	8415 [R_{int} = 0.0318, R_{sigma} = 0.0215]
Data/restraints/parameters	8415/0/528
Goodness-of-fit on F^2	1.073
Final R indexes [I>=2σ (I)]	R_1 = 0.0339, wR_2 = 0.0804
Final R indexes [all data]	R_1 = 0.0394, wR_2 = 0.0846
Largest diff. peak/hole / e Å^{-3}	0.39/-0.41

Table S5 Fractional Atomic Coordinates (×10^4) and Equivalent Isotropic Displacement Parameters (Å^2×10^3) for CCDC 1997610. U_{eq} is defined as 1/3 of of the trace of the orthogonalised U_{IJ} tensor.

Atom	*x*	*y*	*z*	U(eq)
$Mn_{(1)}$	2293.7(3)	7529.4(2)	5010.1(2)	18.02(9)
$S_{(1)}$	120.5(6)	6481.0(3)	5744.1(3)	22.46(13)
$S_{(2)}$	282.4(6)	8670.5(3)	4273.4(3)	23.14(14)

$F_{(2)}$	2006.0(17)	5714.0(8)	6036.3(11)	43.7(4)
$F_{(1)}$	235.1(18)	5243.6(7)	6036.2(9)	40.3(4)
$F_{(4)}$	2233.4(18)	9387.8(8)	4010.8(11)	48.7(5)
$O_{(1)}$	826.3(18)	6855.5(8)	5221.2(10)	28.4(4)
$N_{(1)}$	3672(2)	6859.3(9)	5530.7(11)	20.5(4)
$O_{(4)}$	993.0(18)	8297.8(8)	4808.4(10)	28.9(4)
$N_{(4)}$	3763(2)	8118.6(9)	4479.7(11)	21.1(4)
$O_{(2)}$	278(2)	6689.1(9)	6486.0(10)	34.7(5)
$F_{(5)}$	1233(2)	9656.5(8)	4968.8(10)	50.1(5)
$N_{(3)}$	2428.3(19)	7195.1(9)	3855.5(11)	19.4(4)
$F_{(3)}$	1061(2)	5512.6(8)	5023.2(10)	48.9(5)
$F_{(6)}$	496(2)	9889.7(8)	3910.9(11)	48.6(5)
$O_{(6)}$	-936.2(19)	8826.3(9)	4506.7(11)	33.8(4)
$N_{(2)}$	2611(2)	7859.1(9)	6157.6(11)	20.2(4)
$O_{(5)}$	406(2)	8449.2(9)	3535.4(10)	35.5(5)
$O_{(3)}$	-1116.8(18)	6342.1(9)	5507.4(11)	34.5(5)
$C_{(24)}$	3878(2)	8074.3(10)	3748.0(12)	17.6(5)
$C_{(1)}$	3884(2)	6911.1(10)	6257.3(12)	17.2(5)
$C_{(25)}$	4647(2)	8482.0(10)	3367.1(13)	18.7(5)
$C_{(7)}$	3296(3)	7571.0(11)	7362.1(14)	18.8(5)
$C_{(19)}$	3135(2)	7546.2(10)	3401.3(13)	17.0(5)
$C_{(21)}$	2509(2)	6892.6(11)	2363.0(13)	19.5(5)
$C_{(2)}$	4659(2)	6482.6(11)	6624.7(13)	19.1(5)
$C_{(6)}$	3238(2)	7463.8(10)	6614.9(13)	16.6(5)
$C_{(20)}$	3182(2)	7406.5(11)	2658.9(13)	17.8(5)
$C_{(28)}$	4433(3)	8564.3(12)	4825.0(14)	28.1(6)
$C_{(11)}$	6118(2)	5511.6(11)	6653.5(13)	23.5(5)
$C_{(29)}$	2600(3)	6729.1(11)	1554.2(13)	22.0(5)
$C_{(5)}$	4257(3)	6390.1(12)	5172.2(14)	24.9(5)
$C_{(3)}$	5280(2)	5992.0(11)	6249.1(13)	20.2(5)
$C_{(10)}$	2045(2)	8374.5(11)	6445.3(14)	24.3(5)
$C_{(9)}$	2057(3)	8506.7(11)	7180.3(14)	24.3(5)
$C_{(8)}$	2677(2)	8100.1(11)	7663.0(13)	19.9(5)
$C_{(22)}$	1773(3)	6540.9(12)	2845.3(14)	24.8(5)
$C_{(26)}$	5337(2)	8958.7(11)	3729.6(13)	19.7(5)
$C_{(4)}$	5063(3)	5959.5(11)	5502.1(14)	25.5(6)
$C_{(27)}$	5221(3)	8987.3(12)	4482.8(14)	27.4(6)

$C_{(15)}$	2655(3)	8236.2(11)	8481.5(13)	22.1(5)
$C_{(14)}$	7071(3)	5876.2(13)	7119.7(15)	29.7(6)
$C_{(35)}$	7117(3)	9038.2(12)	2879.2(15)	28.2(6)
$C_{(37)}$	900(3)	5695.8(12)	5708.7(15)	29.1(6)
$C_{(33)}$	6148(2)	9424.8(11)	3304.7(13)	21.0(5)
$C_{(34)}$	6833(3)	9899.9(13)	3803.8(16)	33.2(6)
$C_{(12)}$	6812(3)	5069.4(14)	6132.7(17)	38.9(7)
$C_{(36)}$	5310(3)	9800.5(13)	2772.1(16)	30.4(6)
$C_{(23)}$	1759(3)	6707.8(11)	3571.5(14)	24.4(5)
$C_{(13)}$	5286(3)	5103.6(14)	7144.5(18)	40.8(7)
$C_{(38)}$	1110(3)	9444.1(12)	4292.2(15)	32.1(6)
$C_{(32)}$	3963(3)	6630.8(13)	1371.0(14)	32.0(6)
$C_{(16)}$	3377(3)	7732.4(13)	8923.4(14)	35.0(6)
$C_{(30)}$	1907(3)	6103.4(13)	1357.7(14)	33.3(6)
$C_{(31)}$	2060(3)	7285.8(13)	1100.8(14)	34.0(6)
$C_{(17)}$	1314(3)	8223.3(16)	8729.9(16)	41.8(7)
$C_{(18)}$	3206(3)	8906.9(12)	8631.0(15)	35.9(6)

Table S6 Anisotropic Displacement Parameters (Å^2×10^3) for CCDC 1997610. The Anisotropic displacement factor exponent takes the form: -2π^2[h^{2}a*^{2}U$_{11}$+2hka*b*U$_{12}$+…].

Atom	U_{11}	U_{22}	U_{33}	U_{23}	U_{13}	U_{12}
$Mn_{(1)}$	24.03(16)	18.71(15)	11.33(14)	1.37(11)	1.0(2)	0.13(15)
$S_{(1)}$	27.7(3)	23.2(3)	16.5(3)	2.8(2)	2.4(2)	-1.2(2)
$S_{(2)}$	30.0(3)	23.1(3)	16.3(3)	0.9(2)	-2.0(2)	2.5(2)
$F_{(2)}$	35.8(10)	37.8(9)	57.4(11)	3.5(8)	-5.6(8)	6.4(7)
$F_{(1)}$	54.4(11)	24.7(7)	41.9(10)	7.5(7)	5.0(8)	-6.9(8)
$F_{(4)}$	42.0(10)	39.1(9)	65.0(13)	3.3(8)	9.1(10)	-7.7(8)
$O_{(1)}$	30.8(10)	29.3(9)	25.0(9)	7.3(7)	2.5(8)	-6.1(8)
$N_{(1)}$	24.6(11)	22.8(9)	14.1(9)	-0.8(8)	1.3(9)	1.8(8)
$O_{(4)}$	34.4(11)	28.7(9)	23.6(9)	6.0(7)	-0.8(8)	9.1(8)
$N_{(4)}$	24.8(11)	24.2(10)	14.4(9)	-0.2(7)	-0.6(9)	-2.4(8)
$O_{(2)}$	54.2(13)	31.0(9)	19.0(9)	-2.2(7)	-0.1(9)	5.6(9)
$F_{(5)}$	69.5(13)	41.5(9)	39.1(10)	-15.0(8)	-17.3(10)	-2.1(9)
$N_{(3)}$	24.8(11)	19.1(9)	14.2(9)	0.1(7)	2.7(8)	-2.5(8)
$F_{(3)}$	75.3(14)	40.5(9)	30.9(9)	-11.4(8)	14.6(10)	3.9(9)
$F_{(6)}$	66.1(13)	27.1(8)	52.4(11)	11.6(7)	-11.3(10)	4.2(8)

$O_{(6)}$	29.3(10)	39.3(10)	32.7(10)	1.5(8)	-0.7(8)	7.0(8)
$N_{(2)}$	26.2(11)	18.9(9)	15.4(10)	0.5(7)	0.8(8)	1.7(8)
$O_{(5)}$	54.6(13)	33.2(10)	18.6(9)	-2.9(7)	1.9(9)	-5.2(9)
$O_{(3)}$	24.9(10)	43.0(11)	35.7(11)	10.4(9)	0.1(8)	-5.0(9)
$C_{(24)}$	22.1(12)	16.8(10)	13.9(11)	0.0(8)	-0.3(10)	2.2(9)
$C_{(1)}$	20.9(12)	17.2(10)	13.5(11)	-0.3(8)	3.9(10)	-4.4(9)
$C_{(25)}$	23.3(13)	17.5(10)	15.4(11)	-0.3(8)	0.8(10)	1.9(10)
$C_{(7)}$	25.1(14)	14.4(10)	16.8(11)	0.5(8)	0.7(10)	-0.1(9)
$C_{(19)}$	18.1(12)	16.5(10)	16.4(12)	1.7(8)	-0.5(10)	0.2(9)
$C_{(21)}$	23.5(13)	18.7(10)	16.4(11)	-0.7(8)	-0.8(10)	0.0(9)
$C_{(2)}$	22.5(12)	20.0(10)	14.7(11)	-0.3(8)	1.6(10)	-1.4(10)
$C_{(6)}$	18.7(12)	16.2(10)	14.8(12)	2.2(8)	1.5(10)	-1.0(9)
$C_{(20)}$	20.8(12)	18.7(10)	13.8(11)	1.4(9)	3.0(10)	-2.0(9)
$C_{(28)}$	35.8(15)	34.5(13)	14.2(12)	-3.4(9)	-1.3(10)	-6.9(11)
$C_{(11)}$	25.9(14)	21.6(11)	22.9(13)	0.4(9)	-0.5(11)	5.5(10)
$C_{(29)}$	27.1(14)	21.6(11)	17.4(12)	-3.9(9)	0.3(10)	-2.2(10)
$C_{(5)}$	32.9(14)	28.4(12)	13.5(11)	-3.9(9)	1.7(10)	2.4(10)
$C_{(3)}$	20.3(12)	19.9(10)	20.5(12)	0.4(9)	1.9(10)	-0.3(9)
$C_{(10)}$	31.9(14)	20.2(11)	20.8(12)	0.8(9)	-1.9(10)	5.8(10)
$C_{(9)}$	32.7(14)	19.1(11)	21.1(12)	-5.0(9)	0.2(10)	7.1(10)
$C_{(8)}$	23.2(12)	19.6(10)	17.0(11)	-2.1(9)	0.7(10)	-2.9(10)
$C_{(22)}$	31.1(14)	22.8(11)	20.3(12)	-2.4(9)	1.6(10)	-9.4(10)
$C_{(26)}$	21.3(12)	18.5(10)	19.2(12)	0.7(9)	-2.2(10)	0.8(9)
$C_{(4)}$	33.6(15)	23.2(11)	19.7(12)	-4.1(9)	4.3(11)	6.9(11)
$C_{(27)}$	32.1(15)	29.3(12)	20.9(12)	-5.3(10)	-2.7(11)	-8.7(11)
$C_{(15)}$	31.3(14)	21.1(11)	14.1(11)	-3.2(9)	1.2(10)	1.0(10)
$C_{(14)}$	25.4(14)	35.4(14)	28.2(14)	-2.2(11)	-3.1(11)	7.6(11)
$C_{(35)}$	22.9(14)	30.7(13)	30.9(15)	-1.1(11)	4.7(12)	-4.1(11)
$C_{(37)}$	36.3(15)	25.7(12)	25.4(13)	-0.8(10)	2.8(11)	-1.2(11)
$C_{(33)}$	23.2(13)	19.3(11)	20.5(12)	0.6(9)	-2.6(10)	-4.7(10)
$C_{(34)}$	41.0(16)	26.4(13)	32.2(15)	-3.2(11)	-2.3(13)	-16.2(12)
$C_{(12)}$	46.4(18)	34.6(15)	35.7(16)	-6.3(12)	-3.4(14)	20.3(14)
$C_{(36)}$	31.6(15)	27.5(12)	32.2(14)	10.5(11)	-0.3(12)	-1.0(11)
$C_{(23)}$	32.5(14)	22.3(11)	18.5(12)	0.3(9)	4.0(10)	-7.7(10)
$C_{(13)}$	40.5(19)	32.4(14)	49.6(18)	20.0(13)	2.6(14)	3.4(13)
$C_{(38)}$	39.4(16)	26.2(12)	30.7(14)	-0.3(11)	-5.7(12)	2.4(11)
$C_{(32)}$	33.9(15)	34.0(14)	28.2(13)	-	6.6(11)	-3.1(11)

				10.2(11)		
$C_{(16)}$	57.2(19)	30.3(13)	17.6(12)	-3.2(10)	-1.6(12)	4.6(13)
$C_{(30)}$	48.0(17)	31.7(14)	20.1(12)	-6.8(10)	-0.2(11)	-12.6(12)
$C_{(31)}$	47.7(17)	33.7(14)	20.5(12)	0.4(10)	-3.6(12)	-1.2(13)
$C_{(17)}$	39.2(16)	53.7(18)	32.6(15)	-4.1(13)	7.9(13)	-0.8(14)
$C_{(18)}$	54.4(18)	25.3(13)	27.8(13)	-3.7(10)	-4.5(12)	-4.4(12)

Table S7 Bond Lengths for CCDC 1997610.

Atom	Atom	Length/Å	Atom	Atom	Length/ Å
$Mn_{(1)}$	$O_{(1)}$	2.1465(18)	$C_{(1)}$	$C_{(6)}$	1.492(3)
$Mn_{(1)}$	$N_{(1)}$	2.245(2)	$C_{(25)}$	$C_{(26)}$	1.403(3)
$Mn_{(1)}$	$O_{(4)}$	2.1522(18)	$C_{(7)}$	$C_{(6)}$	1.393(3)
$Mn_{(1)}$	$N_{(4)}$	2.228(2)	$C_{(7)}$	$C_{(8)}$	1.397(3)
$Mn_{(1)}$	$N_{(3)}$	2.238(2)	$C_{(19)}$	$C_{(20)}$	1.396(3)
$Mn_{(1)}$	$N_{(2)}$	2.243(2)	$C_{(21)}$	$C_{(20)}$	1.396(3)
$S_{(1)}$	$O_{(1)}$	1.4523(18)	$C_{(21)}$	$C_{(29)}$	1.529(3)
$S_{(1)}$	$O_{(2)}$	1.4401(19)	$C_{(21)}$	$C_{(22)}$	1.397(3)
$S_{(1)}$	$O_{(3)}$	1.433(2)	$C_{(2)}$	$C_{(3)}$	1.399(3)
$S_{(1)}$	$C_{(37)}$	1.830(3)	$C_{(28)}$	$C_{(27)}$	1.374(4)
$S_{(2)}$	$O_{(4)}$	1.4649(18)	$C_{(11)}$	$C_{(3)}$	1.534(3)
$S_{(2)}$	$O_{(6)}$	1.422(2)	$C_{(11)}$	$C_{(14)}$	1.536(4)
$S_{(2)}$	$O_{(5)}$	1.4389(19)	$C_{(11)}$	$C_{(12)}$	1.523(4)
$S_{(2)}$	$C_{(38)}$	1.832(3)	$C_{(11)}$	$C_{(13)}$	1.529(4)
$F_{(2)}$	$C_{(37)}$	1.337(3)	$C_{(29)}$	$C_{(32)}$	1.525(4)
$F_{(1)}$	$C_{(37)}$	1.325(3)	$C_{(29)}$	$C_{(30)}$	1.537(3)
$F_{(4)}$	$C_{(38)}$	1.326(4)	$C_{(29)}$	$C_{(31)}$	1.535(4)
$N_{(1)}$	$C_{(1)}$	1.359(3)	$C_{(5)}$	$C_{(4)}$	1.384(4)
$N_{(1)}$	$C_{(5)}$	1.333(3)	$C_{(3)}$	$C_{(4)}$	1.394(3)
$N_{(4)}$	$C_{(24)}$	1.355(3)	$C_{(10)}$	$C_{(9)}$	1.379(3)
$N_{(4)}$	$C_{(28)}$	1.331(3)	$C_{(9)}$	$C_{(8)}$	1.393(4)
$F_{(5)}$	$C_{(38)}$	1.326(3)	$C_{(8)}$	$C_{(15)}$	1.531(3)
$N_{(3)}$	$C_{(19)}$	1.346(3)	$C_{(22)}$	$C_{(23)}$	1.379(4)
$N_{(3)}$	$C_{(23)}$	1.345(3)	$C_{(26)}$	$C_{(27)}$	1.392(4)
$F_{(3)}$	$C_{(37)}$	1.328(3)	$C_{(26)}$	$C_{(33)}$	1.520(3)
$F_{(6)}$	$C_{(38)}$	1.333(3)	$C_{(15)}$	$C_{(16)}$	1.532(4)
$N_{(2)}$	$C_{(6)}$	1.353(3)	$C_{(15)}$	$C_{(17)}$	1.521(4)
$N_{(2)}$	$C_{(10)}$	1.338(3)	$C_{(15)}$	$C_{(18)}$	1.533(3)

$C_{(24)}$	$C_{(25)}$	1.376(3)	$C_{(35)}$	$C_{(33)}$	1.533(4)
$C_{(24)}$	$C_{(19)}$	1.497(3)	$C_{(33)}$	$C_{(34)}$	1.533(3)
$C_{(1)}$	$C_{(2)}$	1.392(3)	$C_{(33)}$	$C_{(36)}$	1.541(4)

Table S8 Bond Angles for CCDC 1997610.

Atom	Atom	Atom	Angle/°	Atom	Atom	Atom	Angle/°
$O_{(1)}$	$Mn_{(1)}$	$N_{(1)}$	90.64(7)	$N_{(2)}$	$C_{(6)}$	$C_{(7)}$	122.5(2)
$O_{(1)}$	$Mn_{(1)}$	$O_{(4)}$	91.63(7)	$C_{(7)}$	$C_{(6)}$	$C_{(1)}$	122.5(2)
$O_{(1)}$	$Mn_{(1)}$	$N_{(4)}$	164.04(7)	$C_{(21)}$	$C_{(20)}$	$C_{(19)}$	121.2(2)
$O_{(1)}$	$Mn_{(1)}$	$N_{(3)}$	91.21(7)	$N_{(4)}$	$C_{(28)}$	$C_{(27)}$	123.9(2)
$O_{(1)}$	$Mn_{(1)}$	$N_{(2)}$	97.93(7)	$C_{(3)}$	$C_{(11)}$	$C_{(14)}$	110.3(2)
$O_{(4)}$	$Mn_{(1)}$	$N_{(1)}$	163.81(7)	$C_{(12)}$	$C_{(11)}$	$C_{(3)}$	112.0(2)
$O_{(4)}$	$Mn_{(1)}$	$N_{(4)}$	89.22(7)	$C_{(12)}$	$C_{(11)}$	$C_{(14)}$	108.4(2)
$O_{(4)}$	$Mn_{(1)}$	$N_{(3)}$	96.24(7)	$C_{(12)}$	$C_{(11)}$	$C_{(13)}$	109.4(2)
$O_{(4)}$	$Mn_{(1)}$	$N_{(2)}$	92.04(7)	$C_{(13)}$	$C_{(11)}$	$C_{(3)}$	107.3(2)
$N_{(4)}$	$Mn_{(1)}$	$N_{(1)}$	92.98(6)	$C_{(13)}$	$C_{(11)}$	$C_{(14)}$	109.6(2)
$N_{(4)}$	$Mn_{(1)}$	$N_{(3)}$	72.85(7)	$C_{(21)}$	$C_{(29)}$	$C_{(30)}$	112.5(2)
$N_{(4)}$	$Mn_{(1)}$	$N_{(2)}$	97.97(8)	$C_{(21)}$	$C_{(29)}$	$C_{(31)}$	109.7(2)
$N_{(3)}$	$Mn_{(1)}$	$N_{(1)}$	99.74(7)	$C_{(32)}$	$C_{(29)}$	$C_{(21)}$	108.0(2)
$N_{(3)}$	$Mn_{(1)}$	$N_{(2)}$	167.50(7)	$C_{(32)}$	$C_{(29)}$	$C_{(30)}$	107.8(2)
$N_{(2)}$	$Mn_{(1)}$	$N_{(1)}$	71.78(7)	$C_{(32)}$	$C_{(29)}$	$C_{(31)}$	110.2(2)
$O_{(1)}$	$S_{(1)}$	$C_{(37)}$	101.94(12)	$C_{(31)}$	$C_{(29)}$	$C_{(30)}$	108.6(2)
$O_{(2)}$	$S_{(1)}$	$O_{(1)}$	114.03(12)	$N_{(1)}$	$C_{(5)}$	$C_{(4)}$	123.4(2)
$O_{(2)}$	$S_{(1)}$	$C_{(37)}$	104.22(12)	$C_{(2)}$	$C_{(3)}$	$C_{(11)}$	120.9(2)
$O_{(3)}$	$S_{(1)}$	$O_{(1)}$	113.36(11)	$C_{(4)}$	$C_{(3)}$	$C_{(2)}$	116.2(2)
$O_{(3)}$	$S_{(1)}$	$O_{(2)}$	117.06(13)	$C_{(4)}$	$C_{(3)}$	$C_{(11)}$	122.9(2)
$O_{(3)}$	$S_{(1)}$	$C_{(37)}$	103.89(13)	$N_{(2)}$	$C_{(10)}$	$C_{(9)}$	122.8(2)
$O_{(4)}$	$S_{(2)}$	$C_{(38)}$	101.07(12)	$C_{(10)}$	$C_{(9)}$	$C_{(8)}$	120.5(2)
$O_{(6)}$	$S_{(2)}$	$O_{(4)}$	113.57(12)	$C_{(7)}$	$C_{(8)}$	$C_{(15)}$	122.8(2)
$O_{(6)}$	$S_{(2)}$	$O_{(5)}$	116.43(13)	$C_{(9)}$	$C_{(8)}$	$C_{(7)}$	116.7(2)
$O_{(6)}$	$S_{(2)}$	$C_{(38)}$	104.37(12)	$C_{(9)}$	$C_{(8)}$	$C_{(15)}$	120.4(2)
$O_{(5)}$	$S_{(2)}$	$O_{(4)}$	114.59(12)	$C_{(23)}$	$C_{(22)}$	$C_{(21)}$	119.5(2)
$O_{(5)}$	$S_{(2)}$	$C_{(38)}$	104.40(12)	$C_{(25)}$	$C_{(26)}$	$C_{(33)}$	120.5(2)
$S_{(1)}$	$O_{(1)}$	$Mn_{(1)}$	148.76(12)	$C_{(27)}$	$C_{(26)}$	$C_{(25)}$	116.9(2)
$C_{(1)}$	$N_{(1)}$	$Mn_{(1)}$	118.69(16)	$C_{(27)}$	$C_{(26)}$	$C_{(33)}$	122.6(2)
$C_{(5)}$	$N_{(1)}$	$Mn_{(1)}$	123.56(17)	$C_{(5)}$	$C_{(4)}$	$C_{(3)}$	120.3(2)
$C_{(5)}$	$N_{(1)}$	$C_{(1)}$	117.7(2)	$C_{(28)}$	$C_{(27)}$	$C_{(26)}$	119.1(2)

$S_{(2)}$	$O_{(4)}$	$Mn_{(1)}$	147.61(12)	$C_{(8)}$	$C_{(15)}$	$C_{(16)}$	112.8(2)
$C_{(24)}$	$N_{(4)}$	$Mn_{(1)}$	117.72(16)	$C_{(8)}$	$C_{(15)}$	$C_{(18)}$	109.6(2)
$C_{(28)}$	$N_{(4)}$	$Mn_{(1)}$	123.81(17)	$C_{(16)}$	$C_{(15)}$	$C_{(18)}$	108.9(2)
$C_{(28)}$	$N_{(4)}$	$C_{(24)}$	118.0(2)	$C_{(17)}$	$C_{(15)}$	$C_{(8)}$	108.0(2)
$C_{(19)}$	$N_{(3)}$	$Mn_{(1)}$	117.44(15)	$C_{(17)}$	$C_{(15)}$	$C_{(16)}$	108.2(2)
$C_{(23)}$	$N_{(3)}$	$Mn_{(1)}$	124.29(16)	$C_{(17)}$	$C_{(15)}$	$C_{(18)}$	109.3(2)
$C_{(23)}$	$N_{(3)}$	$C_{(19)}$	117.9(2)	$F_{(2)}$	$C_{(37)}$	$S_{(1)}$	111.70(18)
$C_{(6)}$	$N_{(2)}$	$Mn_{(1)}$	118.42(15)	$F_{(1)}$	$C_{(37)}$	$S_{(1)}$	111.09(19)
$C_{(10)}$	$N_{(2)}$	$Mn_{(1)}$	123.06(17)	$F_{(1)}$	$C_{(37)}$	$F_{(2)}$	107.4(2)
$C_{(10)}$	$N_{(2)}$	$C_{(6)}$	117.6(2)	$F_{(1)}$	$C_{(37)}$	$F_{(3)}$	107.6(2)
$N_{(4)}$	$C_{(24)}$	$C_{(25)}$	121.4(2)	$F_{(3)}$	$C_{(37)}$	$S_{(1)}$	110.39(19)
$N_{(4)}$	$C_{(24)}$	$C_{(19)}$	115.0(2)	$F_{(3)}$	$C_{(37)}$	$F_{(2)}$	108.4(2)
$C_{(25)}$	$C_{(24)}$	$C_{(19)}$	123.6(2)	$C_{(26)}$	$C_{(33)}$	$C_{(35)}$	109.07(19)
$N_{(1)}$	$C_{(1)}$	$C_{(2)}$	121.8(2)	$C_{(26)}$	$C_{(33)}$	$C_{(34)}$	112.1(2)
$N_{(1)}$	$C_{(1)}$	$C_{(6)}$	114.6(2)	$C_{(26)}$	$C_{(33)}$	$C_{(36)}$	108.0(2)
$C_{(2)}$	$C_{(1)}$	$C_{(6)}$	123.7(2)	$C_{(35)}$	$C_{(33)}$	$C_{(36)}$	109.8(2)
$C_{(24)}$	$C_{(25)}$	$C_{(26)}$	120.6(2)	$C_{(34)}$	$C_{(33)}$	$C_{(35)}$	108.1(2)
$C_{(6)}$	$C_{(7)}$	$C_{(8)}$	119.7(2)	$C_{(34)}$	$C_{(33)}$	$C_{(36)}$	109.8(2)
$N_{(3)}$	$C_{(19)}$	$C_{(24)}$	115.7(2)	$N_{(3)}$	$C_{(23)}$	$C_{(22)}$	123.8(2)
$N_{(3)}$	$C_{(19)}$	$C_{(20)}$	121.2(2)	$F_{(4)}$	$C_{(38)}$	$S_{(2)}$	111.27(18)
$C_{(20)}$	$C_{(19)}$	$C_{(24)}$	123.1(2)	$F_{(4)}$	$C_{(38)}$	$F_{(6)}$	108.0(2)
$C_{(20)}$	$C_{(21)}$	$C_{(29)}$	120.8(2)	$F_{(5)}$	$C_{(38)}$	$S_{(2)}$	110.77(19)
$C_{(20)}$	$C_{(21)}$	$C_{(22)}$	116.4(2)	$F_{(5)}$	$C_{(38)}$	$F_{(4)}$	107.8(2)
$C_{(22)}$	$C_{(21)}$	$C_{(29)}$	122.7(2)	$F_{(5)}$	$C_{(38)}$	$F_{(6)}$	108.3(2)
$C_{(1)}$	$C_{(2)}$	$C_{(3)}$	120.7(2)	$F_{(6)}$	$C_{(38)}$	$S_{(2)}$	110.6(2)
$N_{(2)}$	$C_{(6)}$	$C_{(1)}$	115.0(2)				

Table S9 Hydrogen Atom Coordinates (Å×10^4) and Isotropic Displacement Parameters (Å^2×10^3) for CCDC 1997610.

Atom	*x*	*y*	*z*	U(eq)
$H_{(25)}$	4710	8440	2854	22
$H_{(7)}$	3753	7286	7666	23
$H_{(2)}$	4767	6524	7136	23
$H_{(20)}$	3680	7666	2350	21
$H_{(28)}$	4362	8591	5339	34
$H_{(5)}$	4114	6349	4664	30
$H_{(10)}$	1617	8662	6130	29

$H_{(9)}$	1639	8878	7359	29
$H_{(22)}$	1285	6190	2674	30
$H_{(4)}$	5471	5640	5218	31
$H_{(27)}$	5681	9295	4756	33
$H_{(14A)}$	7615	6130	6803	45
$H_{(14B)}$	7567	5565	7398	45
$H_{(14C)}$	6640	6166	7456	45
$H_{(35A)}$	7659	8808	3220	42
$H_{(35B)}$	7613	9334	2582	42
$H_{(35C)}$	6698	8725	2563	42
$H_{(34A)}$	6230	10166	4066	50
$H_{(34B)}$	7368	10179	3511	50
$H_{(34C)}$	7339	9658	4154	50
$H_{(12A)}$	6218	4807	5857	58
$H_{(12B)}$	7364	4784	6410	58
$H_{(12C)}$	7302	5332	5796	58
$H_{(36A)}$	4942	9499	2422	46
$H_{(36B)}$	5805	10123	2512	46
$H_{(36C)}$	4651	10018	3044	46
$H_{(23)}$	1247	6464	3889	29
$H_{(13A)}$	4865	5385	7494	61
$H_{(13B)}$	5792	4787	7408	61
$H_{(13C)}$	4669	4877	6847	61
$H_{(32A)}$	4421	7031	1470	48
$H_{(32B)}$	4044	6519	855	48
$H_{(32C)}$	4301	6280	1670	48
$H_{(16A)}$	3023	7303	8837	53
$H_{(16B)}$	3322	7837	9442	53
$H_{(16C)}$	4247	7735	8773	53
$H_{(30A)}$	2229	5746	1653	50
$H_{(30B)}$	2030	6006	841	50
$H_{(30C)}$	1022	6159	1454	50
$H_{(31A)}$	1187	7348	1227	51
$H_{(31B)}$	2126	7181	583	51
$H_{(31C)}$	2521	7684	1203	51
$H_{(17A)}$	844	8556	8469	63
$H_{(17B)}$	1277	8308	9254	63

H(17C)	956	7797	8627	63
H(18A)	4073	8915	8476	54
H(18B)	3159	9001	9153	54
H(18C)	2737	9234	8360	54

9.2 Crystallographic data of bis-μ-O_2-Mn_2 complex

A suitable single crystal of $C_{59}H_{80}F_9Mn_2N_6O_{13}S_3$ [CCDC 2050295] was selected and mounted on a MiTeGen loop using Parabar oil and placed on a 'Bruker D8 Venture' diffractometer. The crystal was kept at 150.0 K during data collection. Using Olex2[72], the structure was solved with the SHELXT[73] structure solution program using Intrinsic Phasing and refined with the ShelXL[74] refinement package using Least Squares minimisation.

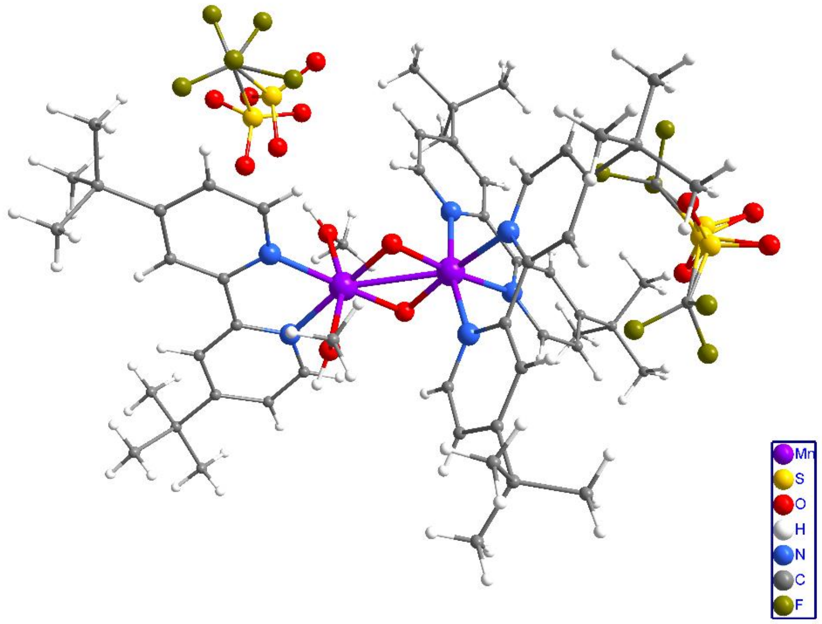

CCDC 2050295 contains the supplementary crystallographic data for this compound. This data can be obtained free of charge from The Cambridge Crystallographic Data Centre via www.ccdc.cam.ac.uk/data_request/cif.

Crystal Data for $C_{59}H_{80}F_9Mn_2N_6O_{13}S_3$ (M =1458.35 g/mol): monoclinic, space group C2/c (no. 15), a = 22.0876(18) Å, b = 17.1700(15) Å, c = 20.5317(17) Å, β = 114.686(3)°, V = 7074.9(10) Å^3, Z = 4, T = 150.0 K, μ(MoKα) = 0.530 mm^{-1}, $Dcalc$ = 1.369 g/cm^3, 47875 reflections measured (4.628° ≤ 2Θ ≤ 52.944°), 7260 unique (R_{int} =

0.0466, R_{sigma} = 0.0314) which were used in all calculations. The final R_1 was 0.0760 (I > 2σ(I)) and wR_2 was 0.2268 (all data).

Table S10 Crystal data and structure refinement for CCDC 2050295.

Identification code	ox_Mn_cmplx_0m
Empirical formula	$C_{59}H_{80}F_9Mn_2N_6O_{13}S_3$
Formula weight	1458.35
Temperature/K	150.0
Crystal system	monoclinic
Space group	C2/c
a/Å	22.0876(18)
b/Å	17.1700(15)
c/Å	20.5317(17)
α/°	90
β/°	114.686(3)
γ/°	90
Volume/Å^3	7074.9(10)
Z	4
ρ_{calc}g/cm^3	1.369
μ/mm^{-1}	0.530
F(000)	3036.0
Crystal size/mm^3	0.1 × 0.08 × 0.05
Radiation	MoKα (λ = 0.71073)
2Θ range for data collection/°	4.628 to 52.944
Index ranges	-27 ≤ h ≤ 27, -21 ≤ k ≤ 21, -25 ≤ l ≤ 25
Reflections collected	47875
Independent reflections	7260 [R_{int} = 0.0466, R_{sigma} = 0.0314]
Data/restraints/parameters	7260/367/516
Goodness-of-fit on F^2	1.035
Final R indexes [I>=2σ (I)]	R_1 = 0.0760, wR_2 = 0.2105
Final R indexes [all data]	R_1 = 0.0901, wR_2 = 0.2268
Largest diff. peak/hole / e Å^{-3}	1.99/-1.27

Table S11 Fractional Atomic Coordinates (×10^4) and Equivalent Isotropic Displacement Parameters (Å^2×10^3) for CCDC 2050295. U_{eq} is defined as 1/3 of of the trace of the orthogonalised U_{IJ} tensor.

Atom	*x*	*y*	*z*	U(eq)
Mn1	5000	6543.9(4)	2500	22.6(2)
Mn2	5000	4991.7(4)	2500	26.6(2)
S2A	3558.3(10)	4233.7(16)	4052.3(13)	40.2(5)
O1	4686.9(12)	5789.5(13)	1845.0(13)	26.1(5)
O2	3983.5(14)	4781.5(17)	2479.6(15)	38.2(6)
N1	5495.4(14)	7404.8(17)	3235.6(15)	24.9(6)
N2	5830.5(15)	6670.4(17)	2335.6(16)	26.2(6)
N3	4743.9(15)	4076.8(18)	1801.8(16)	28.4(6)
C10	5943.4(19)	6290(2)	1825.0(19)	29.8(7)

C5	6117.0(16)	7576.2(19)	3299.9(18)	24.5(7)
C3	6305.8(18)	8463(2)	4274.6(19)	28.6(7)
C9	6532(2)	6371(2)	1751(2)	35.6(9)
C4	6526.3(17)	8095(2)	3805.1(19)	28.6(7)
C2	5666(2)	8294(2)	4188(2)	33.7(8)
C6	6307.4(16)	7166(2)	2777.4(18)	26.3(7)
C1	5280.4(19)	7769(2)	3674(2)	32.1(8)
C7	6903.8(18)	7271(2)	2722(2)	33.2(8)
C23	4861.3(18)	3349(2)	2102.4(19)	29.3(8)
C8	7033.3(19)	6863(3)	2205(2)	36.4(9)
C11	6766.3(19)	9012(2)	4855(2)	35.1(8)
C21	4472.6(18)	2754(2)	933(2)	31.1(8)
C20	4348(2)	3503(2)	642(2)	33.7(8)
C24	4335(2)	2028(2)	465(2)	34.5(8)
C19	4491(2)	4144(2)	1091(2)	33.9(8)
C22	4727.8(18)	2692(2)	1681.2(19)	29.7(7)
C13	7027(2)	9649(2)	4507(3)	44.3(10)
C12	6413(3)	9392(3)	5274(2)	47.6(11)
F6	4649(2)	3950(5)	5153(3)	70.4(16)
C25	4134(2)	2231(3)	-327(2)	45.2(10)
C14	7358(3)	8514(3)	5372(3)	58.0(14)
C27	4960(3)	1519(3)	719(3)	54.1(12)
F4	3797(3)	3604(4)	5275(3)	72.0(15)
C28	3362(2)	4921(3)	1885(3)	48.2(11)
C26	3762(3)	1582(3)	524(3)	49.9(11)
O6	3936(4)	4245(6)	3629(4)	72(3)
O7	2949(3)	3796(5)	3743(4)	52.4(17)
C15	7701(2)	6952(4)	2159(3)	53.9(13)
C17	8253(2)	6735(5)	2890(3)	73.4(19)
C16	7773(3)	7814(4)	1981(3)	71.6(18)
F5	4249(4)	3072(6)	4706(5)	223(5)
C18	7754(3)	6411(5)	1588(3)	81(2)
C30	4067(3)	3819(6)	4790(4)	113(4)
O5	3522(4)	4971(4)	4382(5)	64.3(19)
F4A	3754(4)	4315(7)	5085(5)	79(3)
S2	3503.6(14)	3884(2)	3789.7(16)	44.5(7)
O6A	3941(4)	3718(5)	3459(4)	43.4(17)
O7A	3115(5)	3281(7)	3866(5)	61(2)
O8	3124(6)	4588(8)	3540(8)	111(5)
S1	5000	10146(11)	2500	137(10)
O4	4676(4)	10999(4)	2476(4)	65(2)
O3	5332(4)	9926(4)	3166(4)	153(3)
F2	3648(4)	9812(6)	2297(5)	101(3)
C29	4302(6)	9714(6)	2746(6)	98(6)
F1	4245(5)	8935(3)	2943(6)	161(6)
F3	4283(7)	9926(6)	3387(5)	142(5)
S1A	5118(2)	10227(6)	2467(5)	55(3)
F6A	4455(6)	4703(8)	4724(7)	127(6)

Table S12 Anisotropic Displacement Parameters (Å^2×10^3) for CCDC 2050295. The Anisotropic displacement factor exponent takes the form: -2π^2[h^{2}a*^{2}U$_{11}$+2hka*b*U$_{12}$+…].

Atom	U$_{11}$	U$_{22}$	U$_{33}$	U$_{23}$	U$_{13}$	U$_{12}$
Mn1	22.6(4)	24.0(4)	23.5(4)	0	11.8(3)	0
Mn2	32.6(4)	25.3(4)	23.8(4)	0	13.5(3)	0
S2A	32.3(10)	55.2(14)	34.5(11)	14.5(10)	15.5(9)	1.6(10)
O1	30.4(12)	24.3(12)	25.5(12)	-0.5(9)	13.6(10)	-2.7(9)
O2	36.9(14)	41.3(15)	40.8(15)	7.2(12)	20.7(12)	-1.5(12)
N1	25.4(14)	26.1(14)	25.0(14)	-0.5(11)	12.4(11)	-2.2(11)
N2	29.5(15)	26.0(14)	26.1(14)	1.7(11)	14.6(12)	3.0(11)
N3	33.1(16)	27.4(15)	26.2(14)	-0.6(12)	13.9(12)	-2.3(12)
C10	34.4(18)	31.4(18)	28.4(17)	2.6(14)	17.7(15)	3.4(15)
C5	24.0(16)	24.5(16)	25.0(16)	4.7(13)	10.4(13)	1.0(12)
C3	33.3(18)	22.6(16)	25.0(17)	2.5(13)	7.3(14)	-3.2(13)
C9	40(2)	41(2)	35(2)	8.8(16)	24.5(17)	14.0(17)
C4	26.6(16)	27.7(17)	29.3(17)	3.5(14)	9.4(14)	-1.5(13)
C2	39(2)	33.8(19)	32.0(19)	-8.2(15)	18.0(16)	-4.5(15)
C6	22.8(16)	31.7(18)	25.6(16)	5.7(14)	11.4(13)	2.6(13)
C1	31.5(18)	33.5(19)	36.3(19)	-5.7(15)	19.2(16)	-6.8(15)
C7	24.5(17)	47(2)	28.8(18)	9.5(16)	11.5(14)	1.0(15)
C23	29.5(17)	30.4(18)	29.8(19)	1.3(14)	14.3(15)	-0.6(14)
C8	30.3(19)	51(2)	34.5(19)	16.1(17)	19.8(16)	10.7(17)
C11	36(2)	27.9(18)	33.0(19)	-0.8(15)	5.5(16)	-3.5(15)
C21	30.3(18)	37(2)	28.6(17)	-3.2(15)	14.8(14)	-4.0(15)
C20	39(2)	39(2)	23.0(17)	1.0(15)	12.6(15)	-5.2(16)
C24	38(2)	38(2)	28.6(18)	-6.2(15)	15.5(16)	-4.6(16)
C19	42(2)	32.6(19)	29.0(18)	3.9(15)	16.7(16)	-1.4(15)
C22	32.9(18)	29.4(18)	28.1(18)	-2.3(14)	14.1(15)	-0.6(14)
C13	44(2)	28.2(19)	57(3)	-2.7(18)	17(2)	-10.2(17)
C12	64(3)	38(2)	41(2)	-12.8(18)	23(2)	-14(2)
F6	38(3)	125(5)	46(3)	20(3)	15(2)	23(3)
C25	58(3)	48(2)	31(2)	-9.2(18)	19.2(19)	-14(2)
C14	60(3)	40(2)	39(2)	-2.4(19)	-14(2)	1(2)
C27	57(3)	60(3)	46(3)	-11(2)	21(2)	11(2)
F4	71(3)	108(5)	47(3)	20(3)	34(3)	16(3)
C28	38(2)	52(3)	51(3)	0(2)	15(2)	-4.9(19)
C26	60(3)	44(2)	56(3)	-12(2)	34(2)	-20(2)
O6	47(4)	124(8)	56(4)	44(5)	34(3)	18(5)
O7	27(3)	75(5)	50(4)	-7(4)	11(2)	-4(3)
C15	30(2)	94(4)	46(2)	15(3)	24.4(19)	8(2)
C17	32(2)	134(6)	57(3)	25(3)	22(2)	23(3)
C16	44(3)	112(5)	64(3)	26(3)	29(3)	-17(3)
F5	204(8)	311(11)	233(8)	201(9)	169(7)	181(8)
C18	51(3)	147(6)	68(4)	4(4)	46(3)	18(4)
C30	59(4)	222(11)	63(4)	-61(6)	31(3)	3(5)
O5	59(4)	40(3)	101(6)	6(3)	41(4)	-3(3)
F4A	55(5)	124(9)	68(6)	-63(6)	36(4)	-31(5)
S2	37.9(14)	61.6(19)	37.7(15)	-4.7(13)	19.4(12)	-13.4(13)
O6A	38(4)	54(4)	45(4)	2(4)	24(3)	-8(3)

O7A	53(5)	88(7)	53(5)	-28(5)	32(4)	-45(5)
O8	84(7)	118(9)	170(12)	87(9)	92(8)	64(7)
S1	240(20)	48(7)	46(7)	0	-13(8)	0
O4	106(6)	41(3)	56(4)	-1(3)	42(5)	5(4)
O3	180(7)	113(5)	109(5)	30(4)	5(5)	50(5)
F2	89(6)	97(6)	103(6)	-12(5)	26(5)	5(5)
C29	163(15)	35(6)	48(6)	-6(5)	-5(8)	4(7)
F1	122(7)	15(3)	191(10)	9(4)	-86(7)	2(3)
F3	270(15)	88(6)	73(5)	-32(5)	77(8)	-74(8)
S1A	26(5)	41(3)	72(6)	36(3)	-3(4)	-8.6(18)
F6A	98(7)	164(11)	158(11)	-130(10)	91(8)	-111(8)

Table S13 Bond Lengths for CCDC 2050295.

Atom	Atom	Length/Å	Atom	Atom	Length/Å
Mn1	Mn2	2.6652(10)	C11	C12	1.528(6)
Mn1	O1	1.786(2)	C11	C14	1.552(6)
Mn1	O1[1]	1.786(2)	C21	C20	1.397(5)
Mn1	N1[1]	2.069(3)	C21	C24	1.524(5)
Mn1	N1	2.069(3)	C21	C22	1.403(5)
Mn1	N2[1]	2.011(3)	C20	C19	1.385(6)
Mn1	N2	2.011(3)	C24	C25	1.535(5)
Mn2	O1[1]	1.840(2)	C24	C27	1.530(6)
Mn2	O1	1.840(2)	C24	C26	1.526(6)
Mn2	O2[1]	2.257(3)	F6	C30	1.208(8)
Mn2	O2	2.257(3)	F4	C30	1.407(9)
Mn2	N3	2.041(3)	C15	C17	1.533(7)
Mn2	N3[1]	2.041(3)	C15	C16	1.550(9)
S2A	O6	1.434(7)	C15	C18	1.537(8)
S2A	O7	1.438(7)	F5	C30	1.376(11)
S2A	C30	1.626(9)	C30	F4A	1.384(10)
S2A	O5	1.453(8)	C30	S2	1.910(8)
O2	C28	1.425(5)	C30	F6A	1.776(16)
N1	C5	1.356(4)	S2	O6A	1.423(8)
N1	C1	1.336(5)	S2	O7A	1.395(9)
N2	C10	1.344(5)	S2	O8	1.437(10)
N2	C6	1.363(5)	S1	O4	1.621(18)
N3	C23	1.369(5)	S1	O4[1]	1.621(18)
N3	C19	1.331(5)	S1	O3	1.308(8)
C10	C9	1.378(5)	S1	O3[1]	1.308(8)
C5	C4	1.379(5)	S1	C29	1.957(13)
C5	C6	1.484(5)	S1	C29[1]	1.957(13)
C3	C4	1.399(5)	O4	O4[1]	1.393(17)
C3	C2	1.378(5)	O4	S1A	1.651(13)
C3	C11	1.527(5)	O4	S1A[1]	1.390(12)
C9	C8	1.395(6)	O3	S1A	1.407(11)
C2	C1	1.379(5)	O3	S1A[1]	1.366(12)
C6	C7	1.380(5)	F2	C29	1.361(13)
C7	C8	1.398(6)	C29	F1	1.419(11)
C23	C23[1]	1.485(7)	C29	F3	1.384(12)

C23	C22	1.377(5)	C29	S1A	2.283(14)
C8	C15	1.523(5)	C29	S1A^1	1.752(17)
C11	C13	1.543(6)	S1A	S1A^1	0.592(12)

Table S14 Bond Angles for CCDC 2050295.

Atom	Atom	Atom	Angle/°	Atom	Atom	Atom	Angle/°
O1	Mn1	Mn2	43.49(8)	C20	C21	C22	117.1(3)
O1^1	Mn1	Mn2	43.49(8)	C22	C21	C24	120.8(3)
O1^1	Mn1	O1	86.98(15)	C19	C20	C21	119.8(3)
O1	Mn1	N1^1	92.75(11)	C21	C24	C25	112.1(3)
O1^1	Mn1	N1^1	171.22(11)	C21	C24	C27	109.7(3)
O1	Mn1	N1	171.21(11)	C21	C24	C26	108.3(3)
O1^1	Mn1	N1	92.76(11)	C27	C24	C25	108.2(4)
O1^1	Mn1	N2	96.39(11)	C26	C24	C25	108.4(3)
O1	Mn1	N2	92.60(11)	C26	C24	C27	110.2(4)
O1	Mn1	N2^1	96.39(11)	N3	C19	C20	122.4(3)
O1^1	Mn1	N2^1	92.60(11)	C23	C22	C21	120.6(3)
N1	Mn1	Mn2	135.58(8)	C8	C15	C17	108.1(4)
N1^1	Mn1	Mn2	135.58(8)	C8	C15	C16	107.9(4)
N1	Mn1	N1^1	88.84(16)	C8	C15	C18	111.7(5)
N2	Mn1	Mn2	96.20(8)	C17	C15	C16	110.2(5)
N2^1	Mn1	Mn2	96.20(8)	C17	C15	C18	108.5(5)
N2^1	Mn1	N1	92.40(11)	C18	C15	C16	110.4(5)
N2	Mn1	N1	78.70(12)	F6	C30	S2A	129.5(8)
N2	Mn1	N1^1	92.39(11)	F6	C30	F4	105.7(6)
N2^1	Mn1	N1^1	78.70(12)	F6	C30	F5	87.7(7)
N2	Mn1	N2^1	167.61(17)	F4	C30	S2A	116.2(5)
O1	Mn2	Mn1	41.90(7)	F5	C30	S2A	114.5(5)
O1^1	Mn2	Mn1	41.89(7)	F5	C30	F4	94.3(8)
O1^1	Mn2	O1	83.79(15)	F5	C30	F4A	148.5(10)
O1^1	Mn2	O2	101.53(11)	F5	C30	S2	90.7(5)
O1	Mn2	O2	92.19(11)	F5	C30	F6A	127.7(6)
O1	Mn2	O2^1	101.52(11)	F4A	C30	S2	102.3(6)
O1^1	Mn2	O2^1	92.19(11)	F4A	C30	F6A	81.5(8)
O1	Mn2	N3	98.65(11)	F6A	C30	S2	89.2(7)
O1	Mn2	N3^1	174.61(11)	O6A	S2	C30	103.5(4)
O1^1	Mn2	N3	174.61(11)	O6A	S2	O8	114.2(6)
O1^1	Mn2	N3^1	98.65(11)	O7A	S2	C30	89.5(5)
O2	Mn2	Mn1	99.20(8)	O7A	S2	O6A	118.5(6)
O2^1	Mn2	Mn1	99.20(8)	O7A	S2	O8	111.7(7)
O2	Mn2	O2^1	161.60(16)	O8	S2	C30	117.0(8)
N3	Mn2	Mn1	140.33(9)	O4^1	S1	O4	50.9(8)
N3^1	Mn2	Mn1	140.33(9)	O4^1	S1	C29	136.0(9)
N3	Mn2	O2	83.23(12)	O4^1	S1	C29^1	88.0(5)
N3	Mn2	O2^1	82.63(11)	O4	S1	C29^1	136.0(9)
N3^1	Mn2	O2^1	83.23(12)	O4	S1	C29	88.0(5)
N3^1	Mn2	O2	82.63(11)	O3^1	S1	O4^1	109.9(8)
N3	Mn2	N3^1	79.34(17)	O3	S1	O4	109.9(8)

O6	S2A	O7	114.4(5)
O6	S2A	C30	102.7(4)
O6	S2A	O5	115.4(5)
O7	S2A	C30	109.8(5)
O7	S2A	O5	115.9(4)
O5	S2A	C30	95.6(5)
Mn1	O1	Mn2	94.61(11)
C28	O2	Mn2	125.7(3)
C5	N1	Mn1	115.6(2)
C1	N1	Mn1	126.7(2)
C1	N1	C5	117.6(3)
C10	N2	Mn1	123.9(3)
C10	N2	C6	118.6(3)
C6	N2	Mn1	117.5(2)
C23	N3	Mn2	116.2(2)
C19	N3	Mn2	124.7(3)
C19	N3	C23	119.1(3)
N2	C10	C9	121.9(4)
N1	C5	C4	122.2(3)
N1	C5	C6	114.1(3)
C4	C5	C6	123.8(3)
C4	C3	C11	120.4(3)
C2	C3	C4	116.7(3)
C2	C3	C11	122.9(3)
C10	C9	C8	120.6(4)
C5	C4	C3	120.1(3)
C3	C2	C1	120.6(3)
N2	C6	C5	113.8(3)
N2	C6	C7	121.7(3)
C7	C6	C5	124.4(3)
N1	C1	C2	122.8(3)
C6	C7	C8	120.1(4)
N3	C23	C23^1	114.14(19)
N3	C23	C22	121.0(3)
C22	C23	C23^1	124.9(2)
C9	C8	C7	117.0(3)
C9	C8	C15	122.5(4)
C7	C8	C15	120.5(4)
C3	C11	C13	109.4(3)
C3	C11	C12	111.8(3)
C3	C11	C14	106.8(3)
C13	C11	C14	109.7(4)
C12	C11	C13	109.7(3)
C12	C11	C14	109.4(4)
C20	C21	C24	122.1(3)
O3^1	S1	O4	100.5(8)
O3	S1	O4^1	100.5(8)
O3^1	S1	O3	146.4(17)
O3	S1	C29^1	90.0(7)
O3	S1	C29	77.3(7)
O3^1	S1	C29	90.0(7)
O3^1	S1	C29^1	77.3(6)
C29	S1	C29^1	135.5(12)
O4^1	O4	S1	64.6(4)
O4^1	O4	S1A	53.5(3)
S1A^1	O4	S1A	20.2(4)
S1	O3	S1A^1	13.8(4)
S1A^1	O3	S1A	24.6(5)
F2	C29	S1	120.4(8)
F2	C29	F1	96.5(9)
F2	C29	F3	99.6(12)
F2	C29	S1A	120.6(8)
F2	C29	S1A^1	116.7(9)
F1	C29	S1	127.1(12)
F1	C29	S1A^1	134.8(11)
F1	C29	S1A	127.4(10)
F3	C29	S1	119.1(9)
F3	C29	F1	86.2(9)
F3	C29	S1A^1	114.9(8)
F3	C29	S1A	118.6(9)
S1A^1	C29	S1A	7.51(16)
O4^1	S1A	O4	53.7(7)
O4^1	S1A	O3	107.8(9)
O4^1	S1A	C29	127.7(8)
O4^1	S1A	C29^1	104.6(6)
O4	S1A	C29^1	154.6(6)
O4	S1A	C29	76.9(5)
O3	S1A	O4	103.4(8)
O3^1	S1A	O4^1	121.5(7)
O3^1	S1A	O4	96.6(6)
O3^1	S1A	O3	129.2(8)
O3^1	S1A	C29^1	83.8(8)
O3^1	S1A	C29	75.8(5)
O3	S1A	C29^1	95.8(6)
O3	S1A	C29	64.4(5)
C29^1	S1A	C29	127.2(8)
S1A^1	S1A	O4	54.2(4)
S1A^1	S1A	O3	73.8(18)
S1A^1	S1A	C29	22.8(5)

Table S15 Torsion Angles for CCDC 2050295.

A	B	C	D	Angle/°	A	B	C	D	Angle/°
Mn1	N1	C5	C4	-175.1(3)	C7	C8	C15	C17	56.8(6)

Mn1	N1	C5	C6	5.6(4)
Mn1	N1	C1	C2	174.8(3)
Mn1	N2	C10	C9	-177.3(3)
Mn1	N2	C6	C5	-3.1(4)
Mn1	N2	C6	C7	177.8(3)
Mn2	N3	C23	C23^1	2.0(5)
Mn2	N3	C23	C22	-177.8(3)
Mn2	N3	C19	C20	177.6(3)
O1^1	Mn1	O1	Mn2	0.002(1)
O1^1	Mn2	O1	Mn1	-0.002(0)
O2^1	Mn2	O1	Mn1	-90.98(11)
O2	Mn2	O1	Mn1	101.36(11)
N1^1	Mn1	O1	Mn2	-171.21(11)
N1	C5	C4	C3	0.2(5)
N1	C5	C6	N2	-1.7(4)
N1	C5	C6	C7	177.4(3)
N2^1	Mn1	O1	Mn2	-92.28(12)
N2	Mn1	O1	Mn2	96.27(12)
N2	C10	C9	C8	-0.7(6)
N2	C6	C7	C8	-0.1(5)
N3	Mn2	O1	Mn1	-175.15(11)
N3	C23	C22	C21	0.5(6)
C10	N2	C6	C5	177.6(3)
C10	N2	C6	C7	-1.5(5)
C10	C9	C8	C7	-0.9(6)
C10	C9	C8	C15	178.0(4)
C5	N1	C1	C2	-1.0(5)
C5	C6	C7	C8	-179.2(3)
C3	C2	C1	N1	-0.6(6)
C9	C8	C15	C17	-122.0(5)
C9	C8	C15	C16	118.9(5)
C9	C8	C15	C18	-2.7(6)
C4	C5	C6	N2	179.0(3)
C4	C5	C6	C7	-2.0(5)
C4	C3	C2	C1	1.9(5)
C4	C3	C11	C13	54.5(5)
C4	C3	C11	C12	176.2(3)
C4	C3	C11	C14	-64.2(5)
C2	C3	C4	C5	-1.7(5)
C2	C3	C11	C13	-126.3(4)
C2	C3	C11	C12	-4.6(5)
C2	C3	C11	C14	115.0(4)
C6	N2	C10	C9	1.9(5)
C6	C5	C4	C3	179.4(3)
C6	C7	C8	C9	1.3(5)
C6	C7	C8	C15	-177.6(4)
C1	N1	C5	C4	1.2(5)
C1	N1	C5	C6	-178.1(3)
C7	C8	C15	C16	-62.3(5)
C7	C8	C15	C18	176.1(4)
C23	N3	C19	C20	-0.7(6)
C23^1	C23	C22	C21	-179.2(4)
C11	C3	C4	C5	177.5(3)
C11	C3	C2	C1	-177.3(4)
C21	C20	C19	N3	-0.4(6)
C20	C21	C24	C25	7.1(5)
C20	C21	C24	C27	127.2(4)
C20	C21	C24	C26	-112.5(4)
C20	C21	C22	C23	-1.6(5)
C24	C21	C20	C19	-179.0(3)
C24	C21	C22	C23	179.0(3)
C19	N3	C23	C23^1	-179.6(4)
C19	N3	C23	C22	0.6(5)
C22	C21	C20	C19	1.5(6)
C22	C21	C24	C25	-173.5(4)
C22	C21	C24	C27	-53.4(5)
C22	C21	C24	C26	66.9(5)
O6	S2A	C30	F6	47.5(10)
O6	S2A	C30	F4	-169.6(8)
O6	S2A	C30	F5	-61.1(8)
O7	S2A	C30	F6	169.6(8)
O7	S2A	C30	F4	-47.5(8)
O7	S2A	C30	F5	61.0(7)
O5	S2A	C30	F6	-70.2(8)
O5	S2A	C30	F4	72.7(7)
O5	S2A	C30	F5	-178.8(6)
O4^1	O4	S1A	O3^1	-124.6(9)
O4^1	O4	S1A	O3	102.4(8)
O4^1	O4	S1A	C29^1	-35.3(18)
O4^1	O4	S1A	C29	161.7(8)
O4^1	O4	S1A	S1A^1	161(2)
O3	S1	O4	O4^1	87.4(9)
O3^1	S1	O4	O4^1	-107.2(8)
C29	S1	O4	O4^1	163.1(6)
C29^1	S1	O4	O4^1	-24.7(10)
S1A^1	O4	S1A	O4^1	-161(2)
S1A^1	O4	S1A	O3^1	74.7(18)
S1A^1	O4	S1A	O3	-58.2(19)
S1A^1	O4	S1A	C29^1	164(3)
S1A^1	O4	S1A	C29	1.0(18)
S1A^1	O3	S1A	O4	45.9(7)
S1A^1	O3	S1A	O4^1	101.6(10)
S1A^1	O3	S1A	O3^1	-64.4(9)
S1A^1	O3	S1A	C29	-22.3(7)
S1A^1	O3	S1A	C29^1	-151.0(9)

Table S16 Hydrogen Atom Coordinates (Å×10^4) and Isotropic Displacement Parameters (Å^2×10^3) for CCDC 2050295.

Atom	*x*	*y*	*z*	U(eq)
H2	3916(10)	4540(30)	2816(14)	57
H10	5607.57	5956.24	1505.85	36
H9	6597.19	6088.65	1387.31	43
H4	6958.81	8202.37	3834.36	34
H2A	5489.88	8542.06	4484.35	40
H1	4843.1	7661.52	3630.07	38
H7	7226.99	7621.11	3036.68	40
H20	4164.45	3573.44	136.68	40
H19	4406.11	4649.92	885.46	41
H22	4809.06	2191.73	1899.51	36
H13A	6651	9947.23	4164.37	66
H13B	7270.21	9404.23	4255.51	66
H13C	7327.08	9998.08	4879.24	66
H12A	6713.44	9765.23	5618.11	71
H12B	6284.57	8989.61	5530.74	71
H12C	6013.14	9663.75	4940.47	71
H25A	3718.59	2531.06	-508.65	68
H25B	4067.97	1749.96	-605.07	68
H25C	4486.98	2541.12	-371.62	68
H14A	7601.77	8296.84	5110.06	87
H14B	7187.8	8089.28	5567.23	87
H14C	7657.76	8842.09	5765	87
H27A	5330.96	1821.99	706.28	81
H27B	4875.89	1065.68	403.6	81
H27C	5073.55	1344.12	1210.73	81
H28A	3421.99	5314.6	1571.27	72
H28B	3037.24	5108.8	2059.07	72
H28C	3200.35	4436.25	1616.81	72
H26A	3894.43	1415.93	1021.52	75
H26B	3655.3	1122.99	212.02	75
H26C	3369.93	1919.63	376.89	75
H17A	8684.71	6744.32	2861.24	110
H17B	8170.25	6211.72	3025.41	110
H17C	8257.17	7110.87	3251.32	110
H16A	7758.49	8149.82	2360.34	107
H16B	7407.01	7952.62	1522.49	107
H16C	8199.25	7885.98	1947.44	107
H18A	8190.87	6476.53	1579.79	122
H18B	7403.25	6542.21	1117.21	122
H18C	7700.24	5868.58	1704.42	122

Table 17 Atomic Occupancy for CCDC 2050295.

Atom	*Occupancy*	Atom	*Occupancy*	Atom	*Occupancy*
S2A	0.55	F6	0.6	F4	0.62
O6	0.55	O7	0.55	O5	0.55
F4A	0.38	S2	0.45	O6A	0.45

O7A	0.45	O8	0.45	S1	0.4
O4	0.5	F2	0.5	C29	0.5
F1	0.5	F3	0.5	S1A	0.3
F6A	0.4				

Table S18 Solvent masks information for CCDC 2050295.

Number	X	Y	Z	Volume	Electron count	Content
1	0.193	0.428	0.169	19.4	0.4	?
2	0.193	0.572	0.669	19.4	0.4	?
3	0.250	0.250	0.000	55.1	0.4	?
4	0.250	0.750	0.500	55.1	0.4	?
5	0.307	0.072	0.831	19.4	0.4	?
6	0.307	0.928	0.331	19.4	0.4	?
7	0.693	0.072	0.669	19.4	0.4	?
8	0.693	0.928	0.169	19.4	0.4	?
9	0.750	0.250	0.500	55.1	0.4	?
10	0.750	0.750	0.000	55.1	0.4	?
11	0.807	0.428	0.331	19.4	0.4	?
12	0.807	0.572	0.831	19.4	0.4	?
